# Enteric Viruses Nucleic Acids Distribution along the Digestive Tract of Rhesus Macaques with Idiopathic Chronic Diarrhea

**DOI:** 10.3390/v14030638

**Published:** 2022-03-19

**Authors:** Eric Delwart, David Merriam, Amir Ardeshir, Eda Altan, Yanpeng Li, Xutao Deng, Dennis J. Hartigan-O’Connor

**Affiliations:** 1 Vitalant Research Institute, 270 Masonic Ave., San Francisco, CA 94118, USA; edaltan@hotmail.com (E.A.); alphaleeyp@hotmail.com (Y.L.); xdeng@vitalant.org (X.D.); 2 Department of Laboratory Medicine, University of California, San Francisco, CA 94118, USA; 3 California National Primate Research Center, University of California, Davis, CA 95616, USA; david.merriam@ucdenver.edu (D.M.); aardeshir@ucdavis.edu (A.A.); dhartigan@ucdavis.edu (D.J.H.-O.); 4 Department of Pediatric Infectious Diseases, University of Colorado School of Medicine, Aurora, CO 80045, USA

**Keywords:** idiopathic chronic diarrhea, viral metagenomics, virome, biogeography

## Abstract

Idiopathic chronic diarrhea (ICD) is a little understood common clinical problem in captive rhesus macaques claiming 33% of medical culls unrelated to research. The eukaryotic virome in digestive tract tissues collected at necropsy from nine animals with ICD was characterized using viral metagenomics. We compared the distribution of viral reads in tissues and mucosal scrapings from the stomach, duodenum, jejunum, ileum, and the proximal, transverse, and distal colons. In situ hybridization (ISH) using viral probes were performed on fixed tissues. Deep sequencing revealed multiple viruses in the *Parvoviridae* and *Picornaviridae* family. Tissues and mucosal scraping from the same locations showed closely related viral reads contents while different gut tissues from the same animal varied widely. ISH showed punctuated staining for both RNA and DNA viruses in the distal colon. Parvovirus staining was also detected in the stomach/duodenum/jejunum in distinct oval-shaped structures. The location of enteric viral nucleic acid differed widely between different viral families and along the length of the digestive tract.

## 1. Introduction

Idiopathic chronic diarrhea (ICD) in captive macaques is the most common cause of euthanasia that is not related to medical research in primate research centers, reducing the availability of healthy non-human primates (NHP) for biomedical research [1,2,3,4,5]. Clinically, ICD is seen as persistent or recurring non-bloody diarrhea with microscopic ulcers in the colon [3,6,7,8]. The mucus layer is also thickened, with shortened crypts showing a very high level of lymphocyte and plasma-cell infiltration, typical signs of a response to viral infections. The affected animals tested negative for enteropathogenic agents, including parasitic worms and bacteria (*Shigella*, *Campylobacter*, *Yersinia*, *Salmonella*, and *Clostridium difficile*) and were non-responsive to antibiotic treatments. These clinical observations point to viral infections as possible causes of ICD. This condition resembles human ulcerative colitis, a subset of human inflammatory bowel disease (IBD) affecting the colon and rectum (distinct from IBD’s other major form, Crohn’s disease, which affects the entire GI tract) [9].

We recently described numerous picornaviruses and parvoviruses as well as the less prevalent adenovirus and sapelovirus in the feces of captive rhesus macaques in the US with and without ICD [10]. Several enteroviruses were weakly associated with acute resolving diarrhea and ICD [10]. Healthy captive and wild cynomolgus macaques from Thailand were similarly analyzed revealing a different set of enteric viruses [11].

Here we analyzed the distribution of viral reads along the digestive tracts of nine rhesus macaques that were necropsied following a diagnosis of ICD. Different gut tissues and scrapings of their surfaces were analyzed for viral nucleic acids following enrichment of virus-like particles associated nucleic acid, followed by non-specific amplification of DNA and RNA and next-generation sequencing. Fixed gut tissues were also hybridized with viral probes to identify the anatomical location of viral RNA along the digestive tract.

## 2. Materials and Methods

Sample collection: All animals were born and housed at the CNPRC. Animals were provided with species-appropriate environmental enrichment, fed chow twice daily (LabDiet Monkey Diet 5047, Purina Laboratory, St Louis, MO, USA), offered water without restriction through automatic watering devices, and supplemented with vegetables biweekly.

All rhesus macaques analyzed here were submitted to necropsy due to the primary problem of ICD. Animals were considered to have ICD based on the following criteria: greater than 45 days of recorded diarrhea and/or greater than three hospitalizations for treatment of nonpathogenic diarrhea in the previous six months, tested negative for bacterial and parasitic pathogens in the previous six months, and no etiology for diarrhea identified during microscopic analysis of tissues or at the time of necropsy.

Necropsy: All animals underwent complete necropsies, including cultures of small intestinal and large intestinal contents, and bile for *Campylobacter* spp., *Shigella flexneri*, *Yersinia* species, and *Escherichia* spp., as well as fecal examination by direct microscopy and IFA for *Giardia* and *Cryptosporidium*. The intestinal tract and any gross lesions identified at the time of necropsy were examined microscopically. Routine sections included the stomach, duodenum, jejunum, ileum, proximal colon, transverse colon, and distal colon. Samples were stored at −80 °C until use.

Metagenomic analysis: Tissue and mucosal scraping samples from the stomach, duodenum, ileum, jejunum, proximal colon, transverse colon, and distal colon from nine animals were processed (a total of 126 samples) for metagenomic analysis. Tissues and mucosal scrapings were homogenized with a hand-held rotor in 1ml of PBS buffer with 100 µL of zirconia beads and then rapidly frozen on dry ice and thawed five times before centrifugation at 9000 rpm for 5 min. The supernatants were passed through a 0.45 µm filter (Millipore, Burlington, MA, USA) and filtrates digested for 1.5 h at 37 °C with a mixture of nuclease enzymes consisting of 14U of Turbo DNase (Ambion, Life Technologies, Austin, TX, USA), 3U of Baseline-ZERO (Epicentre, Madison, WI, USA), 30U of Benzonase (Novagen, Madison, WI, USA), and 30U of RNase One (Promega, Madison, WI, USA) in DNase buffer (Ambion, Life Technologies, Austin, TX, USA). We then extracted 150 µL using the MagMAX^TM^ Viral RNA Isolation kit (Applied Biosystems, Life Technologies, Carlsbad, CA, USA), and nucleic acids were resuspended in 50 µL water with 1 µL RiboLock RNAse inhibitor. 11 µL of nucleic acids were incubated for 2 min at 72 °C with 100 pmol of primer A (5′GTTTCCCACTGGANNNNNNNN3′) followed by a reverse transcription step using 200 units of Superscript III (Invitrogen) at 50 °C for 60 min with a subsequent Klenow DNA polymerase step using five units (New England Biolabs, Ipswich, MA, USA) at 37 °C for 60 min. cDNA was then amplified by a PCR step with 35 cycles using AmpliTaq Gold™ DNA polymerase LD with primer A-short (5′GTTTCCCACTGGATA3′) at an annealing temperature of 59 °C. The random amplified products were quantified by a Quant-iT™ DNA HS Assay Kit (Invitrogen, Waltham, MA, USA) using a Qubit fluorometer (Invitrogen, USA) and diluted to 1 ng of DNA for library input. The library was generated using the transposon-based Nextera™ XT Sample Preparation Kit using 15 cycles (Illumina, San Diego, CA, USA), and the concentration of DNA libraries was measured by Quant-iT™ DNA HS Assay Kit. The libraries were pooled at equal concentration and size selected for 300–1000 bp using the Pippin Prep (Sage Science, Beverly, MA, USA). The library was quantified using the KAPA library quantification kit for Illumina platform (Kapa Biosystems, Wilmington, MA, USA) and a 10 pM concentration was loaded on the MiSeq sequencing platform for 2 × 250 cycles pair-end sequencing with dual barcoding.

Bioinformatics: Human and bacterial reads were identified and removed by comparing the raw reads with human reference genome hg38 and bacterial genomes release 66 (collected from ftp://ftp.ncbi.nlm.nih.gov/blast/db/FASTA/, accessed on 20 October 2017) using the local search mode. Low quality sequence ends below Phred 30 scores, as well as adaptor and primer sequences, were trimmed by using VecScreen [12]. The remaining reads were de-duplicated if the base positions 5 to 55 were identical with one random copy retained. The reads were then de novo assembled by Ensemble Assembler [13]. Assembled contigs and all singlet reads were aligned to an in-house viral protein database (collected from ftp://ftp.ncbi.nih.gov/refseq/release/viral/, accessed on 20 October 2017) using BLASTx (version 2.2.7) using an E-value cutoff of 0.01. The significant hits to the virus were then aligned to an in-house non-virus non-redundant (NVNR) universal proteome database using DIAMOND to eliminate the false viral hits [10]. Hits with more significant (lower) E-value to NVNR than to the viral database were removed. Remaining singlets and contigs were compared to all eukaryotic viral protein sequences in GenBank’s non-redundant database using BLASTx The genome coverage of the target viruses was further analyzed by Geneious R11.1.4 software (Biomatters, Auckland, New Zealand).

Enterovirus typing: Enterovirus contigs were typed using the typing tool https://www.rivm.nl/mpf/typingtool/enterovirus/ accessed on 15 February 2020.

Quantitation of viral reads: Genetic variation was observed between homologous sequences belonging to the same enterovirus type. With the goal of assessing the viral abundance of each viral type, closely related contigs (of the same viral type) were concatenated (with 100 bp ‘N’ spacers to prevent reads aligning to the areas where the contigs are joined). These contigs of contigs were then used to find matching reads using the nucleotide aligner program Bowtie2 with the seed length parameter set at 30, and the alignment was considered a hit if the read identity to the reference concatamer was greater than 95%. A total of 11 concatenated contigs representing the most commonly detected viruses were generated. For normalization (to account for the variable number of total reads generated from different samples), the number of matching read hits were converted to reads per million (RPM). Comparing the distribution of RPM to different viruses in different tissues along the gut was done using one-way ANOVA (Kruskal-Wallis test).

In situ hybridization using RNAScope: Custom RNA probes were ordered from ACD (Advanced Cell Diagnostics, Newark, CA, USA) by providing the target information and allowing ACD to design the probe sequences, which are not provided to the customer. The erythroparvovirus, protoparvovirus, and enterovirus 19 probes were respectively based on GenBank accession numbers KT961659, KT961664, and KT961647. Tissues were fixed in 10% neutral buffered formalin between three days and 12 weeks prior to being routinely processed, embedded in paraffin, and sectioned at 5 μm. One section of each animal tissue stained with the RNAscope was also stained with Mayer’s hematoxylin and eosin-Y. An RNA scope was done on a subset of animals, and probes were designed using the metagenomics data of those viruses of interest. Paraffin-embedded tissues were stained using ACD’s RNAScope Fluorescent Multiplex Kit V2 following the manufacturer’s instructions. An equivalent steamer was used instead of the recommended brand in the protocol. A TSA resolution was performed using TSA Cy5 from PerkinElmer, followed by 1 min DAPI incubation and mounting with ProLong Gold. Slides were allowed to cure for at least 24 h before image capture. Images were captured on an Evos2 Auto Fluorescent System. Samples were analyzed on FIJI (Fiji Is Just ImageJ) and utilized FIJI’s built-in macro language for automated segmentation, deconvolution, and quantification. A single section was stained per gut segment and animal. All of the microscopic fields from each stained section were evaluated, therefore integrating information from a wide swath of tissue as quantified using the Auto Fluorescent System.

RNAscope in situ hybridization assays were validated initially using a Human Hela Cell Pellet slide provided by the manufacturer (ACD #31004) as a negative external control. In addition, for each assay subsequently performed, one tissue sample per color was stained using a probe targeting the constitutively expressed gene *PPIB* as a positive control, and a probe targeting *dapB* from *Bacillus* *subtilis* strain SMY as a negative control.

## 3. Results

The goal of this study was to characterize the eukaryotic viruses in different gut tissues of macaques suffering from ICD. Nuclease-resistant (viral particle-associated) nucleic acids were enriched from frozen/thawed tissue biopsies and from matching scraped mucosae acquired during necropsies. A total of seven necropsied tissues, plus mucosal scrapings from the same tissues, from each of nine animals with ICD were analyzed separately. Illumina MiSeq runs were therefore used to analyze 126 samples, yielding a total of 78,985,006 reads, with a range of 19,724 to 2,571,490 reads and an average of 636,975 reads per samples (NCBI BioProject PRJNA607332). A total of 74 contigs greater than 500 bp were generated and grouped into 11 concatamers representing 11 viruses from three different viral families, *Picornavirudae*, *Caliciviridae*, and *Parvoviridae* (Table 1). Further information on the % sequence identity of each contig are in the supplemental data (Table S1). The concatenated sequences can be found in supplemental data (Dataset S1).

**Figure 1:**
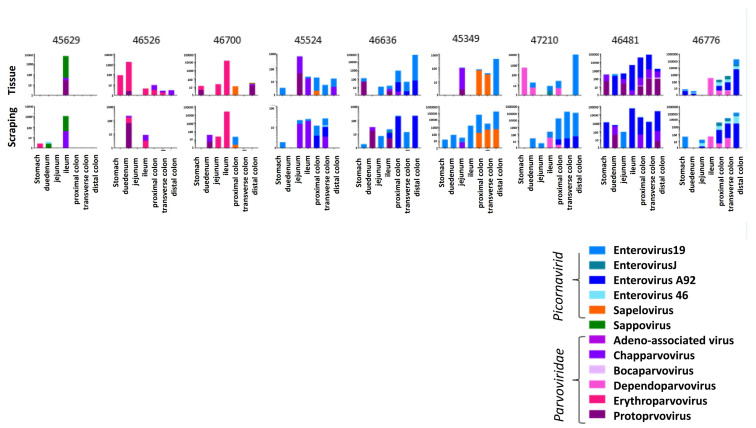
Viruses detected and their abundance in RPM in tissue and mucosal scrapings from the stomach, duodenum, jejunum, ileum, proximal colon, transverse colon, and distal colon of nine macaques with ICD.

These 11 contiguous viral “probes” were then used to quantify matching viral reads in each library (materials and methods). The results are shown as viral reads per million total reads (RPM) (Figure 1). Each animal showed a unique pattern of viruses in tissues and mucosal scrapings (Figure 1). The viral reads from tissues and mucosal scraping were generally although to be not always similar. For example, in animal #45629, two viruses predominated which were mainly detected in the ileum tissue biopsy and the corresponding mucosal scraping.

The following viruses were detected in the 9 ICD animals. In the *Picornaviridae* family (enterovirus B species), we detected enterovirus 19 and 46 in seven animals each and the enterovirus A92 in five macaques. Enterovirus J was found in two and sapelovirus in three animals. Sapovirus in the *Caliciviridae* family was found in one animal. In the *Parvoviridae* family we found chapparvovirus in seven, protoparvovirus in six, erythroparvovirus in three, dependoparvovirus in two, and bocaparvovirus in one animal. The viral pattern observed by viral metagenomics was generally concordant between the biopsied tissues and the overlying mucosal scrapings, indicating that the viruses within the mucosal layer largely reflected those in the underlying tissues.

**Figure 2:**
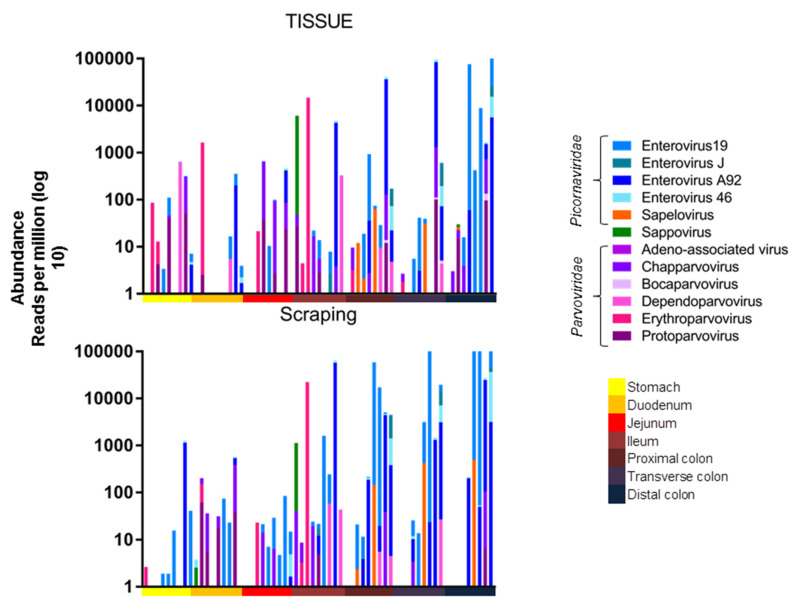
Abundance of viral reads in RPM along length of the digestive tract. Top panel: Abundance of eukaryotic viral reads from tissues along the intestinal tract for all animals. Bottom panel: Abundance of eukaryotic viral reads from mucosal scrapings along the intestinal tract for all animals. Blue shading represents different enteroviruses, while shades of pink or purple represent different *parvoviruses*.

The viral abundance of the different viruses from these animals were then plotted by anatomical location. A higher number of reads was observed in the lower intestines, which were dominated by enteroviruses (Figure 2).

**Figure 3:**
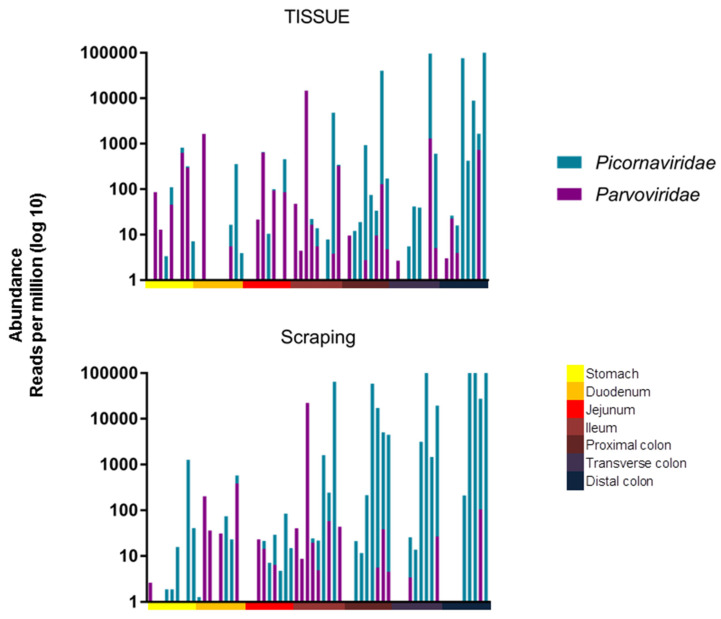
Abundance of picornavirus and parvovirus reads in RPM along the length of the digestive tract. Top panel: Abundance of eukaryotic viral reads from tissues along the intestinal tract for all animals. Bottom panel: Abundance of eukaryotic viral reads from mucosal scraping along the intestinal tract for all animals. Shades of blue represent different enteroviruses, while shades of pink or purple represent different *parvoviruses*.

A one-way ANOVA (Kruskall-Wallis test) was used to compare the total viral read abundance of the tissues and mucosal scrapings along the gastrointestinal tract, resulting in a *p*-values of *p* < 0.0001 and *p* = 0.0017, respectively. The viral RPM values therefore increased towards the end of the digestive tract for both tissues and mucosal scrapings. The read abundance was also plotted after amalgamating reads of the different picornaviruses and different parvoviruses (Figure 3).

**Figure 4:**
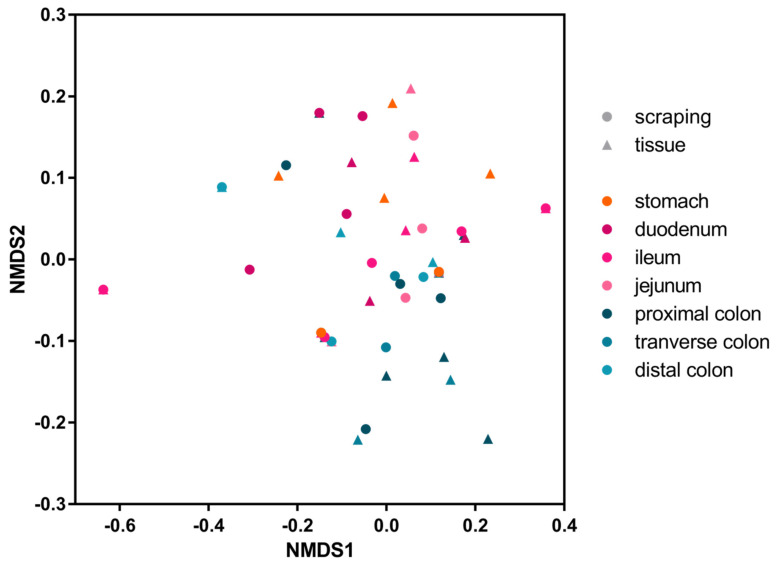
MDS plot of a Bray-Curtis dissimilarity matrix. The large intestine tissues are represented in shades of blue, the small intestine tissues are in shades of pink, and the stomach is in orange. Circles represent mucosal scrapings, while triangles represent tissue samples.

The same statistical test was used to show that there was a significant difference in their distribution, with parvoviruses being found further up the digestive tract than picornaviruses when analyzing mucosal scraping (*p* = 0.0003), but there was only a trend when analyzing tissue biopsies (*p* = 0.0856). To assess the similarity, measured by viral presence and abundance, of viruses at each location of the gastrointestinal tract, a Bray-Curtis dissimilarity matrix was created and plotted as a multidimensional scale (MDS) (Figure 4). The MDS plot shows that the profile of viral reads in the stomach and small intestine (reddish colors) clustered differently than those in the large intestine (blueish colors). A two-tailed Wilcoxon rank sum test for differential clustering of viral reads in these two regions of the digestive tract yielded a *p* value of 0.00064.

Based on the abundance of different viruses, this clustering was likely driven by the higher abundance of picornaviruses in the lower intestinal tract.

**Figure 5:**
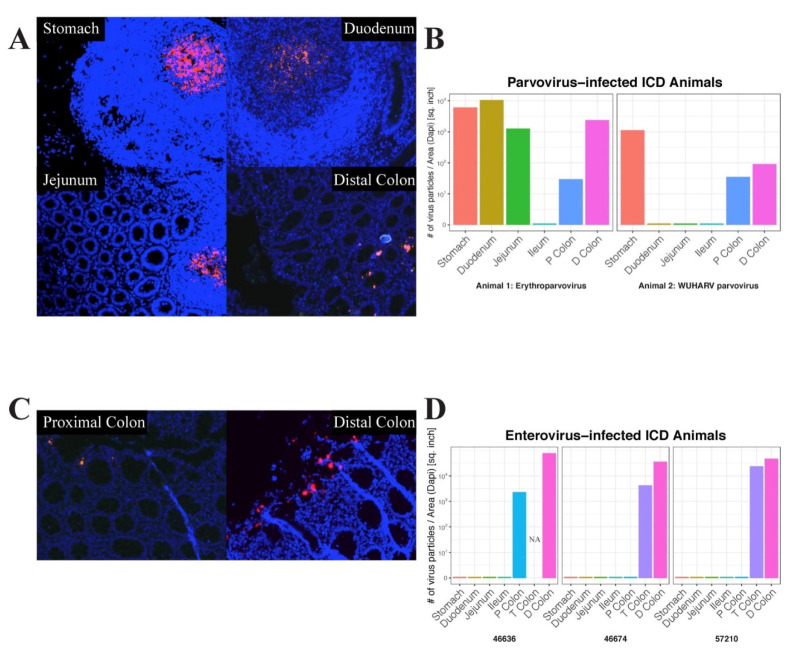
Representative positive microscope fields of different gut tissues hybridized with RNAScope viral probes. (**A**) Erythroparvovirus probes hybridized with 46526 tissues. (**B**) Quantitation of fluorescent signal of erythroparvovirus RNA in 46526 (left frame) and protoparvovirus RNA probe in 46700 (right frame). (**C**) Enterovirus 19 probe hybridized with 46636 tissues. (**D**) Quantitation of RNAScope signal for enterovirus 19 RNA in 46636 (left frame), 46774 (middle frame), and 57210 (right frame).

In situ hybridization was next used to locate the anatomical sites of viral RNA by annealing fluorescently labeled RNAScope probes to fixed gut tissues. Parvovirus RNA was used to probe different tissues from the digestive tracts of two animals and enterovirus RNA was used to probe the same tissues from three animals. Images of positive microscopic fields in different tissues are shown (Figure 5A,C).

The signal distribution of erythroparvovirus RNA was restricted to oval-shaped structures in the stomach, duodenum, and jejunum which were reminiscent of Peyer’s patches. Distinct and more punctuated staining was also seen in the distal colon (Figure 5A). When the erythroparvovirus ISH signal was quantified, it similarly showed high staining in the stomach, duodenum, and jejunum tissue and then in the lower transverse and distal colons (Figure 5B left frame). The distribution of protoparvovirus RNA in another animal showed a similar pattern of staining in the stomach and the lower colon tissues only (Figure 5B right frame). The distribution of enterovirus 19 RNA was also probed in three animals: 46636, 46776, and 57210 (Figure 5D). For enteroviruses, only a punctuated distribution of fluorescent signal was detected in the proximal, transverse, and distal colons, i.e., exclusively in the lower digestive tract (Figure 5C).

Fluorescent signal quantitation therefore supported the visualized signal distribution as well as the results of the deep sequencing, indicating a greater viral signal in the upper digestive tract, particularly the stomach, for parvoviruses and in the lower tract, particularly the distal colon, for picornaviruses.

## 4. Discussion

We show here, using viral metagenomics and in situ hybridization, that the distribution of viral nucleic acids differs along the length of the digestive tract. Enterovirus reads were found at a greater frequency in the lower parts of the colon than in the upper digestive tract. The converse was observed for parvovirus reads, which were more common in the upper digestive tract. The viral reads from the mucosal surface scrapings largely tracked those of the underlying tissues. A subset of animals and viruses were also analyzed using in situ hybridization to locate viral nucleic acids in fixed tissues. The metagenomics and the ISH results were broadly concordant with strong erythroparvovirus staining in the stomach, jejunum, and duodenum, and for protoparvovirus, in the stomach of another animal in structures evocative of Peyer’s patches. Parvovirus staining was also seen in the distal colon but in a more punctuated form, possibly reflecting viral particle production at the tip of villi.

A previous study of rhesus macaques with ICD reported that the lumen contents of the proximal colon, distal colon, and rectum showed more picornavirus reads than in the more upstream terminal ileum [14]. Here picornavirus reads were more abundant in the colon, especially the terminal and distal colon, while very few reads or RNA staining were detected in the ileum or further upstream in the digestive tract, consistent with this prior study. This study also reported no difference in parvovirus reads distributions from the ileum down [14]. Here we found parvovirus reads (and ISH staining) starting higher up in the digestive tract, including the stomach, duodenum, and jejunum, anatomical sites not analyzed in the prior study. Parvovirus reads were also detected in the proximal and distal colon but not in the ileum. The distribution of parvovirus ISH signals in different locations was also highly distinct, with the terminal colon showing punctuated staining while the staining in the stomach/duodenum/jejunum was distributed in large oval structures. The more punctuated ISH signals seen in the lower intestine may reflect virus replication in highly permissive cells. The nature of these oval structures remains uncertain but could consist of gut-associated lymphoid tissues (GALT) in Peyer’s patches, where parvovirus particles may be presented to B lymphocytes.

We therefore show that in rhesus macaques with idiopathic chronic diarrhea, viral nucleic acids are unevenly distributed along the length of the digestive tract. The viral reads in mucosal scrapings largely reflect those in the underlying tissues. Parvovirus RNA was found in oval-shaped structures from the stomach to the jejunum, while both picornavirus and parvovirus RNA were also found in the lower part of the large intestine in a different, more punctuated, staining pattern.

Future experiments suggested by these results include the co-staining of ISH positive cells for cellular differentiation markers to better understand the cellular tropisms of macaque enteric viruses. Whether anatomical regions of viral replication, as reflected by ISH staining, also overlap with the microscopic ulcers typical of ICD will also require further studies.

## Figures and Tables

**Table 1:** The number of contigs per viral species included in the concatamers used for virus read quantitation using Bowtie.

Family	Genus	Species	Type	No. of Contigs
*Caliciviridae*	Sappovirus	Sapporo virus		2
*Picornaviridae*	Enterovirus	A	SV19/EV-A122	23
*Picornaviridae*	Enterovirus	A	SV46/EV-A124	5
*Picornaviridae*	Enterovirus	A	EV-A92	9
*Picornaviridae*	Enterovirus	J	EV-J103	9
*Picornaviridae*	Sapelovirus	B		7
*Parvoviridae*	Bocaparvovirus	Unclassified		1
*Parvoviridae*	Protoparvovirus	Unclassified	WUHARV	2
*Parvoviridae*	Chaphamaparvovirus	Unclassified		8
*Parvoviridae*	Dependoparvovirus	Adeno-associated virus 11		2
*Parvoviridae*	Erythroparvovirus	Unclassified		4

## Data Availability

Illumina sequence datasets generated for this study are available under NCBI BioProject PRJNA607332. All data generated or analysed during this study are included in this published article and its supplementary information files.

## Supplementary Materials

The following are available online at https://www.mdpi.com/article/10.3390/v14030638/s1, Table S1: Composition of concatamers used to quantify reads matching different viral species and genotypes. Dataset S1: The concatenated sequences.

## Abbreviations

ICD	Idiopathic chronic diarrhea
ISH	In situ hybridization
NHP	Non-human primates
GI	Gastro-intestinal
IBD	Inflammatory bowel disease
CNPRC	California National Primate Research Center
AAALAC	Association for Assessment and Accreditation of Laboratory Animal Care
IACUC	Institutional Animal Care and Use Committee
RPM	Reads per million
NVNR	Non-virus non-redundant
MDS	Muti-dimensional scale
GALT	Gut-associated lymphoid tissue
