# Supplementary material for: Enteric Viruses Nucleic Acids Distribution along the Digestive Tract of Rhesus Macaques with Idiopathic Chronic Diarrhea
Source: Viruses. 2022 Mar 19;14(3):638. doi: 10.3390/v14030638 (PMC8951234; doi:10.3390/v14030638)
Supplement: Supplementary file 1 [file viruses-14-00638-s001.zip › Dataset S1.pdf]

>Sappovirus
CAAGCATTTGCATCGCAGTTTGGCTGCCACCCCCACATTCAAAGATAAGGGTTTGCTGGACTCATTCCTT
TCAAAGCCACCTGTTGACATCAATCCTGATACAACATTCCGTGAGCTATTCGGCATTGACCCACACGAAC
AATTCCCATTGTCCATCCACGATTTGGCAAGATTACAGGGTGAGCTTGTGGATGCGGCACGTAACCCAGG
CCACGTGTTGCGTCGCCATTACTCCACCGATTCGCTCACCGCCCTAATCAACAAAATCACAAAATACGTC
CCTGTGCATGCCACACTCCAGGAAATGCAAGCACGCAGAGCTTTCGAGCGAGAGCGCGCGGAGCTGTTCA
AGGAACTGCCACATGCCGACTTGGACGTGAGTCGTCAACAGAAGTCGTACTTTTATGCCATGTGGCGTCA
GGTGGTCAAGAAGGGCAAGGAGTTTTTCATCCCTTTGGTCAAGTGTACATCTTGGCGTAAGAAATTCACA
GAGCCTGCGGAGATTGTTAGACAGGTGCTGGTTCACTTCTGTGAAGGGATGAGATCACAGTTTTCCACCA
ATGCAAATTACATCAACTTGTCCCTTATCGCCAAACTTCGACCAACAGTCCTCACAATGATTCTTCAGCA
ACATAAGAACACCTACAGGGGGTGGTTGGCAACAGTCACAGCTCTTGTTGAGGTATACTCTAATCTGTTC
CAGGACATGCGAGACACTGCAGTGTCAGCAGTGTCGGCCATTACATTAGTGTTTGGAACCATCAAGGATT
TTGTGGTCAATGTGATAGACTTAGTTAAGAGCACGTTTCAGCCACAAGGTCCAACATCTTGCGGCTGGGC
TGCCATCATTGCTGGCGCAGTGCTTATCCTAATGAAGCTGTCAGGGTGCTCCAACACCACGAGCTATTGG
CACCGACTCCTCAAGGTGTGCGGGGGTGTCACTACCATTGCTGCAGCGGCCCGCGCTGTCGTGTGGGTGC
GTGACATCATAGCAGAAGCTGATGGCAAAGCAAGACTGAAGAAGTACATGGCTCGCACAGCAGCCCTGCT
TGAGCTTGCAGCATCCCGAGATGTAACCGGCACTGACGAACTCAAGCGTTTGTTAGATTGTTTTACACAA
CTTATTGAGGAGGGCACTGAGCTGATACAGGAATTTGGCACATCACCCCTTGCTGGTCTGACTAGATCAT
ATGTGAGTGAACTTGAGTCCACTGCAAACAGTATTAGAAGCACCATCCTTCTGGACACACCCCGGAAGGT
TCCGGTCGCGATTATCTTGACTGGCCCCCCTGGAATAGGCAAAACAAGGCTTGCACAGCATCTTGCTGCA
GGGTTTGGTAAAGTGTCAAACTTTTCCGTCACGTTAGACCACCATGACTCATACACCGGGAATGAGGTCG
CGATTTGGGATGAATTTGACGTGGATACACAGGGTAAATTTGTGGAGACGATGATTGGCATAGTCAACAC
CGCCCCCTACCCACTCAATTGTGACCGAGTGGAGAACAAAGGTAAGGTGTTTACATCTGAATACATCATA
TGTACCAGCAATTACCCAACTTCTGTGTTGCCTGACAACCCACGGGCGGGAGCGTTTTATCGCCGGGTCA
CCACGATAGATGTGTCATCCCCAACTATTGAAGACTGGAAAAAGAAAAACCCAGGGAAGAAACCCCCTCC
TGATTTGTACAAGAATGATTTCACACACCTTCGCCTATCTGTTAGACCATTCTTGGGGTACAACCCAGAG
GGAGACACCTTAGATGGTGTCCGGGTCAAGCCTGTGCTTACCAGTGTGGATGGGCTGTCACGCTTGATGG
AGACCAAATTCAAGGAACAGGGCAATGAACAACGGAACTTGTGGATAACATGCCCGCGAGACCTTGTAGC
CCCCGCTGCGTCCGGCTTGAAGGCATACATGGCTGCCAATCGAGCACTTGCACAAGTGTTTCAGGAACCA
TCTTCTCAGGATATTGGCGAAACTTGCACGTCCCGTGTGTATGTGTCGTGTAACAATCCACCCCCCACGT
ACAGTGGGCGGGTGGTGAAGATCACGGCCATCAATCCGTGGGATGCGTCACTTGCTAGTTCCATGTTGTC
AATGTTTGAGACCACCAGCCACATTCCTGCTTCAATCCAACGTGAAATCATGTACAGGGTTTGGGACCCA
CTGGTCCACTTACAGACACGTGAACCAAACACGCAGATGCTCCCTTATATCAACAGGGTGGTCCCAGTGT
CTTCTGCATTCGACTTCATCCGAGGCCTTAGGCACCATCTTGGCCTTTGTTCAGTCAAGGGTATGTGGAG
AGCTTACCAAGGTTGGAACAGTTCTAGTTCAATCTTGGAATTTTTGTCAAAACATATGGCTGATGTTGCC
TTCCCACACAACCCCGAGTGCACCGTTTTTCGAGCCCCAGACGGTGATGTCATCTTTTACACATTTGGGT
CTTACGCTTGCTTTGTGTCCCCAGCCCGCGTCCCATTCGTTGGGGAGCCCCCAAAGAACGTGCATTCAAA
CATAACACGCAATAAGACATGGGCCGAGACACTTCGTCTGCTGGCAGAGACCATAACTGAAAGCCTGGTA
CACTTTGGCCCCTTCTTACTCATGATGCACAATGTCTCATATCTTGCCACCCGGTCTGGTCGAGAGGAGG
AGGCCAAAGGGAAAACCAAACATGGCCGTGGCACCAAACACGCCAGGAGGGGAGGTGTTAGCTTGTCTGA
TGATGAGTATGATGAGTGGCGTGATCTGGTGCGAGACTGGCGGCAGGACATGACTGTTGGGGAGTTTGTG
GAACTTCGTGAGCGATACGCGCTTGGAATGGACTCTGAGGATGTGCAACGTTATCGTGCTTGGCTAGAGC
TACGAGCAATGCGCATGGGTGCAGGTGCCTACCAACACGCCACTATCATTGGAAGAGGGGGGGTGCAAGA
CACCATCATTCGCACCCAACCAATGCGTGCTCCACGCGCGCCCCGGGGTCAAGGCTATGACGAAGAGGCT
CCCACGCCCATTGTCACATTCACATCTGGGGGTGATCACATTGGGTATGGTTGTCACATGGGTAATGGTG
TGGTTGTCACAGTCACTCATGTTGCCTCCGCATCCGATCAAGTGGAGGGACAGGATTTCACGATCAGGAA
GACTGAGGGTGAGACCACATGGGTGAACACCAACCTTGGACATTTACCCCATTACCAGGTTGGTGACGGC
GCTCCTGTCTACTACTCGGCACGCCTACACCCTGTGACTGCGCTCGCAGAGGGCACGTATGAAACACCTA
ACATCACGGTCCAGGGATACCACCTGCGCATTTTAAATGGGTACCCAACAAAGCGCGGGGATTGTGGCAC
ACCCTACTTTGACTCATGCCGCCGTCTGGTTGGTCTCCACGCCGCCACATCAACAAATGGGGAAACCAAA

CTTGCCCAACGTGTGACCAAAACATCCAAGGTGGAGAATGCTTTTGCTTGGAAGGGCCTTGCGGTTGTCC
GGGGTCCCGACTGTGGTGGCATGCCCACAGGGACACGTTACCACCGCTCACCCGCATGGCCCAACCCCGT
GGAAGGAGAAACACATGCCCCTGCGCCGTTTGGCTCCGGTGATGAACGGTACAAGTTCTCCCAAGTGGAG
ATGTTGGTCAATGGTTTGAAGCCTTACTCAGAACCCACCCCTGTTGGGATGAGGACAAGCAGTGTTATAC
TGGTGTGTTGGCGGAACACCTTGCCAAAGCTTGGGATGCAGCCAACAGGGGCGTTGCACCCCAGAACGCC
TATAAATTAGCTTTAAAAGATGAGTTGAGACCAATTGAAAAGAATGCACAAGGAAAAAGGCGCCTTCTGT
GGGGGTGTGACGCGGGTGCCACTTTGGTGGCCACCGCGGCCTTCAAGGGAGTTGCGACCCGTCTCCAAGC
GGTTGCCCCAATGACTCCAGTTGGTGTTGGTATAAACATGGACAGTTACCAGGTTGAGGTGTTGAATGAG
TCACTCAAAGGTGGAGTGCTTTACTGTCTTGATTACAGCAAATGGGACTCAACACAACACCCCGCCGTCA
CGGCCGCCTCACTTGGAATTTTAGAAAGACTGTCTGAAGCTACTCCTATCACAACATCAGCTGTTGAGTT
ACTGTCCTCCCCTGCAAGAGGCCATTTGAATGACATTGTGTTTATCACTAAATCTGGTCTTCCATCTGGT
ATGCCATTCACAAGTGTCATCAACTCACTCAACCATATGACATACTTCGCAGCCGCAGTACTGAAGGCAT
ATGAACAACATGGAGCACCATACACGGGCAACGTGTTTCAGGTGGAGACTGTTCACACCTACGGGGATGA
CTGCTTATACTCGGTGTGCCCTGCTACTGCTTCCATTTTCCAAACAGTTTTGGCCAATTTGACTTCTTTT
GGCCTTAAGCCAACTGCTGCTGACAAGAGTGAGACAATAGCCCCCACCCACACTCCTGTTTTCCTCAAGA
GGACGCTGACCTGCACACCACGTGGCGTGCGTGGCCTGTTGGACATCACATCCATAAAGAGACAATTCTT
GTGGATCAAGGCCAATAGGACGGTTGACATCAATTCACCACCGGCGTACGATCGCGACGCGCGCAGTATC
CAGTTGGAAAATGCCCTTGCGTACGCGTCACAGCATGGTCATGCAGTTTTTGAGGAGGTTGCTGAGTTGG
CACGCAACACAGCCAAGGCTGAGGGACTAGTGCTAACTAATGTCAATTATGACCAGGCTCTCGCCACCTA
CGAATCTTGGTTCATAGGCGGTACAGGCCTGGTGCAAGGCAGCCCCAGTGAAGAGACCACCAAATTAGTG
TTTGAAATGGAGGGTGTATCCCGCCCAGAGGGCCCTAAGGCCAATTCCAATGAAAATGTGCCTCTTGCAT
CACCACAGGACACAATTGGACCAAACGCAGCACTTCTCCTGCCTACGCAGATTGAGACACCCAACGGAGC
TGCGCAAAGGGTGGAGATGGCCGCAGCAACAGGGGCCATTAGCAACAACGTTCCCATGTGTGTGAGGGAA
TGTTTTGCTAGTGTGACCACGCTCCCTTGGACAACGCGCCAAGCATCCAACACCTTTCTTGGTGCCATCC
ATCTCGGTCCACGCATCAACCCCTACACCGCACACTTGAGTGCCATGTTTGCTGGCTGGGGTGGAAGTTT
CCAAATACGAGTTACCATATCTGGCTCTGGGCTGTATGCAGGCCGTGCAGTGGTCGCAGTGCTGCCTCCA
GGTGTCAACCCAGCCAATGTCCAAAATCCTGGAGTGTTCCCACATGCATTCATTGATGCCCGTACCGTGG
ATCCTATCCTCATCAACCTCCCTGACATCCGTGCAGTGGACTACCATCGTGTGGACGGCGATGAGCAAAC
TGCAACTGTTGGACTTTGGGTCGCCCAACCGCTTATCAACCCGTTCCAAGCTGGCTCCATTTCCACATGT
TGGTTGACATTTGAGACCAGACCTGGACCTGATTTTGACTTTTGTCTTCTCAAGGCCCCAGAGCAGGAAA
TGGATAACGGCATATCACCTGCCAACTTGCTCCCCCGCCGCCTTGGACGGTCACGAGGCAATAGGCTCGG
TGGCCGGGTGGTGGGCCTCGTGGTGGTGGCCGTTGCAGAACAAGTTAACCATCACTTTGCTGCCAACTCC
ACCACTCTTGGTTGGTCAACACTCCCAATTGAGCCCATTGCTGGTGCTATATCCTGGTTTCAAAACACCA
CGCCGGGCATTAGCACGCGCGGCCTGCTCAGTGCTGAAGGTAAAGGCATCATCTTCCCAAACATAGTTAA
CCACTGGACTGACGTTGCTCTCTCCAGCAAGACCTCCGGGAGGACTACTGTACCAACTGATCAAGCCAAC
CTCAACCTGTGTCCTGGAGCCTCTGGTCCCGTTGTGATGTTCCAAAATAATGGAGATGTGAATGAGGCTA
GTGCCAACAACTGTGTGTTAACAGCTGCTTCACATGATTTTGTTAACTTGTCTAGCAACTTTGATGCGGC
TGGTATGTGGGTCTGGCTTCCCTGGACAACCACCAAGCCTGACGCCGCAATAAACCGCAATGTCTACATC
ACTCCAACTTGGATCAATGGTGACCCCGCTCGACCAATCCATGGCAAGTGTACCAACATGGTTGGAACCA
ACTTTCAATTTGGTGGCACTGGTACCAACAACATCATGCTCTGGCAAGAGCAGCATTTCACCTCTTATCC
TGGAGCAGCAGAAGTTTACTGTTCACAACTTGAGAGCACGGCTGAGATGTTCCAGAACAATGTTGTCAAT
ATACCAGCCAACCAGATGGCTGTGTTCAATGTGGAAACTGCAGGCAATACATTCCAAATTGCTATCATGC
CAAATGGTTACTGTGTCACCAACGCAGCCGTTGGTACCCATCAACTTCTGGATTACGAGACAAGTTTTAG
GTTTGTTGGACTTTTCCCCCAAAGCACTAGTCTTCAGGGGCCCAATGGGAACGCTGGACGGGCCGTCAGA
TTCCTTGAATAATGTCTTGGTTTACAGGAGCAGCTCTTGCAGCCGGGTCCCTTGTGGACATGGCAGGCAC
TGTATCATCCATTGTGGCACAACATCGCCAAATTGACTTGATGGCACAAGCAAACCAAATCCAAAGAGAC
TGGGTCAACAAGCAAGAGGCACTCATGAGGCGAGGTCAGGACATCTCACGTGACCTTGCGGTCAACGGCA
CCGCCCAACGGGTGGAGTCGTTAGTAGGTGCGGGTTTCACCCCTGTTGATGCTCGACGGCTGGCTGGGAG
CTCAGAAACAGTTCAGTATGGCCTGTTGGACCGCCCAATTCTACAGCGGGAAGTCCTGGCAGGCATTAGT
GAAACACACCACCTTCAATCCATGCAAGCTTCTCTGAGTGCATTTAAGAAAGGATCCAGTTATGGTGCCC

CACCTGCCCCTGTTGGGTTTGCCAGCCCAAATTTCCAACCAGCTCCCCCCAGGGTTAATTTAGGATTCAG
GCCCCCATATATCATGTTTAGGATATTAATCTTTCCTCCCTCTTACAATCAACTTTATTTTCTTTTCTTT
TCCAAGTGGTATCCTNNNNNNNNNNNNNNNNNNNNNNNNNNNNNNNNNNNNNNNNNNNNNNNNNNNNNNN
NNNNNNNNNNNNNNNNNNNNNNNNNNNNNNNNNNNNNNNNNNNNNNNNNNNNNNNNNNNNNNNNNNNNNN
NNNNNNNNNNNNNNNNNNNNNNNNNNNNGCTGCCAATCGAGCACTTGCACAAGTGTTTCAGGAACCATCT
TCTCAGGATATTGGCGAAACTTGCACGTCCCGTGTATATGTGTCGTGTAACAATCCACCCCCCACGTACA
GTGGGCGGGTGGTGAAGATCACGGCCATCAATCCGTGGGATGCGTCACTTGCTAGTTCCATGTTGTCAAT
GTTTGAGACCACCAGCCACATTCCTGCTTCAATCCAACGTGAAATCATGTACAGGGTTTGGGACCCACTG
GTCCACTTACAGACACGTGAACCAAACACGCAGATGCTCCCTTATATCAACAGGGTGGTCCCAGTGTCTT
CTGCATTCGACTTCATCCGAGGCCTTAGGCACCATCTTGGCCTTTGTTCAGTCAAGGGTATGTGGAGAGC
TTACCAAGGTTGGAACAGTTCTAGTTCAATCTTGGAATTTTTGTCAAAACATATGGCTGATGTTGCCTTC
CCACACAACCCCGAGTGCACCGTTTTTCGAGCCCCAGACGGTGATGTCATCTTTTACACATTTGGGTCTT
ACGCTTGCTTTGTGTCCCCAGCCCGCGTCCCATTCGTTGGGGAGCCCCCAAAGAACGTGCATTCAAACAT
AACACGCAATATGACATGGGCCGAGACACTTCGTCTGCTGGCAGAGACCATAACTGAAAGCCTGGTACAC
TTT

>enterovirus A SV19
TGCCAACATCAGGATCAGAATCTATATGAGAATGAAGCATGTTAGAGCTTGGATACCAAGACCATTCAGG
AGCCAACCTTACTTGCTAAAGAACTATCCCAACTWYAATGGYGATGACCTGAAGTGCACTAGTGTTAGTA
GAGCTAAGATAACCACCACAGGTGCTTTTGGCCAACAATCTGGTGCTGTCTACGTTTGTAACTACAGAAT
TGTCAATAGACACTTGGCTACTGAAGAGGATTGGGAGAACCTTGTGTGGGAGGACTACCAGAGAGACCTC
CTAGTTTCCAATACCAAAGCACATGGTTGTGATACTATTGCTAGGTGCAAGTGCCAGACAGGGGTCTACT
TTTGCAAGTCTCAAAATAAACATTATCCTGTTAGCTTTCAGGGCCCTGGCCTAGTTCACGTACAGGCTAA
TGAATACTACCCTGAGCGTTACCAGTCCCACGTTCTTTTGGCTAATGGTATTTCAAAGGCTGGTGATTGT
GGTGGAATTCTCAGATGTCAGCATGGTGTGATTGGGCTTGTGACCATGGGCGGTGATGGTCTAGTTGGCT
TTGCAGACCTCAGAGACTTGCTCTGGCTCGAAGATGATGTGATGGAACAAGGTGTTACAGACTACATTAA
GGGTCTCGGAGACGCATTTGGAACAGGTTTCACTGAATCCATCTCTAGAGAAGTTCAGGCACTTAAAGAT
CACCTGATGGGCGCAGAAGGAATAGTGGAAAAGATTCTTAAGAACTTAGTTAAGATTGTGTCGGCGCTGG
TCATAGTGATCAGGAGTGATTACGACCTAGTCACGATGACTGCAACACTTGCTTTGATCGGTTGTCACAC
AAGCCCATGGGCCTGGTTGAAATCTAAGGTATGTAGCTTCCTTGGCCTCCCGATAGCACAGAAGCAAGGA
GACAATTGGCTGAAGAAATTCAATGACATGGCAAATGCGGCTAAGGGTCTAGAATGGGTAGCAATCAAAA
TCTCCAAGTTCATAGATTGGCTTAAGCAGAAAGTTGTCCCAGCCGCCAGGGAAAAAGTAGAATTCCTCAA
CAATCTCAAACAACTGCCCTTGTTGGAGAACCAGGTTAGCAACCTAGAACAAGCAGCAGCTAGCCAGGAT
GATCTAGAAGCTCTCTTTGGGAATGTGCAGTACCTTGCTATCCATTGCAGGAAATACCAACCTTTGTATG
CCTCTGAAGCACGCAGAATTGCGACATTGGAACGCAGGGTCAACAACTACATGCAGTTCAAGAACAAACA
ACGGATTGAGCCTGTATGTTTGATCATTCGTGGTGCTCCAGGTACAGGAAAATCTCTTGCTACAGGCATC
ATTGCTAGGGCTATTGCAGAGAAATACCACTCCAGTGTATACTCCCTGCCACCTGACCCCGACCACTTCG
ACGGTTACCGTCAACAAGTTGTTACTGTGATGGATGACCTGTGCCAGAACCCAGATGGAAAAGACATCTC
CCTGTTTTGTCAAATGGTTTCTACTGTTGAGTTTATACCACCTATGGCATCATTAGAAGAGAAGGGTCTG
CCTTTTACATCAAAGTTTGTTATTGCTTCCACAAATTCTAGCAACATCATAGTTCCCACTGTTKCAGACA
GCGATGCAATCAGGCGCCGCTTTTACATGGACTGTGACATTGAGGTCCCTGAATCTTACAGACTTGAAAA
TGGGAAGTTGGATGCTTCGAGAGCAGCTAGACTGTGCTCTGAGAACAACACAGCTAACTTTAAGAAATGC
AGCCCTTTAGTTTGTGGAAAAGCTATTCAGTTAAAAGATAGGAAATCTAAGGTGAGATACAGTCTAGACT
CAGTTGTCTCAGAGCTTATCAGAGAGTACAACAACAGAAACAGTGTTGGTAACACCATTGAAGCCCTCTT
CCAAGGGCCCCCGGTGTTTAAGCCCATCAAAATCTCCATGACAGAACCGGCTCCCCCAGCCATTGCTGAC
CTGTTGTCATCAGTTGACAGTGAAGAAGTTAGACAGTATTGTAAGGATAAGGGTTGGATCATCCCAGATG
TGCCCACAAACATTGAAAGACATGTTAACAGAGCTGTTGCAGTCTTGCAATCTATCACCACTGTAGTTGC
TGTAGTGTCCTTGGTTTATGTCATCTACAAGTTGTTCGCTGGCTTCCAGGGTGCTTACTCCGGTATGCCC
AAAACTGCTTTGAAGAAACCAGTCTTGAGGACTGCTACAGTGCAGGGGCCCTGCCTTGACTTCGCCCTGT
CACTACTCCGTAGGAACATTAGGCAAGTACAGACAGACAAGGGCCATTTCACCATGCTCGGGGTGCGTGA

CCGCATTGCTGTGTTGCCACGCCATGCTGAACCAAGTGGAACCATCTGGATTGAAGGTAAACAGGTAAAG
ATCTTAGACGCTGTTGAATTGGTAGATGAACAACAAGTCAATCTTGAACTCACTCTCGTCACCCTTGACA
TGAATGAGAAGTTTAGAGATATTACCAAATTTATTCCAGAGGAAATTGAACCAGTTTCAGAAGCCACTCT
TGTGATTAACACAGAGCAAATGCCCTCCATGTTTGTCCCTGTAGGGAAAGTTGAAAACTATGGGTTCTTG
AACCTAAGTGGGAGACCAACTCACCGTACTCTCATGTACAACTTCCCTACCAAGGCTGGTCAGTGTGGAG
GAGTGGTCACCAGTGTTGGAAAAATCATTGGGATCCACGTTGGTGGAAACGGACGCCAAGGCTTCTGTGC
TGCTCTGAAGCGCTGCTACTTCACTTCTGAGCAAGGTGAAATTCAGTGGATGAAACCAAACAGCGAGACT
GGAAGGTACAACATCAACGGGCCCACTAACACCAAGTTGGAGCCAAGCATCTTCCATGATGTGTTCGAAG
GGAAGAAGGAACCAGCAGCACTGTCATCTAAGGATAAGAGATTAGAAGTTGATTTCGAACAGGCCCTCTT
CTCCAAGTACATAGGTAACAAAATCCATGAACCAGATGAATACATGATTCAAGCTGCCCATCACTATGCT
AATCAGTTAAAACAATTGAACATTGATACATCCAGAATGTCTATGGCTGATGCTTGTTATGGGACTGATA
ATTTAGAAGCCATAGATCTCAGCACCAGTGCAGGATATCCTTACAATGCTCTAGGTATCAAGAAGAGGCA
AATTTTGAACCCTGAGACTCGTGATGTATCCAAAATGAAGTTCTACATGGATAAGTATGGTCTGGATCTC
CCCTACTCTACCTATGTGAAAGATGAACTGAGGAGCATTGAGAAGATCAAGAAAGGTAAATCCCGTTTGA
TTGAGGCAAGCAGCTTGAATGACTCAGTTTACACCAGAATGTGCTTCGGACATCTTTTTGAAGCTTTCCA
TGCTAACCCAGGTACCATCACCGGATCTGCTGTAGGCTGTAACCCAGACACCTTCTGGAGCAAGATCCCC
ATACTCTTACCAGGATCCCTCTTTGCATTTGACTACACTGCCTATGATGCAAGTTTGAGTCCAGCTTGGT
TTAGAGCTTTGGAGATAGTGCTCAGGGAGCTAGGGTACGACTCTGAAGCCATCAATGTTATTGAAGGTAT
CAACCACTCACACCACATTTACAGGAACAAGACCTACTGTGTGTTGGGCGGCATGCCATCTGGCTGCTCT
GGGACTAGTATATTTAATTCCATGATTAACAACATCATCATTAGAACCTTACTTATTAGAACTTTCAAAG
GGATTGATTTAGATGAATTAAACATGATTTCTTACGGTGATGATGTTTTGGCAAGTTACCCCTTCCCAAT
TGATTGTCAAGAATTGGCTAATACTGGTAAGGAATATGGTTTGGTCATGACTCCCGCTGATAAGGGCAGT
TGTTTCAATGAGGTGACTTGGGAGAATGCAACTTTTCTGAAAAGAGGTTTCAAACCCGACGAGCAGTTCC
CTTTCCTCATTCACCCAACAATGCCAATGTCAGAGATCCATGAATCCATCAGATGGACCAGGGACGCACG
TAACACCCAAGACCACGTTCGCTCTCTGTGCCTTCTTGCCTGGCANNNNNNNNNNNNNNNNNNNNNNNNN
NNNNNNNNNNNNNNNNNNNNNNNNNNNNNNNNNNNNNNNNNNNNNNNNNNNNNNNNNNNNNNNNNNNNNN
NNNNNNNNNNNNNNNNNNNNNNNNNNNNNNNNNNNNNNNNNNNNNNNNNNNNNNNNNNNNNNNNNNNNNN
NNNNNNNNNNNNNNNNNNNNNGCAATCCAGGTCGGTTTCTATCAAGCACTTCTGTTTCCCCGGACTTAGT
ACCAATAGGCTGTACCCACGGCTGAAAGGGAAAACGTTCGTTACCCGGCCACTTACTTCGAGAAGCCTAG
TACCATCATTGAATGTCTCAAGTGTTACGTTCAGCACAACCCCAGTGTAGTTCAGGTCGATGAGTCACCG
AATCCCCCACGGGCGACCGTGTCGGTGGCTGCGTTGGCGGCCTGCCTGCGGGTCTTACTCGCAGGACGCT
TGATTCATGACATGGTGCGAAGAGCCTATTGAGCTAGGTGGTAGTCCTCCGGCCCCTGAATGCGGATAAT
CCTAACCACGGAGCCATGCACCACAATCCAGTGGTGTTGTGGTCGTAACGAGTAATTCTGTGGCGGAACC
GACTACTTTGGGTGTCCGTGTTTCCTGTTTTATTTACCTTGGCTGCTTATGGTGACAATCTAGAGTTGTC
ATCATATAGCGATTGGATTGGCCATCCGGATATATTCTTTGTGTGATTTATTTCTTGCTGCCAAGCAACT
GTCATTCGCCATCAATACAATGGGTGCCCAGGTTTCCACTCAGAAAACCGGTTCTCACGAGAATGCCAAT
CTGGCAACTGGTGGTTCTACCATCAATTACACCACCATAAATTACTACAAAGAGTCTTATGCTGCATCAG
CAACAAAACAAGACTTCAGTCAAGACCCATCTAAATTTACTCAACCTGTAGTTGATGTGATAAAAGAATC
ATCCGTCCCTCTTAAATCACCCTCAGCGGAGGCCTGCGGATACAGTGACAGAGTGGCCCAACTTACTCTC
GGAAATTCCACCATTACAACACAAGAAGCAGCTAACATAACGGTGGGTTATGCTGAATGGCCTGAGTTCT
GCTCGGATACCGACGCAACAGCGGTGGACAAACCAACACGCCCTGATGTCTCAGTTAACAGATTTTACAC
GCTCAGCGCCAAGCTGTGGAGCAAAGAATCAAAAGGTTGGTATTGGAAGTTCCCTGACGTCTTAACAGAG
ACAGGTGTGTTCGGGCAGAACGCACAGTTCCATTACTTGTACAGGTCAGGTTTCTGCATTCATGTACAGT
GCAATGCTAGTAAATTTCACCAAGGGACCTTGCTTGTGGCTGCCATCCCAGAATTCACCGTCGCTAGATC
AGATACCTCGACCAACCCTAGCTCTGCGAGACATCCAACATACGAGGCTGCACAACCAGGGAAAAGCGGA
AAGGAACTTGCATTTCCCTATGTTCTGGACTCAGGTGTACCACTGTCACAAGCTCTCATTTACCCACACC
AATGGATCAATTTGAGGACTAACAATTGTGCCACAATAGTGGTGCCATACATAAATGCTGTCCCCTACGA
TAGCGCATTAAATCACTGCAACTTCGGACTGGTTGTTGTTCCGGTGGCACCTTTAGACTACAACACAGGT
GCTACCACCGCCATACCTATAACCATTACCATTGCCCCAATGTGCGCTGAATTTGCTGGATTGCGCAACG
CTGTCACGCAGGGGATCCCAGTGGAATTAAAGCCAGGGTCTAATCAGTTCCTCACCACCGATGATGAAGT

ATCAGCACCCATTTTACCTGGATTTCACCCCACTCCCGAGATACACATCCCAGGCGAAGTTAAAAACATG
CTGGAGATAGCTCAGGTGGAGTCGATCCTGGAGATTAACAACTTGACTAGCAACGAGGGCAACCCCATGA
ACCGACTAGTCATACCTGTGAGTGTCCAGTCTGATGTAGACCAACTCTGTGCTAGCTTCCGTGTCGATCC
TGGCCGCGCTGGTCCATGGGAGTCTACTCTTGTGGGACAATTGTGCCGCTACTACACCCAATGGTCAGGG
TCTCTTGAAGTTACCTTCATGTTTACTGGTTCTTTCATGGCGACAGGTAAAATGCTAATAGCCTACACAC
CACCAGGGGGTGCTCAGCCACTAACAAGGGAGGTCGCAATGCTTGGGACCCATGTTATTTGGGATTTTGG
GCTGCAATCGTCTGTCACTCTGATCATACCATGGATTAGCAACACACACTACCGCACTCAAGCAACATCC
AACTACTTTGAGTACTACTCCAGCGGAATAGTGACACTCTGGTATCAGACCAACTATGTCGTACCAGACG
GGGCTCCTACAAGTGCTTACATCATTGCGCTAGGAGCAGCCCAACCCAACTTTTCGCTGAAGCTCTGTAA
AGACACTGAATCCATCGCACAGACAGCTATTTTGCAGGGGGATCCTGAGGCACCATTAGAAACATTTGTG
GGGAACCAGATTAGCAAGGCTCTCACGGCAGCTGACACAAAACCTAGTACTCACAAGATAGCGACTGGTG
ACACACCAGCCTTGCAAGCAGCAGAAACTGGTGCCACATCAAATGCCAGTGATGAAAACATGATTGAGAC
CAGATGTGTCGTTAACAAACACTCTGTTGCCGAGACATCCGTGGAACACTTCTATGGGCGTGCAGCTCTA
ATAGGCTATGTGTCTATACTCACATCAGAGACCACAGCCAATGGTTTCACCAATTGGGACATCGACATCA
TGGGCTTTGTGCAACAAAGAAGGAAGCTAGAAATTTTCACATACATGCGCTTTGATGCTGAATTTACCTT
CATTGCCACCTCTGACGATGGCCAGGCAGATTCCACAATGTTGCAATATATGTATATACCTCCTGGTGCC
CCCAAACCAACACAAAGAGACTCATTTGAGTGGCAATCAGCAACAAACCCATCCATCTTCACAAGGATGA
CAGACCCGCCAGCACAAGTCTCCGTGCCTTTTATGTCACCTGCTTCAGCTTACCAATGGTTTTACGATGG
TTACCCAACCTTCGGGCAGCANNNNNNNNNNNNNNNNNNNNNNNNNNNNNNNNNNNNNNNNNNNNNNNNN
NNNNNNNNNNNNNNNNNNNNNNNNNNNNNNNNNNNNNNNNNNNNNNNNNNNNNNNNNNNNNNNNNNNNNN
NNNNNNNNNNNNNNNNNNNNNNNNNNNNNNNNNNNNNNNATATGGGTGSACCTTGTGTGGGAGGACTACC
AGAGAGACCTCCTAGTTTCCAATACCAAAGCACATGGTTGTGATACTATTGCTAGGTGCAAGTGCCAGAC
AGGGGTCTACTTTTGCAAGTCTCAAAATAAACATTATCCTGTTAGCTTTCAGGGCCCTGGCCTAGTTCAC
GTACAGGCTAATGAATACTACCCTGAGCGTTACCAGTCCCACGTTCTTTTGGCTAATGGTATTTCAAAGG
CTGGTGATTGBGGTGGAATTCTCAGATGTCAGCATGGTGTGATTGGGCTTGTGACCATGGGCGGTGATGG
TCTAGTTGGCTTTGCAGACCTCAGAGACTTGCTCTGGCTCGAAGATGATGTGATGGAACAAGGTGTTACA
GACTACATTAAGGGTCTCGGAGACGCATTTGGAACAGGTTTCACTGAATCCATCTCTAGAGAAGTTCAGG
CACTTAAAGATCACCTGATGGGCGCAGAAGGAATAGTGGAAAAGATTCTTAAGAACTTAGTTAAGATTGT
GTCGGCGCTGGTCATAGTGATCAGGAGTGATTACGACCTAGTCACGATGACTGCAACACTTGCTTTGATC
GGTTGTCACACAAGCCCATGGGCCTGGTTGAAATCTAAGGTATGTAGCTTCCTTGGCCTCCCGATAGCAC
AGAAGCAAGGAGACAATTGGCTGAAGAAATTCAATGACATGGCAAATGCGGCTAAGGGTCTAGAATGGGT
AGCAATCAAAATCTCCAAGTTCATAGATTGGCTTAAGCAGAAAGTTGTCCCAGCCGCCAGGGAAAAAGTA
GAATTCCTCAACAATCTCAAACAACTGCCCTTGTTGGAGAACCAGGTTAGCAAYCTAGAACAAGCAGCAG
CTAGCCAGGAYGATCTAGAAGCTCTCTTYGGGAATGTGCAGTACCTTGCTATCCATTGCAGGAAATACCA
ACCTTTGTATNNNNNNNNNNNNNNNNNNNNNNNNNNNNNNNNNNNNNNNNNNNNNNNNNNNNNNNNNNNN
NNNNNNNNNNNNNNNNNNNNNNNNNNNNNNNNNNNNNNNNNNNNNNNNNNNNNNNNNNNNNNNNNNNNNN
NNNNNNNNNNNNNNNNNNNNNNNNNNNNNNNNNNNNNNNNNNNNNNNNNNNNNNNNNGTGGWAGYCCTCC
GGCCCCTGAATGCGGMTAATCCTAACCACGGAGCCATGCACCACAATCCAGTGGTGTTGTGGTCGTAACG
AGTAATTCTGTGGCGGAACCGACTACTTTGGGTGTCCGTGTTTCCTGTTTTATTTACCTTGGCTGCTTAT
GGTGACAATCTAGAGTTGTCATCATATAGCGATTGGATTGGCCATCCGGATATATTCTTTGTGTGATTTA
TTTCTTGCTGCCAAGCAACTGTCATTCGCCATCAATACAATGGGTGCCCAGGTTTCCACTCAGAAAACCG
GTTCTCACGAGAATGCCAATCTGGCAACTGGTGGTTCTACCATCAATTACACCACCATAAATTACTACAA
AGAGTCTTATGCTGCATCAGCAACAAAACAAGACTTCAGTCAAGACCCATCTAAATTTACTCAACCTGTA
GTTGATGTGATAAAAGAATCATCCGTCCCTCTTAAATCACCCTCAGCGGAGGCCTGCGGATACAGTGACA
GAGTGGCCCAACTTACTCTCGGAAATTCCACCATTACAACACAAGAAGCAGCTAACATAACGGTGGGTTA
TGCTGAATGGCCTGAGTTCTGCTCGGATACCGACGCAACAGCGGTGGACAAACCAACACGCCCTGATGTC
TCAGTTAACAGATTTTACACGCTCAGCGCCAAGCTGTGGAGCAAAGAATCAAAAGGTTGGTATTGGAAGT
TCCCTGACGTCTTAACAGAGACAGGTGTGTTCGGGCAGAACGCACAGTTCCATTACTTGTACAGGTCAGG
TTTCTGCATTCATGTACAGTGCAATGCTAGTAAATTTCACCAAGGGACCTTGCTTGTGGCTGCCATCCCA
GAATTCACCGTCGCTAGATCAGATACCTCGACCAACCCTAGCTCTGCGAGACATCCAACATACGAGGCTG

CACAACCAGGGAAAAGCGGAAAGGAACTTGCATTTCCCTATGTTCTGGACTCAGGTGTACCACTGTCACA
AGCTCTCATTTACCCACACCAATGGATCAATTTGAGGACTAACAATTGTGCCACAATAGTGGTGCCATAC
ATAAATGCTGTCCCCTACGATAGCGCATTAAATCACTGCAACTTCGGACTGGTTGTTGTTCCGGTGGCAC
CTTTAGACTACAACACAGGTGCTACCACCGCCATACCTATAACCATTACCATTGCCCCAATGTGCGCTGA
ATTTGCTGGATTGCGCAACGCTGTCACGCAGGGGATCCCASTGGARKWWAWRSSRKGSTSWWAYCAGTTC
CTCACCACCGATGATGAAGTATCANNNNNNNNNNNNNNNNNNNNNNNNNNNNNNNNNNNNNNNNNNNNNNN
NNNNNNNNNNNNNNNNNNNNNNNNNNNNNNNNNNNNNNNNNNNNNNNNNNNNNNNNNNNNNNNNNNNNNN
NNNNNNNNNNNNNNNNNNNNNNNNNNNNNNNNNNNNNNNNNNNNNNNNNNNNNNNNNNNNAGACCTGCCC
TCACAGCAAAGGTTCCCATGAGGTTGTTCGGGCATACACCATACCAACTATATGTGCTTGCGGTGTGCTG
CCCGAAGGTTGGGTAACCATCGTAAAACCATTGGTAAGCTGAAGCAGGTGACATAAAAGGCACGGAGACT
TGTGCTGGCGGGTCTGTCATCCTTGTGAAGATGGRTGGGTTTGTTGCTGATTGCCACTCAAATGAGTCTC
TTTGTGTTGGTTTGGGGGCACCAGGAGGTATATACATATATTGCAACATTGTGGAATCTGCCTGGCCATC
GTCAGAGGTGGCAATGAAGGTAAATTCAGCATCAAAGCGCATGTATGTGAAAATTTCTAGCTTCCTTCTT
TGTTGCACAAAGCCCATGATGTCGATGTCCCAATTGGTGAAACCATTGGCTGTGGTCTCTGATGTGAGTA
TAGACACATAGCCTATTAGAGCTGCACGCCCATAGAAGTGTTCCACGGATGTCTCGGCAACAGAGTGTTT
GTTAACGACACATCTGGTCTCAATCATGTTTTCATCACTGGCATTTGATGTGGCACCAGTTTCTGCTGCT
TGCAAGGCTGGTGTGTCACCAGTCGCTATCTTGTGAGTACTAGGTTTTGTGTCAGCTGCCGTGAGAGCCT
TGCTAATCTGGTTCCCCACAAATGTTTCTAATGGTGCCTCAGGATCCCCCTGCAAAATAGCTGTCTGTGC
GATGGATTCAGTGTCTTTACAGAGCTTCAGCGAAAAGTTGGGTTGGGCTGCTCCTAGCGCAATGATGTAA
GCACTTGTAGGAGCCCCGTCTGGTACGACATAGTTGGTCTGATACCAGAGTGTCACTATTCCGCTGGAGT
AGTACTCAAAGTAGTTGGATGTNNNNNNNNNNNNNNNNNNNNNNNNNNNNNNNNNNNNNNNNNNNNNNNN
NNNNNNNNNNNNNNNNNNNNNNNNNNNNNNNNNNNNNNNNNNNNNNNNNNNNNNNNNNNNNNNNNNNNNN
NNNNNNNNNNNNNNNNNNNNNNNNNNNNNNNNNNNNNNNNNNNNNNNNNNNNNNNNNNNNNNNNNNNNNN
NNNNNNNNNGAGGATAGAGGACGGTGAGTTGGTCTCCCACTTAGGTTCAAGAACCCATAGTTTTCAACTT
TCCCTACRKRKRYAWRYMTGKRKGKYWWWWKCWMKGTGTTAATCACAAGAGTGGCTTCTGAAACTGGTTC
AATTTCCTCTGGAATAAATTTGGTAATATCTCTAAACTTCTCATTCATGTCAAGGGTGACGAGAGTGAGT
TCAAGATTGACTTGTTGTTCATCTACCAATTCAACAGCGTCTAAGATCTTTACCTGTTTACCTTCAATCC
AGATGSYTCCACTTGGCTCAGCATGGCGTGGCAACACAGCAATGCGGTCACGCACCCCGAGCATGGTGAA
ATGGCCCTTGTCTGTCTGTACTTGCCTAATGTTCCTACGGAGTAGTGACAGGGCGAAGTCAAGGCAGGGC
CCCTGCACTGTAGCAGTCCTCAAGACTGGTTTCTTCAAAGCAGTTTTGGGCATACCGGAGTAAGCACCCT
GGAAGCCAGCGAACAACTTGTAGATGACATAAACCAAGGACACTACAGCAACTACAGTGGTGATAGATTG
CAAGACTGCAACAGCTCTGTTAACATGTCTTTCAATGTTTGTGGGCACATCTGGGATGATCCAACCCTTA
TCCTTACAATACTGTCTAACTTCTTCACTGTCAACTGATGACAACAGGTCAGCAATGGCTGGGGGAGCCG
GTTCTGTCATGGAGATTTTGATGGGCTTAAACACCGGGGGCCCTTGGAAGAGGGCTTCAATGGTGTTACC
AACACTGTTTCTGTTGTTGTACTCTCTGATAAGCTCTGAGACAACTGAGTCTAGACTGTATCTCACCTTA
GATTTCCTATCTTTTAACTGAATAGCTTTTCCACAAACTAAAGGGCTGCATTTCTTAAAGTTAGCTGTGT
TGTTCTCAGAGCACAGTCTAGCTGCTCTCGAAGCATCCAACTTCCCATTTTCAAGTCTGTAAGATTCAGG
GACCTCAATGTCACAGTCCATGTAAAAGCGGCGCCTGATTGCATCAAATCAATGGGTGGCCGGAATAGAT
CTGCAAGGGTAGTTAATTTCNNNNNNNNNNNNNNNNNNNNNNNNNNNNNNNNNNNNNNNNNNNNNNNNNN
NNNNNNNNNNNNNNNNNNNNNNNNNNNNNNNNNNNNNNNNNNNNNNNNNNNNNNNNNNNNNNNNNNNNNN
NNNNNNNNNNNNNNNNNNNNNNNNNNCATGCSSYTTKATGCTGAGTTCACTTTCATTGCTACYGCTGATG
ACGGCAAAGCAGATTCTACAATGTTACAGTACATGTACATCCCACCAGGTGCACCCAAACCAACACAAAG
GGACTCTTTTGAATGGCAATCAGCAACCAACCCTTCTATCTTTGTTAGAATGACCGACCCACCTGCACAG
GTTTCTGTACCCTTCATGTCGCCTGCTTCCGCTTACCAGTGGTTCTATGACGGTTACCCAACATTTGGAA
CTCACTCTGCCAGCACAGACAGTTGGTATGGAGTCTGTCCTAACAACCTCATGGGGACCTTTGCGGTGAG
AGCAGTTGGTTCAGGTAGAATTACATCAAACATCACAGTAAGAATTTACATGAGGATGAAGCATGTTAAA
GCTTGGATACCAAGGCCATTCAGGAGTCAACCATACCTTTTGAAGAATTACCCCAACATGGATGGTAACA
ACTTGAAGTGTACTAGTACTAGTAGGACTAGAATAACCACCACAGGGGTTTTTGGCCAACAATCCGGTGC
TGTCTATGTTTGCAACTACAGAATTGTCAACAGGCACTTGGCCACCAATGAAGATTGGGAAAACCTTGTG
TGGGAGGATTACCAGAGAGATCTCCTCGTCTCCAACACCAATGCCCACGGTTGCGATAACATTGCTAGAT

GCAAGTGCCAAACAGGGGTCTACTTCTGCAAGTCCCAAAACAAACACTACCCAGTCAGTTTCCAGGGTCC
TGGCTTAGTACAAGTGCAGGCAAATGAATACTACCCCGAGCGTTACCAGTCACATGTTCTGCTGGCTAAT
GGCATCTCAAAGGCTGGCGATTGTGGTGGAATTCTCAGGTGCCAACACGGTGTGATTGGACTTGTGACGA
TGGGCGGTGACGGCCTGGTTGGCTTTGCAGACATCAGAGACCTGCTTTGGCTTGAAGATGATGTGATGGA
ACAAGGTGTTACAGATTACATCAAGGGTCTTGGAGATGCATTTGGGACAGGTTTCACAGAATCTATCTCT
AGGGAAGTGCAGGCGCTCAAAGACCATCTAATGGGAGCAGAGGGGATAGTGGAAAAGATCCTCAAGAACC
TGGTCAAGATTGTGTCGGCGCTGGTCATAGTAATCAGGAGTGACTACGACTTGGTCACGATGACTGCAAC
ACTAGCTTTGATCGGCTGTCACACAAGTCCATGGGCCTGGTTGAAATCTAAAGTGTGTAGCTTCCTTGGC
CTCCCAATAGCACAGAAGCAAGGAGACAATTGGCTAAAGAAATTTAATGACATGGCAAATGCAGCTAAGG
GTCTAGAATGGGTGGCAATCAAAATCTCCAAGTTCATAGATTGGCTCAAGCAGAAAGTTGTCCCAGCCGC
CAGGGAAAAGGTAGAGTTCCTTAACAATCTCAAACAACTGCCCTTGTTGGAGAACCAGGTTAGCAACTTA
GAACAAGCAGCAGCTAGCCAGGACGACCTAGAAGCTCTCTTCGGGAATGTGCAGTACCTTGCCATCCATT
GCAGGAAATACCAACCTTTGTATGCCTCTGAAGCACGCAGAATTGCGACATTGGAGCGCAGAGTCAACAA
CTACATGCAGTTCAAGAACAAACAACGGATTGAACCTGTATGTTTGATCATCCGTGGTGCTCCAGGCACA
GGAAAATCTCTTGCTACAGGCANNNNNNNNNNNNNNNNNNNNNNNNNNNNNNNNNNNNNNNNNNNNNNNN
NNNNNNNNNNNNNNNNNNNNNNNNNNNNNNNNNNNNNNNNNNNNNNNNNNNNNNNNNNNNNNNNNNNNNN
NNNNNNNNNNNNNNNNNGTTGTATTCTGGTATGGACAGTGCCAAGCCAATAGGCACTGATCTGATTTTCT
TGACAAATTGGTCATATGTTTCTTTCCCATTATGCCAGGCAAGAGAGCACAGAGAGCGAACGTGTTCTTG
GGTGTTGCGTGCGTCTCTGGTCCATCTGATGGATTCATGGATCTCTGACATTGGCATTGTTGGGTGGATG
AGGAAAGGGAACTGCTCATCAGGTTTGAAACCTCTTTTCAGAAAAGTTGCGTTCTCCCAAGTCACCTCAT
TGAAGCAACTGCCCTTATCAGCGGGTGTCATGACCAAACCATATTCCTTGCCAGTGTTAGCCAATTCTTG
ACAATCGATTGGAAAGGGGTAACTTGCCAAAACATCATCACCGTAGGAAATCATGTTTAATTCATCTAAA
TCAATTCCTTTAAAGGTTCTGATAAGCAAGGTTCTAATGATGATGTTATTAATCATGGAATTGAATATGC
TAGTTCCAGAGCAGCCAGATGGCATGCCGCCCAACACACAATAGGTCTTGTTTCTGTAAATGTGGTGTGA
GTGGTTGATTCCTTCAATAACATTGATGGCTTCAGAGTCATACCCTAGCTCTCTAAGCACTATTTCCAAA
GCTCTAAACCAAGCGGGACTCAAACTTGCATCATAAGCAGTGTAGTCAAATGCAAAGAGGGATCCTGGTA
AGAGTATAGGGATCTTGCTCCAGAAGGTGTCTGGGTTACAACCTACAGCAGATCCAGTGATGGTACCTGG
GTTAGCATGGAAAGTTTCAAAAAGATGCCCGAAACACATTCTGGTGTAAACTGAGTCATTCAAACTGCTT
GCCTCAATCAAACGGGATTTACCTTTCTTGATCTTCTCAATGCTCCTCAGTTCATCTTTCACATAGGTAG
AGTAGGGGAGATCCAGACCATACTTATCCATATAGAACTTCATTTTGGACACATCGCGAGTCTCGGGGTT
CAAAATCTGTCTCTTCTTGATACCTAGAGCATTGTAAGGGTATCCTGCACTAGTGCTGAGATCTATGGCT
TCTAGGTTATCAGTCCCATAGCAAGCATCAGCCATAGACATTCTGGATGTATCAATGTTCAATTGCTTCA
ACTGATTAGCATAGTGATGGGCAGCTTGAATCATGTATTCATCTGGTTCATGGATTTTGTTACCTATGTA
CTTGGAGAAAAGGGCCTGTTCAAAATCAACTTCTAACCTCTTATCCTTAGATGATAGTGCTGCTGGTTCC
TTTTTCCCTTCAAACACATCATGGAAGATACTTGGCTCTAACTTGGTGTTAGTGGGCCCGTTGATGTTGT
ACCTTCCAGTTTCGCTGTTTGGTTTCATCCACTGAATTTCACCTTGTTCGGAGGTGAAGTAGCAGCGCTT
CAAAGCGGCACAGAAGCCTTGGCGTCCGTTTCCACCAACGTGGATTCCGATGATTTTTCCAACACTGGTG
ACCACTCCTCCACACTGACCAGCCTTGGTAGGGAAGTTGTACATGAGAGTGCGGTGGGTTGGTCTACCAC
TTAGGTTCAAGAACCCATAGTTTTCAACTTTCCCTACAGGGACAAACATGGAGGGCATTTGCTCTGTGTT
AATCACAAGAGTAGCTTCTGAAACTGGTTCAATTTCCTCTGGGATAAATTTGGTGATGTCTCTAAACTTC
TCATTCATGTCAAGGGTGACTAGAGTGAGTTCGAGATTGACTTGTTGTTCGTCTACCAATTCAACAGCGT
CCAAGATCTTCACCTGTTTGCCTTCAATCCAGATGGTTCCGCTCGGTTCAGCATGGCGTGGCAACACAGC
AATGCGATCGCGCACCCCGAGCATGGTGAAATGGCCCTTGTCTGTCTGCACTTGCCTAATGTTCCTACGG
AGTAATGACAGGGCAAAGTCAAGGCAGGGTCCCTGCACTGTAGCAGTCCTCAAGACTGGTTTCTTCAAAG
CAGTTTTGGGCATACCGGAGTAAGCACCCTGGAAGCCAGCGAACAACTTGTAGATGACGTAAACCAAGGA
CACTACAGCTACCACAGTGGTAATGGATTGCAAAACGGCAACAGCTCTGTTGACATGTCTTTCAATGTTT
GTGGGCACATCTGGGATGATCCAACCCTTATCCTTGCAATACTGTCTAACTTCTTCACTGTCAACTGATG
ACAACAGATCAGCAATAGCTGGGGGAGCCGGTTCTGTCATGGAGATTTTAATGGGCTTAAACACTGGGGG
CCCTTGGAAGAGGGCTTCAATGGTGTTACCAACACTGTTTCTGTTGTTGTATTCTCTGATAAGTTCTGAG
ACAACTGAATCTAAACCGTATCTCACCTTAGATTTCCTATCTTTTAACTGAATAGCTTTTCCACAAACTA

GAGGGCTACACTTCTTAAAGTTAGCTGTGTTGTTCTCAGAGCACAGTCTGGCTGCTCTCGAGGCATCCAA
CTTTCCATTCTCAAGTCTGTAAGATTCAGGGACCTCAATGTCACAGTCCATGTAAAAGCGGCGTCTGATT
GCTTCGCTGTCTGAAACAGTGGGGACTATGATATTGCTAGAATTCGTGGAAGCAATGACAAACTTTGATG
TAAAGGGCAGACCCTTCTCTTCTAGTGATGCCATAGGTGGTATGAACTCAACAGTAGAAACCATTTGACA
AAACAAGGAGATGTCTTTCCCGTCTGGGTTCTGGCACAGGTCATCCATCACAGTGACAACTTGTTGACGG
TAACCATCGAAGTGGTCAGGGTCAGGTGGTAGGGAGTATACGCTAGAGTGGTATTTCTCAGCAATAGCCC
TAGCAATGATGCCTGTAGCAAGAGATTTTCCTGTGCCTGGAGCACCACGGATGATCAAACATACAGGTTC
AATCCGTTGTTTGTTCTTGAACTGCATGTAGTTGTTGACTCTGCGCTCCAATGTCGCAATTCTGCGTGCT
TCAGAGGCATACAAAGGTTGGTATTTCCTGCAATGGATGGCAAGGTACTGCACATTCCCGAAGAGAGCTT
CTAGGTCGTCCTGGCTAGCTGCTGCTTGTTCTAAGTTGCTAACCTGGTTCTCCAACAAGGGCAGTTGTTT
GAGATTGTTAAGGAACTCTACCTTTTCCCTGGCGGCTGGGACAACTTTCTGCTTGAGCCAATCTATGAAC
TTGGAGATTTTGATTGCCACCCATTCTAGACCCTTAGCTGCATTTGCCATGTCATTAAATTTCTTTAGCC
AATTGTCTCCTTGCTTCTGTGCTATTGGGAGGCCAAGGAAGCTACACACTTTAGATTTCAACCAGGCCCA
TGGACTTGTGTGACAGCCGATCAAAGCTAGTGTTGCAGTCATCGTGACCAAGTCGTAGTCACTCCTGATT
ACTATGACCAGCGCCGACACAATCTTGACCAGGTTCTTGAGGATCTTTTCCACTATCCCCTCTGCTCCCA
TTAGATGGTCTTTGAGCGCCTGCACTTCCCTAGAGATAGATTCTGTGAAACCTGTCCCAAATGCATCTCC
AAGACCCTTGATGTAATCTGTAACACCTTGTTCCATCACATCATCTTCAAGCCAAAGCAGGTCTCTGATG
TCTGCAAAGCCAACCAGGCCGTCACCGCCCATCGTCACAAGTCCAATCACACCGTGTTGGCACCTGAGAA
TTCCACCACAATCGCCAGCCTTTGAGATGCCATTAGCCAGCAGAACATGTGACTGGTAACGCTCGGGGTA
GTATTCATTTGCCTGCACTTGTACTAAGCCAGGACCCTGGAAACTGACTGGGTAGTGTTTGTTTTGGGAC
TTGCAGAAGTAGACCCCTGTTTGGCACTTGCATCTAGCAATGTTATCGCAACCGTGGGCATTGGTGTTGG
AGACGAGGAGATCTCTCTGGTAATCCTCCCACACAAGGTTTTCCCAATCTTCATTGGTGGCCAAGTGCCT
GTTGACAATTCTGTAGTTGCAAACATAGACAGCACCGGATTGTTGGCCAAAAACCCCTGTGGTGGTTATT
CTAGTCCTACTAGTACTAGTACACTTCAAGTTGTTACCATCCATGTTGGGGTAATTCTTCAAAAGGTATG
GTTGACTCCTGAATGGCCTTGGTATCCAAGCTTTAACATGCTTCATCCTCATGTAAATTCTTACTGTGAT
GTTTGATNNNNNNNNNNNNNNNNNNNNNNNNNNNNNNNNNNNNNNNNNNNNNNNNNNNNNNNNNNNNNNN
NNNNNNNNNNNNNNNNNNNNNNNNNNNNNNNNNNNNNNNNNNNNNNNNNNNNNNNNNNNNNNNNNNNNNN
NNNNNNNNNNNNNNNNNNNNNNNNNNNNNNNNNNNNNNGTCTCACTCGCAGGACGCTTGATTCATGACAT
GGTGCGAAGAGCCTATTGAGCTAGGTAGTAGTCCTCCGGCCCCTGAATGCGGCTAATCCTAACCACGGAG
CCATGCACTACAATCCAGTGGTGTTGTGGTCGTAACGAGTAATTCTGTGGCGGAACCGACTACTTTGGGT
GTCCGTGTTTCCTGTTTTATTTACCTTGGCTGCTTATGGTGACAATCTAGAGTTGTCATCATATAGCGAT
TGGATTGGCCATCCGGATATATTTTTGTGTGATTCACTCTTTGCTCTTCAGCATTTGCAATACTTCATAG
TTTCATCATGGGAGCTCAAGTGTCTACCCAACAATCTGGAACCCACGAAAACACCAATGTGGCCACAGGT
GGATCTACCATTCACTACACCACCATAAACTATTACAAGGATTCCTACGCTGCTTCGGCTAACAAGCAAG
ACTTCAGTCAGGATCCGAGCAAGTTTACCCAACCAGTAGTTGATGTTCTCAAGGAATCATCAGTACCACT
TAAATCTCCTTCAGCAGAGGCGTGTGGTTACAGCGACAGAGTCTGCCAAATGACAGTTGGAAATTCCACC
ATTACCACACAGGAGGCTGCTAATGTGATTGTAGGGTATGGCGAGTGGCCCGAGTACTGTAGTGATTCAG
ATGCCACTGCTGTCGACAAACCAACTAGACCAGATGTTTCGGTTAACAGATTTTACACACTATCAGCAAA
ATTGTGGCAATCAAACAGCAAGGGGTGGTACTGGAAATTCCCCGATGTGTTAACAGAGACAGGTGTTTTC
GGGCAGAATGCACAGTTCCACTACTTATACAGATCAGGCTTTGCTGTCCACGTACAATGCAATGCAAGCA
AGTTTCACCAGGGAACCCTCCTGGTGGCATGTTTACCTGAATACACCAATGGGAGGAGTGATGCAACTAA
TACACCCGGTTCTGCAAAGCACCCATCGTATAATGCCACTCAACCTGGACCCAGTGGTAAAGAATTGGCC
TTTCCCTATGTGTTAGATTCTGGGATAGCTTTCTCTCAATTGCTTGTTGCCCCTCACCAATGGATTAACC
TCAGGACCAATAATTGTGCCACCATAATCATGCCCTATGTGAATGCAGTGCCTTATGATTCGGCCCTAAA
TCACTGCAACTTTGGCTTGTTTGTTATTCCCGTTGCCCCACTAGCGTATAGTGATGGAGCCACAACGGCT
GTGCCAATAACAGTCACCATTGCACCACTGTGTGCTGAGTTTGCCGGTCTGAGGAATGCAGTTACTCAGG
GCTTGCCAGTAGAGTTGAAACCTGGCAGCAACCAATTTCTTACCACAGATGATGGTGTTTCAGCACCCAT
ACTTCCTGGCTTCCACCCAACCCCCTTGATTCACATTCCAGGTGAAGTAAACAACTTGCTACAACTGTGT
CAAATTGAAAGCATACTTGAAGTAAACAATGTGGAGAGTGCATCTCAAATGGAAAGACTTTTGATACCAG
TGTCTGTTCAGAGTGAGGTCGACCAGCTATGTGCCTCCTTTAGAGTGGATCCAGGCCGAGATGGTCCATG

GCAGTCCACTATGGTGGGTCAACTGTGCAGATATTACACTCAATGGTCAGGTTCATTAGAAATTACCTTC
ATGTTTACAGGCAGTTTTATGGCCACTGGAAAACTCTTGATAGCTTACACACCTCCTGGTGGAGTGCAGC
CTACAAGTAGAGCTGTGGCAATGCTTGGCACCCATGTTATCTGGGACTTTGGTCTTCAGTCATCCGTTAC
ATTGGTGATTCCTTGGATTAGCAACACGCATTTTAGGACGAATGCGTCAGGAACCTACTTTGATTATTAC
ACTGCAGGTATTGTCACCATTTGGTACCAAACAAACTTTGTTGTTCCAGCAGGTGCTCCAACCAGTGCTT
ACCTCATAGCTCTGGGCGCTGCTCAACCAAATTTCACCATGCGGCTGTGTAAAGACACTGACGAAATCTC
TCAAACTGCAGTGTTGCAAGGTGATATCAAGGATATGCTAAAAACTCACATCAATGCTACAGCACACAAA
GTTCTTACCAACACTGAGGGCGCCTCTACTAGCACCGGCGCTGACACCAGTGCAAGCTCACACAGCTTAT
TGACAGGGTCTGCACCAGCGCTACAGGCAGCTGAGACGGGTGCAACATCAGTTACCTCTGATGAGTCTAT
GATAGAGACAAGGTGTGTTCACAACCACCACAGTGTTGCTGAAACGAGTCTGGAAAACTTCTTTGGCAGG
GCAGCCCTTGTTGGGATGGCCACTCTACTCACATCAGAGAGCACAACAAATGGTTTTACCAACTGGCCAA
TTGACATAATGGGCTATGTACAACAGCGGAGGAAGCTGGAAATTTTCACATACATGAGGTTTGACTCTGA
GTTCACTTTTGTTTGTACCGACTCAACAGGCCAAGGGCCCAATGTGGTGGTTCAGTACATGTATATTCCC
CCAGGTGCTCCTGCCCCAACTGCAAGGGACTCTTTTGAGTGGCAATCTGCCACAAACCCCTCTGTGTTCG
TCAAGGCATCTGACCCTCCAGCTCAGGTCTCTGTTCCTTTCATGTCTCCAGCTTCAGCTTATCAATGGTT
CTATGATGGGTACCCCACATTCGGTACACACCCTGCATCTGGTGATGTGAATTATGGGATCAGTCCTAAC
AATCTTTTTGGCACCTTTGCTTTTAGAGTGGTGGGGTCTGAGAATGTCACAACCAAATTGACTGTCAGAA
TTTACATGAGAATTAAACACGTTAGGGCATGGGTCCCTAGACCAATGAGAAGCCAGCCGTATGTGTTGAA
GAACTAYMMWARMTYKWMYKRCAATRRTANCASNGCACATGGSTRTAAACAGGGCTARMATAACCACMAC
TGGRGCCTTTGGTCAGCAGAGTGGTGCTGTGTATGTGTGTAATTACAGGATTGTCAACAGACACCTGGCT
ACTGAGAATGATTGGGACAACCTTGAATGGGAGGACTATCAGAGAGACATTCTTGTTTCCAGAACCACTG
GGCATGGGTGTGACAAGATCGCCAGGTGCAACTGCAACACAGGAGTGTATTTCTGCAAATCAGCAAACAA
ACATTACCCAGTTTCCTTCCAGGGCCCTGGTCTTGTGCACGTCCAAGCAAACGAGTACTACCCTGAAAGA
TACCAATCACATGTACTATTAGCAAATGGGATCGCTGTAGCAGGTGATTGTGGTGGCATCCTTAGGTGCC
CCCATGGTGTGATTGGCTTAGTTACCATGGGCGGTGATGGGTTGGTGGGTTTTGCTGACCTGAGAGACCT
CCTTTGGCTAGAGGATGAAGTGATGGAACAGGGTGTCACTGACTACATTAAGGGCTTGGGCGACGCTTTC
GGAACCGGTTTCACCGAGTCAATCTCCAGAGAGGTCCAGAACATCAAAGATACACTCGTTGGATCAGAAG
GTGTAGTAGAGAAAATCCTGAAGAACCTGGTCAAGTTGATATCCGCTCTGGTCATAATTGTGAGGAGTGA
GTACGATCTGGTTACTGTCACAGCAACATTGGCACTGATTGGTTGTACAGGTAGTCCATGGAGATGGCTT
AAAACAAAGGTCGCATCAATTCTAGGCATCCCCATCGCCCAAAAACAATCTGACTCTTGGCTCAAGAAAT
TCAATGACATGGCCAACGCAGCTCGTGGCCTTGAATGGATTGCGAACAAAATTAGCAAGTTCATTGATTG
GATAAGAGAAAAGATCATACCTATTGCAAGAGAAAAGGCAGAATTTATTAACAACCTCAAGCAACTTCCT
CTACTGGAAAATCAGATTAACAACCTGGAACAGGCTGCTGCAAGTCAGGAGAGTTTAGAACAACTGTTTG
GCAATGTTCAATATTTGGCAATCAATTGTAGGAAATTCCAACCACTCTACGCAGCTGAGGCTAAGAGAGT
CTTCTCAATGGAGAAGAGAATGGTCAACTACATGCAGTTCAAGGGCAAACAACGTATTGAACCTGTATGC
CTGATCATTAGAGGCTCTCCTGGGACAGGGAAATCCCTGGCAACCAGCATAATTGCCAGAGCCATCGCTG
AGAAATTCAACTCCAGCGTGTACTCATTACCACCTGACCCTGACCACTTCGATGGTTACAGTCAACAAGT
TGTCACTGTGATGGATGACCTGTGCCAGAACCCAGACGGGAAAGACATCTCCTTGTTTTGTCAAATGGTT
TCCACAGTTGAGTTCATACCACCAATGGCCTCACTAGAAGAGAAAGGTATTGCATTCAACTCAAAATTCG
TCATCGCTTCCACCAATGCTGGTAACATCATTGTGCCCACCGTTTCTGACTCAGAAGCAATTAGGAGGAG
GTTCTACATGGATTGTGACATTGAAGTCCCAGACAGCTTTAGATTGGAAAGTGGTAAATTGGATGCAGGC
AGAGCAGCAAAGTTGTGCTCAGAAAACAACACCGCCAACTTCAAAAGATGCAGCCCACTGGTCTGTGGTA
AGGCTATTCAGTTAAAAGACAGAAAGTCAAAAGTTAGATACAGTTTGGATTCAGTGGTCTCAGAATTGAT
TAGAGAATACAACAATAGAATGTCAGTTGGAAATACAATTGAGGCATTATTCCAAGGCCCACCAAAGTTT
AAACCTCTCACCATTTCTTTGGACACTCCAGCCCCAGACGCTATTGCCGACCTCTTGAGAAGTGTTGACA
ATGAAGAAGTGAGAGAATACTGCAAGGAAAAAGGGTGGATCATCCCCGAGGTACCAACAAATTTAGAGAG
ACAATACAATAAAGCAATTGCACTAATCCAGTCCATCACTACCATTCTGGCTGTTGTATCTATGGTGTAT
GTCATCTACAGGCTGTTCGCTGGATTCCAAGGTGCTTACTCCGGAATGCCCAAGACGGCAGTGAAGAAAC
CAGTCTTAAGAACAGCAGTAGCACAAGGGCCAGGCTTAGACTTTGCCATCTCACTACTTAAAAAGAACAT
CAGAAAAGTGCAAACTGAAGAGGGACACTTTACCATGCTTGGTGTTAGAGACAGGCTTGCAGTGCTCCCA

AGGCATGCAAAACCTGGCAAGACCATCTGGCTAGAAGGAAAACAAGTGACAGTCCTAGATGCGGTTGAAC
TAGTTGATGAGCAGCAAGTAAACTTGGAATTAACCTTAGTCACATTGGACATGAATGAGAAATTTAGAGA
CATTACTAAATTCATCCCTGAATCATTTGAACACTGTATGGATGCTACACTCATCATCAACACAGAGCAG
ATGCCATCAATGTTTGTTCCAGTGGGTGATGTCCAGTTTTATGGCTTCCTGAATCTGAGTGGAAAACCAA
CACACAGAACAATGATGTACAACTTCCCAACCAAAGCCGGCCAGTGTGGTGGAGTGGTTACTAGTACAGG
AAAAGTGATTGGAATTCATGTTGGAGGTAATGGAAAGCAAGGGTTCTGTGCGGCACTGAAGAGGAGTTAC
TTTGCCACCGAGCAAGGTGAGATCCAGTGGATGAAACCGAACAAGGAAACTGGCCGCTACAACATTAATG
GTCCAACCCAGACCAAACTTGAACCCAGTGTGTTTCATGATGTGTTTGAGGGTAAGAAGGAGCCTGCAGC
TCTTAGTGCAAAGGACCCAAGGTTGGAAGTTGACTTTGAGGAAGCCCTGTTCAGCAAATACATTGGCAAT
GCTATTCATGAACCAGATGAGTATATCATTCAAGCAGCTAAACATTACTCAAACCAACTAAAGCAATTGA
ACATTGATACCAGTAGGATGAGTATGGAAGATGCTTGCTATGGAACAGAGAACTTGGAAGCAATTGACCT
TAGCACCAGTGCAGGATACCCCTACAATGCTTTAGGCATCAAGAAAAGGCAGATTTTGGACCCTACCACT
AGAGATGTCACTAAAATGAAGTACTACATGGATAAGTATGGATTAGACCTGCCCTTCTCCACATATATCA
AGGATGAGTTAAGAAGCAGGGAGAAAGTGAAGAAAGGAAAATCCAGACTGATTGAAGCATCAAGTCTCAA
TGATTCAGTATACATGAGGATGTGCTTTGGACACCTGTTTGAAAAATTCCATGAGAATCCAGGCACCATT
ACTGGTTCTGCAGTAGGAGCAAACCCTGATACCTTCTGGAGTAAGATCCCCATCCTTCTTCCAGGCAGTT
TGTTTGCTTTTGACTACACCGGTTATGACGCTAGCTTGAGCCCAGCATGGTTCAGAGCACTAGAAATGGT
CTTGAAAGATCTAGGTTATGACGATGAGGCTGTCAGCCTCATTGAGGGTATTAACCACTCCCACCACATC
TACCGCAACAAAACATACTGCGTAGTTGGTGGCATGCCCTCAGGCTGCTCTGGGACCAGTATATTCAATA
GCATGATCAATAATATTATCATTAGGACCCTGTTAATTAGAACATTCAAGGGCATTGATTTAGATGAATT
AAACATGATTGCTTATGGAGATGATGTGTTAGCTAGCTACCCGTTCCCAATCGACTGCGCTGAGTTAGCA
AGGACTGGTAGGGAATATGGTTTAGTCATGACACCTGCAGACTAANNNNNNNNNNNNNNNNNNNNNNNNN
NNNNNNNNNNNNNNNNNNNNNNNNNNNNNNNNNNNNNNNNNNNNNNNNNNNNNNNNNNNNNNNNNNNNNN
NNNNNNNNNNNNNNNNNNNNNNNNNNNNNNNNNNNNNNNNNNNNNNNNNNNNNNNNNNNNNNNNNNNNNN
NNNNNNNNNNAGTGAGTTCMAAATGTTGGGTAACCGTCATAGACACTGGTAGHGATARGCAGGCGACATG
AAGGGTACAGAAACCTGTGCAGGTGGGTCGGTCATTCTAACAAAGATAGAAGGGTTGGTTGCTGATTGCC
ATTCAAAAGAGTCCCTTTGTGTTGGTTTGGGTGCACCTGGTGGGATGTACATGTACTGTAACATTGTAGA
ATCTGCTTTGCCGTCATCAGCAGTAGCAATGAAAGTGAACTCAGCATCAAAGCGCATGTAGGTGAAGATC
TCAAGTTTCCTTCTTTGCTGAACAAAGCCCATGATATCTATATCCCAATTAGTGAAGCCATTNGCTGTMC
CTCCCGAGGTGAGTATGGATACTAAGCCAACGAGTGCCGCACGCCCATAGAAGTGCTCAATAGAAGTTTC
TGCGACAGAGTGCTTGTTAACCACACATCTTGTCTCAATCATGCCCTCATCACTGGTGTTTGATGTTGCC
CCAGTTTCTGCAGCTTGCAGCGCTGGGGTGTTGCCTGTTGAAATGTTGTGGTTACTGGGCTTGGTGTCAG
CTGCTGTCAAGGTCTTGCTGACTTGGCTCTCCACAAATATCCTYCAGGATTNNNNNNNNNNNNNNNNNNN
NNNNNNNNNNNNNNNNNNNNNNNNNNNNNNNNNNNNNNNNNNNNNNNNNNNNNNNNNNNNNNNNNNNNNN
NNNNNNNNNNNNNNNNNNNNNNNNNNNNNNNNNNNNNNNNNNNNNNNNNNNNNNNNNNNNNNNNNNNNTTC
TCMATGCTCCTCAGTTCATCCTTCACATAGGTAGAGTAGGGGAGGTCTAATCCATACTTATCCATGTAGT
ACTTCATTTTAGTGACATCTCTAGTGGTAGGGTCCAAAATCTGCCTTTTCTTGATGCCTAAAGCATTGTA
GGGGTATCCTGCACTGGTGCTAAGGTCAATTGCTTCCAAGTTCTCTGTTCCATAGCAAGCATCTTCCATA
CTCATTCTGGATGTATCAATGTTCAATTGCTTCAACTGATTAGCATAGTGATGGGCAGCTTGAACCATGT
ATTCATCTGGTTCATGGATTTTGTTACCTATGTACTTGGAGAAAAGGGCCTGTTCAAAATCAACTTCTAA
CCTCTTATCCTTAGATGATAGTGCTGCTGGTTCCTTTTTCCCTTCAAACACATCATGGAAGATACTTGGC
TCCAACTTGGTGTTAGTGGCCCGTTGATGTTGTACCTTCCAGTTTCGCTGTTTGGTTTCATCCACTGAA
TTTCACCTTGTTCGGAGGTGAAGTAGCAGCGCTTCAAAGCGGCACAGAAGCCTTGGCGTCCGTTTCCACC
AACGTGGATTCCGATGATTTTTCCAACACTGGTGACCACTCCTCCACACTGACCAGCCTTGGTAGGGAAG
TTGTACATGAGAGTGCGGTGGGTTGGTCTACCACTTAGGTTCAAGAACCCATAGTTTTCAACTTTCCCTA
CAGGGACAAACATGGAGGGCATTTGCTCTGTGTTAATCACAAGAGTAGCTTCTGAAACTGGTTCAATTTC
CTCTGGGATAAATTTGGTGATGTCTCTAAACTTCTCATTCATGTCAAGGGTGACTAGAGTGAGTTCGAGA
TTGACTTGTTGTTCGTCTACCAATTCAACAGCGTCCAAGATCTTCACCTGTTTGCCTTCAATCCAGATGG
TTCCGCTCGGTTCAGCATGGCGTGGCAACACAGCAATGCGATCGCGCACCCCGAGCATGGTGAAATGGCC
CTTGTCTGTCTGCACTTGCCTAATGTTCCTACGGAGTAATGACAGGGCAAAGTCAAGGCAGGGTCCCTGC

ACTGTAGCAGTCCTCAAGACTGGTTTCTTCAAAGCAGTTTTGGGCATACCGGAGTAAGCACCCTGGAAGC
CAGCGAACAACTTGTAGATGACGTAAACCAAGGACACTACAGCTACCACAGTGGTAATGGATTGCAAAAC
GGCAACAGCTCTGTTGACATGTCTTTCAATGTTTGTGGGCACATCTGGGATGATCCAACCCTTATCCTTG
CAATACTGTCTAACTTCTTCACTGTCAACTGATGACAACAGATCAGCAATAGCTGGGGGAGCCGGTTCTG
TCATGGAGATTTTAATGGGCTTAAACACTGGGGGCCCTTGGAASAGKGCYTCAATGGTGTTACCAACACT
GTTTCTGTTGTTGTATTCTCTGATAAGTTCTGAGACAACTGAATCCAAACTGTATCTCACCTTAGATTTT
CTGTCTTTTAACTGAATAGCTTTACCACAAACTAGAGGGCTACACTTCTTAAAGTTAGCTGTGTTGTTCT
CAGAGCACAGTCTGGCTGCTCTCGAGGCATCCAACTTTCCATTCTCAAGTCTGTAAGATTCAGGGACCTC
AATGTCACAGTCCATGTAAAAGCGGCGTCTGATTGCTTCGCTGTCTGAAACAGTGGGGACTATGATATTG
CTAGAATTCGTGGAAGCAATGACAAACTTTGATGTAAAGGGCAGACCCTTCTCTTCTAGTGATGCCATAG
GTGGTATAAACTCAACAGTAGAAACCATTTGACAAAACAAGGAGATGTCTTTCCCGTCTGGGTTCTGGCA
CAGGTCATCCATCACAGTGACAACTTGTTGACGGTAACCATCGAAGTGGTCAGGGTCAGGTGGTAGGGAG
TATACGCTAGAGTGGTATTTCTCAGCAATAGCCCTAGCAATGATGCCTGTAGCAAGAGATTTTCCTGTGC
CTGGAGCACCACGGATGATCAAACATACAGGTTCAATCCGTTGTTTGTTCTTGAACTGCATGTAGTTGTT
GACTCTGCGCTCCAATGTCGCAATTCTGCGTGCTTCAGAGGCATACAAAGGTTGGTATTTCCTGCAATGG
ATGGCAAGGTACTGCACATTCCCGAAGAGAGCTTCTAGGTCGTCCTGGCTAGCTGCTGCTTGTTCTAAGT
TGCTAACCTGGTTCTCCAACAAGGGCAGTTGTTTGAGATTGTTAAGGAACTCTACCTTTTCCCTGGCGGC
TGGGACAACTTTCTGCTTGAGCCAATCTATGAACTTGGAGATTTTGATTGCCACCCATTCTAGACCCTTA
GCTGCATTTGCCATGTCATTAAATTTCTTTAGCCAATTGTCTCCTTGCTTCTGTGCTATTGGGAGGCCAA
GGAAGCTACACACTTTAGATTTCAACCAGGCCCATGGACTTGTGTGACAGCCGATCAAAGCTAGTGTTGC
AGTCATCGTGACCAAGTCGTAGTCACTCCTGATTACTATGACCAGCGCCGACACAATCTTGACCAGGTTC
TTGAGGATCTTTTCCACTATCCCCTCTGCTCCCATTAGATGGTCTTTGAGCGCCTGCACTTCCCTAGAGA
TAGATTCTGTGAAACCTGTCCCAAATGCATCTCCAAGACCCTTGATGTAATCTGTAACACCTTGTTCCAT
CACATCATCTTCAAGCCAAAGCAGGTCTCTGATGTCTGCAAAGCCAACCAGGCCGTCACCGCCCATCGTC
ACAAGTCCAATCACACCGTGTTGGCACCTGAGAATTCCACCACAATCGCCAGCCTTTGAGATGCCATTAG
CCAGCAGAACATGTGACTGGTAACGCTCGGGGTAGTATTCATTTGCCTGCACTTGTACTAAGCCAGGACC
CTGGAAACTGACTGGGTAGTGTTTGTTTTGGGACTTGCAGAAGTAGACCCCTGTTTGGCACTTGCATCTA
GCAATGTTATCGCAACCGTGGGCATTGGTGTTGGAGACGAGGAGATCTCTCTGGTAATCCTCCCACACAA
GGTTTTCCCAATCTTCATTGGTGGCCAAGTGCCTGTTGACAATTCTGTAGTTGCAAACATAGACAGCACC
GGATTGTTGGCCAAAAACCCCTGTGGTGGTTATTCTAGTCCTACTAGTACTAGTACACTTCAAGTTGTTA
CCATCCATGTTGGGGTAATTCTTCAAAAGGTATGGTTGACTCCTGAATGGCCTTGGTATCCAAGCTTTAA
CATGCTTCATCCTCATGTAAATTCTTACTGTGATGTTTGATGTAATTCTACCTGAACCAACTGCTCTCAC
CGCAAAGGTCCCCATGAGGTTGTTAGGACAGACTCCATACCAACTGTCTGTGCTGGCAGAGTGAGTTCCA
AATGTTGGGTAACCGTCATAGAACCACTGGTAAGCGGAAGCAGGCGACATGAAGGGTACAGAAACCTGTG
CAGGTGGGTCGGTCATTCTAACAAAGATAGAAGGGTTGGTTGCTGATTGCCATTCAAAAGAGTCCCTTTG
TGTTGGTTTGGGTGCACCTGGTGGGATGTACATGTACTGTAACATTGTAGAATCTGCTTTGCCGTCATCA
GCAGTAGCAATGAAAGTGAACTCAGCATCAAAGCGCATGTAGGTGAAGATCTCAAGTTTCCTTCTTTGCT
GAACAAAGCCCATGATATCTATATCCCAATTAGTGAAGCCATTTGCTGTACCTTCCGAGGTGAGTATGGA
TACTAAGCCAACGAGTGCCGCACGCCCATAGAAGTGCTCAATAGAAGTTTCTGCGACAGAGTGCTTGTTA
ACCACACATCTTGTCTCAATCATGCCCTCATCACTGGTGTTTGATGTTGCCCCAGTTTCTGCAGCTTGCA
GCGCTGGGGTGTTGCCTGTTGAAATGTTGTGGTTACTGGGCTTGGTGTCAGCTGCTGTCAAGGTCTTGCT
GACTTGGCTCTCCACAAATGTCTCTAGTGAGGATTCTGGGTCACCCTGCAGGATGGCTGTCTGTGAGATG
GATTCAGTATCCTTGCAAAGTTTCAGTGTGAAGTTAGGTTGGGCAGCTCCCAGCGCAATGATGTAAGCAC
TGGTGGGGGCTCCCTCAGGCACAACATAATTGGTTTGATACCATATTGTCACTATTCCACTGGTGTAGTA
CTCAAAGTATTTATCAGTTGCCTGTGTTCTGTAGTGAGTGTTACTAATCCATGGGATAATCATGGTGACA
GACGATTGCAAGCCAAAATCCCAGATGACATGTGTTCCGAGCATAGCGATCTCTCTTGATGCTGGTTGTG
CACCACCTGGTGGTGTGTAAGCTATCAGCATCTTACCTGTTGCCATGAAAGAACCAGTGAACATGAAAGT
CACTTCTAGTGACCCCGACCATTGAGTATAGTAGCGGCACAGCTGGCCAACAAGTGTAGATTCCCAAGGC
CCTGCACGGCCAGGATCCACACGAAAGCTAGCGCACAGTTGGTCCACTGCTGATTGCACACTTACAGGTA
TGAGAAGGCGTTGCATGGGGTTGTTTTCATTTGTGGTAAGGTTGTTTACCTCGAGTATCGATTCCACTTG

AGCAATTTCCAATAGGTTTTTGACCTCTCCTGGGATATGTATCTCTGGTGTTGGGTGATATCCGGGTAGG
ATGGGTGCGGACACCTCATCATCGGTGGTTAAGAATTGGTTAGAACCTGGTTTCAGCTCAACTGGAAGAC
CTTGGGCGACGGCATTGCGGAGACCTGCAAATTCCGAATTCATTGGGGCAATGGTAACGGTGATTGGTAT
AGCTGTGGTTGCACCTGTATTAAAGTCTAGCGGTGCCACAGGGATGATAACTAAGCCAAAGTTACAGTGG
TTCAAGGCACTATCATAGGGTACGGCATTGACGTATGGCATCACTATTGTAGCACAGTTATTTGTCCTCA
GGTTAATCCACTGGTGTGGGTAGATAAGAGCCTGGGATAGTGGGACACCTGAATCAAGGACATAAGGTAT
AGCAAGTTCCTTACCGTTTTTGCCTGGCTGAGCTGCTGCATAAGATGGGTGCCTTGCAGTGGCAGGGTCA
GTTGCGCTGTCAGAGCGGGCCACAGTGAATTCTGGGATAACAGCGACTAACAAGGCCCCCTGGTGGAACT
TGCTYGCATTACATTGTACATGTATGCAGAAACCTGATCTGTATAAGTAGTGAAACTGTGCATTCTGGCC
AAAAACACCTGTCTCAGTTAAGACATCAGGAAATTTCCAATACCACCCCTTGCTGTTTGATTGCCACAAT
TTTGCTGATAGTGTGTAAAATCTGTTAACCGAAACATCTGGTCTAGATGGCTTGTCCACAGCTGTTGCAT
CTGTGTTTGAGCAAAATTCAGGCCATTCACCATAGCCTACTGTTATGTTGGCAGCTTCCTGGGTTGTGAT
GGTGGAATTACCTAGAGTAAGTTGGGCCACTCTATCGCTGTAACCACACGCTTCAGCTGAGGGGGATTTA
AGAGGCACAGAAGACTCCTTTATCATATCCACTACAGGTTGAGTAAACTTAGATGGGTCCTGACTGAAGT
CTTGTTTAGTTGCCGAAGCAGCATAAGATTCTTTGTAATAATTTATGGTGGTGTAATTGATGGTGGAACC
ACCAGTCGCCATGTTGGCATTTTCGTGAGAGCCAGTTTTCTGAGTGGAAACTTGGGCACCCATAATGTTG
TTGGTAAATGATAACTGCTTGGTATCAAGAGGTGAATCACACAAAAGATGTATCCGGATGGCCAATCCAA
TCGCTATATGATGACAACTCTAGATTGTCACCATAAGCAGCCAAGGTAAATAAAACAGGAAACACGGACA
CCCAAAGTAGTCGGTTCCGCCACAGAATTACTCGTTACGACCACAACACCACTGGATTGTAGTGCATGGC
TCCGTGGTTAGGATTAGCCGCATTCAGGGGCCGGAGGACTACTACCTAGCTCAATAGGCTCTTCGCACCA
TGTCATGAATCAAGCGTCCTGCSAGTGAGANNNNNNNNNNNNNNNNNNNNNNNNNNNNNNNNNNNNNNNN
NNNNNNNNNNNNNNNNNNNNNNNNNNNNNNNNNNNNNNNNNNNNNNNNNNNNNNNNNNNNNNNNNNNNNN
NNNNNNNNNNNNNNNNNNNNNNNNNNNNNNNNNNNNNNNNNNNNNNNNNNNNNNNNGATCTTGGGTGTTA
CGCGCATCCCTGGTCCATCTGATGGATTCATGGATCTCTGACATTGGCATTGTTGGGTGGATGAGGAAGG
GGAACTGCTCATCAGGTTTGAAGCCTCTTTTCAGAAAAGTTGCATTCTCCCAGGTCACCTCATTGAAACA
ACTGCCCTTATCAGCGGGGGTCATGACCAAACCATATTCCTTACCAGTGTTAGCCAATTCTTGACAGTCA
ATTGGGAAGGGGTAACTTGCCAAAACATCATCACCGTAAGCAACCATGTTCAACTCATCTAAATCAATTC
CTTTGAAAGTTCTAATAAGTAAGGTTCTAATGATAATGTTGTTAATCATGGAGTTGAACACGCTAGTTCC
AGAGCAGCCAGATGGCATGCCGCCCAACACACAGTAGGTCTTGTTCCTGTAAATGTGGTGTGAGTGGTTG
ATACCTTCAATAACATTGATGGCTTCAGAGTCATACCCTAGCTCCCTAAGCACTATTTCCAAAGCTCTAA
ACCAAGCTGGACTCAAACTTGCATCATAGGCAGTGTAGTCAAATGCAAAGAGGGATCCTGGTAAGAGTAT
AGGGATCTTGCTCCAAAAGGTGTCTGGGTTACAACCTACAGCAGATCCAGTGATGGTACCTGGGTTAGCA
TGGAAAGTTTCAAAAAGATGCCCGAAGCACATTCTGGTGTAAACTGAGTCATTCAAACTACTTGCCTCAA
TCAAACGGGATTTACCTTTCTTGATCTTTTCGATGTTCCTCAGTTCATCTTTCACATAGGTAGAGTAGGG
GAGATCCAGACCATACTTGTCCATATAAAACTTCATTTTGGATACATCGCGAGTCTCGGGGTTCAGAATC
TGCCTCTTCTTGATACCTAGAGCATTGTAAGGATATCCTGCACTGGTGCTGAGATCTATAGCTTCTAGGT
TTTCAGTCCCATAGCAAGCATCAGCCATAGACATTCTGGATGTATCAATATTCAATTGCTTCAATTGATT
AGCATAGTGATGAGCAGCTTGAATCATGTACTCATCTGGCTCATGGATTTTGTTGCCTATGTACTTGGAG
AAAAGGGCCTGTTCAAAATCAACCTCTAATCTCTTATCCTTAGATGACAGAGCTGCTGGCTCCTTCTTCC
CTTCGAACACATCATGGAAGATGCTTGGCTCCAACTTGGTGTTAGTGGGCCCGTTGATGTTGTACCTTCC
AGTCTCGCTGTTTGGTTTCATCCACTGAATTTCACCTTGCTCAGAAGTGAAGTAGCAGCGCTTCAGAGCA
GCACAGAAGCCTTGGCGTCCGTTTCCACCAACATGGATTCCAATGATTTTTCCAACACTGGTGACCACTC
CTCCACACTGACCAGCCTTAGTAGGGAAGTTGTACATGAGAGTACGGTGGGTTGGTCTCCCACTTAGGTT
CAAGAACCCATAGTTTTCGACTTTCCCCACAGGGACAAACATGGAGGGCATTTGCTCTGTGTTAATCACG
AGAGTAGCTTCTGAAACTGGTTCAATTTCCTCTGGAATGAATTTGGTGATGTCTCTAAACTTCTCATTCA
TGTCAAGGGTGACGAGAGTGAGTTCGAGATTAACCTGTTGCTCATCCACCAGTTCAACAGCGTCCAAGAT
CTTCACCTGTTTGCCTTCAATCCAGATGGTTCCACTTGGTTCAGCATGGCGTGGTAACACAGCAATGCGA
TCACGTACCCCGAGCATGGTGAAATGGCCCTTGTCTGTCTGCACTTGCCTAATGTTCCTACGCAGTAATG
ACAGGGCAAAGTCAAGACAGGGTCCCTGTACTGTAGCAGTCCTCAAGACTGGTTTCTTCAAAGCAGTTTT
GGGCATACCGGAGTAAGCACCTTGGAAGCCAGCGAACAACTTGTAGATGACGTAAACCAAGGACACTACA

GCAACTACAGTGGTGATGGATTGCAAAACTGCAACAGCTCTGTTAACATGTCTTTCAATGTTTGTGGGCA
CATCTGGGATGATCCAACCCTTATCCTTACAATACTGTCTAACTTCTTCACTGTCAACTGATGACAACAG
ATCAGCAATAGCTGGGGGAGCTGGTTCTGTCATGGAGATCTTGATGGGTTTAAACACTGGGGGCCCTTGG
AAGAGGGCTTCAATGGTGTTACCAACACTGTTTCTGTTGTTGTACTCTCTGATAAGTTCTGAGACAACTG
AATCTAAACTGTACCTCACCTTAGATTTCCTATCTTTTAACTGAATAGCTTTTCCACAAACTAGAGGGCT
GCACTTCTTGAAGTTAGCTGTGTTGTTCTCGGAGCACAGTCTGGCTGCTCTCGAAGCATCCAGCTTCCCA
TTCTCAAGTCTGTAAGATTCAGGGACCTCAATGTCACAATCCATGTGAAAACGACGCCTGATTGCTTCGC
TGTCTGAAACAGTGGGGACTATGATATTGCTAGAATTTGTGGAAGCAATGACAAACTTTGATGTAAAGGG
CAGACCCTTCTCTTCTAATGATGCCATAGGTGGTATAAACTCAACAGTGGAAACCATTTGACAAAACAAG
GAGATGTCCTTCCCGTCTGGGTTCTGGCATAAGTCATCCATTACAGTAACAACTTGTTGACGGTAACCGT
CGAAATGGTCAGGGTCAGGCGGCAGGGAATACACGCTGGAGTGGTATTTCTCAGCAATAGCCCTAGCAAT
GATGCCTGTAGCAAGAGATTTTCCTGTACCTGGAGCGCCACGAATGATCAAACATACAGGTTCAATCCGT
TGTTTGTTCTTGAACTGCATGTAGTTGTTGACTCTGCGTTCCAATGTTGCAATTCTGCGTGCTTCAGAGG
CATACAAAGGTTGGTATTTCCTGCAATGGATAGCAAGGTACTGCACATTCCCGAAGAGAGCTTCCAGATC
GTCCTGGCTAGCTGCTGCTTGTTCTAGATTGCTAACCTGGTTCTCCAACAAGGGCAGTTGTTTGAGATTG
TTGAGGAATTCCACTTTTTCCCTGGCGGCTGGGACAACTTTCTGTTTGAGCCAATCTATAAACTTGGAGA
TTTTGATTGCCACCCATTCCAGACCCTTAGCCGCATTTGCCATGTCATTGAATTTCTTTAGCCAATTGTC
TCCTTGCTTCTGTGCTATTGGGAGGCCAAGGAAACTACATACCTTAGATTTCAACCAGGCCCATGGGCTT
GTGTGACAGCCGATCAAAGCAAGTGTTGCAGTCATCGTGACTAGGTCGTAGTCACTCCTGATTACTATGA
CCAGCGCCGACACAATCTTGACCAGGTTCTTGAGGATCTTTTCCACTATCCCCTCTGCTCCCATTAGATG
GTCTTTGAGCGCCTGCACTTCCCTAGAGATAGATTCTGTGAAACCTGTTCCAAATGCATCTCCAAGACCC
TTGATGTAGTCTGTGACACCTTGCTCCATCACATCATCTTCAAGCCAAAGCAAATCTCTGAGGTCTGCAA
AGCCAACCAGACCATCACCGCCCATGGTCACAAGTCCAATCACACCGTGTTGGCACCTGAGAATTCCGCC
ACAATCGCCAGCCTTTGAGATGCCATTAGCCAGTAGAACATGTGACTGATAGCGCTCAGGGTAGTATTCA
TTTGCTTGCACTTGCACTAAACCAGGACCCTGGAAACTGACTGGGTAGTGTTTGTTTTGAGACTTACAGA
AATAGACCCCTGTTTGGCACTTGCATCTGGCAATGGTGTCACAACCGTGGGCTTTGGTGTTGGAAACGAG
GAGATCTCTCTGGTAATCCTCCCACACAAGGTTCTCCCAATCTTCTTCAGTGGCCAAATGTCTGTTAACG
ATCCTGTAGTTGCATACATAAACAGCACCGGATTGTTGACCAAAAGCTCCTGTGGTGGTTATGGTTTTCC
TGCTAGTGCTGGTGCACTTCAACTTGGTACCATCCATATTTGGGTAATTCTTCAGAAGGTACGGTTGACT
CCTGAACGGTCTTGGCACCCATGCTTTGACATGCTTCATCCTCATGTATATTCTTATTGTGATGTTAGAT
GTGACTTGACCTGAGCCAACTGCTCTCACCGCAAAGGTCCCCATAAGGTTATTAGGGCATACCCCATACC
AACTTTCTGTACTTGCGTCATGAGTTCCAAATGTTGGGTAACCATCATAAAACCACTGGTAAGCAGAAGC
AGGTGACATGAAGGGTACAGAAACCTGCGCTGGTGGGTCAGTCATTCTAATAAAGATGGAAGGGTTAGTG
GCTGATTGCCATTCAAAAGAATCTCTTTGCGTTGGTTTGGGCGCACCTGGTGGGATGTACATGTATTGCA
GCATTGTGGAATCTGCTCTGCCATTATCATCAGTAGCAACGAAAGTGAACTCCGCGTCAAAGCGCATGTA
GGTGAAAATTTCTAGTTTTCTTCTTTGCTGGACAAAGCCCATGATGTCTATATCCCAATTGGTAAAGCCA
CTTGATGTTTCTGTCGAGGTGAGTATGGATACTAAACCTACAAGCGCTGCACGCCCGTAGAAGTGTTCAA
CAGAAGTCTCTGCAACAGAGTGCTTGTTAACCACACATCTTGTTTCGATCATACCTTCATCACTGGTATT
TGATGTCGCCCCAGTTTCTGCAGCCTGCAATGCTGGAGTGTTGCCCGTCGAAATGTTGTGGCTACTGGGC
TTGGTATCAGCTGCTGTCAGGGTTTTACTAACCTGACTCTCCACAAATGTCTCTAGTGACGATTCCGGGT
CACCTTGTAAAATGGCTGTTTGTGAGATTGATTCAGTATCCTTGCAGAGTTTCAGTGTGAAGTTAGGTTG
AGCGGCTCCTAATGCAACGATATAAGCACTGGTTGGGGCTCCCTCTGGTACAACATAATTAGTTTGATAC
CATATTGTCACTATTCCACTGGTGTAGTATTCGAAGTATTTGTTAGTTGCCTGTGTGCGGTAGTGAGTGT
TACTGATCCATGGAATAATCATGGTAATAGACGACTGCAAACCAAAATCCCAGATAACATGTGTTCCAAG
CATAGCGATCTCTCTTGATGCTGGTTGTGCGCTACCTGGTGGTGTGTAAGCTATCAACATCTTGCCTGTT
GCCATGAAGGAGCCGGTGAACATGAAAGTCACTTCCAGCGACCCTGACCATTGAGTGTAATAACGGCACA
GCTGACCAACAAGTGTGGACTCCCAAGGTCCTGCGCGGCCAGGGTCCACACGAAAACTGGCGCACAGTTG
ATCCACTGCTGATTGTACACTCACGGGTATGACAAGGCGCTGCATGGGGTCGCTTTCATTTGTGGTGAGG
TTGTTTACTTCGAGTATTGATTCCACTTGGGCAATCTCCAGTAGGTTCTTGACCTCGCCTGGGATGTGTA
TCTCTGGTGTTGGGTGGTACCCAGGTAGGATGGGTGCAGATACCTCGTCATCAGTGGTTAAAAATTGATT

AGAGCCTGGTTTCAGCTCGACTGGGAGGCCCTGGGCAACAGCGTTACGGAGACCTGCAAATTCCGAGTTC
ATTGGAGCAATGGTGACAGTGATCGGTATAGCTGTAGTTGCACCAGTATTAAAATCAAGTGGAGCTACAG
GAATGATAGCTAAGCCAAAGTTGCAGTGGTTCAAGGCGCTGTCATAAGGTACAGCATTGATGTATGGCAT
CACTATTGTGGCACAGTTGTTTGTCCTCAAGTTAATCCACTGGTGCGGGTAAATAAGGGCTTGGGATAAT
GGGACACCTGAATCAAGAACATAGGGTATAACAAAGCTCTTACCATTTTTACCTGGTTGTGCTGCTTCGT
AACTTGGGTGTTTGGCTTCGGCAGGATTAGTTTTTGAGTCAGATCTAGCTACGGTGAATTCTGGGATAGC
GACCACCAGCAAAGTTCCCTGATGGAACTTGCTGGCATTGCACTGGACATGTATGCAAAATCCTGATCTG
TATAGGTAATGGAACTGTGCATTCTGACCAAAAACCCCAGTTTCTGTCAACACATCGGGGAACTTCCAGT
ACCACCCTTTAGATCCCTTTTCCCACAATTTAGCACTTAGGGTGTAAAATCTATTCACTGAGACATCTGG
TCTTGATGGTTTGTCTACGGCTGTAGCGTCAGTGTTGGAACAGAACTCGGGCCACTCACCGTATCCTACT
GTTATATTTGCAGCCTCTTGGGTTGTAATAGTGGAGTTTCCAAGTGTCAGTTGAGCAACTCTGTCACTGT
AACCACAGGCCTCTGCTGAGGGCGATTTAAGTGGTACAGAGGATTCCTTGATCATATCAACCACTGGTTG
GGTAAATTTGGATGGATCTTGGCTGAAATCTTGTTTGGTTGCTGAGGCAGCATAAGATTCTTTGTAATAG
TTTATGGTAGTGTAGTTGATGGTGGAACCACCGACCGCCATGTTGGCGTTTTCATGTGAGCCAGTTTTCT
GAGAAGAGATTTGAGCACCCATGGTAATATTGTTTAGTAATTGACAATTGCTTGGTAGCAATAAGTGAAC
CACACAAAGATATATCCGGATGGCCAATCCAATCGCTATATGATGACAACTCTAGATTGTCACCATAAGC
AGCCAAGGTAAATAAAACAGGAAACACGGACACCCAAAGTAGTCGGTTCCGCCACAGAATTACTCGTTAC
GACCATAACGCCACTGGGTTGTAGCGCATGGCTCCGTGGTTAGGATTAGCCGCATTCAGGGGCCGGAGGA
CTACTACCTAGCTCAATAGGCTCTTCGCACCATGTCATGAGTCAAGCGTCCTGCGAGTAGAACTCGCAGG
CAGGCCGCCAACGCAGCCACCGACACGGTCGCCCGTGGGGAATTCGGTGACTCATCGACCTGAACTACAC
TGGGGTTGTGCTGAACGTAACACTTGAGACATTCAATGATGGTACTAGGCTTCTCGAAGTAAGTAGCCGG
GTAACGAACGTTTTCCCCTTTAGCCGTGGGTACAGCCTATTGGTACTAAGTCCGGGGAAACAGAAGTGCT
TGACTAAAACCCACCTGGGTTGGTGGNNNNNNNNNNNNNNNNNNNNNNNNNNNNNNNNNNNNNNNNNNNN
NNNNNNNNNNNNNNNNNNNNNNNNNNNNNNNNNNNNNNNNNNNNNNNNNNNNNNNNNNNNNNNNNNNNNN
NNNNNNNNNNNNNNNNNNNNNNNNNNNNNNNNNNNNNNNNNNNNNNNNNNNNNNNNNNNNNNNNNNNNNN
NNNNNNNNNNNNNNNNNNNNCAGTGGTTGATATGATCAAGGAATCCTCTGTACCACTTAAATCGCCCTCA
GCAGAGGCCTGTGGTTACAGTGACAGAGTTGCTCAACTGACACTTGGAAACTCCACTATTACAACCCAAG
AGGCTGCAAATATAACAGTAGGATACGGTGAGTGGCCCGAGTTCTGTTCCAACACTGACGCTACAGCCGT
AGACAAACCATCAAGACCAGATGTCTCAGTGAATAGATTTTACACCCTAAGTGCTAAATTGTGGGAAAAG
GGATCTAAAGGGTGGTACTGGAAGTTCCCCGATGTGTTGACAGAAACTGGGGTTTTTGGTCAGAATGCAC
AGTTCCATTACCTATACAGATCAGGATTTTGCATACATGTCCAGTGCAATGCCAGCAAGTTCCATCAGGG
AACTTTGCTGGTGGTCGCTATCCCAGAATTCACCGTAGCTAGATCTGACTCAAAAACTAATCCTGCCGAA
GCCAAACACCCAAGTTACGAAGCAGCACAACCAGGTAAAAATGGTAAGAGCTTTGTTATACCCTATGTTC
TTGATTCAGGTGTCCCATTATCCCAAGCCCTTATTTACCCGCACCAGTGGATTAACTTGAGGACAAACAA
CTGTGCCACAATAGTGATGCCATACATCAATGCTGTACCTTATGACAGCGCCTTGAACCACTGCAACTTT
GGCTTAGCTATCATTCCTGTAGCTCCACTTGATTTTAATACTGGTGCAACTACAGCTATACCGATCACTG
TCACCATTGCTCCAATGAACTCGGAATTTGCAGGTCTCCGTAACGCTGTTGCCCAGGGCCTCCCAGTCGA
GCTGAAACCAGGCTCTAATCAATTTTTAACCACTGATGACGAGGTATCTGCACCCATCCTACCTGGGTAC
CACCCAACACCAGAGATACACATCCCAGGCGAGGTCAAGAACCTACTGGAGATTGCCCAAGTGGAATCAA
TACTCGAAGTAAACAACCTCACCACAAATGAAAGCGACCCCATGCAGCGCCTTGTCATACCCGTGAGTGT
ACAATCAGCAGTGGATCAACTGTGCGCCAGTTTTCGTGTGGACCCTGGCCGCGCAGGACCTTGGGAGTCC
ACACTTGTTGGTCAGCTGTGCCGTTATTACACTCAATGGTCAGGGTCGCTGGAAGTGACTTTCATGTTCA
CCGGCTCCTTCATGGCAACAGGCAAGATGTTGATAGCTTACACACCACCAGGTAGCGCACAACCAGCATC
AAGAGAGATCGCTATGCTTGGAACACATGTTATCTGGGATTTTGGTTTGCAGTCGTCTATTACCATGATT
ATTCCATGGATCAGTAACACTCACTACCGCACACAGGCAACTAACAAATACTTCGAATACTACACCAGTG
GAATAGTGACAATATGGTATCAAACTAATTATGTTGTACCAGAGGGAGCCCCAACCAGTGCTTATATCGT
TGCATTAGGAGCCGCTCAACCTAACTTCACACTGAAACTCTGCAAGGATACTGAATCAATCTCACAAACA
GCCATTTTACAAGGTGACCCGGAATCGTCACTAGAGACATTTGTGGAGAGTCAGGTTAGTAAAACCCTGA
CAGCAGCTGATACCAAGCCCAGTAGCCACAACATTTCGACGGGCAACACTCCAGCATTGCAGGCTGCAGA
AACTGGGGCGACATCAAATACCAGTGATGAAGGTATGATCGAAACAAGATGTGTGGTTAACAAGCACTCT

GTTGCAGAGACTTCTGTTGAACACTTCTACGGGCGTGCAGCGCTTGTAGGTTTAGTATCCATACTCACCT
CGACAGAAACATCAAGTGGCTTTACCAATTGGGATATAGACATCATGGGCTTTGTCCAGCAAAGAAGAAA
ACTAGAAATTTTCACCTACATGCGCTTTGACGCGGAGTTCACTTTCGTTGCTACTGATGATAATGGCAGA
GCAGATTCCACAATGCTGCAATACATGTACATCCCACCAGGTGCGCCCAAACCAACGCAAAGAGATTCTT
TTGAATGGCAATCAGCCACTAACCCTTCCATCTTTATTAGAATGACTGACCCACCAGCGCAGGTTTCTGT
ACCCTTCATGTCACCTGCTTCTGCTTACCAGTGGTTTTATGATGGTTACCCAACATTTGGAACTCATGAC
GCAAGTACAGAAAGTTGGTATGGGGTATGCCCTAATAACCTTATGGGGACCTTTGCGGTGAGAGCAGTTG
GCTCAGGTCAAGTCACATCTAACATCACAATAAGAATATACATGAGGATGAAGCATGTCAAAGCATGGGT
GCCAAGACCGTTCAGGAGTCAACCGTACCTTCTGAAGAATTACCCAAATATGGATGGTACCAAGTTGAAG
TGCACCAGCACTAGCAGGAAAACCATAACCACCACAGGAGCTTTTGGTCAACAATCCGGTGCTGTTTATG
TATGCAACTACAGGATCGTTAACAGACATTTGGCCACTGAAGAAGATTGGGAGAACCTTGTGTGGGAGGA
TTACCAGAGAGATCTCCTCGTTTCCAACACCAAAGCCCACGGTTGTGACACCATTGCCAGATGCAAGTGC
CAAACAGGGGTCTATTTCTGTAAGTCTCAAAACAAACACTACCCAGTCAGTTTCCAGGGTCCTGGTTTAG
TGCAAGTGCAAGCAAATGAATACTACCCTGAGCGCTATCAGTCACATGTTCTACTGGCTAATGGCATCTC
AAAGGCTGGCGATTGTGGCGGAATTCTCAGGTGCCAACACGGTGTGATTGGACTTGTGACCATGGGCGGT
GATGGTCTGGTTGGCTTTGCAGACCTCAGAGATTTGCTTTGGCTTGAAGATGATGTGATGGAGCAAGGTG
TCACAGACTACATCAAGGGTCTTGGAGATGCATTTGGAACAGGTTTCACAGAATCTATCTCTAGGGAAGT
GCAGGCGCTCAAAGACCATCTAATGGGAGCAGAGGGGATAGTGGAAAAGATCCTCAAGAACCTGGTCAAG
ATTGTGTCGGCGCTGGTCATAGTAATCAGGAGTGACTACGACCTAGTCACGATGACTGCAACACTTGCTT
TGATCGGCTGTCACACAAGCCCATGGGCCTGGTTGAAATCTAAGGTATGTAGTTTCCTTGGCCTCCCAAT
AGCACAGAAGCAAGGAGACAATTGGCTAAAGAAATTCAATGACATGGCAAATGCGGCTAAGGGTCTGGAA
TGGGTGGCAATCAAAATCTCCAAGTTTATAGATTGGCTCAAACAGAAAGTTGTCCCAGCCGCCAGGGAAA
AAGTGGAATTCCTCAACAATCTCAAACAACTGCCCTTGTTGGAGAACCAGGTTAGCAATCTAGAACAAGC
AGCAGCTAGCCAGGACGATCTGGAAGCTCTCTTCGGGAATGTGCAGTACCTTGCTATCCATTGCAGGAAA
TACCAACCTTTGTATGCCTCTGAAGCACGCAGAATTGCAACATTGGAACGCAGAGTCAACAACTACATGC
AGTTCAAGAACAAACAACGGATTGAACCTGTATGTTTGATCATTCGTGGCGCTCCAGGTACAGGAAAATC
TCTTGCTACAGGCATCATTGCTAGGGCTATTGCTGAGAAATACCACTCCAGCGTGTATTCCCTGCCGCCT
GACCCTGACCATTTCGACGGTTACCGTCAACAAGTTGTTACTGTAATGGATGACTTATGCCAGAACCCAG
ACGGGAAGGACATCTCCTTGTTTTGTCAAATGGTTTCCACTGTTGAGTTTATACCACCTATGGCATCATT
AGAAGAGAAGGGTCTGCCCTTTACATCAAAGTTTGTCATTGCTTCCACAAATTCTAGCAATATCATAGTC
CCCACTGTTTCAGACAGCGAAGCAATCAGGCGTCGTTTTCACATGGATTGTGACATTGAGGTCCCTGAAT
CTTACAGACTTGAGAATGGGAAGCTGGATGCTTCGAGAGCAGCCAGACTGTGCTCCGAGAACAACACAGC
TAACTTCAAGAAGTGCAGCCCTCTAGTTTGTGGAAAAGCTATTCAGTTAAAAGATAGGAAATCTAAGGTG
AGGTACAGTTTAGATTCAGTTGTCTCAGAACTTATCAGAGAGTACAACAACAGAAACAGTGTTGGTAACA
CCATTGAAGCCCTCTTCCAAGGGCCCCCAGTGTTTAAACCCATCAAGATCTCCATGACAGAACCAGCTCC
CCCAGCTATTGCTGATCTGTTGTCATCAGTTGACAGTGAAGAAGTTAGACAGTATTGTAAGGATAAGGGT
TGGATCATCCCAGATGTGCCCACAAACATTGAAAGACATGTTAACAGAGCTGTTGCAGTTTTGCAATCCA
TCACCACTGTAGTTGCTGTAGTGTCCTTGGTTTACGTCATCTACAAGTTGTTCGCTGGCTTCCAAGGTGC
TTACTCCGGTATGCCCAAAACTGCTTTGAAGAAACCAGTCTTGAGGACTGCTACAGTACAGGGACCCTGT
CTTGACTTTGCCCTGTCATTACTGCGTAGGAACATTAGGCAAGTGCAGACAGACAAGGGCCATTTCACCA
TGCTCGGGGTACGTGATCGCATTGCTGTGTTACCACGCCATGCTGAACCAAGTGGAACCATCTGGATTGA
AGGCAAACAGGTGAAGATCTTGGACGCTGTTGAACTGGTGGATGAGCAACAGGTTAATCTCGAACTCACT
CTCGTCACCCTTGACATGAATGAGAAGTTTAGAGACATCACCAAATTCATTCCAGAGGAAATTGAACCAG
TTTCAGAAGCTACTCTCGTGATTAACACAGAGCAAATGCCCTCCATGTTTGTCCCTGTGGGGAAAGTCGA
AAACTATGGGTTCTTGAACCTAAGTGGGAGACCAACCCACCGTACTCTCATGTACAACTTCCCTACTAAG
GCTGGTCAGTGTGGAGGAGTGGTCACCAGTGTTGGAAAAATCATTGGAATCCATGTTGGTGGAAACGGAC
GCCAAGGCTTCTGTGCTGCTCTGAAGCGCTGCTACTTCACTTCTGAGCAAGGTGAAATTCAGTGGATGAA
ACCAAACAGCGAGACTGGAAGGTACAACATCAACGGGCCCACTAACACCAAGTTGGAGCCAAGCATCTTC
CATGATGTGTTCGAAGGGAAGAAGGAGCCAGCAGCTCTGTCATCTAAGGATAAGAGATTAGAGGTTGATT
TTGAACAGGCCCTTTTCTCCAAGTACATAGGCAACAAAATCCATGAGCCAGATGAGTACATGATTCAAGC

TGCTCATCACTATGCTAATCAATTGAAGCAATTGAATATTGATACATCCAGAATGTCTATGGCTGATGCT
TGCTATGGGACTGAAAACCTAGAAGCTATAGATCTCAGCACCAGTGCAGGATATCCTTACAATGCTCTAG
GTATCAAGAAGAGGCAGATTCTGAACCCCGAGACTCGCGATGTATCCAAAATGAAGTTTTATATGGACAA
GTATGGTCTGGATCTCCCCTACTCTACCTATGTGAAAGATGAACTGAGGAACATCGAAAAGATCAAGAAA
GGTAAATCCCGTTTGATTGAGGCAAGTAGTTTGAATGACTCAGTTTACACCAGAATGTGCTTCGGGCATC
TTTTTGAAACTTTCCATGCTAACCCAGGTACCATCACTGGATCTGCTGTAGGTTGTAACCCAGACACCTT
TTGGAGCAAGATCCCTATACTCTTACCAGGATCCCTCTTTGCATTTGACTACACTGCCTATGATGCAAGT
TTGAGTCCAGCTTGGTTTAGAGCTTTGGAAATAGTGCTTAGGGAGCTAGGGTATGACTCTGAAGCCATCA
ATGTTATTGAAGGTATCAACCACTCACACCACATTTACAGGAACAAGACCTACTGTGTGTTGGGCGGCAT
GCCATCTGGCTGCTCTGGAACTAGCGTGTTCAACTCCATGATTAACAACATTATCATTAGAACCTTACTT
ATTAGAACTTTCAAAGGAATTGATTTAGATGAGTTGAACATGGTTGCTTACGGTGATGATGTTTTGGCAA
GTTACCCCTTCCCAATTGACTGTCAAGAATTGGCTAACACTGGTAAGGAATATGGTTTGGTCATGACCCC
CGCTGATAAGGGCAGTTGTTTCAATGAGGTGACCTGGGAGAATGCAACTTTTCTGAAAAGAGGCTTCAAA
CCTGATGAGCAGTTCCCCTTCCTCATCCACCCAACAATGCCAATGTCAGAGATCCATGAATCCATCAGAT
GGACCAGGGATGCGCGTAACACCCAAGATCACGTTCGCTCTCTGTGCCTTCNNNNNNNNNNNNNNNNNNN
NNNNNNNNNNNNNNNNNNNNNNNNNNNNNNNNNNNNNNNNNNNNNNNNNNNNNNNNNNNNNNNNNNNNNN
NNNNNNNNNNNNNNNNNNNNNNNNNNNNNNNNNNNNNNNNNNNNNNNNNNNNNNNNNNNNNNNNNNNNNN
NNNNNNGAGGAAAGAAAGGGAGTAGAGTTTGATTATGCCTTTTTGGGTGGTTACAGTTATGGAGGCAGTA
ATGTGTGCTTGTGAGATTATTTTAGGTATAGATTTCTCTAGCACTATGCTAATCAATTGAAGCAATTGAA
TATTGATACATCCAGAATGTCTATGGCTGATGCTTGCTATGGGACTGAAAACCTAGAAGCTATAGATCTC
AGCACCAGTGCAGGATATCCTTACAATGCTCTAGGTATCAAGAAGAGGCAGATTCTGAACCCCGAGACTC
GCGATGTATCCAAAATGAAGTTTTATATGGACAAGTATGGTCTGGATCTCCCCTACTCTACCTATGTGAA
AGATGAACTGAGGAACATCGAAAAGATCAAGAAAGGTAAATCCCGTTTGATTGAGGCAAGTAGTTTGAAT
GACTCAGTTTACACCAGAATGTGCTTCGGGCATCTTTTTGAAACTTTCCATGCTAACCCAGGTACCATCA
CTGGATCTGCTGTAGGTTGTAACCCAGACACCTTTTGGAGCAAGATCCCTATACTCTTACCAGGATCCCT
CTTTGCATTTGACTACACTGCCTATGATGCAAGTTTGAGTCCAGCTTGGTTTAGAGCTTTGGAAATAGTG
CTTAGGGAGCTAGGGTATGACTCTGAAGCTATCTATGTTATTGAAGGTATCAACCACTCACACCACATTT
ACAGGAATTTATCCNNNNNNNNNNNNNNNNNNNNNNNNNNNNNNNNNNNNNNNNNNNNNNNNNNNNNNNN
NNNNNNNNNNNNNNNNNNNNNNNNNNNNNNNNNNNNNNNNNNNNNNNNNNNNNNNNNNNNNNNNNNNNNN
NNNNNNNNNNNNNNNNNNNNNNNNNNNNNNNNNNNNNNNNNNNNATCTAAGGTGAGGTACAGTTTAGATT
CAGTTGTCTCAGAACTTATCAGAGAGTACAACAACAGAAACAGTGTTGGTAACACCATTGAAGCCCTCTT
CCAAGGGCCCCCAGTGTTTAAACCCATCAAGATCTCCATGACAGAACCAGCTCCCCCAGCTATTGCTGAT
CTGTTGTCATCAGTTGACAGTGAAGAAGTTAGACAGTATTGTAAGGATAAGGGTTGGATCATCCCAGATG
TGCCCACAAACATTGAAAGACATGTTAACAGAGCTGTTGCAGTTTTGCAATCCATCACCACTGTAGTTGC
TGTAGTGTCCTTGGTTTACGTCATCTACAAGCTGTTCGCTGGCTTCCAAGGTGCTTACTCCGGTATGCCC
AAAACTGCTTTGAAGAAACCAGTCTTGAGGACTGCTACAGTACAGGGACCCTGTCTTGACTTTGCCCTGT
CATTACTGCGTAGGAACATTAGGCAAGTGCAGACAGACAAGGGCCATTTCACCATGCTCGGGGTACGTGA
TCGCATTGCTGTGTTACCACGCCATGCTGAACCAAGTGGAACCATCTGGATTGAAGGCAAACAGGTGAAG
ATCTTGGACGCTGTTGAACTGGTGGATGAGCAACAGGTTAATCTCGAACTCACTCTCGTCACCCTTGACA
TGAATGAGAAGTTTAGAGACATCACCAAATTCATTCCAGAGGAAATTGAACCAGTTTCAGAAGCTACTCT
CGTGATTAACACAGAGCAGNNNNNNNNNNNNNNNNNNNNNNNNNNNNNNNNNNNNNNNNNNNNNNNNNNN
NNNNNNNNNNNNNNNNNNNNNNNNNNNNNNNNNNNNNNNNNNNNNNNNNNNNNNNNNNNNNNNNNNNNNN
NNNNNNNNNNNNNNNNNNNNNNNNNNNNNNNNNNNNNNNNNNNNNNNNNNNNNNNNNNNNNNNNNNNNNN
NAGGATAGAAGAAAGCACAAACGACAAATAGCAAATCCACCAACCCAGGTGGGTTTTAGTCAAGCACTTC
TGTTTCCCCGGACTTAGTACCAATAGGCTGTACCCACGGCTAAAGGGGAAAACGTTCGTTACCCGGCTAC
TTACTTCGAGAAGCCTAGTACCATCATTGAATGTCTCAAGTGTTACGTTCAGCACAACCCCAGTGTAGTT
CAGGTCGATGAGTCACCGAATTCCCCACGGGCGACCGTGTCGGTGGCTGCGTTGGCGGCCTGCCTGCGAG
TTCTACTCGCAGGACGCTTGACTCATGACATGGTGCGAAGAGCCTATTGAGCTAGGTAGTAGTCCTCCGG
CCCCTGAATGCGGCTAATCCTAACCACGGAGCCATGCGCTACAACCCAGTGGCGTTATGGTCGTAACGAG
TAATTCTGTGGCGGAACCGACTACTTTGGGTGTCCGTGTTTCCTGTTTTATTTACCTTGGCTGCTTATGG

TGACAATCTAGAGTTGTCATCATATAGCGATTGGATTGGCCATCCGGATATATCTTTGTGTGGTTCACTT
ATTGCTACCAAGCAATTGTCAATTACTAAACAATATTACCATGGGTGCTCAAATCTCTTCTCAGAAAACT
GGCTCACATGAAAACGCCAACATGGCGGTCGGTGGTTCCACCATCAACTACACTACCATAAACTATTACA
AAGAATCTTATGCTGCCTCAGCAACCAAACAAGATTTCAGCCAAGATCCATCCAAATTTACCCAACCAGT
GGTTGATATGATCAAGGAATCCTCTGTACCACTTAAATCGCCCTCAGCAGAGGCCTGTGGTTACAGTGAC
AGAGTTGCTCAACTGACACTTGGAAACTCCACTATTACAACCCAAGAGGCTGCAAATATAACAGTAGGAT
ACGGTGAGTGGCCCGAGTTCTGTTCCAACACTGACGCTACAGCCGTAGACAAACCATCAAGACCAGATGT
CTCAGTGAATAGATTTTACACCCTAAGTGCTAAATTGTGGGAAAAGGGATCTAAAGGGTGGTACTGGAAG
TTCCCCGATGTGTTGACAGAAACTGGGGTTTTTGGTCAGAATGCACAGTTCCATTACCTATACAGATCAG
GATTTTGCATACATGTCCAGTGCAATGCCAGCAAGTTCCATCAGGGAACTTTGCTGGTGGTCGCTATCCC
AGAATTCACCGTAGCTAGATCTGACTCAAAAACTAATCCTGCCGAAGCCAAACACCCAAGTTACGAAGCA
GCACAACCAGGTAAAAATGGTAAGAACTTTGTTATACCCTATGTTCTTGATTCAGGTGTCCCATTATCCC
AAGCCCTTATTTACCCGCACCAGTGGATTAACTTGAGGACAAACAACTGTGCCACAATAGTGATGCCATA
CATCAATGCTGTACCTTATGACAGCGCCTTGAACCACTGCAACTTTGGCTTAGCTATCATTCCTGTAGCT
CCACTTGATTTTAATACTGGTGCAACTACAGCTATACCGATCACTGTCACCATTGCTCCAATGAACTCGG
AATTTGCAGGTCTCCGTAACGCTGTTGCCCAGGGCCTCCCAGTCGAGCTGAAACCAGGCTCTAATCAATT
TTTAACCACTGATGACGAGGTATCTGCACCCATCCTACCTGGGTACCACCCAACACCAGAGATACACATC
CCAGGCGAGGTCAAGAACCTACTGGAGATTGCCCAAGTGGAATCAATACTCGAAGTAAACAACCTCACCA
CAAATGAAAGCGACCCCATGCAGCGCCTTGTCATACCCGTGAGTGTACAATCAGCAGTGGATCAACTGTG
CGCCAGTTTTCGTGTGGACCCTGGCCGCGCAGGACCTTGGGAGTCCACACTTGTTGGTCAGCTGTGCCGT
TATTACACTCAATGGTCAGGGTCGCTGGAAGTGACTTTCATGTTCACCGGCTCCTTCATGGCAACAGGCA
AGATGTTGATAGCTTACACACCACCAGGTAGCGCACAACCAGCATCAAGAGAGATCGCTATGCTTGGAAC
ACATGTTATCTGGGATTTTGGTTTGCAGTCGTCTATTACCATGATTATTCCATGGATCAGTAACACTCAC
TACCGCACACAGGCAACTAACAAATACTTCGAATACTACACCAGTGGAATAGTGACAATATGGTATCAAA
CTAATTATGTTGTACCAGAGGGAGCCCCAACCAGTGCTTATATCGTTGCATTAGGAGCCGCTCAACCTAA
CTTCACACTGAAACTCTGCAAGGATACTGAATCAATCTCACAAACAGCCATTTTACAAGGTGACCCGGAA
TCGTCACTAGAGACATTTGTGGAGAGTCAGGTTAGTAAAACCCTGACAGCAGCTGATACCAAGCCCAGTA
GCCACAACATTTCGACGGGCAACACTCCAGCATTGCAGGCTGCAGAAACTGGGGCGACATCAAATACCAG
TGATGAAGGTATGATCGAAACAAGATGTGTGGTTAACAAGCACTCTGTTGCAGAGACTTCTGTTGAACAC
TTCTACGGGCGTGCAGCGCTTGTAGGTTTAGTATCCATACTCACCTCGACAGAAACATCAAGTGGCTTTA
CCAATTGGGATATAGACATCATGGGCTTTGTCCAGCAAAGAAGAAAACTAGAAATTTTCACCTACATGCG
CTTTGACGCGGAGTTCACTTTCGTTGCTACTGATGATAATGGCAGAGCAGATTCCACAATGCTGCAATAC
ATGTACATCCCACCAGGTGCGCCCAAACCAACGCAAAGAGATTCTTTTGAATGGCAATCAGCCACTAACC
CTTCCATCTTTATTAGAATGACTGACCCACCAGCGCAGGTTTCTGTACCCTTCATGTCACCTGCTTCTGC
TTACCAGTGGTTTTATGATGGTTACCCAACATTTGGAACTCATGACGCAAGTACAGAAAGTTGGTATGGG
GTATGCCCTAATAACCTTATGGGGACCTTTGCGGTGAGAGCAGTTGGCTCAGGTCAAGTCACATCTAACA
TCACAATAAGAATATACATGAGGATGAAGCATGTCAAAGCATGGGTGCCAAGACCGTTCAGGAGTCAACC
GTACCTTCTGAAGAATTACCCAAATATGGATGGTACCAAGTTGAAGTGCACCAGCACTAGCAGGAAAACC
ATAACCACCACAGGAGCTTTTGGTCAACAATCCGGTGCTGTTTATGTATGCAACTACAGGATCGTTAACA
GACATTTGGCCACTGAAGAAGATTGGGAGAACCTTGTGTGGGAGGATTACCAGAGAGATCTCCTCGTTTC
CAACACCAAAGCCCACGGTTGTGACACCATTGCCAGATGCAAGTGCCAAACAGGGGTCTATTTCTGTAAG
TCTCAAAACAAACACTACCCAGTCAGTTTCCAGGGTCCTGGTTTAGTGCAAGTGCAAGCAAATGAATACT
ACCCTGAGCGCTATCAGTCACATGTTCTACTGGCTAATGGCATCTCAAAGGCTGGCGATTGTGGCGGAAT
TCTCAGGTGCCAACACGGTGTGATTGGACTTGTGACCATGGGCGGTGATGGTCTGGTTGGCTTTGCAGAC
CTCAGAGATTTGCTTTGGCTTGAAGATGATGTGATGGAGCAAGGTGTCACAGACTACATCAAGGGTCTTG
GAGATGCATTTGGAACAGGTTTCACAGAATCTATCTCTAGGGAAGTGCAGGCGCTCAAAGACCATCTAAT
GGGAGCAGAGGGGATAGTGGAAAAGATCCTCAAGAACCTGGTCAAGATTGTGTCGGCGCTGGTCATAGTA
ATCAGGAGTGACTACGACCTAGTCACGATGACTGCAACACTTGCTTTGATCGGCTGTCACACAAGCCCAT
GGGCCTGGTTGAAATCTAAGGTATGTAGTTTCCTTGGCCTCCCAATAGCACAGAAGCAAGGAGACAATTG
GCTAAAGAAATTCAATGACATGGCAAATGCGGCTAAGGGTCTGGAATGGGTGGCAATCAAAATCTCCAAG

TTTATAGATTGGCTCAAACAGAAAGTTGTCCCAGCCGCCAGGGAAAAAGTGGAATTCCTCAACAATCTCA
AACAACTGCCCTTGTTGGAGAACCAGGTTAGCAATCTAGAACAAGCAGCAGCTAGCCAGGACGATCTGGA
AGCTCTCTTCGGGAATGTGCAGTACCTTGCTATCCATTGCAGGAAATACCAACCTTTGTATGCCTCTGAA
GCACGCAGAATTGCAACATTGGAACGCAGAGTCAACAACTACATGCAGTTCAAGAACAAACAACGGATTG
AACCTGTATGTTTGATCATTCGTGGCGCTCCAGGTACAGGAAAATCTCTTGCTACAGGCATCATTGCTAG
GGCTATTGCTGAGAAATACCACTCCAGCGTGTATTCCCTGCCGCCTGACCCTGACCATTTCGACGGTTAC
CGTCAACAAGTTGTTACTGTAATGGATGACTTATGCCAGAACCCAGACGGGAAGGACATCTCCTTGTTTT
GTCAAATGGTTTCCACTGTTGAGTTTATACCACCTATGGCATCATTAGAAGAGAAGGGTCTGCCCTTTAC
ATCAAAGTTTGTCATTGCTTCCACAAATTCTAGCAATATCATAGTCCCCACTGTTTCAGACAGCGAAGCA
ATCAGGCGTCGTTTTCACATGGATTGTGACATTGAGGTCCCTGAATCTTACAGACTTGAGAATGGGAAGC
TGGATGCTTCGAGAGCAGCCAGACTGTGCTCCGAGAACAACACAGCTAACTTCAAGAAGTGCAGCCCTCT
AGTTTGTGGAAAAGCTATTCAGTTAAAAGATAGGAAATCTAAGGTGAGGTACAGTTTAGATTCAGTTGTC
TCAGAACTTATCAGAGAGTACAACAACAGAAACAGTGTTGGTAACACCATTGAAGCCCTCTTCCAAGGGC
CCCCAGTGTTTAAACCCATCAAGATCTCCATGACAGAACCAGCTCCCCCAGCTATTGCTGATCTGTTGTC
ATCAGTTGACAGTGAAGAAGTTAGACAGTATTGTAAGGATAAGGGTTGGATCATCCCAGATGTGCCCACA
AACATTGAAAGACATGTTAACAGAGCTGTTGCAGTTTTGCAATCCATCACCACTGTAGTTGCTGTAGTGT
CCTTGGTTTACGTCATCTACAAGTTGTTCGCTGGCTTCCAAGGTGCTTACTCCGGTATGCCCAAAACTGC
TTTGAAGAAACCAGTCTTGAGGACTGCTACAGTACAGGGACCCTGTCTTGACTTTGCCCTGTCATTACTG
CGTAGGAACATTAGGCAAGTGCAGACAGACAAGGGCCATTTCACCATGCTCGGGGTACGTGATCGCATTG
CTGTGTTACCACGCCATGCTGAACCAAGTGGAACCATCTGGATTGAAGGCAAACAGGTGAAGATCTTGGA
CGCTGTTGAACTGGTGGATGAGCAACAGGTTAATCTCGAACTCACTCTCGTCACCCTTGACATGAATGAG
AAGTTTAGAGACATCACCAAATTCATTCCAGAGGAAATTGAACCAGTTTCAGAAGCTACTCTCGTGATTA
ACACAGAGCAAATGCCCTCCATGTTTGTCCCTGTGGGGAAAGTCGAAAACTATGGGTTCTTGAACCTAAG
TGGGAGACCAACCCACCGTACTCTCATGTACAACTTCCCTACTAAGGCTGGTCAGTGTGGAGGAGTGGTC
ACCAGTGTTGGAAAAATCATTGGAATCCATGTTGGTGGAAACGGACGCCAAGGCTTCTGTGCTGCTCTGA
AGCGCTGCTACTTCACTTCTGAGCAAGGTGAAATTCAGTGGATGAAACCAAACAGCGAGACTGGAAGGTA
CAACATCAACGGGCCCACTAACACCAAGTTGGAGCCAAGCATCTTCCATGATGTGTTCGAAGGGAAGAAG
GAGCCAGCAGCTCTGTCATCTAAGGATAAGAGATTAGAGGTTGATTTTGAACAGGCCCTTTTCTCCAAGT
ACATAGGCAACAAAATCCATGAGCCAGATGAGTACATGATTCAAGCTGCTCATCACTATGCTAATCAATT
GAAGCAATTGAATATTGATACATCCAGAATGTCTATGGCTGATGCTTGCTATGGGACTGAAAACCTAGAA
GCTATAGATCTCAGCACCAGTGCAGGATATCCTTACAATGCTCTAGGTATCAAGAAGAGGCAGATTCTGA
ACCCCGAGACTCGCGATGTATCCAAAATGAAGTTTTATATGGACAAGTATGGTCTGGATCTCCCCTACTC
TACCTATGTGAAAGATGAACTGAGGAACATCGAAAAGATCAAGAAAGGTAAATCCCGTTTGATTGAGGCA
AGTAGTTTGAATGACTCAGTTTACACCAGAATGTGCTTCGGGCATCTTTTTGAAACTTTCCATGCTAACC
CAGGTACCATCACTGGATCTGCTGTAGGTTGTAACCCAGACACCTTTTGGAGCAAGATCCCTATACTCTT
ACCAGGATCCCTCTTTGCATTTGACTACACTGCCTATGATGCAAGTTTGAGTCCAGCTTGGTTTAGAGCT
TTGGAAATAGTGCTTAGGGAGCTAGGGTATGACTCTGAAGCCATCAATGTTATTGAAGGTATCAACCACT
CACACCACATTTACAGGAACAAGACCTACTGTGTGTTGGGCGGCATGCCATCTGGCTGCTCTGGAACTAG
CGTGTTCAACTCCATGATTAACAACATTATCATTAGAACCTTACTTATTAGAACTTTCAAAGGAATTGAT
TTAGATGAGTTGAACATGGTTGCTTACGGTGATGATGTTTTGGCAAGTTACCCCTTCCCAATTGACTGTC
AAGAATTGGCTAACACTGGTAAGGAATATGGTTTGGTCATGACCCCCGCTGATAAGGGCAGTTGTTTCAA
TGAGGTGACCTGGGAGAATGCAACTTTTCTGAAAAGAGGCTTCAAACCTGATGAGCAGTTCCCCTTCCTC
ATCCACCCAACAATGCCAATGTCAGAGATCCATGAATCCATCAGATGGACCAGGGATGCGCGTAACACCC
AAGATCACGTTCGCTCTCTGTGCCTTCTTGCCTGGCATAATGGGAAAGAAACATATGACCAATTTGTCAA
GAAAATCAGATCAGTGCCTATTGGCTTGGCACTGTCCATACCAGAATACAACNNNNNNNNNNNNNNNNNN
NNNNNNNNNNNNNNNNNNNNNNNNNNNNNNNNNNNNNNNNNNNNNNNNNNNNNNNNNNNNNNNNNNNNNN
NNNNNNNNNNNNNNNNNNNNNNNNNNNNNNNNNNNNNNNNNNNNNNNNNNNCTGAGCACTATTTCCAAAGCT
CTAAACCAAGCTGGACTCAAACTTGCATCATAGGCAGTGTAGTCAAATGCAAAGRGRKATCCTGGTAAGA
GTATAGGGATCTTGCTCCAGAAGGTGTCTGGGTTACAGCCTWCAGCAGATCCAGWGAWGGYMCCKGGGTW
AGCATGGAAAGTTTCAAAAAGGTGCCCGAAACACATTCTGGTGTAAACTGAGTCATTCAAACTGCTTGCC

TCAATCAAACGGGATTTACCTTTCTTGATCTTCTCAATGCTCCTCAGTTCATCTTTCACATAGGTAGAGT
AGGGGAGATCCAGACCATACTTATCCATATAGAACTTCATTTTGGACACATCGCGGGTTTCGGGGTTCAA
GATCTGCCTCTTCTTGATACCTAGAGCATTGTAAGGATATCCTGCGCTGGTGCTGAGATCTATAGCTTCT
AGGTTGTCAGTCCCGTAGCAAGCATCAGCCATAGACATTCTGGATGTATCAATGTTCAATTGCTTCAACT
GATTAGCATAGTGATGGGCAGCTTGGATCATGTATTCATCTGGTTCATGGATTTTGTTGCCTATGTACTT
GGAGAAAAGGGCCTGTTCAAAATCAACTTCCAATCTCTTGTCCTTAGATGACAGTGCTGCTGGTTCCTTT
TTCCCTTCAAACACATCATGGAAGATGCTTGGTTCCAACTTGGTGTTAGTGGGCCCGTTGATGTTGTACC
TTCCAGTCTCACTGTTTGGTTTCATCCACTGAATTTCACCTTGTTCGGAAGTGAAGTAGCAACGCTTCAA
AGCAGCACAGAAGCCTTGGCGTCCGTTTCCACCAACGTGGATTCCAATGATTTTCCCAACGCTGGTGACC
ACTCCTCCACACTGACCAGCCTTGGTGGGGAAGTTGTACATGAGAGTACGGTGGGTTGGTCTTCCACTTA
GGTTCAGGAACCCGTAGTTTTCAACTTTCCCCACAGGGACAAACATGGAAGGCATTTGCTCCGTGTTGAT
CACAAGAGTAGCTTCTGAAACTGGTTCGATTTCCTCTGGGATGAATTTGGTAATGTCTCTAAACTTCTCA
TTCATGTCAAGGGTGACGAGAGTGAGTTCAAGATTGACTTGTTGTTCATCTACCAATTCAACAGCGTCCA
AGATCTTCACCTGCTTGCCTTCAATCCAGATGGTTCCACTTGGTTCAGCATGGCGCGGCAACACAGCAAT
GCGATCACGCACTCCGAGCATGGTAAAATGGCCCTTGTCTGTCTGCACTTGCCTAATGTTCCTACGGAGT
AATGATAGGGCAAAATCAAGGCAGGGTCCCTGCACTGTGGCGGTCCTCAAGACTGGTTTCTTCAAAGCGG
TTTTGGGCATACCGGAGTAAGCGCCCTGGAAGCCAGCGAACAACTTGTAGATAACATAAACCAAGGACAC
TACAGCAACCACAGTGGKGATGGATTGCAAAACTGCAACAGCTCTGTTAACATGTCTTTCATTGTTTGTA
GGCACATCTGGGATGATBCACCCNNNNNNNNNNNNNNNNNNNNNNNNNNNNNNNNNNNNNNNNNNNNNNN
NNNNNNNNNNNNNNNNNNNNNNNNNNNNNNNNNNNNNNNNNNNNNNNNNNNNNNNNNNNNNNNNNNNNNN
NNNNNNNNNNNNNNNNNNNNNNNNNNNTGGTGTGTGTGGTTGGTGCCTTCAATAACATTAATGGCTTCAG
AGTCATACCCTAGCTCCCTGAGCACTATTTCCAAAGCTCTAAACCAAGCTGGACTCAAACTTGCATCATA
GGCAGTGTAGTCAAATGCAAAGAGGGATCCTGGTAAGAGTATAGGGATCTTGCTCCAGAAGGTGTCTGGG
TTACAGCCTACAGCAGATCCAGTGATGGTACCTGGGTTAGCATGGAAAGTTTCAAAAAGGTGCCCGAAAC
ACATTCTGGTGTAAACTGAGTCATTCAAACTGCTTGCCTCAATCAAACGGGATTTACCTTTCTTGATCTT
CTCAATGCTCCTCAGTTCATCTTTCACATAGGTAGAGTAGGGGAGATCCAGACCATACTTATCCATATAG
AACTTCATTTTGGACACATCGCGGGTTTCGGGGTTCAAGATCTGCCTCTTCTTGATACCTAGAGCATTGT
AAGGATATCCTGCGCTGGTGCTGAGATCTATAGCTTCTAGGTTGTCAGTCCCGTAGCAAGCATCAGCCAT
AGACATTCTGGATGTATCAATGTTCAATTGCTTCAACTGATTAGCATAGTGATGGGCAGCTTGGATCATG
TATTCATCTGGTTCATGGATTTTGTTGCCTATGTACTTGGAGAAAAGGGCCTGTTCAAAATCAACTTCCA
ATCTCTTGTCCTTAGATGACAGTGCTGCTGGTTCCTTTTTCCCTTCAAACACATCATGGAAGATGCTTGG
TTCCAACTTGGTGTTAGTGGGCCCGTTGATGTTGTACCTTCCAGTCTCACTGTTTGGTTTCATCCACTGA
ATTTCACCTTGTTCGGAAGTGAAGTAGCAACGCTTCAAAGCAGCACAGAAGCCTTGGCGTCCGTTTCCAC
CAACGTGGATTCCAATGATTTTCCCAACGCTGGTGACCACTCCTCCACACTGACCAGCCTTGGTGGGGAA
GTTGTACATGAGAGTACGGTGGGTTGGTCTTCCACTTAGGTTCAGGAACCCGTAGTTTTCAACTTTCCCC
ACAGGGACAAACATGGAAGGCATTTGCTCCGTGTTGATCACAAGAGTAGCTTCTGAAACTGGTTCGATTT
CCTATGGGRAGAATTTGGTAATGTCTCTNNNNNNNNNNNNNNNNNNNNNNNNNNNNNNNNNNNNNNNNNN
NNNNNNNNNNNNNNNNNNNNNNNNNNNNNNNNNNNNNNNNNNNNNNNNNNNNNNNNNNNNNNNNNNNNNN
NNNNNNNNNNNNNNNNNNNNNNNNNNNNNNNNNNNNNNNNCTTGTACTAAGCCGGGACCCTGGAAACTGA
CTGGGTAGTGTTTGTTTTGGGACTTGCAGAAGTAGACCCCTGTTTGGCATTTGCATCTAGCAATGGTATC
GCAACCGTGGGCATTGGTGTTGGAGACGAGGAGATCTCTCTGGTAATCTTCCCACACAAGGTTTTCCCAA
TCTTCATTAGTGGCCAAGTGCCTGTTGACAATTCTGTAGTTGCAAACATAGACAGCACCAGATTGTTGGC
CAAAAACCCCTGTGGTGGTTATTCCAGTCCTACTAGTACTAGTACACTTCAAGTTGCTACCATCCATGTT
GGGGTAATTCTTCAAAAGGTATGGTTGACTCCTGAATGGCCTTGGTATCCAAGCTTTAACATGCTTCATC
CTCATGTAAATTCTTACTGTGATGTTTGATGTAATTCTACCTGAACCAACTGCTCTCACCGCAAAGGTCC
CCATGAGGTTGTTAGGACAGACTCCATACCAACTGTCTGTGCTGGCAGTGTGGGTTCCAAATGTTGGGTA
ACCGTCATAGAACCACTGGTAAGCGGAAGCAGGCGACATGAAGGGTACAGAAACCTGTGCAGGTGGGTCA
GTCATTCTAACAAAGATAGAAGGGTTGGTTGCTGATTGCCATTCAAAAGAGTCCCTTTGTGTTGGTTTGG
GTGCACCCGGTGGGATGTACATGTACTGCAACATTGTAGAATCTGCTTTGCCGTCATCAGCAGTAGCAAT
AAAAGTGAACTCAGCATCGAAGCGCATGTAGGTGAAGATCTCAAGTTTCCTTCTTTGCTGAACAAAGCCC

ATGATATCTATATCCCAATTAGTGAAGCCATTTGCTGTATCTGCCGAGGTGAGTATGGATACTAAGCCCA
CGAGTGCCGCACGCCCATAGAAGTGTTCAATAGAAGTTTCTGCGACAGAGTGCTTGTTAACCACACATCT
TGTCTCAATCATACCCTCATCACTGGTGTTTGATGTTGCCCCAGTTTCTGCAGCTTGCAGCGCTGGGGTG
TTGCCTGTTGAAATGTTGTGACTACTGGGCTTAGTGTCAGCTGCTGTCAAGGTCTTGCTGACTTGGCTCT
CCACAAATGTCTCTAGTGAAGATTCTGGGTCACCCTGCAAAATGGCTGTCTGTGAGATGGAGTCAGTATC
CTTACAAAGTTTCAGTGTGAAGTTAGGTTGGGCAGCTCCCAGCGCAATGATGTAGGCACTGGTGGGGGCT
CCCTCAGGCACAACATAATTGGTTTGATACCATATTGTCACTATTCCACTGGTGTAGTACTCAAAGTATT
TATCAGTTGCCTGTGTTCTGTAGTGAGTGTTGCTAATCCATGGGATAATCATGGTAACAGACGATTGCAA
GCCAAAATCCCAGATGACATGTGTTCCGAGCATAGCGATCTCTCTTGATGCTGGTTGTGCACCACCTGGT
GGTGTGTAAGCTATCAGCATCTTACCTGTTGCCATGAAAGAACCAGTGAACATGAAAGTCACTTCTAGTG
ACCCCGACCATTGAGTGTAGTAGCGGCACAGCTGGCCAACAAGTGTAGATTCCCAAGGCCCTGCACGGCC
AGGGTCCACACGAAAGCTAGCGNNNNNNNNNNNNNNNNNNNNNNNNNNNNNNNNNNNNNNNNNNNNNNNN
NNNNNNNNNNNNNNNNNNNNNNNNNNNNNNNNNNNNNNNNNNNNNNNNNNNNNNNNNNNNNNNNNNNNNN
NNNNNNNNNNNNNNNNNNNNNNNNNNNNNNNNNNNNNNNNNNNNTGGTGTGAGTGGTTGATACCTTCAAT
AACATTAATGGCTTCAGAGTCATACCCTAGCTCCCTGAGCACTATTTCCAAAGCTCTAAACCAAGCTGGA
CTCAAACTTGCATCATAGGCAGTGTAGTCAAATGCAAAGAGGGATCCTGGTAAGAGTATAGGGATCTTGC
TCCAGAAGGTGTCTGGGTTACAGCCTACAGCAGATCCAGTGATGGTACCTGGGTTAGCATGGAAAGTTTC
AAAAAGGTGCCCGAAACACATTCTGGTGTAAACTGAGTCATTCAAACTGCTTGCCTCAATCAAACGGGAT
TTACCTTTCTTGATCTTCTCAATGCTCCTCAGTTCATCTTTCACATAGGTAGAGTAGGGGAGATCCAGAC
CATACTTATCCATATAGAACTTCATTTTGGACACATCGCGGGTTTCGGGGTTCAAGATCTGCCTCTTCTT
GATACCTAGAGCATTGTAAGGATATCCTGCGCTGGTGCTGAGATCTATAGCTTCTAGGTTGTCAGTCCCG
TAGCAAGCATCAGCCATAGACATTCTGGATGTATCAATGTTCAATTGCTTCAACTGATTAGCATAGTGAT
GGGCAGCTTGGATCATGTATTCATCTGGTTCATGGATTTTGTTGCCTATGTACTTGGAGAAAAGGGCCTG
TTCAAAATCAACTTCCAATCTCTTGTCCTTAGATGACAGTGCTGCTGGTTCCTTTTTCCCTTCAAACACA
TCATGGAAGATGCTTGGTTCCAACTTGGTGTTAGTGGGCCCGTTGATGTTGTACCTTCCAGTCTCACTGT
TTGGTTTCATCCACTGAATTTCACCTTGTTCGGAAGTGAAGTAGCAACGCTTCAAAGCAGCACAGAAGCC
TTGGCGTCCGTTTCCACCAACGTGGATTCCAATGATTTTCCCAACGCTGGTGACCACTCCTCCACACTGA
CCAGCCTTGGTGGGGAAGTTGTACATGAGAGTACGGTGGGTTGGTCTTCCACTTAGGTTCAGGAACCCGT
AGTTTTCAACTTTCCCCACAGGGACAAACATGGAAGGCATTTGCTCCGTGTTGATCACAAGAGTAGCTTC
TGAAACTGGTTCGATTTCCTCTGGGATGAATTTGGTAATGTCTCTAAACTTCTCATTCATGTCAAGGGTG
ACGAGAGTGAGTTCAAGATTGACTTGTTGTTCATCTACCAATTCAACAGCGTCCAAGATCTTCACCTGCT
TGCCTTCAATCCAGATGGTTCCACTTGGTTCAGCATGGCGCGGCAACACAGCAATGCGATCACGCACTCC
GAGCATGGTAAAATGGCCCTTGTCTGTCTGCACTTGCCTAATGTTCCTACGGAGTAATGATAGGGCAAAA
TCAAGGCAGGGTCCCTGCACTGTGGCGGTCCTCAAGACTGGTTTCTTCAAAGCGGTTTTGGGCATACCGG
AGTAAGCGCCCTGGAAGCCAGCGAACAACTTGTAGATAACATAAACCAAGGACACTACAGCAACCACAGT
GGTGATGGATTGCAAAACTGCAACAGCTCTGTTAACATGTCTTTCAATGTTTGTAGGCACATCTGGGATG
ATCCAACCCTTATCCTTACAATACTGTCTGACTTCTTCACTGTCAACTGATGACAACAAATCAGCAATAG
CTGGGGGGGCCGGTTCTGTCATGGAGATTTTGATGGGCTTGAACACCGGGGGCCCTTGGAAGAGGGCTTC
AATGGTGTTGCCAACACTGTTTCTGTTGTTGTACTCTCTGATAAGTTCTGAGACAACTGAATCTAAACTG
TATCTCACCTTAGATTTCCTATCTTTCAACTGAATAGCTTTTCCACAAACTAGGGGACTGCACTTCTTAA
AGTTGGCTGTGTTGTTCTCAGAGCACAGTCTGGCTGCTCTCGAGGCATCCAACTTCCCATTCTCAAGCCT
GTAAGATTCAGGGACCTCAATGTCACAATCCATGTAAAAGCGGCGCCTGATTGCTTCGCTATCTGAAACA
GTGGGGACTATGATATTGCTAGAATTTGTGGAAGCAATGACGAACTTTGATGTAAAGGGCAGACCCTTCT
CTTCTAAAGATGCCATAGGTGGTATAAACTCAACAGTAGAAACCATTTGGCAAAACAAGGAGATGTCCTT
CCCGTCTGGGTTCTGGCACAAGTCATCCATCACAGTGACAACTTGTTGACGGTAACCGTCGAAGTGGTCA
GGGTCAGGTGGTAGGGAGTATACGCTGGAGTGGTATTTTTCAGCAATAGCCCTAGCAATGATGCCTGTAG
CAAGAGATTTTCCTGTACCTGGAGCGCCACGAATGATCAAACATACAGGTTCAATCCGTTGTTTGTTCTT
GAACTGCATGTAGTTGTTGACTCTGCGTTCCAATGTTGCAATTCTGCGTGCTTCAGAGGCATACAAAGGT
TGGTATTTCCTGCAATGGATAGCAAGGTACTGCACATTCCCGAAGAGAGCTTCTAGATCGTCCTGGCTAG
CTGCTGCTTGCTCTAAGTTGCTAACCTGGTTCTCCAACAAGGGCAGTTGTTTGAGATTATTAAGGAACTC

TACTTTTTCCTTGGCGGCTGGGACAACTTTTTGCTTAAGCCAATCTATGAACTTGGAGATTTTGATTGCT
ACCCATTCTAGACCCTTAGCCGCATTTGCCATGTCATTGAATTTCTTTAGCCAATTGTCTCCTTGTTTCT
GTGCTATCGGGAGGCCAAGGAAGCTACATACCTTAGATTTCAACCAGGCCCATGGGCTTGTGTGACAACC
GATCAAAGCAAGTGTTGCAGTCATCGTGACTAAGTCGTAATCACTCCTGATTACTATGACCAGCGCCGAC
ACAATCTTGACCAGGTTCTTGAGGATCTTTTCCACTATCCCCTCTGCTCCCATTAGATGGTCTTTGAGCG
CCTGCACTTCCCTAGAGATAGATTCTGTAAAACCTGTCCCAAATGCATCTCCAAGACCCTTGATGTAATC
TGTGACACCTTGTTCCATCACATCATCTTCGAGCCAAAGCAGGTCTCTGATGTCTGCAAAGCCAACCAGG
CCGTCACCGCCCATTGTCACAAGTCCAATCACACCGTGTTGGCACCTGAGAATTCCACCACAATCACCAG
CCTTTGAGATGCCATTAGCCAACAGAACATGTGNNNNNNNNNNNNNNNNNNNNNNNNNNNNNNNNNNNNN
NNNNNNNNNNNNNNNNNNNNNNNNNNNNNNNNNNNNNNNNNNNNNNNNNNNNNNNNNNNNNNNNNNNNNN
NNNNNNNNNNNNNNNNNNNNNNNNNNNNNNNNNNNNACGGCATTGCGGAGACCTGCAAATTCCGAATTCA
TTGGGGCAATGGTAACGGTGATTGGTATAGCTGTAGTTGCACCTGTATTAAAGTCAAGTGGTGCCACAGG
GATGATAACTAAGCCAAAGTTACAGTGATTCAAAGCACTATCATAGGGTACGGCATTGACATATGGCATC
ACTATTGTAGCACAGTTATTTGTCCTCAGGTTAATCCACTGGTGTGGGTAGATAAGAGCCTGGGATAGTG
GGACACCTGAATCAAGGACATAAGGTATAGCAAGTTCCTTACCGTTTTTGCCTGGCTGAGCTGCTGCATA
AGATGGGTGTTTTGCATTGGCAGGGTCAGTTGCGCTGTCAGAACGGGCCACAGTGAATTCTGGGATAGCA
GCGACTAACAAGGTCCCCTGATGGAACTTGCTCGCATTACATTGTACATGTATGCAGAAACCTGATCTAT
ATAGGTAGTGAAACTGTGCATTCTGCCCGAAAACACCTGTCTCTGTTAGTACATCGGGGAATTTCCAGTA
CCATCCGTTGGAGGTTTTTTCCCATAATTTAGCACTTAGGGTGTAAAACCTGTTCACTGATACATCTGGT
CTAGATGGCTTGTCCACAGCTGTTGCATCTGTGTTTGAGCAAAATTCAGGCCATTCACCATAGCCTACTG
TTATGTTGGCAGCTTCTTGGGTTGTGATGGTGGAATTACCTAGAGTAAGTTGGGCCACTCTATCGCTGTA
ACCACACGCTTCGGCTGAGGGGGATTTGAGAGGTACAGAAGACTCCTTTATCATATCCACTACAGGTTGA
GTAAACTTAGATGGGTCCTGACTGAAGTCTTGTTTAGTTGCTGAAGCAGCATAAGATTCTTTGTAATAAT
TTATGGTGGTGTAATTGATGGTGGAACCACCAGTCGCCATGTTGGCATTCTCGTGAGAGCCAGTTTTCTG
AGTGGAAACTTGGGCACCCATAATGTTGTTGATAAATGATAATTGCTTGGTAGCAAGAGGTAAATCACAC
AAAAGATGTATCCGGATGGCCAATCCAATCGCTATATGATGACAACTCTAGATTGTCACCATAAGCAGCC
AAGGTAAATAAAACAGGAAACACGGACACCCAAAGTAGTCGGTTCCGCCACAGAATTACTCGTTACGACC
ACAACACCACTGGATTGTAGTGCATGGCTCCGTGGTTAGGATTAGCCGCATTCAGGGGCCGGAGGACTAC
TACCTAGCTCAATAGGCTCTTCGCACCATGTCATGAATCAAGCGTCCTGCGAGTGAGACTCGCAGGCAGG
CCGCCAACGCAGCCACCGACACGGTCGCCCGTGGGGAATTCGGTGACTCATCGACCTGAACTACACTGGG
GTTGTGCTGAGCGTAACACTCGAGACATTCAATGGTGGTACTAGGCTTCTCGAAGTAGGTAGCCGGATAA
CGAACGTTTTCCCTTTCGGCCGTGGGCACAGCCTATTGGTACTAAGTCCGGGGAAACAGAAGTGCTTGAC
TAAAAACCCACCTAT

>Enterovirus A SV46
AAGTTATCATACTGAGGAATTGACAGCACCTTGCCAATTGGCACTGTTCTGATCTTTTCAACAAATTCAT
TGTACTCCTCCCTCCCAGCGTGCCAAGCCAAGAGACACAGCGAATGCACATGATCTTGAGTGTTACGTGC
GTCCTTAGTCCAGCGAATGGACTCGTGAATCTCTGACATGGGCATGACTGGGTGGATCAGGAATGGAAAT
TGTTCATCTGGTTTGAAACTTCTCTTCAAGAATGTTGCATTTTCCCAAGTGACCTCATTGAAGCAAGAAG
ATTTGTCTGCAGGTGTCATGACTAAACCATATTCCCTACCAGTCCTTGCTAACTCAGCGCAGTCGATTGG
GAACGGGTAGCTAGCTAACACATCATCTCCATAAGCAATCATGTTTAATTCATCTAAATCAATGCCCTTG
AATGTTCTAATTAACAGGGTCCTAATGATAATATTATTGATCATGGTATTGAATATACTGGTCCCAGAGC
AGCCTGAGGGCATGCCACCAACCACGCAGTATGTTTTGTTGCGGTAGATGTGGTGTGAGTGGTTGATTCC
TTCAATAACATTGATGGCTTCAGAGTCATACCCTAGCTCTCTAAGCACTATTTCCAAAGCTCTAAACCAA
GCGGGACTCAAACTTGCATCATAAGCAGTGTAGTCAAATGCAAAGAGGGATCCTGGTAAGAGTATAGGGA
TCTTGCTCCAGAAGGTGTCTGGGTTACAACCTACAGCAGATCCAGTGATGGTACCTGGGTTAGCATGGAA
AGTTTCAAAAAGATGCCCGAAACACATTCTGGTGTAAACTGAGTCATTCAAACTGCTTGCCTCAATCAAA
CGGGATTTACCTTTCTTGATCTTCTCAATGCTCCTCAGTTCATCTTTCACATAGGTAGAGTAGGGGAGAT
CCAGACCATACTTATCCATATAGAACTTCATTTTGGACACATCGCGAGTCTCGGGGTTCAAAATCTGTCT
CTTCTTGATACCTAGAGCATTGTAAGGGTATCCTGCACTAGTGCTGAGATCTATGGCTTCTAGGTTATCA

GTCCCATAGCAAGCATCAGCCATAGACATTCTGGATGTATCAATGTTCAATTGCTTCAACTGATTAGCAT
AGTGATGGGCGAGNNNNNNNNNNNNNNNNNNNNNNNNNNNNNNNNNNNNNNNNNNNNNNNNNNNNNNNN
NNNNNNNNNNNNNNNNNNNNNNNNNNNNNNNNNNNNNNNNNNNNNNNNNNNNNNNNNNNNNNNNNNNNNN
NNNNNNNNNNNNNNNNCTACGCAGTATGTTTTGTTGCGGTAGAAGTGGTGGGAGTGGTTAATACCCTCAA
TGAGGCTGACAGCCTCATCGTCATAACCTAGATCTTTCAAGACCATTTCTAGTGCTCTGAACCATGCTGG
GCTCAAGCTAGCGTCATAACCGGTGTAGTCAAAAGCAAACAAACTGCCTGGAAGAAGGATGGGGATCTTA
CTCCAGAAGGTATCAGGGTTTGCTCCTACTGCAGAACCAGTAATGGTGCCTGGATTCTCATGGAATTTTT
CAAACAGGTGTCCAAAGCACATCCTCATGTATACTGAATCATTGAGACTTGATGCTTCAATCAGTCTGGA
TTTTCCTTTCTTCACTTTCTCCCTGCTTCTTAACTCATCCTTGATATATGTGGAGAAGGGCAGGTCTAAT
CCATACTTATCCATGTAGTACTTCATTTTAGTGACATCTCTAGTGGTAGGGTCCAAAATCTGCCTTTTCT
TGATGCCTAAAGCATTGTAGGGGTATCCTGCACTGGTGCTAAGGTCAATTGCTTCCAAGTTCTCTGTTCC
ATAGCAGGCATCTTCCATACTCATCCTACTAGTATCGATGTTCAATTGCTTTAGTTGGTTTGAGTAATGT
TTAGCTGCTTGAATGATATACTCATCTGGTTCATGAATAGCATTGCCAATGTATTTGCTGAACAGGGCTT
CCTCAAAGTCAACTTCCAACCTTGGGTCCTTTGCACTAAGAGCTGCAGGCTCCTTCTTACCCTCAAACAC
ATCATGAAACACACTGGGTTCAAGTTTGGTCTGGGTTGGACCATTAATGTTGTAGCGGCCAGTTTCCTTG
TTGGGTTTCATCCACTGGATCTCACCTTGCTCGGTGGCAAAGTAACTCCTCTTCAGTGCCGCACAGAACC
CTTGCTTTCCATTGCCTCCAACATGAATTCCAATCACTTTTCCTGTACTAGTAACCACTCCACCACACTG
GCCGGCTTTGGTTGGGAAGTTGTACATCATTGTTCTGTGTGTTGGTTTTCCACTCAGATTCAGGAAGCCA
TAAAACTGGACATCACCCACTGGAACAAACATTGATGGCATCTGCTCTGTGTTGATGATGAGTGTAGCAT
CCATACAGTGTTCAAATGATTCAGGGATGAATTTAGTAATGTCTCTAAATTTCTCATTCATGTCCAATGT
GACTAAGGTTAATTCCAAGTTTACTTGCTGCTCATCAACTAGTTCAACCGCATCTAGGACTGTCACTTGT
TTTCCTTCTAGCCAGATGGTCTTGCCAGGTTTTGCATGCCTTGGGAGCACTGCAAGCCTGTCTCTAACAC
CAAGCATGGTAAAGTGTCCCTCTTCAGTTTGCACTTTTCTGATGTTCTTTTTAAGTAGTGAGATGGCAAA
GTCTAAGCCTGGCCCTTGTGCTACYGCCGTTCTYAAGACTGGTTTCTTCACTGCTGTTTTGGGCATTCCA
GAGTAGGCACCCTGAAATCCAGCAAACAACCTATAGATGACATACACCATAGATATTACAGCCAGAATGG
TGGTGATAGACTGGATCAATGCAATTGCCCTGTTGTATTGTCTTTCAAGATTGGTTGGCACATCAGGGAT
TATCCATCCCTTCTCCTTGCARTAYTCTCTCACTTCTTCATTGTCAACACTTCTCAAGAGGTCGGCAATA
GCGTCTGGGGCTGGAGTGTCCAAAGAAATGGTGAGAGGTTTAAACTTTGGTGGGCCTTGGAATAATGCCT
CAATTGTATTTCCAACTGACATTCTATTGTTGTATTCTCTAATCAATTCTGAGACCACTGAATCCAAACT
GTATCTAACTTTTGACTTTCTGTCTTTTAACTGAATAGCCTTACCACAGACCAGTGGGCTGCATCTTTTG
AAGTTGGCGGTGTTGTTTTCTGAGCACAACTTTGCTGCTCTGCCTGCATCCAATTTACCACTTTCCAATC
TAAAGCTGTCTGGGACTTCAATGTCACAATCCATGTAGAACCTCCTCCTAATTGCTTCTGAGTCAGAAAC
GGTGGGCACAATGATGTTACCAGCATTGGTGGAAGCGATGACGAATTTTGAGTTGAATGCAATACCTTTC
TCTTCTAGTGAGGCCATTGGTGGTATGAACTCAACTGTGGAAACCATTTGACAAAAGAGGGAGATGTCCT
TTCCATCAGGRTTTTGGCACAAATCATCCATAACTGTGACTACTTGCTGCTTGTACCCATCAAAATGATC
TGGATCTGGTGGTAATGAGTACACGCTGGAGTTGAATTTCTCAGCGATGGCTCTGGCAATTATGCTGGTT
GCCAGGGATTTCCCTGGCCCAGGATATCCTCNNNNNNNNNNNNNNNNNNNNNNNNNNNNNNNNNNNNNNN
NNNNNNNNNNNNNNNNNNNNNNNNNNNNNNNNNNNNNNNNNNNNNNNNNNNNNNNNNNNNNNNNNNNNNN
NNNNNNNNNNNNNNNNNNNNNNNNNNNNNNNNNNGAAAAGGTAGAGTTCCTTAACAATCTCAAACAACTG
CCCTTGTTGGAGAACCAGGTTAGCAACYTRGAACAGGCTGCTGCAAGTCAGGAGAGTTTAGAACAACTGT
TTGGCAATGTTCAATATTTGGCAATCAATTGTAGGAAATTCCAACCACTCTACGCAGCTGAGGCTAAGAG
AGTCTTCTCAATGGAGAAGAGAATGGTCAACTACATGCAGTTCAAGGGCAAACAACGTATTGAACCTGTA
TGCCTGATCATTAGAGGCTCTCCTGGGACAGGGAAATCCCTGGCAACCAGCATAATTGCCAGAGCCATCG
CTGAGAAATTCAACTCCAGCGTGTACTCATTACCACCAGACCCAGACCATTTCGATGGGTACAAGCAGCA
AGTAGTCACAGTTATGGATGATTTGTGCCAAAATCCTGATGGAAAGGACATCTCCCTCTTTTGTCAAATG
GTTTCCACAGTTGAGTTCATACCACCAATGGCCTCACTAGAAGAGAAAGGTATTGCATTCAACTCAAAAT
TCGTCATCGCTTCCACCAATGCTGGTAACATCATTGTGCCCACCGTTTCTGACTCAGAAGCAATTAGGAG
GAGGTTCTACATGGATTGTGACATTGAAGTCCCAGACAGCTTTAGATTGGAAAGTGGTAAATTGGATGCA
GGCAGAGCAGCAAAGTTGTGCTCAGAAAACAACACCGCCAACTTCAAAAGATGCAGCCCACTGGTCTGTG
GTAAGGCTATTCAGTTAAAAGACAGAAAGTCAAAAGTTAGATACAGTTTGGATTCAGTGGTCTCAGAATT

GATTAGAGAATACAACAATAGAATGTCAGTTGGAAATACAATTGAGGCATTATTCCAAGGCCCACCAAAG
TTTAAACCTCTCACCATTTCTTTGGACACTCCAGCCCCAGACGCTATTGCCGACCTCTTGAGAAGTGTWG
ACAATGARGAAGTKAGAGARTAYTGCAAGGARAARGGRTGGATMATCCCYGAKGTRCCAACAAATYTWGA
RAGACAATACAATAAAGCAATTGCACTAATCCAGTCCATCACTACCATTCTGGCTGTTGTATCTATGGTG
TATGTCATCTACAGGCTGTTCGCTGGATTCCAAGGTGCTTACTCCGGAATGCCCAAGACGGCAGTGAAGA
AACCAGTCTTAAGAACAGCAGTAGCACAAGGGCCAGGCTTAGACTTTGCCATCTCACTACTTAAAAAGAA
CATCAGAAAAGTGCAAACTGAAGAGGGACACTTTACCATGCTTGGTGTTAGAGACAGGCTTGCAGTGCTC
CCAAGGCATGCAAAACCTGGCAAGACCATCTGGCTAGAAGGAAAACAAGTGACAGTCCTAGATGCGGTTG
AACTAGTTGATGAGCAGCAAGTAAACTTGGAATTAACCTTAGTCACATTGGACATGAATGAGAAATTTAG
AGACATTACTAAATTCATCCCTGAATCATTTGAACACTGTATGGATGCTACACTCATCATCAACACAGAG
CAGATGCCATCAATGTTTGTTCCAGTGGGTGATGTCCAGTTTTATGGCTTCCTGAATCTGAGTGGAAAAC
CAACACACAGAACAATGATGTACAACTTCCCAACCAAAGCCGGCCAGTGTGGTGGAGTGGTTACTAGTAC
AGGAAAAGTGATTGGAATTCATGTTGGAGGTAATGGAAAGCAAGGGTTCTGTGCGGCACTGAAGAGGAGT
TACTTTGCCACCGAGCAAGGTGAGATCCAGTGGATGAAACCGAACAAGGAAACTGGCCGCTACAACATTA
ATGGTCCAACCCAGACCAAACTTGAACCCAGTGTGTTTCATGATGTGTTTGAGGGTAAGAAGGAGCCTGC
AGCTCTTAGTGCAAAGGACCCAAGGTTGGAAGTTGACTTTGAGGAAGCCCTGTTCAGCAAATACATTGGC
AATGCTATTCATGAACCAGATGAGTATATCATTCAAGCAGCTAAACATTACTCAAACCAACTAAAGCAAT
TGAACATTGATACCAGTAGGATGAGTATGGAAGATGCTTGCTATGGAACAGAGAACTTGGAAGCAATTGA
CCTTAGCACCAGTGCAGGATACCCCTACAATGCTTTAGGCATCAAGAAAAGGCAGATTTTGGACCCTACC
ACTAGAGATGTCACTAAAATGAAGTACTACATGGATAAGTATGGATTAGACCNNNNNNNNNNNNNNNNNN
NNNNNNNNNNNNNNNNNNNNNNNNNNNNNNNNNNNNNNNNNNNNNNNNNNNNNNNNNNNNNNNNNNNNNN
NNNNNNNNNNNNNNNNNNNNNNNNNNNNNNNNNNNNNNNNNNNNNNNNNNNNNNNNCATTCAACTCAAAAT
TTGTCATTGCATCCACTAATGCTGGCAACATAATTGTGCCAACTGTGTCTGATTCAGAGGCCATCAGGAG
AAGATTCTACATGGATTGTGATATTGAAGTCCCAGACAGTTTCAGGTTAGATAGTGGTAAGTTAGATGCA
GGCAGAGCAGCAAAATTGTGTTCAGAGAACAACACTGCCAATTTCAAGAGATGTAGCCCACTGGTCTGTG
GTAAAGCTATTCAGTTAAGAGACAGAAAATCCAAGGTGAGATACAGTCTGGATTCAGTTGTCTCGGAACT
TATCAGAGAGTACAACAACAGAATGTCTGTTGGAAACACCATTGAGGCACTGTTCCAAGGTCCACCAAAA
TTCAAGCCCCTCACAATCTCTTTAGACACACCAGCCCCAGATGCTATTGCAGATCTTCTGAGAAGTGTAG
ACAACGAGGAAATCAGAGAGTATTGCAAGGAGAAGGGGTGGATAATCCCTGATGTACCAACCAATCTTGA
AAGACACTACAACAGGGCAATCGCACTGATCCAGTCTATCACTACCATTCTGGCTGTAGTATCTATGGTG
TATGTCATCTATAGGTTGTTTGCTGGATTTCAGGGTGCTTACTCTGGAATGCCCAAAACAGCAGTGAAGA
AACCAGTCTTGAGAACAGCAGTAGCACAGGGTCCAGGCTTAGACTTTGCCATCTCACTCCTCAAAAAGAA
CATCAGAAAGGTGCAGACTGAAGAGGGACACTTCACTATGCTTGGTGTTAGAGACAGACTTGCAGTGCTA
CCAAGACATGCAAAACCAGGCAAGACCATCTGGATGGAAGGCAAACAGGTCACTGTATTAGATGCAGTTG
AACTAGTTGATGAACAGCAGGTGAATTTGGAATTGACCCTGGTAACATTAGACATGAATGAGAAATTCAG
AGACATCACTAAATTTATTCCTGAGTCTTTTGAACACTGTATGGATGCCACCCTGATTATCAATACGGAG
CAGATGCCATCAATGTTTGTCCCAGTAGGAGATGTTCAGTTTTATGGCTTCCTGAATTTGAGTGGAAAAC
CAACGCACAGGACAATGATGTACAATTTCCCAACCAAAGCTGGTCAGTGTGGTGGAGTGGTTACTAGTAC
AGGGAAAGTGATTGGAATTCATGTTGGAGGTAATGGAAAGCAAGGGTTCTGTGCGGCATTGAAGAGAAGC
TACTTTGCTACAGAGCAAGGTGAGATCCAATGGATGAAATCTAACAAAGAGACTGGCCGCTACAATATCA
ATGGTCCAACCCAGACCAAACTTGAACCCAGCGTGTTTCATGATGTGTTTGAGGGTAAGAAGGAGCCTGC
AGCACTTAGTGCAAAGGACCCAAGGTTGGAAGTTGATTTTGAGGAAGCCCTGTTCAGTAAATATATTGGC
AATGCTATCCATGAACCAGATGAGTACATCATCCAAGCAGCCAAACATTACTCAAACCAACTAAAGCAAC
TGAACATTGACACCAGTAGGATGAGCATGGAAGATGCCTGCTATGGGACTGAGAACTTAGAAGCAATTGA
CCTTAGTACCAGTGCAGGATACCCCTACAATGCTTTGGGCATCAAGAAGAGGCAGATTTTGGACCCTACC
ACTAGAGATGTCACCAAGATGAAATATTACATGGATAAATATGGATTGGATCTACCCTTTTCTACATACA
TCAAGGATGAACTGAGGAGCAGAGAAAAAGTGAAGAAGGGGAAGTCTAGACTGATTGAGGCATCAAGTCT
CAATGATTCAGTGTATATGAGAATGTGTTTTGGACACCTGTTTGAAAAGTTCCACGAGAATCCAGGCACC
ATCACAGGCTCTGCAGTAGGAGCAAACCCTGACACCTTCTGGAGCAAGATCCCCATCCTTCTCCCAGGCA
GCTTGTTTGCTTTTGATTACACCGGCTATGACGCTAGCTTAAGTCCAGCATGGTTCAGAGCACTAGAAAT

AGTTTTGAAAGATCTAGGCTATGATGATGAGGCTATTAGCCTCATCGAGGGCATAAATCATTCTCACCAC
ATCTATCGCAACAAAACATACTGCGTGGTTGGTGGCATGCCCTCAGGCTGCTCTGGAACCAGTATCTTCA
ATAGCATGATCAACAACATTATCATCAGGACCTTGCTGATTAGAACATTCAAGGGCATTGATTTGGATGA
ATTGAACATGATTGCCTATGGAGATGATGTGTTAGCTAGTTACCCATTCCCAATTGATTGTGCAGAACTT
GCAAGGACTGGTAGGGAATATGGTTTGGTTATGACACCTGCAGACAAATCTTCTTGCTTTAATGAGGTCA
CTTGGGAAAATGCAACATTCTTGAAAAGAAGTTTCAAACCAGATGAACAATTTCCATTCTTGATCCACCC
TGTCATGCCCATGTCAGAGATCCACGAGTCCATTCGCTGGACTAAGGATGCGCACAACACTATCCTCCNN
NNNNNNNNNNNNNNNNNNNNNNNNNNNNNNNNNNNNNNNNNNNNNNNNNNNNNNNNNNNNNNNNNNNNNN
NNNNNNNNNNNNNNNNNNNNNNNNNNNNNNNNNNNNNNNNNNNNNNNNNNNNNNNNNNNNNNNNNNNNNN
NTGTATGCCTGATCATTAGAGGCTCTCCTGGAACAGGGAAATCCCTAGCAACCAGCATAATCGCCAGAGC
CATTGCTGAGAAATTCAACTCCAGCGTGTATTCATTACCACCAGACCCAGACCACTTTGATGGGTACAAG
CAGCAAGTAGTCACAGTTATGGATGATTTGTGCCAAAATCCTGATGGAAAGGACATCTCCCTCTTTTGTC
AAATGGTTTCCACAGTTGAGTTCATACCACCAATGGCCTCACTAGAAGAAAAAGGTATTGCATTTAACTC
AAAATTTGTCATCGCTTCCACCAATGCTGGTAACATCATTGTGCCCACTGTCTCTGACTCAGAGGCAATC
AGGAGGAGGTTCTACATGGATTGTGACATTGAAGTCCCAGACAGCTTTAGATTGGAAAGTGGTAAATTGG
ATGCAGGCAGAGCAGCAAAGTTGTGCTCAGAAAACAACACCGCCAACTTCAAAAGATGCAGCCCACTGGT
CTGTGGTAAGGCTATTCAGTTAAGAGACAGAAAATCAAAAGTCAGATACAGTTTGGATTCAGTGGTCTCA
GAATTGATTAGAGAATACAACAATAGAATGTCAGTTGGAAATACAATTGAGGCATTATTCCAAGGCCCAC
CAAAGTTTAAACCTCTCACTATTTCTTTGGACACTCCAGCCCCAGACGCTATTGCTGACCTCTTGAGAAG
TGTTGACAATGAAGAAGTGAGAGAATACTGCAAGGAAAAAGGGTGGATCATCCCCNNNNNNNNNNNNNNN
NNNNNNNNNNNNNNNNNNNNNNNNNNNNNNNNNNNNNNNNNNNNNNNNNNNNNNNNNNNNNNNNNNNNNN
NNNNNNNNNNNNNNNNNNNNNNNNNNNNNNNNNNNNNNNNNNNNNNNNNNNNNNNNNNNCTACGCAGTATG
TTTTGTTGCGGTAGAAGTGGTGGGAGTGGTTAATACCCTCAATGAGGCTGACAGCCTCATCGTCATAACC
TAGATCTTTCAAGACCATTTCTAGTGCTCTGAACCATGCTGGGCTCAAGCTAGCGTCATAACCGGTGTAG
TCAAAAGCAAACAAACTGCCTGGAAGAAGGATGGGGATCTTACTCCAGAAGGTATCAGGGTTTGCTCCTA
CTGCAGAACCAGTAATGGTGCCTGGATTCTCATGGAATTTTTCAAACAGGTGTCCAAAGCACATCCTCAT
GTATACTGAATCATTGAGACTTGATGCTTCAATCAGTCTGGATTTTCCTTTCTTCACTTTCTCCCTGCTT
CTTAACTCATCCTTGATATATGTGGAGAAGGGCAGGTCTAATCCATACTTATCCATGTAGTACTTCATTT
TAGTGACATCTCTAGTGGTAGGGTCCAAAATCTGCCTTTTCTTGATGCCTAAAGCATTGTAGGGGTATCC
TGCACTGGTGCTAAGGTCAATTGCTTCCAAGTTCTCTGTTCCATAGCAGGCATCTTCCATACTCATCCTA
CTAGTATCGATGTTCAATTGCTTTAGTTGGTTTGAGTAATGTTTAGCTGCTTGAATGATATACTCATCTG
GTTCATGAATAGCATTGCCAATGTATTTGCTGAACAGGGCTTCCTCAAAGTCAACTTCCAACCTTGGGTC
CTTTGCACTAAGAGCTGCAGGCTCCTTCTTACCCTCAAACACATCATGAAACACACTGGGTTCAAGTTTG
GTCTGGGTTGGACCATTAATGTTGTAGCGGCCAGTTTCCTTGTTGGGTTTCATCCACTGGATCTCACCTT
GCTCGGTGGCAAAGTAACTCCTCTTCAGTGCCGCACAGAACCCTTGCTTTCCATTGCCTCCAACATGAAT
TCCAATCACTTTTCCTGTACTAGTAACCACTCCACCACACTGGCCGGCTTTGGTTGGGAAGTTGTACATC
ATTGTTCTGTGTGTTGGTTTTCCACTCAGATTCAGGAAGCCATAAAACTGGACATCACCCACTGGAACAA
ACATTGATGGCATCTGCTCTGTGTTGATGATGAGTGTAGCATCCATACAGTGTTCAAATGATTCAGGGAT
GAATTTAGTAATGTCTCTAAATTTCTCATTCATGTCCAATGTGACTAAGGTTAATTCCAAGTTTACTTGC
TGCTCATCAACTAGTTCAACCGCATCTAGGACTGTCACTTGTTTTCCTTCTAGCCAGATGGTCTTGCCAG
GTTTTGCATGCCTTGGGAGCACTGCAAGCCTGTCTCTAACACCAAGCATGGTAAAGTGTCCCTCTTCAGT
TTGCACTTTTCTGATGTTCTTTTTAAGTAGTGAGATGGCAAAGTCTAAGCCTGGCCCTTGTGCTACYGCC
GTTCTYAAGACTGGTTTCTTCACTGCTGTTTTGGGCATTCCAGAGTAGGCACCCTGAAATCCAGCAAACA
ACCTATAGATGACATACACCATAGATATTACAGCCAGAATGGTGGTGATAGACTGGATCAATGCAATTGC
CCTGTTGTATTGTCTTTCAAGATTGGTTGGCACATCAGGGATTATCCATCCCTTCTCCTTGCARTAYTCT
CTCACTTCTTCATTGTCAACACTTCTCAAGAGGTCGGCAATAGCGTCTGGGGCTGGAGTGTCCAAAGAAA
TGGTGAGAGGTTTAAACTTTGGTGGGCCTTGGAATAATGCCTCAATTGTATTTCCAACTGACATTCTATT
GTTGTATTCTCTAATCAATTCTGAGACCACTGAATCCAAACTGTATCTAACTTTTGACTTTCTGTCTTTT
AACTGAATAGCCTTACCACAGACCAGTGGGCTGCATCTTTTGAAGTTGGCGGTGTTGTTTTCTGAGCACA
ACTTTGCTGCTCTGCCTGCATCCAATTTACCACTTTCCAATCTAAAGCTGTCTGGGACTTCAATGTCACA

ATCCATGTAGAACCTCCTCCTAATTGCTTCTGAGTCAGAAACGGTGGGCACAATGATGTTACCAGCATTG
GTGGAAGCGATGACGAATTTTGAGTTGAATGCAATACCTTTCTCTTCTAGTGAGGCCATTGGTGGTATGA
ACTCAACTGTGGAAACCATTTGACAAAAGAGGGAGATGTCCTTTCCATCAGGRTTTTGGCACAAATCATC
CATAACTGTGACTACTTGCTGCTTGTACCCATCAAAATGATCTGGATCTGGTGGTAATGAGTACACGCTG
GAGTTGAATTTCTCAGCGATGGCTCTGGCAATTATGCTGGTTGCCAGGGATTTCCCTGGCCCAGGATATC
CTC

>Enterovirus A92
CCAACAGACATTCTGTTGTTATACTCTCTGATGAGTTCCGAAATCACTGAGTCTAGACTGTATCTGACCT
TGGACTTTCTGTCTCTCAATTGGATGGCCTTACCACAAACCAACGGGCTGCACCTCTTAAAGTTAGCAGT
GTTGTTTTCTGAACACAATTTTGCTGCTCTACCTGCGTCAAGCTTTCCACTCTCTAGTCTGTAGCTGTCT
GGAACTTCAATATCACAATCCATGTAGAATCTTCTCCTGATAGCTTCAGAATCAGATACAGTTGGTACAA
TGATGTTGCCTGAGTTAGTGGATGCAATAACAAATTTTGAATTGAATGCGATGCCCTTTTCCTCCAGTGA
GGCCATTGGCGGAATAAACTCAACTGTAGAAACCATCTGGCAAAATAGAGAGATATCTTTTCCATCAGGG
TTTTGACACAAGTCATCCATAACTGTCACCACTTGCTGTTTGTACCCATCAAAATGATCTGGGTCTGGTG
GCAGTGAGTATACACTGGAGTTGAATTTCTCAGCAATGGCTCTGGCAATTATGCTGGTTGCTAAGGATTT
TCCTGTACCTGGGGATCCTCTGATAATTAAACATACAGGTTCAATACGTTGTTTGCCCTTGAACTGCATG
TAGTTTACCATCCTTTTCTCCATGGAAAACACTCTTTTGGCCTCAGCTGCGTAAAGTGGTTGAAACTTTC
TACAATTTATTGCCAAGTATTGGACATTACCAAATAGCTGCTCTAGGCTCTCCTGACTTGCAGCAGCTTG
CTCCAGGTTATTAATCTGGTTTTCCAACAAGGGAAGTTGCTTAAGGTTGTTGATGAACTCAGCTTTCTCT
CTTGCAATGGGTAGGATTTTCTCTCTGATCCAATCTATGAATTTGCTAATTTTGTTAGCGATCCACTCAA
GACCACGGGCTGCGTTTGCCATGTCGTTGAACTTTTTCAGCCAAGAGTCAGATTGTTTTTGAGCAATAGG
GATGCCTAAGATGGATGCAACTTTTGTCTTAAGCCACCTCCATGGGCTACCTGTACACCCAATTAATGCA
AGTGTTGCTGTAACTGTGACTAAGTCATACTCACTTCTTACAATGATGACCAGTGCTGAGATCAATTTCA
CCAAGTTCTTAAGAATCTTTTCAACTATTCCTTCCGACCCAACAAGTGAGTCCTTGATGTTTTGTACTTC
CCTAGCAATGGACTCTGTAAAACCACTTCCGAAAGCGTCACCCAAGCCCTTGATGTAATCGGTGACACCT
TGTTCCATCACCTCATCCTCTAGCCAAAGGAGGTCTCTTAAGTCAGCGAAACCTACTAGCCCATCTCCAC
CCATGGTGACTAAGCCAATCACACCATGGGGGCACCTTAGGATGCCACCACAATCACCTGCTACAGCGAT
CCCATTTGCCAATAGTACATGTGATTGGTATCTTTCAGGGTAGTATTCGTTTGCTTGAACATGCACAAAA
CCAGGGCCCTGGAAGGAAACTGGGTAATGTTTGTTTGCTGATTTGCAGAAGTACACTCCTGTGTTGCAAT
TACACCTGGCGATCTTGTCACACCCATGTGCACTAGTTCTAGAAACAAGAATATCTCTTTGGTAATCCTC
CCACTCAAGATTATCCCAGTCATTCTTAGTAGCCAGGTGTCTGTTAACAATTCTGTAGTTGCATACATAC
ACGGCACCACTTTGCTGACCAAAGGCTCCAGTTGTGGTTATGCTCGCTCTGTCTCTAGCCATGTGTCTGA
TTTCATTGCCATTGAAATTTGGATAGTTCTTCAATACGTATGGCTGGCTTCTCATTGGTCTGGGGACCCA
TGCCTTAACATGTTTAATTCTCATGTAAATTCTAACAGTTAATTTAGTTGTGACATTCTCGGATCCCACT
ACTCTGAAAGCAAAGGTACCAAAAAGATTGTTAGGGCTAATTCCATAGTTTACATCGCCAGATGCAGGGT
GTTCACCAAACGTAGGGTACCCATCATAAAACCACTGGTAAGCTGAGGCCGGTGACATGAAGGGGACAGA
AACCWGAGCTGGGGGGTCAGATGCTTTGACAAACACAGAGGGGTTAGTGGCAGACTGCCATTCAAAAGAG
TCCCTTGCAGTTGGTGCAGGGGCACCTGGAGGAATGTACATGTATTGAACTACCACATTAGGTCCAGTGC
CAGTGGAGTCAGTGCAGACAAAAGTGAATTCAGAGTCAAACCTCATGTAAGTAAAGATTTCCAATTTTCT
ACGCTGCTGCACGTACCCCATTATGTCAATAGGCCAGTTAGTGAAACCATTTGTTGTGCTTTCTGATGTG
AGCAAGGTGGCCATTCCGACAAGAGCTGCTCTGCCAAAGAAATTCTCCAGACTGGTTTCGGCAACACTAT
GGTGGTTGTGCACACACCTCGTTTCTATCATGGACTCATCAGAGGTGACTGATGTTGCACCCGTCTCAGC
AGCTTGCAACGCTGGTGCAGATCCTGTTAAGAGACTGTGTGAGCTCGCACTAGTGTCAGCACCGGTACTA
GTGGAGGCGCCCTCGGTGTTGGCAAGAACTTTGTGCGTTGTAGCATTGATGTGAGTTTCTAGCATGTCCT
TGATATCACCTTGCAGCACTGCAGTTTGGGAGATCTCGTCGGTGTCTTTGCACAACCGCATAGTGAAGTT
TGGCTGGGCAGCACCCAGAGCTATAAGGTATGCACTAGTTGGAGCACCTGCTGGAACAACAAAGTTTGTT
TGGTACCAAATGGTGACGATACCTGCAGTGTAATAATCAAAGTAGGTTCCTGATGCATTCGTCCTAAAAT
GTGTGTTGCTAATCCAAGGAATCACCAATGTTATGGATGACTGAAGACCAAAATCCCAAATGACGTGGGT
ACCAAGCATTGCCACAGATCTACTTGTGGGCTGCGCTCCACCGGGAGGTGTGTAGGCTATCAAGAGTTTT

CCAGTGGCCATAAAGCTGCCTGTAAACATGAAGGTGACTTCCAATGAACCTGACCACTGGGTATAGTATC
TGCACAGTTGACCCACCATAGTGGACTGCCATGGGCCATCCCGACCTGGATCCACTCTGAATGAAGCACA
TAACTGATCAACCTCACTCTGAACAGACACTGGTATCAAGAGTCTATCCATTTGAGATACATTGTTCACA
TTGTTCACCTCAAGTATGCTTTCAATCTGGCATAGTTGTAACAAATTGTTCACCTCTCCTGGGATGAGGA
TCAAGGGTGTTGGGTGGAAGCCAGGGAGTATAGGTGCCGCAACACCATCATCTGTAGTAAGGAACTGGTT
ACTGCCAGGCTTCAACTCCACTGGCAAACCCTGTGTGATAGCGTTCCTTAGGCCAGCAAACTCAGCACAA
AGGGGTGCGATGGTGACTGTTATGGGCACMGCCGTTGTGGCTCCATCACTGTACGCTAGTGGGGCAACAG
GAACTACAAACAGGCCAAAGTTGCAGTGATTTAAGGCTGAGTCGTAAGGAACTGCATTCACATAAGGCAT
AATAATGGTAGCACAATTGTTTGTCCTGAGATTGATCCACTGGTGAGGAGCGACAAGTAGCTGGGAGAAA
GCTACTCCAGAATCCAGTACGTAGGGAAAGGCCAACTCTTTGCCGTTAGGTCCAGGTTGTGTAGCATTGT
ACGATGGGTGCTTTGCAGAGCTGGGTGTGTTAGTTGCATCACTTCTCCCATTGGTGTATTCAGGTAAGCA
TGCCACCAGGAGGGTTCCCTGGTGAAACTTACTCGCATTGCATTGTACATGGATAGCAAAGCCTGATCTG
TATAAATAGTGAAACTGTGCATTCTGGCCAAAAACACCTGTCTCAGTCAGGACATCAGGAAATTTCCAGT
ACCATCCTTTGCTGTTTGATTGCCACAATTTTGCTGATAGTGTGTAAAATCTGTTAACCGAAACATCTGG
TCTAGTTGGCTTGTCAACAGCAGTAGCATCTGAGTCACTACAGTACTCGGGCCACTCGCCGTACCCTACA
ATCACATTGGCAGCCTCTTGTGTGGTAATGGTGGAATTCCCAACTGTCATTTGGCAGACCCTGTCACTGT
AACCACATGCCTCTGCTGAAGGAGATTTGAGTGGTACTGATGATTCTTTCAGGACATCAACTACTGGTTG
GGTGAACTTGCTTGGATCCTGACTGAAGTCTTGTTTGTTAGCTGAAGCAGCGTAGGAATCCTTGTAATAG
TTTATGGTGGTGTAGTGAATGGTGGATCCACCTGTGGCCACGTTGGTATTCTCGTGGGTTCCAGATTGTT
GGGTGGAAACTTGAGCTCCCATGATGAAACTATGAAGTATTGCAAATGCTGAAGAGCAAAGAGTAATTCA
CACAAAAATATATCCGGATGGCCAATCCAATCGCTATATGATGACAACTCTAGATTGTCACCATAAGCAG
CCAAGGTAAATAAAACAGGAAACACGGACACCCAAAGTAGTCGGTTCCGCCACAGAATTACTCGTTACGA
CCATAATACCACTGGTTTGTGGCACATGGCTCCGTGGTTGGGATTAGCCGCATTCAGGGGCCGGAGGACT
ACTACCTAGCTCAATAGGCTCTTCGCACCATGTCATGAATCAAGCGTCCTGCGAGTGGAACTCGCAGGCA
GGCCGCCAACGCAGCCACCGACACGGTCGCCCGTGGGGAATTCGGTGACTCATCGACCTGAACTACACTG
GGGATGTGCTGAACGTAACACTTGAGACATTCAATGATGGTACTAGGCTTCTCGAAGTAGGTAGCCGGGT
AACGAACGTTTTCCCCTTCAGCCGTGGGTACAGCCTATTGGTACTAGGTCCGGGGAAACAGAAGTGCTTG
ACGAAAATCCACCTGGTTTGGTGGATTTGCTATTTGTCATTTGTGCTTTCTTCTAACNNNNNNNNNNNNN
NNNNNNNNNNNNNNNNNNNNNNNNNNNNNNNNNNNNNNNNNNNNNNNNNNNNNNNNNNNNNNNNNNNNNN
NNNNNNNNNNNNNNNNNNNNNNNNNNNNNNNNNNNNNNNNNNNNNNNNNNNNNNNNNNNNNNNCAAGGTA
GVGGATAAGCGGCGTTAGTACTCTGGTATTACGGTACCTTTGTATGCCTGTTTTATCTCCCTTCCCCCGT
AACGTTAGAAGAATGCACCAATGACAAATAGCAAGTCCACCAAACCAGGTGGATTTTCGTCAAGCACTTC
TGTTTCCCCGGACTTAGTACCAATAAGCTGTACCCACGGCTGAAAGGGAAAACGTTCGTTATCCGGCTAC
CTACTTCGAGAAGCCTAGTACCATCATTGAATGTCTCAAGTGTTACGCTCAGCACATCCCCAGTGTAGTT
CAGGCCGATGAGTCACCGAATTCCCCACGGGCGACCGTGTCGGTGGCTGCGTTGGCGGCCTGCCTGCGAG
TTCCACTCGCAGGACGCTTGATTCATGACATGGTGCGAAGAGCCTATTGAGCTAGGTAGTAGTCCTCCGG
CCCCTGAATGCGGCTAATCCCAACCACGGAGCCATGTGCCACAAACCAGTGGCATTATGGTCGTAACGAG
TAATTCTGTGGCGGAACCGACTACTTTGGGTGTCCGTGTTTCCTGTTTTATTTACCTCGGCTGCTTATGG
TGACAATCTAGAGTTGTCATCATATAGCGATTGGATTGGCCATCCGGATATATTTTGTGTGAATCACTTT
TTGCTCTTTAGCAATTACAATACTACATAGTTTCATCATGGGAGCTCAAGTGTCTACCCAACAATCTGGA
ACCCATGAAAACACCAATGTGGCTACAGGTGGGTCCACTATTCACTACACCACCATAAACTACTACAAGG
ATTCTTATGCTGCTTCAGCCAACAAGCAAGACTTTAGTCAAGATCCAAGTAAGTTTACTCAGCCAGTGGT
TGATGTTCTCAAGGAATCATCAGTACCACTCAAATCTCCTTCAGCAGAGGCGTGTGGTTACAGTGACAGA
GTCTGCCAGATGACAGTTGGAAATTCCACCATTACCACACAAGAGGCTGCTAATGTAATTGTAGGATACG
GTGAGTGGCCCGAATACTGTAGTGATTCAGACGCTACTGCTGTTGACAAGCCAACTAGGCCAGATGTTTC
GGTCAATAGATTTTACACATTATCAGCAAAATTGTGGCAATCAAATAGCAAAGGGTGGTACTGGAAGTTT
CCTGATGTCTTGACCGAGACAGGTGTTTTTGGTCAGAATGCACAGTTTCATTATTTGTACAGATCAGGTT
TCGCCATCCATGTACAATGCAATGCAAGCAAATTTCACCAAGGAACCCTCCTGGTGGCATGCTTACCTGA
ATACACCAATGGAAGGAGTGATGCAACCAACACACCTGGCGATGCAAAACACCCATCGTACGTTGCCACG
CAACCTGGACCTGCAGGCAAAGATTTGGCCTTTCCATATGTATTGGATTCTGGGGTAGCTTTCTCCCAAC

TACTTGTTGCCCCCCACCAGTGGATCAATCTAAGGACAAACAATTGTGCTACCATAATCATGCCTTACGT
GAATGCAGTACCTTATGATTCAGCTTTAAACCACTGTAACTTTGGCTTGTTTGTTATACCTGTTGCCCCA
CTAGCGTACAGTGATGGAGCCACAACAGCTGTGCCCGTAACAGTCACCATTGCACCACTTTGTGCTGAGT
TTGCTGGTTTGAGGAATGCTGTCGCTCAGGGCCTGCCAGTGGAGTTGAAACCAGGCAGTAACCAGTTTCT
TACTACAGATGACGGTGTTGCAGCACCCATATTACCTGGCTTCCACCCAACACCCCTAATTCACATTCCA
GGAGAAGTGAACAACTTGTTGCAATTGTGTCAAATTGAAAGCATACTTGAGGTGAACAATGTGAGCAGTG
CATCTCAGATGCAAAGACTCCTGATACCAGTATCAGTCCAGAGTGAGGTTGATCAATTATGTGCCTCGTT
CAGAGTAGATCCAGGTCGAGATGGCCCTTGGCAGTCCACTATGGTAGGTCAGTTATGCAGGTATTACACC
CAGTGGTCAGGCTCATTGGAAGTCACCTTCATGTTCACGGGCAGTTTTATGGCCACCGGAAAGCTCTTGA
TAGCCTACACACCTCCTGGTGGATCGCAACCCGCAAGCAGATCCATAGCAATGCTTGGTACCCATGTCAT
CTGGGACTTTGGCCTTCAGTCATCTGTAACGCTAGTGATCCCCTGGATTAGCAACACGCACTTTAGGACA
AACGCATCGGGAACATACTTTGATTACTATACTGCAGGTATTGTCACCATTTGGTATCAAACAAACTTTG
TCGTCCCAGCAGGTGCTCCAACTAGTGCATACCTTATAGCTCTGGGCGCTGCCCAGCCAAACTTCACCAT
GCGGCTGTGCAAAGACACTGACGAAATCTCCCAAAACGCAGTGTTGCAAGGTGACATCAAGGATATGTTG
GAGACTCACATTAATGCCACAACACACAAAGTTCTCGCCAGCACTGAGGGCGCCTCCACCAGCACCGGCG
CTGACACTAGTGCGAGCTCACACAGCCTTTTGACAGGGTCTGCACCGGCGCTGCAGGCAGCTGAGACGGG
CGCGACATCAGTTACCTCTGACGAGTCCATGATAGAGACAAGGTGTGTTCACAATCACCACAGTGTTGCT
GAAACTAGTTTAGAAAACTTCTTTGGCAGGGCGGCTCTTGTCGGAATGGCCACTCTACTCACATCAGAAA
CCTCAACAAATGGGTTTACCAACTGGCCAATTGACATAATGGGTTATGTGCAACAACGAAGGAAACTAGA
GATTTTCACATACATGAGATTTGACTCTGAATTCACTTTCGTTTGTACTGACTCAACAGGCCAAGGGCCC
AATGTGGTGGTCCAATACATGTACATACCTCCAGGCGCTCCTACGCCAACTGCAAGGGACTCTTTTGCGT
GGCAATCTGCTACAAACCCCTCTGTATTCGTCAAGGCATCTGACCCCCCAGCTCAAGTTTCTGTTCCCTT
CATGTCTCCAGCCTCAGCTTACCAATGGTTCTATGATGGGTACCCCACATTCGGCTCACACCCTGCATCT
GGTGATGTGAACTATGGCATTAGTCCTAACAATCTTTTTGGTACTTTTGCTTTTAGGGTGGTAGGGTCCG
AGAATGTCACAACCAAGCTAACTGTTAGAATTTACATGAGAATCAAACATGTTAGGGCATGGGTCCCTAG
GCCAATGAGAAGCCAGCCATACATATTGAAGAATTACCCAAATTTTGATGGCGACAACATCAAGCACATG
GCCAAGAATAGAGCAAGCATAACCACGACTGGTGCCTTTGGTCAGCAGAGTGGTGCTGTGTATGTTTGCA
ACTACAGAATTGTCAACAGACACCTAGCTACCAAGAATGATTGGGACAACCTTGAGTGGGAGGACTACCA
AAGAGATGTGCTCGTCTCTAGAACCAGTGCACATGGGTGTGACAAGATCGCCAGGTGCAACTGCAACACA
GGAGTGTACTTCTGCAAATCAGCAAACAAACATTATCCAGTTTCCTTTCAAGGCCCTGGTTTTGTACATG
TTCAGGCAAATGAATACTACCCTGAAAGGTACCAATCACATGTGCTATTGGCAAATGGAATCGCTGTAGC
AGGCGATTGTGGTGGCATCCTCAGGTGCCATCATGGTGTGATCGGTTTAGTCACCATGGGTGGTGACGGG
CTAGTAGGCTTTGCTGACTTGAGAGATCTCCTTTGGCTAGAGGATGAAGTGATGGAACAAGGTGTCACTG
ATTACATCAAGGGCTTAGGCGACGCTTTTGGAAGCGGTTTTACAGAGTCCATCGCTAGAGAAGTACAAAA
CATTAAAGACTCACTTGTTGGATCAGAAGGCATAGTTGAGAAAATCCTTAAGAATTTGGTGAAACTGATC
TCAGCATTAGTGATCATTGTGAGGAGTGAGTACGACCTGGTTACAGTCACAGCAACGCTCGCATTAATTG
GGTGCACAGGCAGCCCATGGAGATGGCTTAAAACTAAAGTTGCCTCTATTTTAGGCATTCCCATCGCCCA
AAAGCAATCTGATTCTTGGCTGAAGAAATTCAATGATATGGCAAATGCAGCCCGCGGTCTTGAGTGGATT
GCCAACAAAATTAGCAAGTTCATAGATTGGATTAGAGAGAAAATCTTACCCATTGCAAGAGAGAAGGCTG
AGTTTATCAACAACCTCAAGCAACTCCCTCTACTGGAAAACCAGATCAACAACCTAGAACAGGCTGCTGC
AAGTCAGGAGAGTCTAGAGCAATTATTTGGTAATGTACAATATTTAGCAATAAATTGCAGAAAGTTTCAG
CCGCTTTATGCAGCTGAGGCCAAGAGAGTGTTTTCCATGGAGAAAAGGATGGTAAACTACATGCAGTTCA
AGGGCAAACAACGTATTGAACCTGTATGTCTGATTATCAGAGGGTCTCCAGGCACAGGAAAGTCCCTGGC
AACTAGTATAATTGCAAGAGCTATCGCTGAGAAATTCAACTCCAGTGTGTACTCCCTACCACCGGATCCA
GACCACTTCGATGGATATAAACAGCAAGTGGTAACAGTCATGGATGATTTGTGTCAAAACCCAGATGGTA
AAGATATCTCTTTATTTTGCCAAATGGTTTCAACAGTTGAATTTATCCCACCAATGGCCTCACTGGAAGA
GAAGGGCATTGCTTTCAACTCAAAATTTGTCATTGCATCCACTAACTCTGGCAACATCATCGTGCCAACT
GTGTCCGATTCTGAGGCCATCAGAAGGAGGTTTTTCATGGATTGTGACATTGAAGTTCCAGATAGCTTTA
GACTAGAGAGTGGAAAACTTGATGCAGGCAGAGCAGCAAAATTGTGCTCAGAAAACAACACCGCCAACTT
TAAGAGGTGTAGCCCACTAGTTTGTGGCAAGGCTATCCAATTGAGAGACAGAAAGTCCAAAGTCAGATAC

AGTCTAGACTCAGTGATCTCAGAACTGATTAGAGAGTACAATAATAGAATGTCAGTTGGAAACACCATTG
AGGCTTTGTTCCAAGGCCCACCTCAGTTCAGACCACTCACCATTTCCTTAGACACCCCTGCTCCAGATGC
CATTGCTGATCTTTTGAGGAGCGTGGATAACGAGGAAGTGAGAGAATACTGCAAAGAAAAGGGATGGATC
ATCCCTGAGGTTCCAACCAACCTTGAGAGGCATTACAACAGGGCTATTGCATTAATCCAATCTATCACTA
CTATCCTGGCTGTGATTTCCATGGTGTATGTCATCTACAGATTATTTGCTGGATTTCAGGGTGCCTACTC
TGGGATGCCAAAAGCAGCTGTGAAGAAGCCAGTCCTAAGGACTGCAGTAGCGCAAGGACCAGGACTAGAT
TTCGCCATTTCACTGCTTAAGAAGAACATAAGAAAAGTCCAAACAGAGGAGGGTCACTTCACCATGCTTG
GTGTCAGAGACAGACTTGCAGTGCTACCAAGACATGCAAAACCAGGCAAGACCATCTGGATGGAAGGCAA
GCAAGTGACTGTGTTGGATGCAGTTGAGTTGGTTGATGAGCAGCAGGTAAACTTGGAATTGACTCTGGTG
ACATTGGATATGAATGAGAAATTTAGAGACATCACCAAATTTATCCCTGAATCTTTCGAGCATTGCATGG
ACGCCACTTTGATCATCAATACAGAGCAAATGCCATCTATGTTTGTCCCAGTGGGAGATGTTCAGTTCTA
TGGTTTCCTTAACTTGAGTGGAAAACCTACACACAGGACAATGATGTACAACTTCCCAACCAAAGCTGGT
CAGTGTGGTGGAGTGGTTACAAGTACAGGGAAAGTGATTGGAATTCATATTGGAGGCAACGGAAAGCAAG
GGTTCTGTGCGGCACTGAAGAGAAGCTACTTTGCTACTGAGCAGGGCGAGATACAGTGGATGAAATCTAA
CAAAGAGACTGGCCGCTACAACATCAATGGTCCAACTCAGACCAAACTTGAACCCAGTGTGTTCCATGAT
GTGTTTGAAGGCAAGAAGGAGCCGGCAGCGTTGAGTGCTAAAGACCCGAGGTTGGAGGTTGACTTTGAAG
AGGCTCTTTTCAGTAAATACATTGGCAATGCTATCCATGAACCAGATGAATACATCATTCAAGCAGCCAA
ACATTACTCAAACCAATTGAAGCAATTAAACATTGATACTAGTAGGATGAGTATGGAAGATGCCTGTTAT
GGGACAGAGAACTTAGAAGCAATTGACCTCAGCACCAGTGCAGGATACCCCTACAACGCCTTAGGTATCA
AGAAAAGGCAGATTTTGGACCCTACCACCAGAGATGTCACTAAAATGAAGTACTACATGGACAAATATGG
ATTAGACCTGCCTTTTTCCACATACATCAAAGATGAACTGAGAAGCAAGGAAAAGGTGAAGAAAGGAAAG
TCTAGACTGATTGAAGCATCAAGTCTTAATGACTCAGTGTACATGAGAATGTGCTTTGGACACCTGTTTG
AGAAGTTCCATGAAAATCCAGGCACTATCACCGGCTCTGCGGTGGGAGCGAACCCTGACACCTTTTGGAG
CAAGATCCCCATTCTCCTTCCAGGAAGTCTGTTTGCCTTTGATTACACTGGTTATGATGCTAGCTTGAGC
CCAGCATGGTTTAGAGCGTTGGAAATAGTCTTGAGAGATCTAGGCTATGACGATGAGGCCATCGGTCTCA
TTGAGGGTATTAATCATTCACATCACATCTACCGCAACAAGACATACTGTGTGGTTGGTGGAATGCCCTC
TGGTTGCTCCGGAACCAGTATCTTCAATAGCATGATTAACAACATCATCATCAGAACCCTGTTAATTAAA
ACATTCAAGGGTATTGATTTGGATGAATTGAACATGATTGCTTATGGAGACGATGTGTTGGCTAGCTACC
CATTCCCAATTGACTGCGCTGAACTGGCGAGGACTGGTAGGGAATATGGCTTGGTTATGACACCTGCAGA
CAAATCATCTTGCTTTAATGAGGTCACTTGGGAGAATGCAACATTCTTGAAAAGAAGTTTCAAACCAGAT
GAACAATTTCCATTCTTGATCCACCCAGTGATGCCCATGTCAGAGATCCACGAGTCCATTCGCTGGACTA
AGGACGCACGCAACACTCAAGACCATGTGCGTTCACTGTGTCTTCTGGCTTGGCATGCTGGGAAGGAAGA
GTATAATGAATTTGTTGAAAAGATTAGAACAGTGCCAATTGGTAAGGTACTGTCCATTCCTCAGTATGAT
AATTTGAGGAGAATTTGGCTTGAATCCTTCTAAATTTAAAACTGAATTGATTAATTCGGACCAATTCGGC
TGATCACGCCAGTCCTGGGCTTGCAAAAGCTGGCGTGTGACAATCACCCCCATATATNNNNNNNNNNNNN
NNNNNNNNNNNNNNNNNNNNNNNNNNNNNNNNNNNNNNNNNNNNNNNNNNNNNNNNNNNNNNNNNNNNNN
NNNNNNNNNNNNNNNNNNNNNNNNNNNNNNNNNNNNNNNNNNNNNNNNNNNNNNNNNNNNNNNNNNNNNN
NNNNNNNNNNNNNNNNNNNNNNNNNNNNNNNNNNNNNNNNNNNNNNNNNNNACTGGAGGATAGGGTTTGTT
TGCTGATTTGCAGAAGTACACTCCTGTGTTGCAATTACACCTGGCAATCTTGTCACACCCGTGTGCGCTA
GTTCTAGAAACAAGAACGTCTCTTTGGTAATCCTCCCACTCAAGGTTATCCCAATCATTCTTAGTAGCCA
GGTGTCTGTTGACAATTCTGTAGTTGCAAACATACACAGCACCACTTTGCTGACCAAAGGCTCCAGTTGT
GGTTATGCTTGCTCTATCTTTAGCCATGTGTCTGATGTCGTTGCCATTGAAATTTGGGTAGTTCTTCAAT
ATGTATGGTTGGCTTCTCATTGGTCTGGGGACCCATGCCTTAACATGTTTAATTCTCATGTAAATTCTAA
CAGTTAATTTAGTTGTGACATTCTCGGATCCCACTACCCTAAAAGCAAAGGTACCAAAAAGATTGTTAGG
GCTAATTCCATAGTTTATATCACCAGATGCAGGATGTGTGCCAAACGTAGGGTACCCATCATAGAACCAC
TGGTAAGCTGAGGCTGGCGACATGAAGGGGACAGAAACCTGAGCTGGGGGGTCAGATGCTTTGACAAACA
CAGAAGGGTTAGTGGCAGACTGCCATTCAAAAGAGTCCCTTGCAGTTGGCGCAGGGGCACCTGGAGGAAT
GTACATATATTGAACTACCACGTTGGGTCCAGTGCCTGTGGAGTCAGTACAGACAAAAGTGAATTCAGAA
TCAAACCTCATGTAAGTAAAGATTTCCAATTTTCTCCGTTGCTGCACGTACCCCATTATGTCAATAGGCC
AGTTAGTAAAACCATTTGTTGTGCTTTCTGATGTGAGCAAGGTGGCCATTCCGACAAGAGCTGCTCTGCC

AAAGAAATTCTCCAGACTGGTTTCAGCAACACTGTGGTGGTTATGCACACACCTTGTTTCTATCATGGAC
TCATCAGAGGTGACTGATGTTGCACCTGTCTCAGCGGCTTGCAACGCTGGTGCAGATCCTGTTAAGAGAC
TGTGTGAGCTTGCACTAGTGTCAGCACCGGTACTAGTAGAAGCACCCTCGGTGTTGGCGAGAATTTTGTG
CGTTGTAGCATTAATGTGAGTTTCTAGCATGTCCTTGATATCACCTTGCAGCACTGCAGTTTGGGAGATC
TCGTCGGTGTCTTTACACAGCCGCATAGTAAAGTTTGGCTGGGCGGCACCCAGAGCTATAAGGTATGCAC
TAGTTGGAGCACCTGCTGGAACAACAAAGTTTGTTTGGTACCAAATGGTGACAATACCTGCAGTGTAATA
ATCAAAGTAGGTTCCTGACGCATTCGTTCTAAAATGCGTGTTGCTAATCCAAGGAATCACCAATGTTATG
GATGACTGAAGACCAAAGTCCCAGATGACGTGGGTACCAAGCATTGCCACAGATCTACTTGTAGGCTGCG
CTCCACCAGGGGGTGTGTAAGCTATCAAAAGTTTTCCAGTGGCCATAAAACTGCCTGTGAACATGAAGGT
AACTTCCAATGAACCTGACCACTGAGTATAATATCTGCACAGTTGACCCACCATAGTGGACTGCCATGGG
CCATCTCGACCTGGATCCACTCTGAATGAAGCACATAACTGGTCAACCTCACTCTGAACAGACACTGGTA
TTAAAAGCCTGTCCATCTGAGTTACATTGCTCACATTTTATNNNNNNNNNNNNNNNNNNNNNNNNNNNNN
NNNNNNNNNNNNNNNNNNNNNNNNNNNNNNNNNNNNNNNNNNNNNNNNNNNNNNNNNNNNNNNNNNNNNN
NNNNNNNNNNNNNNNNNNNNNNNNNNNNNNNNNNNNNNNNNNNNNNNNNNNNNNNNNNNNNNNNNNCTAA
GAGCTGCAGGCTCCTTCTTACCCTCAAACACATCATGAAACACACTGGGTTCAAGTTTGGTCTGGGTTGG
ACCATTAATGTTGTAGCGGCCAGTTTCCTTGTTCGGTTTCATCCACTGGATCTCACCTTGCTCGGTGGCA
AAGTAACTCCTCTTCAGTGCCGCACAGAACCCTTGCTTTCCATTACCTCCAACATGAATTCCAATCACTT
TTCCTGTACTAGTAACCACTCCACCACACTGGCCGGCTTTGGTTGGGAAGTTGTACATCATTGTTCTGTG
TGTTGGTTTTCCACTCAGATTCAGGAAGCCATAAAACTGGACATCACCCACTGGAACAAACATTGATGGC
ATCTGCTCTGTGTTGATGATGAGTGTAGCATCCATACAGTGTTCAAATGATTCAGGGATGAATTTAGTAA
TGTCTCTAAATTTCTCATTCATGTCCAATGTGACTAAGGTTAATTCCAAGTTTACTTGCTGCTCATCAAC
TAGTTCAACCGCATCTAGGACTGTCACTTGTTTTCCTTCTAGCCAGATGGTCTTGCCAGGTTTTGCATGC
CTTGGGAGCACTGCAAGCCTGTCTCTAACACCAAGCATGGTAAAGTGTCCCTCTTCAGTTTGCACTTTTC
TGATGTTCTTTTTAAGTAGTGAGATGGCAAAGTCTAAGCCTGGCCCTTGTGCTACTGCTGTTCTTAAGAC
TGGTTTCTTCACTGCCGTCTTGGGCATTCCGGAGTAAGCACCTTGGAATCCAGCGAACAGCCTGTAGATG
ACATACACCATAGATACAACAGCCAGAATGGTAGTGATGGACTGGATTAGTGCAATTGCTTTATTGTATT
GTCTCTCTAAATTTGTTGGTACCTCGGGGATGATCCACCCTTTTTCCTTGCAGTATTCTCTCACTTCTTC
ATTGTCAACACTTCTCAAGAGGTCGGCAATAGCGTCTGGGGCTGGAGTGTCCAAAGAAATGGTGAGAGGT
TTAAACTTTGGTGGGCCTTGGAATAATGCCTCAATTGTATTTCCAACACTGTTTCTGTTGTTGTATTCTC
TGATAAGTTCTGAGACAACTGAATCTAAACCGTATCTCACCTTAGATTTCCTATCTTTTAACTGAATAGC
TTTTCCACAAACTAGAGGGCTACACTTCTTAAAGTTAGCTGTGTTGTTCTCAGAGCACAGTCTGGCTGCT
CTCGAGGCATCCAACTTTCCATTCTCAAGTCTGTAAGATTCAGGGACCTCAATGTCACAGTCCATGTAAA
AGCGGCGTCTGATTGCTTCGCTGTCTGAAACAGTGGGGACTATGATATTGCTAGAATTCGTGGAAGCAAT
GACAAACTTTGATGTGAAGGGCAGACCCTTCTCTTCTAGTGATGCCATAGGTGGTATAAACTCAACAGTA
GAAACCATTTGACAAAACAAGGAGATGTCTTTCCCGTCTGGGTTCTGGCACAGGTCATCCATCACAGTGA
CAACTTGTTGACGGTAACCATCGAAGTGGTCAGGGTCAGGTGGTAGGGAGTATACGCTAGAGTGGTATTT
CTCAGCAATAGCCCTAGCAATGATGCCTGTAGCAAGAGATTTTCCTGTGCCTGGAGCACCACGGATGATC
AAACATACAGGTTCAATCCGTTGTTTGTTCTTGAACTGCATGTAGTTGTTGACTCTGCGCTCCAATGTCG
CAATTCTGCGTGCTTCAGAGGCATACAAAGGTTGGTATTTCCTGCAATGGATGGCAAGGTACTGCACATT
CCCGAAGAGAGCTTCTAGGTCGTCCTGGCTAGCTGCTGCTTGTTCTAAGTTGCTAACCTGGTTCTCCAAC
AAGGGCAGTTGTTTGAGATTGTTAAGGAACTCTACCTTTTCTCTTGCAATAGGTATGATCTTTTCTCTTA
TCCAATCAATGAACTTGCTAATTTTGTTCGCAATCCATTCAAGGCCACGAGCTGCGTTGGCCATGTCATT
GAATTTCTTGAGCCAAGAGTCAGATTGTTTTTGGGCGATGGGGATGCCTAGAATTGATGCGACCTTTGTT
TTAAGCCATCTCCATGGACTACCTGTACAACCAATCAGTGCCAATGTTGCTGTGACAGTAACCAGATCGT
ACTCACTCCTCACAATTATGACCAGAGCGGATATCAACTTGACCAGGTTCTTCAGGATTTTCTCTACTAC
ACCTTCTGATCCAACGAGTGTATCTTTGATGTTCTGGACCTCTCTGGAGATTGACTCGGTGAAACCGGTT
CCGAAAGCGTCGCCCAAGCCCTTAATGTAGTCAGTGACACCCTGTTCCATCACTTCATCCTCTAGCCAAA
GGAGGTCTCTCAGGTCAGCAAAACCCACCAACCCATCACCGCCCATGGTAACTAAGCCAATCACACCATG
GGGGCACCTAAGGATGCCACCACAATCACCTGCTACAGCGATCCCATTTGCTAATAGTACATGTGATTGG
TATCTTTCAGGGTAGTACTCGTTTGCTTGGACGTGCACAAGACCAGGGCCCTGGAAGGAACTGGGTAAT

GTTTGTTTGCTGATTTGCAGAAATACACTCCTGTGTTGCAGTTGCACCTGGCGATCTTGTCACACCCATG
CCCAGTGGTTCTGGAAACAAGAATGTCTCTCTGATAGTCCTCCCATTCAAGGTTGTCCCAATCATTCTCA
GTAGCCAGGTGTCTGTTGACAATCCTGTAATTACACACATACACAGCACCACTCTGCTGACCAAAGGCTC
CAGTTGTGGTTATGTTAGCCCTGTTTACAGCCATGTGCCTGATATTATTGCCATTAAAGTTTGGGTAGTT
CTTCAACACATACGGCTGGCTTCTCATTGGTCTAGGGACCCATGCCCTAACGTGTTTAATTCTCATGTAA
ATTCTGACAGTCAATTTGGTTGTGACATTCTCAGACCCCACCACTCTAAAAGCAAAGGTGCCAAAAAGAT
TGTTAGGACTGATCCCATAATTCACATCACCAGATGCAGGGTGTGTACCGAATGTGGGGTACCCATCATA
GAACCATTGATAAGCTGAAGCTGGAGACATGAAAGGAACAGAGACCTGAGCTGGAGGGTCAGATGCCTTG
ACGAACACAGAGGGGTTTGTGGCAGATTGCCACTCAAAAGAGTCCCTTGCAGTTGGGGCAGGAGCACCTG
GGGGAATATACATGTACTGAACCACCACATTGGGCCCTTGGCCTGTTGAGTCGGTACAAACAAAAGTGAA
CTCAGAGTCAAACCTCATGTATGTGAAAATTTCCAGCTTCCTCCGCTGTTGTACATAGCCCATTATGTCA
ATTGGCCAGTTGGTAAAACCATTTGTTGTGCTCTCTGATGTGAGTAGAGTGGCCATCCCAACAAGGGCTG
CCCTGCCAAAGAAGTTTTCCAGACTCGTTTCAGCAACACTGTGGTGGTTGTGAACACACCTTGTCTCTAT
CATAGACTCATCAGAGGTAACTGATGTTGCACCCGTCTCAGCTGCCTGTAGCGCTGGTGCAGACCCTGTC
AATAAGCTGTGTGAGCTTGCACTGGTGTCAGCGCCGGTGCTAGTAGAGGCGCCCTCAGTGTTGGTAAGAA
CTTTGTGTGCTGTAGCATTGATGTGAGTTTTTAGCATATCCTTGATATCACCTTGCAACACTGCAGTTTG
AGAGATTTCGTCAGTGTCTTTACACAGCCGCATGGTGAAATTTGGTTGAGCAGCGCCCAGAGCTATGAGG
TAAGCACTGGTTGGAGCACCTGCTGGAACAACAAAGTTTGTTTGGTACCAAATGGTGACAATACCTGCAG
TGTAATAATCAAAGTAGGTTCCTGACGCATTCGTCCTAAAATGCGTGTTGCTAATCCAAGGAATCACCAA
TGTAACGGATGACTGAAGACCAAAGTCCCAGATAACATGGGTGCCAAGCATTGCCACAGCTCTACTTGTA
GGCTGCACTCCACCAGGAGGTGTGTAAGCTATCAAGAGTTTTCCAGTGGCCATAAAACTGCCTGTAAACA
TGAAGGTAATTTCTAATGAACCTGACCATTGAGTGTAATATCTGCACAGTTGACCCACCATAGTGGACTG
CCATGGACCATCTCGGCCTGGATCCACTCTAAAGGAGGCACATAGCTGGTCGACCTCACTCTGAACAGAC
ACTGGTATCAAAAGTCTTTCCATTTGAGATGCACTCTCCACATTGTTTACTTCAAGTATGCTTTCAATTT
GACACAGTTGTAGCAAGTTGTTTACTTCACCTGGAATGTGAATCAAGGGGGTTGGGTGGAAGCCAGGAAG
TATGGGTGCTGAAACACCATCATCTGTGGTAAGAAATTGGTTGCTGCCAGGTTTCAACTCTACTGGCAAG
CCCTGAGTAACTGCATTCCTCAGACCGGCAAACTCAGCACACAGTGGTGCAATGGTGACTGTTATTGGCA
CAGCCGTTGTGGCTCCATCACTATACGCTAGTGGGGCAACGGGAATAACAAACAAGCCAAAGTTGCAGTG
ATTTAGGGCCGAATCATAAGGCACTGCATTCACATAGGGCATGATTATGGTGGCACAATTATTGGTCCTG
AGGTTAATCCATTGGTGAGGGGCAACAAGCAATTGAGAGAAAGCTATCCCTGAATCAAGGACATAAGGTA
TAGCAAGTTCCTTACCGTTTTTGCCTGGCTGAGCTGCTGCATAAGATGGGTGCCTTGCAGTGGCAGGGTC
AGTTGCGCTGTCAGAGCGGGCCACAGTGAATTCTGGGATAACAGCGACTAACAAGGCCCCCTGGTGGAAC
TTGCTCGCATTACATTGTACATGTATGCAGAAACCTGATCTGTATAGGTAGTGGAACTGTGCATTCTGCC
CGAAAACACCTGTCTCTGTTAACACATCGGGGAATTTCCAGTACCATCCCTTGGAGGTTTTTTCCCATAA
TTTAGCACTTAGGGTGTAAAACCTGTTCACTGATACATCTGGTTTAGATGGCTTGTCCACAGCTGTTGCA
TCTGTGTTTGAGCAAAATTCAGGCCATTCACCATAGCCTACTGTTATGTTGGCAGCTTCCTGGGTTGTGA
TGGTGGAATTACCTAGAGTAAGTTGGGCCACTCTATCGCTGTAACCACACGCTTCAGCTGAGGGGGATTT
AAGAGGCACAGAAGACTCCTTTATCATATCCACTACAGGTTGAGTAAACTTAGATGGGTCCTGACTGAAG
TCTTGTTTAGTTGCCGAAGCAGCATAAGATTCTTTGTAATAATTTATGGTGGTGTAATTGATGGTGGAAC
CACCAGTCGCCATGTTGGCATTTTCGTGAGAGCCAGTTTTCTGAGTGGAAACTTGGGCACCCATAATGTT
GTTGGTAAATGATAACTGCTTGGTATCAAGAGGTGAATCACACAAAAGATGTATCCGGATGGCCAATCCA
ATCGCTATATGATGACAACTCTAGATTGTCACCATAAGCAGCCAAGGTAAATAAAACAGGAAACACGGAC
ACCCAAAGTAGTCGGTTCCGCCACAGAATTACTCGTTACGACCACAACACCACTGGATTGTAGTGCATGG
CTCCGTGGTTAGGATTAGCCGCATTCAGGGGCCGGAGGACTACTACCTAGCTCAATAGGCTCTTCGCACC
ATGTCATGAATCAAGCGTCCTGCGAGTGAGACTCGCAGGCAGGCCGCCAACGCAGCCACCGACACGGTCG
CCCGTGGGGAATTCGGTGACTCATCGACCTGAACTACACTGGGGTTGTGCTGAGCGTAACACTCGAGACA
TTCAATGGTGGTACTAGGCTTCTCGAAGTAGGTAGCCGGATAACGAACGTTTTCCCTTTCGGCCGTGGGC
ACAGCCTATTGGTACTAAGTCCGGGGAAACAGAAGTGCTTGACTAAAAACCCACCTGGGTTGGTGGATTT
GCTAATTGTCGTTTGTGCTTTCTTCTAACGTTACGNNNNNNNNNNNNNNNNNNNNNNNNNNNNNNNNNNN
NNNNNNNNNNNNNNNNNNNNNNNNNNNNNNNNNNNNNNNNNNNNNNNNNNNNNNNNNNNNNNNNNNNNNN

NNNNNNNNNNNNNNNNNNNNNNNNNNNNNNNNNNNNNNNNNNNNNNNNNNNNNNNNNNNNNNNNNNNNNNNN
NNNNNNNNNNNNNNNNNNNNNNNNNNNATAGCTTAGGGTAATTGTCACACGCCAGCTTTTACAAGCCCAGA
ACTGGCGTGATCAGCCGAATTGGTTCAAATTAGTCAATTCAGTTTCAAATTTAGAAAGATTCAAGCCGAA
TTCTCCTCAGATTGTCATACTGAGGAATTGACAGCACCTTGCCAATTGGTACTGTTCTGATCTTTTCAAC
AAATTCATTGTACTCCTCCCTCCCAGCGTGCCAAGCCAAGAGACACAGCGAACGCACGTGATCTTGAGTG
TTGCGCGCATCCTTAGTCCAGCGAATGGACTCGTGGATCTCTGACATGGGCATGACAGGGTGGATCAAGA
ATGGAAATTGTTCATCTGGTTTGAAACTTCTTTTCAAGAATGTTGCATTTTCCCAAGTGACCTCATTAAA
GCAAGAAGATTTGTCTGCAGGTGTCATAACCAAACCATATTCCCTACCAGTCCTTGCAAGTTCTGCACAA
TCAATTGGGAATGGGTAACTAGCTAACACATCATCTCCATAGGCAATCATGTTCAATTCATCCAAATCAA
TGCCCTTGAATGTTCTAATCAGCAAGGTCCTGATGATAATGTTGTTGATCATGCTATTGAAGATACTGGT
TCCAGAGCAGCCTGAGGGCATGCCACCAACCACGCAGTATGTTTTGTTGCGATAGATGTGGTGAGAATGA
TTTATGCCCTCGATGAGGCTAATAGCCTCATCATCATAGCCTAGATCTTTCAAAACTATTTCTAGTGCTC
TGAACCATGCTGGACTTAAGCTAGCGTCATAGCCGGTGTAATCAAAAGCAAACAAGCTGCCTGGGAGAAG
GATGGGGATCTTGCTCCAGAAGGTGTCAGGGTTTGCTCCTACTGCAGAGCCTGTGATGGTGCCTGGATTC
TCGTGGAACTTTTCAAACAGGTGTCCAAAACACATTCTCATATACACTGAATCATTGAGACTTGATGCCT
CAATCAGTCTAGACTTCCCCTTCTTCACTTTTTCTCTGCTCCTCAGTTCATCCTTGATGTATGTAGAAAA
GGGTAGATCCAATCCATATTTATCCATGTAATATTTCATCTTGGTGACATCTCTAGTGGTAGGGTCCAAA
ATCTGCCTCTTCTTGATGCCCAAAGCATTGTAGGGGTATCCTGCACTGGTACTAAGGTCAATTGCTTCTA
AGTTCTCAGTCCCATAGCAGGCATCTTCCATGCTCATCCTACTGGTGTCAATGTTCAGTTGCTTTAGTTG
GTTTGAGTAATGTTTGGCTGCTTGGATGATGTACTCATCTGGTTCATGGATAGCATTGCCAATATATTTA
CTGAACAGGGCTTCCTCAAAATCAACTTCCAACCTTGGGTCCTTTGCACTAAGTGCTGCAGGCTCCTTCT
TACCCTCAAACACATCATGAAACACGCTGGGTTCAAGTTTGGTCTGGGTTGGACCATTGATATTGTAGCG
GCCAGTCTCTTTGTTAGATTTCATCCATTGGATCTCACCTTGCTCTGTAGCAAAGTAGCTTCTCTTCAAT
GCCGCACAGAACCCTTGCTTTCCATTACCTCCAACATGAATTCCAATCACTTTCCCTGTACTAGTAACCA
CTCCACCACACTGACCAGCTTTGGTTGGGAAATTGTACATCATTGTCCTGTGCGTTGGTTTTCCACTCAA
ATTCAGGAAGCCATAAAACTGAACATCTCCTACTGGGACAAACATTGATGGCATCTGCTCCGTATTGATA
ATCAGGGTGGCATCCATACAGTGTTCAAAAGACTCAGGAATAAATTTAGTGATGTCTCTGAATTTCTCAT
TCATGTCTAATGTTACCAGGGTCAATTCCAAATTCACCTGCTGTTCATCAACTAGTTCAACTGCATCTAA
TACAGTGACCTGTTTGCCTTCCATCCAGATGGTCTTGCCTGGTTTTGCATGTCTTGGTAGCACTGCAAGT
CTGTCTCTAACACCAAGCATAGTGAAGTGTCCCTCTTCAGTCTGCACCTTTCTGATGTTCTTTTTGAGGA
GTGAGATGGCAAAGTCTAAGCCTGGACCCTGTGCTACTGCTGTTCTCAAGACTGGTTTCTTCACTGCTGT
TTTGGGCATTCCAGAGTAAGCACCCTGAAATCCAGCAAACAACCTATAGATGACATACACCATAGATACT
ACAGCCAGAATGGTAGTGATAGACTGGATCAGTGCGATTGCCCTGTTGTAGTGTCTTTCAAGATTGGTTG
GTACATCAGGGATTATCCACCCCTTCTCCTTGCAATACTCTCTGATTTCCTCGTTGTCTACACTTCTCAG
AAGATCTGCAATAGCATCTGGGGCTGGTGTGTCTAAAGAGATTGTGAGGGGCTTGAATTTTGGTGGACCT
TGGAACAGTGCCTCAATGGTGTTTCCAACAGACATTCTGTTGTTGTACTCTCTGATAAGTTCCGAGACAA
CTGAATCCAGACTGTATCTCACCTTGGATTTTCTGTCTCTTAACTGAATAGCTTTACCACAGACCAGTGG
GCTACATCTCTTGAAATTGGCAGTGTTGTTCTCTGAACACAATTTTGCTGCTCTGCCTGCATCTAACTTA
CCACTATCTAACCTGAAACTGTCTGGGACTTCAATATCACAATCCATGTAGAATCTTCTCCTGATGGCCT
CTGAATCAGACACAGTTGGCACAATTATGTTGCCAGCATTAGTGGATGCAATGACAAATTTTGAGTTGAA
TGCAATGCCCTTTTCCTCCAGTGAAGCCATTGGTGGTATGAACTCAACTGTAGAGACCATTTGACAAAAG
AGTGAGATGTCCTTCCCATCAGGGTTTTGACACAAATCATCCATAACTGTAACCACCTGTTGCTTGTAAC
CATCGAAATGGTCAGGGTCTGGTGGTAGAGAGTACACACTGGAGTTAAATTTTTCAGCAATGGCCCTGGC
AATAATGCTGGTTGCTAATGATTTACCTGTTCCTGGGGAGCCTCTGATGATCAGACATACAGGTTCAATA
CGTTGTTTGCCCTTGAACTGCATGTAATTGACCATTCTCTTCTCCATTGAGAAGACCCTCTTGGCTTCAG
CTGCATAGAGCGGTTGGAATTTCCTGCAATTGATTGCTAAGTACTGTACATTACCAAACAGCTGCTCCAG
GCTCTCCTGGCTTGCAGCAGCTTGTTCTAAGTTGTTGATCTGGTTTTCCAACAAGGGAAGTTGCTTGAGG
TTGTTGATAAATTCAGCCTTCTCTCTAGCAATAGGTAAGATCTTTTCTCTAATCCAATCTATGAACTTAC
TAATCTTGTTGGCGATCCACTCAAGACCGCGGGCTGCATTTGCCATATCATTGAACTTCTTCAACCAGGA
GTCAGACTGCTTCTGAGCGATTGGTATACCCAGGATGGAGGCAACTTTGGTTTTGAGCCACCTCCATGGA

CTACCTGTGCAACCTATTAATGCAAGTGTTGCAGTGACTGTAACCAGGTCGTATTCACTCCTCACAATGA
TCACCAATGCTGAAATCAACTTCACCAAATTCTTAAGGATTTTCTCAACTATGCCTTCTGATCCAACAAG
CGAGTCTTTAATATTCTGCACTTCTCTAGCGATGGATTCTGTAAAACCACTTCCAAAAGCATCGCCCAAG
CCCTTGATGTAGTCAGTGACACCTTGCTCCATTACTTCATCCTCTAGCCAAAGGAGGTCTCTCAAGTCAG
CGAAGCCCACTAATCCATCACCACCCATAGTGACCAAGCCAATCACACCATGGGGACACCTAAGAATGCC
ACCACAATCACCTGCCACAGCAATCCCATTTGCCAATAGTACATGTGATTGGTATCTTTCAGGGTAATAT
TCATTTGCTTGGACATGTACAAAACCAGGGCCCTGGAAGGAAACCGGGTAATGCTTGTTTGCTGATTTGC
AGAAGTACACTCCTGTGTTACAGTTGCATCTGGCAATTTTGTCACACCCATGCGCACTTGTTCTGGAAAC
AAGGATGTCTCTCTGGTAATCTTCCCATTCAAGATTGTCCCAATCATTCTTGGTGGCCAGATGTCTGTTA
ACAATTCTGTAGTTGCAAACATATGCAGCGCCACTCTGTTGACCAAAAGTCCCAGTTGTAGTTATGCTTG
CCCTATCCTTAGCCATGTGTCTGATGTTATTGCCATCGAAATTTGGGTAGTTCTTTAATATGTATGGCTG
ACTTCTCATTGGTCTAGGGACCCATGCCTTAACATGTTTGATTCTCATGTAAATTCTGACAGTTAGTTTG
GTTGTGACATTCTCAGATCCTACTACTCTAAAAGCAAAAGTACCAAATAGATTGTTGGGACTGATTCCAT
AGTTCACATCACCAGATGTAGGGTGTTGACCGAATGTAGGGTACCCATCATAAAACCACTGGTAAGCTGA
GGCTGGTGACATGAAAGGAACAGAAACCTGAGCTGGGGGGTCGGATGCCTTGACAAACACAGAGGGGTTT
GTGGCAGATTGCCACTCAAAAGAGTCCCTTGCAGTTGGTGCAGGAGCACCTGGGGGAATGTACATGTATT
GAACCACCACATTGGGCCCTTGGCCTGTCGAGTCAGTACAAACGAAGGTGAACTCAGAGTCAAATCTCAT
GTATGTGAAAATTTCCAGCTTCCTCCGCTGTTGCACATAGCCCATTATGTCAATTGGCCAATTGGTAAAA
CCATTTGCTGTGCTCTCAGATGTGAGTAGAGTAGCCATTCCAACAAGAGCTGCCCTGCCGAAGAAATTTT
CTAGACTGGTTTCAGCAACACTGTGGTGGTTGTGAACACACCTTGTCTCTATCATAGACTCATCAGAGGT
GACCGATGTTGCACCTGTCTCAGCTGCCTGCAAGGCTGGTGCAGATCCTGTTAAAAGGCTGTGTGAGCTT
GCACTAGTGTCAGCGCCGGTACTGGTGGAGGCACCCTCGGTGTTGGCAAGAACTTTGTGTGTTGTAGCAT
TAATGTGGGTTTCTAGCATGTCCTTAATATCACCCTGCAGCACCGCAGTTTGGGAGATTTCATCAGTATC
CTTACACAGCCGCATGGTGAAGTTTGGTTGGGCAGCACCCAGGGCTATAAGGTATGCGCTAGTTGGAGCG
CCTGCTGGGACAACGAAGTTTGTTTGATACCAAATGGTGACAATACCTGCAGTGTAATAGTCAAAGTAGG
TTCCTGACGCATTTGTCCTAAAATGCGTATTGCTAATCCAAGGAATGACCAATGTTATGGATGACTGAAG
ACCAAAGTCCCAGACGACGTGGGTGCCAAGCATTGCCACAGATCTACTTGGAGGCTGCGTTCCACCTGGA
GGTGTGTAAGCTATCAAGAGTTTTCCAGTGGCCATAAAGCTACCTGTAAACATGAAGGTGACTTCCAGTG
AACCTGACCACTGGGTGTAATATCTACACAGTTGACCCACCATAGTGGACTGCCATGGGCCATCTCGACC
TGGATCCACTCTAAAGGAGGCACATAGCTGGTCGACCTCACTCTGAACAGACACTGGTATCAAAAGTCTT
TGCATCTGGGATGCACTGCTCACATTGTTCACTTCAAGTATACTTTCAACTTGACACAGTTGTAGCAAAT
TGTTTACTTCACCTGGGATGTGAATCAAGGGGGTTGGGTGGAAGCCAGGAAGTATGGGTGCTGCAACACC
ATCATCTGTGGTGAGAAATTGGTTACTGCCAGGTTTTAACTCCACTGGTAAGCCCTGTGTAACAGCATTC
CTCAAACCGGCAAACTCAGCGCAGAGTGGCGCAATGGTGACTGTTATGGGTACAGCTGTTGTGGCCCCAT
CACTGTAAGCTAGTGGGGCAACAGGAACAACAAACAAGCCAAAGTTGCAATGGTTTAGGGCTGAATCATA
AGGTACTGCATTCACATAAGGCATGATTATGGTGGCACAATTATTTGTTCTGAGATTGATCCACTGATGG
GGAGCAACAAGTAGCTGGGAAAAGGCTACCCCAGAGTCCAATACATAAGGGAAGGCCAATTCCTTACCAC
TGGGTCCAGGTTGTGTGGCATTGTACGAGGGGTGTTTCGCACTACCTGGAGTGTTGGTTGCATCACTCCT
TCCATTAGTGTACTCAGGTAAACACGCCACTAGGAGGGTTCCCTGATGGAACTTACTTGCATTGCATTGT
ACATGGATGACAAAGCCTGATCTATATAAGTAGTGAAACTGTGCATTCTGGCCAAAAACACCTGTCTCGG
TCAGGACATCAGGAAATTTCCAGTACCACCCCTTGCTGTTTGATTGCCACAATTTTGCTGATAGTGTATA
AAATCTATTAACCGAAACATCTGGTCTAGTTGGCTTGTCAACGGCAGTAGCATCTGAATCACTACAGTAT
TCGGGCCACTCGCCGTATCCCACAATCACATTGGCGGCCTCTTGTGTGGTGATGGTGGAGTTCCCAACTG
TCATCTGGCAGACCCTGTCACTGTAACCACACGCCTCTGCTGAAGGAGATTTGAGTGGTACTGCTGATTC
TTTAAGGACATCTACCACTGGTTGGGTAAATTTGCTGGGGTCTTGACTAAAATCCTGCTTGTTAGCCGAT
GCAGCGTAGGAATCCTTGTAGTAGTTTATGGTGGTGTAGTGGATGGTGGATCCACCTGTAGCCACGTTGG
TGTTCTCGTGGGTTCCAGATTGCTGAGTGGAAACTTGAGCTCCCATGATGATGTTGTAAGTAAATGTATT
TGCTTTGAAGGCAGGGAAAAATCACACAAGTAATATATCCGGATGGCCAATCCAATCGCTATATGATGAC
AACTCTAGATTGTCACCATAAGCAGCCAAGGTAAATAAAACAGGAAACACGGACACCCAAAGTAGTCGGT
TCCGCCACAGAATTACTCGTTACGACCACAACGTCACTGGATTGTGCCGCATGGCTCCGTGGTTAGGATT

AGCCGCATTCAGGGGCCGGAGGACTACTACCTAGCTCAATAGGCTCTTCGCACCATGTCATGAATCAAGC
GTCCTGCGAGTGGAACTCGCAGGCAGGCCGCCAACGCAGCCACCGACACGGTCGCCCGTGGGGAATTCGG
TGACTCATCGGCCTGAACTACACTGGGGTTGTGCTGAACGTAACACTTGAGACATTCAATGATGGTACTA
GGCTTCTCGAAGTAGGTAGCCGGGTAACGAACGTTTTCCCCTTCAGCCGTGGGTACAGCCTATTGGTACT
AGGTCCGGGGAAACAGAAGTGCTTGACGAAGATCCGCCTGGTTTGGCGAATCTGCTATTTGTCATTGGTG
CTTACTTCTAACGTTACGGGGGAGGGGAGATAAAACAGGCATACAAAGGTACCGTAATACCAGAGTACTA
TCCTCACGTATCCCCYDTBCGTGGGTATATNNNNNNNNNNNNNNNNNNNNNNNNNNNNNNNNNNNNNNNN
NNNNNNNNNNNNNNNNNNNNNNNNNNNNNNNNNNNNNNNNNNNNNNNNNNNNNNNNNNNNNNNNNNNNNN
NNNNNNNNNNNNNNNNNNNNNNNNNNNNNNNNNNNNNNNNNNNNNNNNNNNNNNNNNNNNNNNNNNNNNN
NNNNNNNNNNNNNNNNNNNNNNNNNNNNNNNNNNNNNNNNNNNNNNNNNNNNNNNNNNNNNNNNNNNNNN
NNNNNNNNNNNNATATATTTTTGTGTGAATCACTCTTTGCTCTTTAGCATTTGCAATACTTCATAGTTTC
ATCATGGGAGCTCAAGTTTCCACTCAACAATCTGGAACCCACGAGAACACCAACGTGGCTACAGGTGGAT
CCACCATCCATTACACCACTATTAACTATTACAAGGACTCCTACGCTGCATCAGCTAATAAGCAAGACTT
CAGCCAGGACCCAAGTAAGTTCACTCAACCAGTGGTTGATGTTTTAAAGGAATCATCAGTGCCTCTCAAG
TCTCCTTCAGCAGAAGCATGTGGTTACAGCGACAGGGTTTGCCAAATGACAGTTGGAAATTCCACCATTA
CTACGCAGGAGGCTGCTAATGTGATTGTAGGATATGGCGAATGGCCTGAGTACTGTAGTGATTCAGACGC
CACCGCTGTTGATAAACCAACTAGACCAGATGTCTCGGTTAATAGATTTTACACTCTGTCAGCAAAATTG
TGGCAATCTAACAGTAAGGGATGGTATTGGAAATTTCCTGATGTTCTAACTGAGACAGGTGTTTTTGGCC
AGAATGCGCAGTTCCATTACTTATATAGGTCAGGCTTCGCCATTCATGTGCAGTGCAATGCAAGCAAGTT
TCACCAAGGAACTCTCCTGGTGGCATGTTTACCAGAATACACCAATGGAAGAAGTGATGCAGCCAACACA
CCTAACTCCGCCAAGCACCCATCATACGTTGCCACGCAACCTGGACCCAACGGCAAAGAGTTGGCCTTCC
CCTATGTATTGGATTCTGGAGTGGCTTTCTCCCAGCTACTTGTCGCTCCTCACCAGTGGATCAACCTCAG
GACAAACAATTGTGCCACCATTATTATGCCTTATGTGAATGCAGTTCCTTATGACTCAGCCCTAAATCAC
TGCAACTTTGGTCTGTTTGTTGTTCCTGTTGCCCCATTGGCGTACAGCGATGGAGCCACAACGGCTGTGC
CCATAACAGTTACCATTGCACCACTTTGTGCTGAGTTTGCTGGCCTAAGGAATGCTGTCACACAGGGTTT
GCCAGTGGAGTTGAAGCCTGGCAGTAACCAGTTCCTTACTACAGATGATGGTGTTGCGGCACCCATACTC
CCTGGCTTCCACCCAANNNNNNNNNNNNNNNNNNNNNNNNNNNNNNNNNNNNNNNNNNNNNNNNNNNNNN
NNNNNNNNNNNNNNNNNNNNNNNNNNNNNNNNNNNNNNNNNNNNNNNNNNNNNNNNNNNNNNNNNNNNNN
NNNNNNNNNNNNNNNNNNNNNNNNNNNNNNNNNNNNNNNNNNNNNNNNNNNNNNNNNNNNNNNNNNNNNN
NNNNNNNNNNNNNNNNNNNNNNNNNNNNNNNNNNNNNNNNNNNNNNNNNNNNNNNNNNNNNNNNNNNNCC
CTTCAGCAGAGGCGTGTGGTTACAGCGACAGAGTCTGCCAAATGACAGTAGGAAATTCCACCATTACCAC
ACAGGAGGCTGCTAATGTGATTGTAGGGTATGGCGAGTGGCCCGAGTACTGTAGTGATTCAGATGCCACT
GCTGTCGACAAACCAACTAGACCAGATGTTTCGGTTAACAGATTTTACACACTATCAGCAAAATTGTGGC
AATCAAACAGCAAGGGGTGGTATTGGAAATTTCCTGATGTCTTAACTGAGACAGGTGTTTTTGGCCAGAA
TGCACAGTTTCACTACTTATACAGATCAGGCTTTGCTGTCCACGTACAATGCAATGCAAGCAAGTTTCAC
CAGGGAACCCTCCTGGTGGCATGTTTACCTGAATACACCAATGGGAGGAGTGATGCAACTAATACACCCG
GTTCTGCAAAGCACCCATCGTATAATGCCACTCAACCTGGACCCAGTGGTAAAGAATTGGCCTTTCCCTA
TGTGTTAGATTCTGGGATAGCTTTCTCTCAATTGCTTGTTGCCCCTCACCAATGGATTAACCTCAGGACC
AATAATTGTGCCACCATAATCATGCCCTATGTGAATGCAGTGCCTTATGATTCGGCCCTAAATCACTGCA
ACTTTGGCTTGTTTGTTATTCCCGTTGCCCCACTAGCGTATAGTGATGGAGCCACAACGGCTGTGCCAAT
AACAGTCACCATTGCACCACTGTGTGCTGAGTTTGCCGGTCTGAGGAATGCAGTTACTCAGGGCTTGCCA
GTAGAGTTGAAACCTGGCAGCAACCAATTTCTTACCACAGATGATGGTGTTTCAGCACCCATACTTCCTG
GCTTCCACCCAACCCCCTTGATTCACATTCCAGGTGAAGTAAACAACTTGCTACAACTGTGTCAAATTGA
AAGCATACTTGAAGTAAACAATGTGGAGAGTGCATCTCAAATGGAAAGACTTTTGATACCAGTGTCTGTT
CAGAGTGAGGTCGACCAGCTATGTGCCTCCTTTAGAGTGGATCCAGGCCGAGATGGTCCATGGCAGTCCA
CTATGGTGGGTCAACTGTGCAGATATTACACTCAATGGTCAGGTTCATTAGAAATTACCTTCATGTTTAC
AGGCAGTTTTATGGCCACTGGAAAACTCTTGATAGCTTACACACCTCCTGGTGGAGTGCAGCCTACAAGT
AGAGCTGTGGCAATGCTTGGCACCCATGTTATCTGGGACTTTGGTCTTCAGTCATCCGTTACATTGGTGA
TTCCTTGGATTAGCAACACGCATTTTAGGACGAATGCGTCAGGAACCTACTTTGATTATTACACTGCAGG
TATTGTCACCATTTGGTACCAAACAAACTTTGTTGTTCCAGCAGGTGCTCCAACCAGTGCTTACCTCATA

GCTCTGGGCGCTGCTCAACCAAATTTCACCATGCGGCTGTGTAAAGACACTGACGAAATCTCTCAAACTG
CAGTGTTGCAAGGTGATATCAAGGATATGCTAAAAACTCACATCAATGCTACAGCACGATATCTTCTTAC
CAACACTGAGGGCGCCTCTACNNNNNNNNNNNNNNNNNNNNNNNNNNNNNNNNNNNNNNNNNNNNNNNNN
NNNNNNNNNNNNNNNNNNNNNNNNNNNNNNNNNNNNNNNNNNNNNNNNNNNNNNNNNNNNNNNNNNNNNN
NNNNNNNNNGAGGATAACGGGTTGGGTGGAAGCCAGGAAGTATGGGTGCTGAAACACCATCATCTGTGGT
AAGAAATTGGTTGCTGCCAGGTTTCAACTCTACTGGCAAGCCCTGAGTAACTGCATTCCTCAGACCGGCA
AACTCAGCACACAGTGGTGCAATGGTGACTGTTATTGGCACAGCCGTTGTGGCTCCATCACTATACGCTA
GTGGGGCAACGGGAATAACAAACAAGCCAAAGTTGCAGTGATTTAGGGCCGAATCATAAGGCACTGCATT
CACATAGGGCATGATTATGGTGGCACAATTATTGGTCCTGAGGTTAATCCATTGGTGAGGGGCAACAAGC
AATTGAGAGAAAGCTATCCCAGAATCTAACACATAGGGAAAGGCCAATTCTTTACCACTGGGTCCAGGTT
GAGTGGCATTATACGATGGGTGCTTTGCAGAACCGGGTGTATTAGTTGCATCACTCCTCCCATTGGTGTA
TTCAGGTAAACATGCCACCAGGAGGGTTCCCTGGTGAAACTTGCTTGCATTGCATTGTACGTGGACAGCA
AAGCCTGATCTGTATAAGTAGTGAAACTGTGCATTCTGGCCAAAAACACCTGTCTCAGTTAAGACATCAG
GAAATTTCCAATACCACCCCTTGCTGTTTGATTGCCACAATTTTGCTGATAGTGTGTAAAATCTGTTAAC
CGAAACATCTGGTCTAGTTGGTTTGTCGACAGCAGTGGCATCTGAATCACTACAGTACTCGGGCCACTCG
CCATACCCTACAATCACATTAGCAGCCTCCTGTGTGGTAATGGTGGAATTTCCAACTGTCATTTGGCAGA
CTCTGTCGCTGTAACCACACGCCTCTGCTGAAGGAGATTTAAGTGGTACTGATGATTCCTTGAGAACATC
AACTACTGGTTGGGTAAACTTGCTCGGATCCTGACTGAAGTCTTGCTTGTTAGCCGAAGCAGCGTAGGAA
TCCTTGTAATAGTTTATGGTGGTGTAGTGAATGGTAGATCCACCTGTGGCCACATTGGTGTTTTCGTGGG
TTCCAGATTGTTGGGTAGACACTTGAGCTCCCATGATGAAACTATGAAGTATTGCAAATGCTGAAGAGCA
AAGAGTAATTCACACAAAAATATATCCGGATGGCCAATCCAATCGCTATATGATGACAACTCTAGATTGT
CACCATAAGCAGCCAGGTANNNNNNNNNNNNNNNNNNNNNNNNNNNNNNNNNNNNNNNNNNNNNNNNNNN
NNNNNNNNNNNNNNNNNNNNNNNNNNNNNNNNNNNNNNNNNNNNNNNNNNNNNNNNNNNNNNNNNNNNNN
NNNNNNNNNNNNNNNNNNNNNNNNNNNNAAGTTGCYTGARSTYGBTAATAAATTCTGCCTTTTCTCTTGC
AATAGGTATGATCTTTTCTCTTATCCAATCAATGAACTTGCTAATTTTGTTCGCAATCCATTCAAGGCCA
CGAGCTGCGTTGGCCATGTCATTGAATTTCTTGAGCCAAGAGTCAGATTGTTTTTGGGCGATGGGGATGC
CTAGAATTGATGCGACCTTTGTTTTAAGCCATCTCCATGGACTACCTGTACAACCAATCAGTGCCAATGT
TGCTGTGACAGTAACCAGATCGTACTCACTCCTCACAATTATGACCAGAGCGGATATCAACTTGACCAGG
TTCTTCAGGATTTTCTCTACTACACCTTCTGATCCAACGAGTGTATCTTTGATGTTCTGGACCTCTCTGG
AGATTGACTCGGTGAAACCGGTTCCGAAAGCGTCGCCCAAGCCCTTAATGTAGTCAGTGACACCCTGTTC
CATCACTTCATCCTCTAGCCAAAGGAGGTCTCTCAGGTCAGCAAAACCCACCAACCCATCACCGCCCATG
GTAACTAAGCCAATCACACCATGGGGGCACCTAAGGATGCCACCACAATCACCTGCTACAGCGATCCCAT
TTGCTAATAGTACATGTGATTGGTATCTTTCAGGGTAGTACTCGTTTGCTTGGACGTGCACAAGACCAGG
GCCCTGGAAGGAAACTGGGTAATGTTTGTTTGCTGATTTGCAGAAATACACTCCTGTGTTGCAGTTGCAC
CTGGCGATCTTGTCACACCCATGCCCAGTGGTTCTGGAAACAAGAATGTCTCTCTGATAGTCCTCCCATT
CAAGGTTGTCCCAATCATTCTCAGTAGCCAGGTGTCTGTTGACAATCCTGTAATTACACACATACACAGC
ACCACTCTGCTGACCAAAGGCTCCAGTTGTGGTTATGTTAGCCCTGTTTACAGCCATGTGCCTGATATTA
TTGCCATTAAAGTTTGGGTAGTTCTTCAACACATACGGCTGGCTTCTCATTGGTCTAGGGACCCATGCCC
TAACGTGTTTAATTCTCATGTAAATTCTGACAGTCAATTTGGTTGTGACATTCTCAGACCCCACCACTCT
AAAAGCAAAGGTGCCAAAAAGATTGTTAGGACTGATCCCATAATTCACATCACCAGATGCAGGGTGTGTA
CCGAATGTGGGGTACCCATCATAGAACCATTGATAAGCTGAAGCTGGAGACATGAAAGGAACAGAGACCT
GAGCTGGAGGGTCAGATGCCTTGACGAACACAGAGGGGTTTGTGGCAGATTGCCACTCAAAAGAGTCCCT
TGCAGTTGGGGCAGGAGCACCTGGGGGAATATACATGTACTGAACCACCACATTGGGCCCTTGGCCTGTT
GAGTCGGTACAAACAAAAGTGAACTCAGAGTCAAACCTCATGTATGTGAAAATTTCCAGCTTCCTCCGCT
GTTGTACATAGCCCATTATGTCAATTGGCCAGTTGGTAAAACCATTTGTTGTGCTCTCTGATGTGAGTAG
AGTGGCCATCCCAACAAGGGCTGCCCTGCCAAAGAAGTTTTCCAGACTCGTTTCAGCAACACTGTGGTGG
TTGTGAACACACCTTGTCTCTATCATAGACTCATCAGAGGTAACTGATGTTGCACCCGTCTCAGCTGCCT
GTAGCGCTGGTGCAGACCCTGTCAATAAGCTGTGTGAGCTTGCACTGGTGTCAGCGCCGGTGCTAGTAGA
GGCGCCCTCAGTGTTGGTAAGAACTTTGTGTGCTGTAGCATTGATGTGAGTTTTTAGCATATCCTTGATA
TCACCTTGCAACACTGCAGTTTGAGAGATTTCGTCAGTGTCTTTACACAGCCGCATGGTGAAATTTGGTT

GAGCAGCGCCCAGAGCTATGAGGTAAGCACTGGTTGGAGCACCTGCTGGAACAACAAAGTTTGTTTGGTA
CCAAATGGTGACAATACCTGCAGTGTAATAATCAAAGTAGGTTCCTGACGCATTCGTCCTAAAATGCGTG
TTGCTAATCCAAGGAATCACCAATGTAACGGATGACTGAAGACCAAAGTCCCAGATAACATGGGTGCCAA
GCATTGCCACAGCTCTACTTGTAGGCTGCACTCCACCAGGAGGTGTGTAAGCTATCAAGAGTTTTCCAGT
GGCCATAAAACTGCCTGTAAACATGAAGGTAATTTCTAATGAACCTGACCATTGAGTGTAATATCTGCAC
AGTTGACCCACCATAGTGGACTGCCATGGACCATCTCGGCCTGGATCCACTCTAAAGGAGGCACATAGCT
GGTCGACCTCACTCTGAACAGACACTGGTATCAAAAGTCTTTCCATTTGAGATGCACTCTCCACATTGTT
TACTTCAAGTATGCTTTCAATTTGACACAGTTGTAGCAAGTTGTTTACTTCACCTGGAATGTGAATCAAG
GGGGTTGGGTGGAAGCCAGGAAGTATGGGTGCTGAAACACCATCATCTGTGGTAAGAAATTGGTTGCTGC
CAGGTTTCAACTCTACTGGCAAGCCCTGAGTAACTGCATTCCTCAGACCGGCAAACTCAGCACACAGTGG
TGCAATGGTGACTGTTATTGGCACAGCCGTTGTGGCTCCATCACTATACGCTAGTGGGGCAACGGGAATA
ACAAACAAGCCAAAGTTGCAGTGATTTAGGGCCGAATCATAAGGCACTGCATTCACATAGGGCATGATTA
TGGTGGCACAATTATTGGTCCTGAGGTTAATCCATTGGTGAGGGGCAACAAGCAATTGAGAGAAAGCTAT
CCCAGAATCTAACACATAGGGAAAGGCCAATTCTTTACCACTGGGTCCAGGTTGAGTGGCATTATACGAT
GGGTGCTTTGCAGAACCGGGTGTATTAGTTGCATCACTCCTCCCATTGGTGTATTCAGGTAAACATGCCA
CCAGGAGGGTTCCCTGGTGAAACTTGCTTGCATTGCATTGTACGTGGACAGCAAAGCCTGATCTGTATAA
GTAGTGAAACTGTGCATTCTGGCCAAAAACACCTGTCTCAGTTAAGACATCAGGAAATTTCCAATACCAC
CCCTTGCTGTTTGATTGCCACAATTTTGCTGATAGTGTGTAAAATCTGTTAACCGAAACATCTGGTCTAG
TTGGTTTGTCGACAGCAGTGGCATCTGAATCACTACAGTACTCGGGCCACTCGCCATACCCTACAATCAC
ATTAGCAGCCTCCTGTGTGGTAATGGTGGAATTTCCAACTGTCATTTGGCAGACTCTGTCGCTGTAACCA
CACGCCTCTGCTGAAGGAGATTTAAGTGGTACTGATGATTCCTTGAGAACATCAACTACTGGTTGGGTAA
ACTTGCTCGGATCCTGACTGAAGTCTTGCTTGTTAGCCGAAGCAGCGTAGGAATCCTTGTAATAGTTTAT
GGTGGTGTAGTGAATGGTAGATCCACCTGTGGCCACATTGGTGTTTTCGTGGGTTCCAGATTGTTGGGTA
GACACTTGAGCTCCCATGATGAAACTATGAAGTATTGCAAATGCTGAAGAGCAAAGAGTAATTCACACAA
AAATGTATCCGGATGGCCAATCCAATCGCTATATGATGACAACTCTAGATTGTCACCATAAGCAGCCAAG
GTAAATAAAACAGGAAACACGGACACCCAAAGTAGTCGGTTCCGCCACAGAATTACTCGTTACGACCACA
ACACCACTGGATTGTAGTGCATGGCTCCGTGGTTAGGATTAGCCGCATTCAGGGGCCGGAGGACTACTAC
CTAGCTCAATAGGCTCTTCGCACCATGTCATGAATCAAGCGTCCTGCSAGTGAGA

>Enterovirus J103
CATCTCCATAAGCAATCATGTTTAATTCATCTAAATCAATGCCCTTGAATGTTCTAATTAACAGGGTCCT
AATGATAATATTATTGATCATGCTATTGAATATACTGGTCCCAGAGCAGCCTGAGGGCATGCCACCAACT
ACGCAGTATGTTTTGTTGCGGTAGATGTGGTGGGAGTGGTTAATACCCTCAATGAGGCTGACAGCCTCAT
CGTCATAACCTAGATCTTTCAAGACCATTTCTAGTGCTCTGAACCATGCTGGGCTCAAGCTAGCGTCATA
ACCGGTGTAGTCAAAAGCAAACAAACTGCCTGGAAGAAGGATGGGGATCTTACTCCAGAAGGTATCAGGG
TTTGCTCCTACTGCAGAACCAGTAATGGTGCCTGGATTCTCATGGAATTTTTCAAACAGGTGTCCAAAGC
ACATCCTCATGTATACTGAATCATTGAGACTTGATGCTTCAATCAGTCTGGATTTTCCTTTCTTCACTTT
CTCCCTGCTTCTTAACTCATCCTTGATATATGTGGAGAAGGGCAGGTCTAATCCATACTTATCCATGCAC
TCTTTTAGTTTGGTCAGGTCTCTTGTCTTCTTTGACAATATGTCTCTCTTTTTGATCCCCATTGTGACAT
AGGGGTATCCAGCACTAGTTGTTAGGTCAAGTGCTTCAAGTCCTTCAGTTCCATAGACAGCATCCTCAAG
GCACATCTCACTGGTATCAATGTCAAGTGTCATCAGTTGCCCGGCATAGTGGTCAATGGCCTCCTGCATG
TACTCATCTACATGGGTGTTCACATTACCCACATACTTGGAAAAGATGGCTTCTTCAAAGTCCACTTTGA
GTCTGGGGTCATTCTTATTCAGGACTGCTGGTTCTTTAACACCGGGAAAGATGTCATGAAACACACTAGG
TTCCAGCTTTGTTTTGCTCGGTGCATTGATGATTGGGTACCCAGACTCCTTGTTTGTCTCCATCCACTCG
ATCTCACCCTGCTCTTCGTTGAAGTAATCTCTGAGTAGCGTGGCTGCGAAACCTTGATGTCCATTACCAC
CAACATGAATGCCTATCACCTTGCCGGTGGCCATGAGCACACCACCACACTGTCCTGACTTGGTGGGGAA
GTTGTACATGAGCATTCTGTGGGTGGGTGTCCCACTGAGGTTGAGAAAACCATACTCTTTGACTGCTCCT
ACTGGGATGTACATGTTTGGGAACTTGCTAGTGTTGATTGCAAGTACTGCTTCTGACAGTTCACCACTCT
CCTTGGGAATGAAGCCCCTGATGTCTCTAAACTTTTCATTCCTGTCTAGCTTGATCATGGTCAGCTCCAG
GTTCACTCCCTCAGGATCCACCAGCTCCTTGGCGTCAAGGACTCTGAGCTCCTTGTCATCGACAATCACA

GTGTCTCCAGGTTTAGCGTGGCGTGGTACCACTGCCCATTTGTCCCAGATGCCAAGCATGGTGAATTCTC
CCTGTCCTGTTTTGATTATAGCTGAGTTTCTCTTCATCATTGCCACAGCAAATTCAAAATTGGGTCCCTG
GACTTTTGCTTGTCTGATGGTGGGTACCTTTGGTTTGTTGAATGGTAGCCCAGTGTACGCACCCTGGAAT
CCCGCGAAGAGCTTGTAAACTATGTAAATGCAACCAGCAACTGCCACAAACGTTGAAACAGCCTGAAGGA
TAGTTAGAGCCCTAGAGAGGTGTCTCTCGGTCTGGCATTCTGTGACTGGTATTATCCACCCTTTGCTTTT
ACAATACTCCCTGACCTCCTCACTGTCCACTGATTTTAGTAGGTCTGCAATGGCTGGTGGTGCTGGGGCT
TCGGGGTCCACAGAGATCTTCAGTTCCCTAAACACTGGGGGTCCCTGGAAAAGAGCTTCCAAGACATTTC
CAATGCTGTTGCGGTGGTTGTATTCTCTAAACATCTCTGTCACAAGCATATCAAGTGTGTACCTAACATT
TGTCCTTCTGTCAAGGAATTGTATTGCCTTACCACATACAAGGGGACAGCAGTGTTTGAAGTTTGCTGGG
TCACAGTTACTGCAGTGTTGGCACGCCAACTGCATGTTCAGCTTGTTGTTTAGCTTGTAGCTGTCGTTTA
TCACAATGTCAACATCCAGGTAAAATCTCCTATTAAGTGCTCTGCTGTCTGAGATTGTTGGTGCAGTGAT
GGACCCATGGTTGGTGGAGGCAATCATGAAGGGTGATGTGTAGAGTATGCCTTTCTCCTCAAGACTAGCC
ATTGGTGGGATGAAATCTACAGTAGACACCATTTGACATAACAGGCTAACATCCTTTCCATCTGGGTTCT
GACAAAGATCATCCATTATGACGACAGCTTGTTGTTTGTAGCCATCAAAGTGGTCAGGATCTGGTGGGAG
AGAGTACACACAGCTGTGTAACTTTTCAGCAATAGCGCGTCCAATGAGGCTGGATGCAATTGATTTACCA
GCACCAGGAGTTCCATGAATCAACAAACATACAGGTTCAATGCGGGATTTGGTCTTGAACTGCATGTAAT
TGTTCATTTTCTTTTCTAATGCAAACACTCTTCTAGCCTCTGCAGCGTACAAGGGAGCATACCTCCTGCA
ATAGTGTGCCAGGTACTGCACGTTTCCGTGTAATTTTTCTTGGTCATCTTGGCTTGGTGCACTGTGTTCA
ATGGAAGTCACTTGGGATTCAATGAGGGGCAACTGTTTGAGTTTGTCCAGGAATTGCACCTTCTCTTTGA
CTTCAGGCACTATTTTAGTTTTGAGCCAATCAATGAACTTGCTAATTTTCTGGGCAATCCATTCAAGTCC
TTTTGCGGCATTAGTTGCTTCAGTRAACTTCTTCAACCATGAATCACCCTGCTTYYCTACAGNNNNNNNN
NNNNNNNNNNNNNNNNNNNNNNNNNNNNNNNNNNNNNNNNNNNNNNNNNNNNNNNNNNNNNNNNNNNNNN
NNNNNNNNNNNNNNNNNNNNNNNNNNNNNNNNNNNNNNNNNNNNNNNNNNNNNNNNNNNNNNNNNNNNNN
NNNNNNNNNNNNNNGACTTGAAGCACTTGACCTAACAACTAGTGCTGGATACCCCTATGTCACAATGGGG
ATCAAAAAGAGAGACATATTGTCAAMTACCACTAGAGATGTCACTAAAATGAAGTACTACATGGATAAGT
ATGGATTAGACCTGCCCTTCTCCACATATATCAAGGATGAGTTGAGAAGCAGGGAGAAAGTKGAGCAGGG
AAAATCTCGCCTGATTGAAGCATCCAGTCTCAATGATTCTGTAGCAATGAGACAGTGTTTTGGAAACCTA
TATAAGACTTTCCACCAGAACCCAGGTACGGTTACTGGTAGTGCGGTGGGCTGCAACCCGGACACATTCT
GGAGCAAAATTCCAGTCATGTTGGATGGTGAGCTCTTCGCATTTGATTACACTGGCTACGATGCAAGCTT
GAGCCCAGTTTGGTTCACGTGTCTAAGCCGTGTGCTTGAGAAATTGGGTTATGACTACAAAGCCGGTAGA
TACATCTCTTACCTATGCCACTCATACCACCTCTACAAAAACAAGCACTACTTTGTGCGTGGTGGCATGC
CTTCTGGCTGTTCTGGTACATCAATCTTCAACTCCATGATAAACAACATCATCATTCGTACACTGTTGTT
GAAAACATATAAGAACATTGATTTGGACCAATTCAGAATGATTGCATATGGAGATGATGTGATTGCCAGT
TACCCCTACCCAATTGATGCAGGCCTGCTTGCTAAGGCGGGAAAGGAGTATGGCCTGACAATGACTCCAG
CTGATAAATCTAAGGAGTTCAACAATGTGACATGGGAAAACGTCACATTTCTGAAACGGTACTTTCGTGC
CGATGAACAGTACCCCTTCCTGGTTCATCCAGTAACCCCCATGAAGGAGATACATGAGTCCATTAGATGG
ACTAAAGATGCAAAGAATACGCAGGAGCACGTCAGATCATNNNNNNNNNNNNNNNNNNNNNNNNNNNNNN
NNNNNNNNNNNNNNNNNNNNNNNNNNNNNNNNNNNNNNNNNNNNNNNNNNNNNNNNNNNNNNNNNNNNNN
NNNNNNNNNNNNNNNNNNNNNNNNNNNNNNNNNNNNNNTACAGAATAGTGAACAGACATCTTGCAAATGA
ATTTGATGCACAGTCCACTGTGTGGGAGAGTTATGAGAGAGACCTGCTCGTTTCAACAACCACTGCGCAC
GGCTGCGACAACATTGCTAGGTGCAGATGCAACACAGGTGTGTATTTCTGTCGCAGCAAGGGAAAGCACT
ATCCAGTGTGTTTCCAAGGACCTGGTCTGACATGGATTGAGGCAAATGAGTACTACCCTGCCAGATACCA
ATCCCATGTGTTGCTTGCTTCAGGGCCAGCAGAGCCTGGTGATTGTGGGGGCATTCTTAGATGTCAACAT
GGTGTGATTGGATTGGTCACAGCAGGGGGCAATGGATTAGTGGCTTTTGCTGACCTGCGTGATCTCTTGT
GGTTAGAAGATGATGCCATGGAACAGGGAATCACTGATTACGTGACTCAGCTTGGACAAGCTTTCGGCAG
TGGATTCACCAATGAAATTGCAGAGAAAACTCAGCAGATCAAGGATATGCTACTTGGACAGGATTCCATT
TTGGAGAAATGCTTGAAAGCCCTCATTAAGATCGTTAGTGCCTTAGTCATCATTGTGAGGAATCACGAAG
ATCTCATTACAGTTACTGCTACACTTGCACTAATTGGATGCTCTGGATCCCCATGGCAATGGCTCAAAAC
TAAGGTTTGCNNNNNNNNNNNNNNNNNNNNNNNNNNNNNNNNNNNNNNNNNNNNNNNNNNNNNNNNNNNN
NNNNNNNNNNNNNNNNNNNNNNNNNNNNNNNNNNNNNNNNNNNNNNNNNNNNNNNNNNNNNNNNNNNNNN

NNNNNNNNNNNNNNNNNNNNNNNNNNNNNNNNNNNNNNNNNNNNNNNNNNNNNNNNNNNNNNNNNNNNNA
GGTGGAACTAACACATACATGGAGTATGCTCAGACCATGCCAGGTGAGTCTGGGGGCGTTTTCAACCACC
CCGAGTGGTTGGACAATGGAACAATCATCTCTCAAGCGCTCTTATACCCACACCAGTGGATAAACCTGCG
CACAAACAACAGCGCAACTTTAATCCTACCATACATGAATAATATCCCAGCAGACTCGCCAATAAAACAC
AACAATTGGACAGTGGTTGTGATTCCTGTTTGCAAGTTAGAGTTCGCGTCAGGAGCGACAGGGGTGGTCC
CAATAACAGTCAGCATTGCTCCGTTATGTGCAGAGTTTGATGGGCTTAGGCAAGCAGTATCACAAGGATT
GCCTGTAAAATTAGTACCAGGTTCCAACCAGTTCATGACCACATCGGACGACTGTGTTGAAGGCATCCTT
CCTGATTTCCATTCCACGCCAGAAATCAAAATCCCAGGTGAGGTGAAAAATTTGCTGGAGTGTGCACAGG
TTGAGACCATGCTGATGGTTAACAATGTCGTAGGCGCAACAGGACTTGAGAGGTTCACCATTCCAGTATC
TACTCAATCAGATGTGGACGGCCAGATAATGGCACTTAGGGTAGACCCAGGAAGTGATGGCCCGTGGCAA
GCCACATACCTCGGACAGCTCTGTAGGTACTACAACAACTGGTCTGGCTCCCTAGAAATCACTTTCATGT
TTTGTGGATCTTTCATGGCCACTGGCAAAGTATTGATTGCTTACTCACCACCAGGAGGTTCAGCGCCCAC
TACGCGCAAGGATGCTATGTTAGGCACACACGTTGTTTGGGATTTTGGGCTGCAGTCATCCATCACCCTT
AGTATTCCATGGATTTCGAACACATGCATGCGCAATGTGGCGAGCATAGCCTACCAGGAGTACTTCGCAG
CAGGTTATGTCACAATGTGGTACCAAACAAACTTTGTTGTCCCCTCAGGGGCACCCACAAGTGCTCGGAT
CATTGCGCTGGTCGCAGCTAGACCCAATTTTGTGTTGCGATTGCTAAAGGACAGTGATCAGATCTCGCAG
ACTGCTGTGTTGAACGGTCCTGTAGAAGAAGTGATTAATAATGCTGTCACCAAGCTAGTTGGCACAGCAG
CTGACACCATAGCATCTGGTCCACACCAGGGAGACGCTGTACCAGCACTACAAGCAGCTGAGACAGGTGC
CACAAGCACAGCTGATGATAGTGGAATGATCGAAACTCGCACTGTCTATAACCACAATTCCATTGCTGAA
ACAGGAGTGGARGCATTTTATTCAAGAGCAGGTCTTGTTGGAATGGTGACCCTACTGAGATCAGGTGATC
AAGACACAAATACGTTTACTAACTGGGTCATAGACACAATGGGGTATGTCCAGCAGAGAAGGAAGTTGGA
GCTGTTCACATACATGAGGTTCGATGCAGAATTTACCTTGGTGATCACCCAGGACAATGCAGCAGAATTT
CCCCAGTTAATGGTGCAATATATGTACATCCCACCGGGCAGCCCAGTGCCTAATTCAGCAGATTCTTACC
TTTGGCAATCTGGTACCAACCCCTCAGTATTTGTAAAAACAACTGACCCACCAGCTCAGTTCTCCATTCC
CTTCATGTCAACATGTGCTGCCTACGCATGGTTCTATGACGGTTACCCGTCGTTTGGRAGACCCGTCGAG
TCGGGTGGTAAAGGCAGCGCTTCATATGGTGTGTTACCGCCCAACATGTTTGGCACACTGGCATTTAGAA
TGGTAGGRTCATCTCAGAACACCAACCTGAGAATTCGCATTTACATGAGGCCCAAACATGTAAAGTGCTG
GGGCCNNNNNNNNNNNNNNNNNNNNNNNNNNNNNNNNNNNNNNNNNNNNNNNNNNNNNNNNNNNNNNNNN
NNNNNNNNNNNNNNNNNNNNNNNNNNNNNNNNNNNNNNNNNNNNNNNNNNNNNNNNNNNNNNNNNNNNNN
NNNNNNNNNNNNNNNNNNNNNNNNNNNNNNNNNNNNNNNNNNNNNNNNGAGCTAGGTAGTAGTCCTCCGG
CCCCTGAATGCGGCTAATCCCAACTCCGGAGCAAACGCACGCAACCCAGCGTGTAGTTTGTCGTAACGCG
CAAGTCTGGAGCGGAACCGACTACTTTGGGTGTCCGTGTTTCCTGTTTTACCTTATTTGGCTGCTTATGG
TGACAATCTAGTGTTGTTACCATATAGCTGTTGGATTGGCCATCCGGTGATTGATTATCGCTGAATTTTA
TTGCTCAGCTTTTTATAGCTCAAGCACATAAACCTCATTATGGGTGCACAAGTGTCCAGACAGCAGACTG
GTTCTCATGAGACTAACGCCATTGCCACCGGCGGTAGCACCATTCATTACACCAACATCAACTACTACAA
GGATAGTTATGCAGCTAGTGCCACCAAGCAGGACTTTAGTCAAGATCCATCTAAATTCACACAACCCGTG
GTTGACGCACTACAAGCAACCTCTGTGCCGTTGAAAAGTCCATCTGCTGAAGCATGTGGGTACAGTGACA
GAGTGTTGCAGCTGACCTATGGAAATTCAACAATAACAACCCAGGAGGCTGCAAATGTGGTGGTTGGCTA
TGGCCAATGGCCTGAACACCTGGGGCCACAAGATGCCAATTCGGTAGATGCACCAACCAAGCCAGACGTG
GCAACCAACCGCTTTTACACACTACAAGCGAAGAAGTGGGAGGCTACCTCAAAAGGGTGGTATTGGAAAT
TGCCAGACTGTTTGAATGATATGGGGGTTTTTGGTCAAAACTGTTACTACCATTTCCTTTACAGAGGTGG
CTGGGCAATTCATGTCCAGTGTAACGCCAGTAAGTTCCATCAGGGCACGCTCTTGGTTGTTGCGATACCC
GAGTGGACGCAAGGTAGGGGTCTCAACGCAGGTGGGACCAACACACACATGGAATTTAMWCAAACCATGC
CAGGTGAATCAGGGGGTGTTTTCAACCACCCTGAATGGTTGGACAATGGAACAATCATCTCTCAAGCGCT
CTTATATCCACACCAGTGGATAAACTTGCGCACAAACAACAGTGCAACTCTGATCCTGCCATACATGAAC
AACATACCAGCAGACTCACCAATAAAACACAACAACTGGACATTGGCTGTAATTCCTGTTTGCAAGCTAG
AGTTNNNNNNNNNNNNNNNNNNNNNNNNNNNNNNNNNNNNNNNNNNNNNNNNNNNNNNNNNNNNNNNNNN
NNNNNNNNNNNNNNNNNNNNNNNNNNNNNNNNNNNNNNNNNNNNNNNNNNNNNNNNNNNNNNNNNNNNNG
ATCTGACGTGCTCCTGCGTATTCTTTGCATCTTTAGTCCATCTAATGGACCACTGGAGGATATTCATGGG
CATTACTGGATGAACCAGGAAGGGGTACTGTTCATCGGCACGAAAGTACCGTTTCAGAAATGTGACGTTT

TCCCATGTCACATTGTTGAACTCCTTAGATTTATCAGCTGGAGTCATTGTCAGGCCATACTCCTTTCCCG
CCTTAGCAAGCAGGCCTGCATCAATTGGGTAGGGGTAACTGGCAATCACATCATCTCCATATGCAATCAT
TCTGAATTGGTCCAAATCAATGTTCTTGTATGTTTTCAACAACAGTGTACGAATGATGATGTTGTTTATC
ATGGAGTTGAAGATTGATGTACCAGAACAGCCAGAAGGCATGCCACCACGCACAAAGTAGTGCTTGTTTT
TGTAGAGGTGGTATGAGTGGCATAGGTAAGAGATGTATCTACCGGCTTTGTAGTCATAACCCAATTTCTC
AAGCACACGGCTTAGACACGTGAACCAAACTGGGCTCAAGCTTGCATCGTAGCCAGTGTAATCAAATGCG
AAGAGCTCACCATCCAACATGACTGGAATTTTGCTCCAGAATGTGTCCGGGTTGCAGCCCACCGCACTAC
CAGTAACCGTACCTGGGTTCTGGTGGAAAGTCTTATATAGGTTTCCAAAACACTGTCTCATTGCTACAGA
ATCATTGAGACTGGAKGCTTCAATCARGCGAGATTTTCCYTKCTYRAYYTTCTCMMTGCTYCTCARYTCA
TCYTTCACATAKGTCACCATTGGTAGGTTGAGACCGTACTTGTCCATGCACTCTTTTAGTTTGGTCAGGT
CTCTTGTCTTCTTTGACAATATGTCTCTCTTTTTGATCCCCATTGTGACATAGGGGTATCCAGCACTAGT
TGTTAGGTCAAGTGCTTCAAGTCCTTCAGTTCCATAGACAGCATCCTCAAGGCACATCTCACTGGTATCA
ATGTCAAGTGTCATCAGTTGCCCGGCATAGTGGTCAATGGCCTCCTGCATGTACTCATCTACATGGGTGT
TCACATTACCCACATACTTGGAAAAGATGGCTTCTTCAAAGTCCACTTTGAGTCTGGGGTCATTCTTATT
CAGGACTGCTGGTTCTTTAACACCGGGAAAGATGTCATGAAACACACTAGGTTCCAGCTTTGTTTTGCTC
GGTGCATTGATGATTGGGTACCCAGACTCCTTGTTTGTCTCCATCCACTCGATCTCACCCTGCTCTTCGT
TGAAGTAATCTCTGAGTAGCGTGGCTGCGAAACCTTGATGTCCATTACCACCAACATGAATGCCTATCAC
CTTGCCGGTGGCCATGAGCACACCACCACACTGTCCTGACTTAGTGGGGAAGTTGTACATGAGCATTCTG
TGGGTGGGTGTCCCACTGAGGTTGAGAAAACCATACTCTTTGACTGCTCCTACTGGGATGTACATGTTTG
GGAACTTGCTAGTGTTGATTGCAAGCACTGCTTCTGACAGTTCTCCACTCTCTTTGGGAATGAAGCCCCT
GATGTCTCTAAACTTTTCATTCCTGTCTAGCTTGATCATGGTCAGCTCCAGGTTCACTCCCTCAGGATCC
ACCAGCTCCTTGGCGTCAAGGACTCTGAGCTCCTTGTCATCGACAATCACAGTGTCTCCAGGTTTAGCGT
GGCGTGGTACCACTGCCCATTTGTCCCAGATGCCANNNNNNNNNNNNNNNNNNNNNNNNNNNNNNNNNNN
NNNNNNNNNNNNNNNNNNNNNNNNNNNNNNNNNNNNNNNNNNNNNNNNNNNNNNNNNNNNNNNNNNNNNN
NNNNNNNNNNNNNNNNNNNNNNNNNNNNNNNNNNNNNNNNNNNNNNNNNNNNAGAGGTGGTATGAGTGGCAT
AGGTAAGAGATGTATCTACCGGCTTTGTGGTCATAACCCAATTTCTCAAGCACACGGCTTAGACACGTGA
ACCAAACTGGGCTCAAGCTTGCATCGTAGCCAGTGTAATCAAATGCGAAGAGCTCACCATCCAACATGAC
TGGAATTTTGCTCCAGAATGTGTCCGGGTTGCAGCCCACCGCACTACCAGTAACCGTACCTGGGTTCTGG
TGGAAAGTCTTATATAGGTTTCCAAAACACTGTCTCATTGCTACAGAATCATTGAGGCTGGAGGCTTCAA
TCAGGCGAGATTTTCCCTGCTCAACTTTCTCCCTGCTTCTCAACTCATCCTTCACATATGTCACCATTGG
TAGGTTGAGACCGTACTTGTCCATGCACTCTTTTAGTTTGGTCAGGTCTCTTGTCTTCTTTGACAATATG
TCTCTCTTTTTGATCCCCATTGTGACATAGGGGTATCCAGCACTAGTTGTTAGGTCAAGTGCTTCAAGTC
CTTCAGTTCCATAGACAGCATCCTCAAGGCACATCTCACTGGTATCAATGTCAAGTATCATCAGTTGCCC
AGCATAGTGGTCAATGGCCTCCTGCATGTACTCATCTACGTGGGTGTTCACGTTACCCACATACTTGGAA
AAGATGGCTTCTTCAAAGTCCACTTTGAGTCTGGGGTCATTCTTATTCAGGACTGCTGGTTCTTTAACAC
CGGGAAAGATGTCATGAAACACACTAGGTTCCAGCTTTGTTTTGCTCGGTGCATTGATGATTGGGTACCC
AGACTCCTTGTTTGTCTCCATCCACTCGATCTCACCCTGCTCTTCATTGAAGTAATCTCTGAGTAACGTG
GCTGCGAAACCTTGATGTCCATTACCACCGACATGAATGCCTATCACCTTGCCGGTGGCCATGAGCACAC
CACCACACTGTCCTGACTTGGTGGGGAAGTTGTACATGAGCATTCTGTGGGTGGGTGTCCCACTGAGGTT
GAGAAAACCATACTCTTTGACTGCTCCTACTGGGATGTACATGTTTGGGAACTTGCTAGTGTTGATTGCA
AGTACTGCTTCTGACAGTTCACCACTCTCCTTGGGAATGAAGCCCCTGATGTCTCTAAACTTTTCATTCC
TGTCTAGCTTGATCATGGTCAGCTCCAGGTTCACTCCCTCAGGATCCACCAGCTCCTTGGCGTCAAGGAC
TCTGAGCTCCTTGTCATCGACAATCACAGTGTCTCCAGGTTTAGCGTGGCGTGGTACCACTGCCCATTTG
TCCCAGATGCCAAGCATGGTGAATTCTCCCTGTCCTGTTTTGATTATAGCTGAGTTTCTCTTCATCATTG
CCACAGCAAATTCAAAATTGGGTCCCTGGACCTTTGCTTGTCTGATGGTGGGTACCTTTGGTTTGTTGAA
CGGTAGCCCAGTGTACGCACCCTGGAATCCTGCGAAAAKCTTGTAAACTATGTAAATGCAACCAGCAACT
GCCACAAACGTTGAAACAGCCTGAAGGATAGTTAGAGCCCTAGAGAGGTGTCTCTCGGTCTGGCATTCTG
TGACTGGAATTATCCACCCTTTGCTTTTACAATACTCCCTGACCTCCTCACTGTCCACTGATTTTAGTAG
GKSHBACHTGGCTGGTGGTGCTGGGGCTTCGGGGTCCACAGAGATCTTCAGTTCCCTAAAYASTGGGGGT
CCCTGGAAAAGAGCTTCCAAGACATTTCCAATGCTGTTGCGGTGGTTGTATTCTCTAAACATCTCTGTCA

CAAGCATATCAAGTGTGTACCTAACATTTGTCCTTCTGTCAAGGAATTGTATTGCCTTACCACATACAAG
GGGACAGCAGTGTTTGAAGTTTGCTGGGTCACAGTTACTGCAGTGTTGGCACGCCAACTGCATGTTCAGC
TTGTTGTTTAGCTTGTAGCTGTCGTTTATCACAATGTCAACATCCAGGTAAAATCTCCTATTAAGAGCTC
TGCTGTCTGAGATTGTTGGTGCAGTGATGGACCCATGGTTGGTAGAGGCAATCATAAAGGGTGATGTGTA
GAGTATGCCTTTCTCCTCAAGACTAGCCATTGGTGGGATGAAATCCACAGTAGACACCATTTGACATAAC
AGGCTAACATCCTTTCCATCTGGGTTCTGACAAAGATCATCCATTATGACGACAGCTTGTTGTTTGTAGC
CATCAAAGTGGTCAGGATCTGGTGGGAGAGAGTACACACAGCTGTGTAACTTTTCAGCAATAGCGCGTCC
AATGAGGCTGGATGCAATTGATTTACCAGCACCAGGAGTTCCATGAATCAACAAACATACAGGTTCAATG
CGGGATTTGGTCTTGAACTGCATGTAATTGTTCATTTTCTTTTCTAATGCAAACACTCTTCTAGCCTCTG
CAGCGTACAAGGGAGCATACCTCCTGCAATAGTGTGCCAGGTACTGCACGTTTCCGTGTAATTTTTCTTG
GTCATCTTGGCTTGGTGCACTGTGTTCAATGGAAGTCACTTGGGATTCAATGAGGGGCAACTGTTTGAGT
TTGTCCAGGAATTGCACCTTCTCTTTGACTTCGGGCACTATTTTAGTTTTGAGCCAATCAATGAACTTGC
TAATTTTCTGGGCAATCCACTCAAGTCCTTTCGCAGCATTGGTTGCTTCAGTAAACTTCTTCAACCACGA
ATCGCCCTGCTTCTCTACCATAGGTATTTGAAGATACTGGCAAACCTTAGTTTTGAGCCATTGCCATGGG
GATCCAGAGCATCCAATTAGTGCAAGTGTAGCAGTAACTGTAATGAGATCTTCGTGATTCCTCACAATGA
TGACTAAGGCACTAACGATCTTAATGAGGGCTTTCAAGCATTTCTCCAAAATGGAATCCTGTCCAAGTAG
CATATCCTTGATCTGCTGAGTTTTCTCTGCAATTTCATTGGTGAATCCACTGCCGAAAGCTTGTCCAAGC
TGAGTCACGTAATCAGTGATTCCCTGTTCCATGGCATCATCTTCTAACCACAAGAGATCACGCAGGTCAG
CAAAAGCCACTAATCCATTGCCCCCTGCTGTGACCAATCCAATCACACCATGTTGACATCTAAGAATGCC
CCCACAATCAYCAGGYWCTGCTGGCCCTGAAGCAAGCAACACATGGGATTGGTATCTGGCAGGATAGTAC
TCATTTGCCTCAATCCATGTCAGACCAGGTCCTTGGAAACACACTGGATAGTGCNNNNNNNNNNNNNNNN
NNNNNNNNNNNNNNNNNNNNNNNNNNNNNNNNNNNNNNNNNNNNNNNNNNNNNNNNNNNNNNNNNNNNNN
NNNNNNNNNNNNNNNNNNNNNNNNNNNNNNNNNNNNNNNNNNNNNNNNNNNNNNNNNNNNNNNNNNNNNN
NNNNNNNNNNNNNNNNNNNNNNNNNNNNNNNNNNNNATGAGACATTCTGGCTCCTTTGTGCAAAAGGTACC
CGCCTTGCAAAYSAMTGRRGGATATCGAACAAGCACTTCTGTTACCCCGGTCTTAGTACCTGTGAAGCTG
TACCCACGGCGGAAAGGGAAAAGGATCGTTATCCGGCTATCTACTTCGAGAAGCCTAGTAACAACATAGA
AACTCTTAAGCGTTGCGCTCAGCACAGTACCCCCTGTGTAGCTTTGGCTGATGAGTCTGGGCATTCCCCA
CTGGCGACAGTGGCCCAGGCTGCGTTGGCGGCCAACCGATCCGGGCAACCGGGTCGGACGCTTACTTGTG
GACATGGTGTGAAGAGCCTATTGAGCTAGGTAGTATTCCTCCGGCCCCTGAATGCGGCTAATCCTAACTC
CGGAGCAAATGCATGCAATCCAGCGTGTAGTTTGTCGTAATGCGCAAGTCTGGAGCGGAACCGACTACTT
TGGGTGTCCGTGTTTCCTGTTTTACCTTATCTGGCTGCTTATGGTGACAATCTAGTGTTGTTACCATATA
GCTATTGGATTGGCCATCCGGTGAATAGAATTTGATAAACCTCGTTGAGTAGCTTCTATAGCTTAAACAC
ACAAACTTCATTCTAAACTAGTAGTAGTCATGGGTGCACAAGTGTCCAGACAACAGACTGGTTCTCATGA
AACGAATGCCATCGCTACTGGCGGTAGCACTATCCATTACACCAACATTAATTACTACAAGGATAGTTAT
GCAGCTAGTGCTACCAAACAAGACTTTAGTCAAGATCCATCCAAATTCACGCAACCCGTGGTTGACGCAC
TACAAGCAACCGCCGTGCCGTTGAAAAGTCCATCTGCTGAAGCATGCGGGTACAGTGACAGAGTGTTGCA
GCTGACCTATGGAAATTCAACAATAACAACCCAGGAGGCTGCAAATGTGGTAGTTGGCTATGGCCAGTGG
CCTGAACACCTGGGGCCACAAGACGCCAATTCGGTAGATGCACCAACCAAGCCAGATGTGGCAACTAACC
GCTTCTACACGCTACAAGCAAAGAAGTGGGAGACTAACTCAAAAGGGTGGTATTGGAAGTTACCAGACTG
TCTCAATGACATGGGCGTCTTTGGTCAAAATTGTTACTACCACTTCCTATACAGAGGTGGCTGGGCGATT
CACGTCCAGTGTAACGCCAGCAAATTTCATCAGGGCACACTTTTGGTTGTTGCAATCCCCGAGTGGACTC
AAGGTAGGGGTCTCAACGCAGGTGGAACTAACACATACATGGAGTATGCTCAGACCATGCCAGGTGAGTC
TGGGGGCGTTTTCAACCACCCCGAGTGGTTGGACAATGGAACAATCATCTCTCAAGCGCTCTTATACCCA
CACCAGTGGATAAACCTGCGCACAAACAACAGCGCAACTTTAATCCTACCATACATGAATAATATCCCAG
CAGACTCGCCAATAAAACACAACAATTGGACAGTGGTTGTGATTCCTGTTTGCAAGTTAGAGTTCGCGTC
AGGAGCGACAGGGGTGGTCCCAATAACAGTCAGCATTGCTCCGTTATGTGCAGAGTTTGATGGGCTTAGG
CAAGCAGTATCACAAGGATTGCCTGTAAAATTAGTACCAGGTTCCAACCAGTTCATGACCACATCGGACG
ACTGTGTTGAAGGCATCCTTCCTGATTTCCATTCCACGCCAGAAATCAAAATCCCAGGTGAGGTGAAAAA
TTTGCTGGAGTGTGCACAGGTTGAGACCATGCTGATGGTTAACAATGTCGTAGGCGCAACAGGACTTGAG
AGGTTCACCATTCCAGTATCTACTCAATCAGATGTGGACGGCCAGATAATGGCACTTAGGGTAGACCCAG

GAAGTGATGGCCCGTGGCAAGCCACATACCTCGGACAGCTCTGTAGGTACTACAACAACTGGTCTGGCTC
CCTAGAAATCACTTTCATGTTTTGTGGATCTTTCATGGCCACTGGCAAAGTATTGATTGCTTACTCACCA
CCAGGTGGTTCAGCACCCACAACACGCAAGGATGCTATGTTAGGCACGCACGTTGTTTGGGACTTCGGAT
TACAGTCATCCATCACCCTTAGTATTCCGTGGATTTCAAACACGTGTATGCGCAACGTGGCAAGTTTAGC
CTACCAAGAATACTTTGCAGCAGGGTATGTTACAATGTGGTATCAAACAAACTTTGTTGTCCCTTCAGGG
GCACCCACGAGTGCCCGAATTGTAGCACTTGCTGCGGCTAGACCCAACTTTGTGTTACGGTTGTTGAAGG
ATAGTGATCAGATCTCGCAGACTGCTGTGTTGAACGGTCCTGTAGAAGAAGTGATTAATAATGCTGTCAC
CAAGCTAGTTGGCACAGCAGCTGACACCATAGCATCAGGACCACACCAGGGAGATGCTGTACCAGCGCTA
CAAGCTGCTGAGACAGGAGCTACAAGCACTGCTGATGATAGTGGAATGATCGAAACTCGCACTGTCTATA
ACCACAATTCCATTGCTGAAACAGGAGTGGAAGCATTTTATTCAAGAGCAGGTCTTGTTGGAATGGTGAC
CCTACTGAGATCAGGTGATCAAGACACAAACACATTTGCTAACTGGGTCATAGACACAATGGGGTATGTC
CAGCAAAGAAGGAAATTGGAGTTGTTCACATACATGAGGTTTGATGCTGAGTTCACCTTGGTGATTACAC
AGGATAATGCAGCAGAACTCCCCCAGTTAATGGTGCAATATATGTACATCCCACCGGGCAGCCCAGTGCC
TAATTCAGCAGACTCTTATCTTTGGCAATCCGGCACCAACCCCTCAGTGTTTGTGAAAACAACTGACCCA
CCAGCCCAGTTCTCCATCCCATTTATGTCAACATGTGCTGCTTATGCTTGGTTTTATGACGGTTACCCAT
CGTTCGGGAGACCCACTGAGGTGAAAGGTAAGGGCAGTGCTTCATATGGTGTGCTGCCACCCAATATGTT
TGGTACACTAGCCTTTAGAATGGTGGGGTCGTCACAGAAAACCAACCTGAGAATTCGCATATACATGAAA
CCCAAACATGTGAAGTGCTGGGGCCCGAGACCCTTTCGGATGTTGCCATACATGGAGAAGAACAGACCAT
CCTACAATGGTACGGTGAAACCCATTAGAGATAGGGCCTCAATTACCACAGTGGGCAAGTTTGGTCAACA
GAGTGGTGCAGTTTATGTGGGTAACTACAGAATAGTGAACAGACATCTTGCAAATGAATTTGATGCACAG
TCCACTGTGTGGGAGAGCTATGAAAGAGACCTACTTATTTCAACCACCACCGCACATGGCTGTGACACCA
TTGCCAGGTGCAAATGCAACACAGGTGTGTATTTCTGTCGTAGCAGGGGCAARCAYTATCCAGTGTGCTT
CCAAGGACCTGGTCTGACATGGATTGAGGCAAATGAGTACTACCCTGCCAGATACCAATCCCATGTAYTA
CTCGCYTCAGGRCCAGCAGAACCCGGTGATTGTGGGGGCATTCTTAGATGTCAACATGGTGTGATTGGAT
TGGTCACAGCAGGGGGCAATGGATTAGTGGCTTTTGCTGACCTGCGTGATCTCTTGTGGTTAGAAGATGA
TGCCATGGAACAGGGAATCACTGATTACGTGACTCAGCTTGGACAAGCTTTCGGCAGTGGATTCACCAAT
GAAATTGCAGAGAAAACTCAGCAGATCAAGGATATGCTACTTGGACAGGATTCCATTTTGGAGAAATGCT
TGAAAGCCCTCATTAAGATCGTTAGTGCCTTAGTCATCATTGTGAGGAATCACGAAGATCTCATTACAGT
TACTGCTACACTTGCACTAATTGGATGCTCTGGATCCCCATGGCAATGGCTCAAAACTAAGGTTTGCCAG
TATCTTCAAATACCTATGGTAGAGAAGCAGGGCGATTCGTGGTTGAAGAAGTTTACTGAAGCAACCAATG
CTGCGAAAGGACTTGAGTGGATTGCCCAGAAAATTAGCAAGTTCATTGATTGGCTCAAAACTAAAATAGT
GCCCGAAGTCAAAGAGAAGGTGCAATTCCTGGACAAACTCAAACAGTTGCCCCTCATTGAATCCCAAGTG
ACTTCCATTGAACACAGTGCACCAAGCCAAGATGACCAAGAAAAATTACACGGAAACGTGCAGTACCTGG
CACACTATTGCAGGAGGTATGCTCCCTTGTACGCTGCAGAGGCTAGAAGAGTGTTTGCATTAGAAAAGAA
AATGAACAATTACATGCAGTTCAAGACCAAATCCCGCATTGAACCTGTATGTTTGTTGATTCATGGAACT
CCTGGTGCTGGTAAATCAATTGCATCCAGCCTCATTGGACGCGCTATTGCTGAAAAGTTACACAGCTGTG
TGTACTCTCTCCCACCAGATCCTGACCACTTTGATGGCTACAAACAACAAGCTGTCGTCATAATGGATGA
TCTTTGTCAGAACCCAGATGGAAAGGATGTTAGCCTGTTATGTCAAATGGTGTCTACTGTGGATTTCATC
CCACCAATGGCTAGTCTTGAGGAGAAAGGCATACTCTACACATCACCCTTTATGATTGCCTCTACCAACC
ATGGGTCCATCACTGCACCAACAATCTCAGACAGCAGAGCTCTTAATAGGAGATTTTACCTGGATGTTGA
CATTGTGATAAACGACAGCTACAAGCTAAACAACAAGCTGAACATGCAGTTGGCGTGCCAACACTGCAGT
AACTGTGACCCAGCAAACTTCAAACACTGCTGTCCCCTTGTATGTGGTAAGGCAATACAATTCCTTGACA
GAAGGACAAATGTTAGGTACACACTTGATATGCTTGTGACAGAGATGTTTAGAGAATACAACCACCGCAA
CAGCATTGGAAATGTCTTGGAAGCTCTTTTCCAGGGACCCCCAGTGTTTAGGGAACTGAAGATCTCTGTG
GACCCCGAAGCCCCAGCACCACCAGCCATTGCAGACCTACTAAAATCAGTGGACAGTGAGGAGGTCAGGG
AGTATTGTAAAAGCAAAGGGTGGATAATTCCAGTCACAGAATGCCAGACCGAGAGACACCTCTCTAGGGC
TCTAACTATCCTTCAGGCTGTTTCAACGTTTGTGGCAGTTGCTGGTTGCATTTACATAGTTTACAAGCTT
TTCGCAGGATTCCAGGGTGCGTACACTGGGCTACCGTTCAACAAACCAAAGGTACCCACCATCAGACAAG
CAAAGGTCCAGGGACCCAATTTTGAATTTGCTGTGGCAATGATGAAGAGAAACTCAGCTATAATCAAAAC
AGGACAGGGAGAATTCACCATGCTTGGCATCTGGGACAAATGGGCAGTGGTACCACGCCACGCTAAACCT

GGAGACACTGTGATTGTCGATGACAAGGAGCTCAGAGTCCTTGACGCCAAGGAGCTGGTGGATCCTGAGG
GAGTGAACCTGGAGCTGACCATGATCAAGCTAGACAGGAATGAAAAGTTTAGAGACATCAGGGGCTTCAT
TCCCAAGGAGAGTGGTGAACTGTCAGAAGCAGTACTTGCAATCAACACTAGCAAGTTCCCAAACATGTAC
ATCCCAGTAGGAGCAGTCAAAGAGTATGGTTTTCTCAACCTCAGTGGGACACCCACCCACAGAATGCTCA
TGTACAACTTCCCCACCAAGTCAGGACAGTGCGGTGGTGTGCTCATGGCCACCGGCAAGGTGATAGGCAT
TCATGTCGGTGGTAATGGACATCAAGGTTTCGCAGCCACGTTACTCAGAGATTACTTCAATGAAGAGCAG
GGTGAGATCGAGTGGATGGAGACAAACAAGGAGTCTGGGTACCCAATCATCAATGCACCGAGCAAAACAA
AGCTGGAACCTAGTGTGTTTCATGACATCTTTCCCGGTGTTAAAGAACCAGCAGTCCTGAATAAGAATGA
CCCCAGACTCAAAGTGGACTTTGAAGAAGCCATCTTTTCCAAGTATGTGGGTAACGTGAACACCCACGTA
GATGAGTACATGCAGGAGGCCATTGACCACTATGCTGGGCAACTGATGATACTTGACATTGATACCAGTG
AGATGTGCCTTGAGGATGCTGTCTATGGAACTGAAGGACTTGAAGCACTTGACCTAACAACTAGTGCTGG
ATACCCCTATGTCACAATGGGGATCAAAAAGAGAGACATATTGTCAAAGAAGACAAGAGACCTGACCAAA
CTAAAAGAGTGCATGGACAAGTACGGTCTCAACCTACCAATGGTGACATATGTGAAGGATGAGTTGAGAA
GCAGGGAGAAAGTTGAGCAGGGAAAATCTCGCCTGATTGAAGCCTCCAGCCTCAATGATTCTGTAGCAAT
GAGACAGTGTTTTGGAAACCTATATAAGACTTTCCACCAGAACCCAGGTACGGTTACTGGTAGTGCGGTG
GGCTGCAACCCGGACACATTCTGGAGCAAAATTCCAGTCATGTTGGATGGTGAGCTCTTCGCATTTGATT
ACACTGGCTACGATGCAAGCTTGAGCCCAGTTTGGTTCACGTGTCTAAGCCGTGTGCTTGAGAAATTGGG
TTATGACCACAAAGCCGGTAGATACATCTCTTACCTATGCCACTCATACCACCTCTACAAAAACAAGCAC
TACTTTGTGCGTGGTGGCATGCCTTCTGGCTGTTCTGGTACATCAATCTTCAACTCCATGATAAACAACA
TCATCATTCGTACACTGTTGTTGAAAACATATAAGAACATTGATTTGGACCAATTCAGAATGATTGCATA
TGGAGATGATGTGATTGCCAGTTACCCCTACCCAATTGATGCAGGCCTGCTTGCTAAGGCGGGAAAGGAG
TACGGCCTGACAATGACTCCAGCTGATAAATCTAAGGAGTTCAACAATGTGACATGGGAAAACGTCACAT
TTCTGAAACGGTACTTTCGTGCCGATGAACAGTACCCCTTCCTGGTTCATCCAGTAATGCCCATGAAGGA
GATACATGAGTCCATTAGATGGACTAAAGATGCAAAGAATACGCAGGAGCACGTCAGATC

>Sapelovirus B
GCAATCCAGGTCGGTTTCTATCAAGCACTTCTGTTTCCCCGGACTTAGTACCAATAGGCTGTACCCACGG
CTGAAAGGGAAAACGTTCGTTACCCGGCCACTTACTTCGAGAAGCCTAGTACCATCATTGAATGTCTCAA
GTGTTACGTTCAGCACAACCCCAGTGTAGTTCAGGTCGATGAGTCACCGAATCCCCCACGGGCGACCGTG
TCGGTGGCTGCGTTGGCGGCCTGCCTGCGGGTCTTACTCGCAGGACGCTTGATTCATGACATGGTGCGAA
GAGCCTATTGAGCTAGGTGGTAGTCCTCCGGCCCCTGAATGCGGATAATCCTAACCACGGAGCCATGCAC
CACAATCCAGTGGTGTTGTGGTCGTAACGAGTAATTCTGTGGCGGAACCGACTACTTTGGGTGTCCGTGT
TTCCTGTTTTATTTACCTTGGCTGCTTATGGTGACAATCTAGAGTTGTCATCATATAGCGATTGGATTGG
CCATCCGGATATATTCTTTGTGTGATTTATTTCTTGCTGCCAAGCAACTGTCATTCGCCATCAATACAAT
GGGTGCCCAGGTTTCCACTCAGAAAACCGGTTCTCACGAGAATGCCAATCTGGCAACTGGTGGTTCTACC
ATCAATTACACCACCATAAATTACTACAAAGAGTCTTATGCTGCATCAGCAACAAAACAAGACTTCAGTC
AAGACCCATCTAAATTTACTCAACCTGTAGTTGATGTGATAAAAGAATCATCCGTCCCTCTTAAATCACC
CTCAGCGGAGGCCTGCGGATACAGTGACAGAGTGGCCCAACTTACTCTCGGAAATTCCACCATTACAACA
CAAGAAGCAGCTAACATAACGGTGGGTTATGCTGAATGGCCTGAGTTCTGCTCGGATACCGACGCAACAG
CGGTGGACAAACCAACACGCCCTGATGTCTCAGTTAACAGATTTTACACGCTCAGCGCCAAGCTGTGGAG
CAAAGAATCAAAAGGTTGGTATTGGAAGTTCCCTGACGTCTTAACAGAGACAGGTGTGTTCGGGCAGAAC
GCACAGTTCCATTACTTGTACAGGTCAGGTTTCTGCATTCATGTACAGTGCAATGCTAGTAAATTTCACC
AAGGGACCTTGCTTGTGGCTGCCATCCCAGAATTCACCGTCGCTAGATCAGATACCTCGACCAACCCTAG
CTCTGCGAGACATCCAACATACGAGGCTGCACAACCAGGGAAAAGCGGAAAGGAACTTGCATTTCCCTAT
GTTCTGGACTCAGGTGTACCACTGTCACAAGCTCTCATTTACCCACACCAATGGATCAATTTGAGGACTA
ACAATTGTGCCACAATAGTGGTGCCATACATAAATGCTGTCCCCTACGATAGCGCATTAAATCACTGCAA
CTTCGGACTGGTTGTTGTTCCGGTGGCACCTTTAGACTACAACACAGGTGCTACCACCGCCATACCTATA
ACCATTACCATTGCCCCAATGTGCGCTGAATTTGCTGGATTGCGCAACGCTGTCACGCAGGGGATCCCAG
TGGAATTAAAGCCAGGGTCTAATCAGTTCCTCACCACCGATGATGAAGTATCAGCACCCATTTTACCTGG
ATTTCACCCCACTCCCGAGATACACATCCCAGGCGAAGTTAAAAACATGCTGGAGATAGCTCAGGTGGAG

TCGATCCTGGAGATTAACAACTTGACTAGCAACGAGGGCAACCCCATGAACCGACTAGTCATACCTGTGA
GTGTCCAGTCTGATGTAGACCAACTCTGTGCTAGCTTCCGTGTCGATCCTGGCCGCGCTGGTCCATGGGA
GTCTACTCTTGTGGGACAATTGTGCCGCTACTACACCCAATGGTCAGGGTCTCTTGAAGTTACCTTCATG
TTTACTGGTTCTTTCATGGCGACAGGTAAAATGCTAATAGCCTACACACCACCAGGGGGTGCTCAGCCAC
TAACAAGGGAGGTCGCAATGCTTGGGACCCATGTTATTTGGGATTTTGGGCTGCAATCGTCTGTCACTCT
GATCATACCATGGATTAGCAACACACACTACCGCACTCAAGCAACATCCAACTACTTTGAGTACTACTCC
AGCGGAATAGTGACACTCTGGTATCAGACCAACTATGTCGTACCAGACGGGGCTCCTACAAGTGCTTACA
TCATTGCGCTAGGAGCAGCCCAACCCAACTTTTCGCTGAAGCTCTGTAAAGACACTGAATCCATCGCACA
GACAGCTATTTTGCAGGGGGATCCTGAGGCACCATTAGAAACATTTGTGGGGAACCAGATTAGCAAGGCT
CTCACGGCAGCTGACACAAAACCTAGTACTCACAAGATAGCGACTGGTGACACACCAGCCTTGCAAGCAG
CAGAAACTGGTGCCACATCAAATGCCAGTGATGAAAACATGATTGAGACCAGATGTGTCGTTAACAAACA
CTCTGTTGCCGAGACATCCGTGGAACACTTCTATGGGCGTGCAGCTCTAATAGGCTATGTGTCTATACTC
ACATCAGAGACCACAGCCAATGGTTTCACCAATTGGGACATCGACATCATGGGCTTTGTGCAACAAAGAA
GGAAGCTAGAAATTTTCACATACATGCGCTTTGATGCTGAATTTACCTTCATTGCCACCTCTGACGATGG
CCAGGCAGATTCCACAATGTTGCAATATATGTATATACCTCCTGGTGCCCCCAAACCAACACAAAGAGAC
TCATTTGAGTGGCAATCAGCAACAAACCCATCCATCTTCACAAGGATGACAGACCCGCCAGCACAAGTCT
CCGTGCCTTTTATGTCACCTGCTTCAGCTTACCAATGGTTTTACGATGGTTACCCAACCTTCGGGCAGCA
NNNNNNNNNNNNNNNNNNNNNNNNNNNNNNNNNNNNNNNNNNNNNNNNNNNNNNNNNNNNNNNNNNNNNN
NNNNNNNNNNNNNNNNNNNNNNNNNNNNNNNNNNNNNNNNNNNNNNNNNNNNNNNNNNNNNNNNNNNNNN
NNNNNNNNNNNNNNNNNNGGGGAAACTCCTGCATCACAACCCAAGAGGCTGCCAAAGCAGTGGTCGCATA
TGGCATGTGGCCAACATATAATATAGATGCTGGTGAAGCATTGGATTTGGATACTAAGCCAGGTCCCTCT
TGTGATAGATTCTACACTTTTGATTCTCTAGATTGGAAAGATACACAGTATGGTGAATGGGTGTGTCCGT
TACCTGGATCTCTCATGGATACAGGGGTCTTTGGGCAGAATCTCAGGTATCACTACTTATACAGGTCAGG
GTTTTGTGTGCATGTGCAGGTCAATGCCTCCCAATTCCATCAAGGGTGTCTGCTCGTCGCCATGATCCCT
GAACACCAAACTCCCAGGGAACAGCCCAACGACTTTGCCTATTGGGAACATAAAGATATGTGTGGATCAG
AGTACCCGACTGAGCAGCTGACACTCTTCCCCCACCAGTTTATAAATATCAGGACAAATAATTCTGCAAC
TATCGTATACCCCTACACCAATTGTACTCCTTCTGGTTTTGGTTTATCCCACAACTTTGTTACACTAGTC
ATCAGAGTTGTTGTTCCCCTAAATTATAGTGCTGGAGCTTCCACTGTTGTCCCTATCACTGTGAGCATTG
CACCAATGTGTTCATCATYWKMTSSWTTGAGGTCTGCCGTAACCGCCCAGGGCATCCCTGCTAGGTTAAT
TCCTGGTAGTGGTCAATTCTTAACTGTCCAAAGAGATTCTGGTATCCCATTATATCCTGAGTTTCAAAAA
ACTAAATCATTCACCCTCCCTGGTAGGGTTACCAACTTGCTAGAGGTAGCCAGGGTTCCCACCCTTGTTA
AGATGCCCAATAAGACAACTGACGGGACATACCTGGCAGTTGATGTTACACAGAATTGGCACACTGGAGA
GATTGCTAAATGGGATATGTCATTGGTTGGTGATATGTTTAAACCCACATACCTAGGTAGATTGGCTAAG
ATGTATGCAAATTATAGGGGTAGTATAGAAATTGAGTTCATGTTCTGTGGTTCCAAAATGGCAACAGGTA
AATTTCTTATAGCTTACACCCCTCCAGGTGGCTCAAGTCCTACAACAAGAGAACAAGCTATGTTAGGTAC
ACATGTAATATGGGATGTTGGTCTGCAATCTACTGTTAAGTTTGTTATTCCTTATATCTCCACCTCCACC
TATAGGTCAAGCATGTTAACAGGGACAACTCTGTCTTATGATGGTTGGATATCCATGTGGTATCAGACTG
CTATTGTGGTTCCCCCTGGTGCCCCTACTATCTGTYWSMYAMTKGRRKWKRMYRGMGSSKMTSMCAATTT
TGTGTTTAGGATCCCCTCTGACAACAAATATTATACAAATAATGCAAACATCCAGGGAGAGAAAGATGAG
ATATTGGCCTCTTTACAAGCAACAATCAGTAATCATATTGAGAATGCTTTGACAACCCATAAACAAAAAG
AAATCAAACATGATGGTATACAAATTGAACAGGGTGATTCTGCTGCATTAACTGCTGCTGAGACTGGAGA
AACATCAGGATCTAGTGGGGAGGATACAATGGAGCTCAGGGAGACAAACGTAGTATTCTCTACCAGAGAA
ACAGATTTAGAATACTTATATTCCAGGTACTTTATGTATTATCAAGGCACAGTTACTGTAACGAGTTCTA
CCATTGAAGGAGCTACCCCCATGATTAATAAGGAGTTAACATTTGATGCTGTTGCTGCCTCTGACCCTGC
AATCACTAGGTGGAGGGCATTTACATATTGGAGATTTGATTTAGATGTGTGTGTAGTGATTTATCCCCAG
ATTAATAACAACACATCCCCACAATCCATGACCTTCCAGATTATGTTTTCACCTGCTGGATGTGAAGTGC
CTGAATCATATAATTCTAAGGTGTGGCTGTCCCCCATTAATCCAACTGTGATAACCAAAATCCAAGATCC
CCCCABATATCCNNNNNNNNNNNNNNNNNNNNNNNNNNNNNNNNNNNNNNNNNNNNNNNNNNNNNNNNNN
NNNNNNNNNNNNNNNNNNNNNNNNNNNNNNNNNNNNNNNNNNNNNNNNNNNNNNNNNNNNNNNNNNNNNN
NNNNNNNNNNNNNNNNNNNNNAGTTAGGGCCAAGGATCTATTGTAGTGGTGTTGTATCCCCTTACCTTAT

ACCCATCCCCACCCAAACGGACTGGTATTATGGCTTTGATTGAATAATGGTTACTGAATTTAAACCTGTT
ACTCATTCAGGGGAAAAATGAAGACATTATCTATGGACTGGATCTGTTATGACCAAGAGTTAGCGTCTTG
CGCGCTGGCCGTATGGATTGTTCAATGGTTACTCTATGGATTATGATTTCAGGGTTGGCCGCCCAGGATA
AAAGGCAATTGTGGTAAGTGATGTTAGTCATTGGCAGTTGAAACCTGCCTAGAACATCYTAGGTCTACGC
TGTGCGGGCCGAAGTAAGTCTAGGAACAACAGGGAGTATGCTGTTTTCTGCTTTCACCCAACACGACCGT
ACACGAAAGAGCTAGAGGCATCTGGAGGCAAAGGGAAAAGAATTTCTTAGCCCGAATGTTCATTTGAGTC
CTTGACGAATGCGTCCCCTCTGTCCCGAGGGTGAGGCGTATGGCGCATGCTCATGGCATTACTCCAAGGT
GTATCTAGAGGGGGGGGGCCCCCTCTACTTAGTCTAGTGCTACCTGACAGGGCCGCGGCTGGTCGTTTGT
GTATGGTATAACCAGTAGTAATCCCCCATGGATTGCTTTAACTTCCCCTCCTCCCTTAACAAGACATTCT
CTAAGATGGCAATCTATGAGGATGACTCCTCGGAGTCTGTACAATGTTGCAACTGGATTCCTCGGAATCG
CCGTAACAAAGCAAGAAAGCAAAAGCCCAGGAGTAAGGCCTCCAAAGTTGAGATGTCCCCCGGACCACTT
GTCACMCTSGCSGACGAYGGSTATCTCTATGTGCACAAACATAAAGGGGAAACTAAATTTGTTAGGAGGG
ATGAAATTATTACAAAATATGGTAAATGTATAGTGGAAATGGAAGAGGTTTTCCAATGTGGACAAGTWCA
AYCWAMTMWMACAGGCAATAAACNNNNNNNNNNNNNNNNNNNNNNNNNNNNNNNNNNNNN
NNNNNNNNNNNNNNNNNNNNNNNNNNNNNNNNNNNNNNNNNNNNNNNNNNNNNNNNNNNN
NNNNNNNNNNNNNNNNNNNNNNNNNNNNNNNNNNNNNNNNNNNNNNNNNGAATTGACCAC
TACCAGGAATTAACCTAGCAGGGATGCCCTGGGCGGTTACGGCAGACCTCAATCCAGCAAATGATGAACA
CATTGGTGCAATGCTCACAGTGATAGGGACAACAGTGGAAGCTCCAGCACTATAATTTAGGGGAACAACA
ACTCTGATGACTAGTGTAACAAAGTTGTGGGATAAACCAAAACCAGAAGGAGTACAATTGGTGTAGGGGT
ATACGATAGTTGCAGAATTATTTGTCCTGRTATTTATAAACTGGTGGGGGAAGAKWKTMWGCTGCTCAGT
CGGGTACTCTGATCCACACATATCTTTATGTTCCCAATAGGCAAAGTCGTTGGGCTGTTCCCTGGGAGTT
TGGTGTTCAGGGATCATGGCGACGAGCAGACACCCTTGATGGAATTGGGAGGCATTGACCTGCACATGCA
CACAAAACCCTGACCTGTATAAGTAGTGATACCTGAGATTCTGCCCAAAGACCCCTGTATCCATGAGAGA
TCCAGGTAACGGACACACCCATTCACCATACTGTGTATCTTTCCAATCTRGAGAATCAAAAGTGTAGAAT
CTATCACAAGAGGGACCTGGCTWAGTATCCMAATCYAATGCTTCACCAGCATCTATATTATATGTTGGCC
ACATGCCATATGCGACCACTGCTTTGGCAGCCTCTTGGGTTGTGATGCAGGAGTTTCCCGCTGTTAACTG
CATCAATCTGTCKNGAATATCCAGCTTCTTCAACATTAGGTGATTTAAGTGCTGGTCCCTTCAGGGTGCC
TGCAATTTCTGTGWSRGGTTTGGTGAATTTCTCTGGGTCCATTTGTTGCTGGGATGGGTWATAWGCCTGG
GAATAATCACTTCCATAATAGTTAATATTTGTAAGGTGGTTTCCAGTTCCATAGATAAAGCCAGAGGAAG
GTTTATTGCCTGTTTGAKKAKATTGTRCTTGTCCACATTGGAAAACCTCTTCCATTTCCACTATACATTT
ACCATATTTTGTAATAATTTCATCCCTCCTAACAAATTTAGTTTCCCCTTTATGTTTRTGCACATAGAGA
TACCCGTCGTCCGCGAGTGTGACAAGTGGKCCGGGGGACATCTCAACTTTGGAGGCCTTACTCCTGGGCT
TTTGCTTTCTTGCTTTGTTACGGCGATTCCGAGGAATCCAGTTGCAACATTGTACAGACTCCGAGGAGTC
ATCCTCATAGATTGCCATCTTAGAGAATGTCTTGTTAAGGGAGGAGGGGAAGTTAAAGCAATCCATGGGG
GATTACTACTGGTTATACCATACACAAACGACCAGCCGCGGCCCTGTCAGGTAGCACTAGACTAAGTAGA
GGGGGCCCCCCCTCTAGATACACCTTGGAGTAATGCCATGAGCATGCGCCATACGCCTCACCCTCGGGA
CAGAGGGGACGCATTCGTCAAGGACTCAAATGAACATTCGGGCTAAGAAATTCTTTTCCCTTTGCCTCCA
GATGCCTCTAGCTCTTTCGTGTACGGTCGTGTTGGGTGAAAGCAGAAAACAGCATACTCCCTGTTGTTCC
TAGACTTACTTCGGCCCGCACRGCGTAGACCTARGATGTTCTAGGCAGGTTTCAACTGCCAATGACTAAT
ATCACTTACCACAATTGCCTTTTATCCTGGGCGGCCAACCCAATATNNNNNNNNNNNNNN
NNNNNNNNNNNNNNNNNNNNNNNNNNNNNNNNNNNNNNNNNNNNNNNNNNNNNNNNNNNN
NNNNNNNNNNNNNNNNNNNNNNNNNNNNNNNNNNNNNNNNNNNNNNNNNNNNNNNNNNNT
CCTTTAATTTGTGGTAAGGCTATACTTTTACAGGATAGAAGGACAAATGTTAGGTATACTTTAGATAAGG
TTGTTACTATTATGTTAGCTGAGTCTAGGAGAAGGAGAAATATACTCAATGTGGCTGATGCTTTGTTTCA
GGGTCCTAGCTATGAGAGACCAAAGCTTATCCGAAAACAAGTTGTTGACAARCTCCTGCCAGAGGAAGTT
GTTGAAGTATTGAAAGAGGTTCCATCAGAAGAGCTCATAGCTAAGTTGAAGGAGAAAGGTTATGTGTTCC
CCATTGAGGTTGAGGTTGTGAGGGAGAGGAACAAGGTGCAGAAATATCTAAATTGGTTTACAACTGTGAT
GTCCACTCTTACCACTATTGTATCCTTTTCAACTGTTATATATATGCTTGTTAGTGTCTTTGCTGGTAAA
CAGGGTGCTTATACAGGTAATGTAAAACCCACTATCCAGAAACCTGTACTCAGAAAAGCTGTTGTCCAAG
GACCAGACCTTGAGTTTGCCAAGTCTTTGATGAAAACATCTCTCTTTCCAGTGTGTACTTCTACAGGTTC

CTATACTGCATTGGGTTTGTTTGATCAATGGTTAGTTCTTCCYGCCCATGCGAATCCTAARGATAGTATT
GTATTTAAAGGTGAGAGYGTAAATATCCTTGATTCTATGCTTCTTGAGTCTAGTAGAGGTAACTTAGAAC
TAGTAGTTGTTAAGTTAGATAGGAATGAGAAATTTAGAGATATTAGAAAATATTTAGTTGAGAACTTCCA
TACAGAGAAGGAGTKMTGGTTRGCTTTAAATTCTGATCAATTTCGGGATGTCTATGTCCCAGTTGGYTCT
GTTTCTCTCTTTGGTTTTCTTAATCTATCTATGACACCCACGTATAATACCCTCAAGTATAATTACCCCA
CAAAAGTTGGACAATGTGGAGGAATAGTGGTGAAAGCAGGAAAGATCTTGGGAATGCACATTGGTGGTGA
TGGTGTTTCAGGTTATTCTGCAATGTTGAAGAAATCCTATTTTAGTGTTTGTCAAGGTGAAATTGTACAT
AAGGAATCAACCAAAGAGAGGGGTGTAAAATCTATTAATGTTAAAACCAAGACTGGGCTTTACCCTTCTG
TCTTTCATGATCTGTTTGAGGGAACAAAAGAACCTGCAGCACTTCGACCTGGCGACAGTCGTCTTAAGGT
TGATCTGAATGAGGCCCTCTTTGCAAAATATAAAGGTAACAAACATATTTCTATCCCTCCTGAGACCCTT
ATTGCTATAGATCATTATGCTGAACAAATTAGGCCCCTCTTAYCAGAAAATCTTACTGATCAATTGGAAT
TGGAAGATGTGGTTTATGGGATTGAAAACCTTGAGGGTCTGGACCTCAACACMYCWRYYSTGTATNNNNN
NNNNNNNNNNNNNNNNNNNNNNNNNNNNNNNNNNNNNNNNNNNNNNNNNNNNNNNNNNNNNNNNNNNNNN
NNNNNNNNNNNNNNNNNNNNNNNNNNNNNNNNNNNNNNNNNNNNNNNNNNNNNNNNNNNNNNNNNNNNNN
NNNNNNNNATTCAAGGGTGGTGTTGAAGGGTTTCTTAAAGATAAGCTTTTCAACTCTCTTTTCTCTATAC
TTGTTAAGGTTATAGGTTCTCTTGTAATATATATAAATGCTAAGGATGAATGTAAATTATCTACTCTTCT
TGCTTTAGGTAGTATGCTAGGTGTAGATTTCCTATCTAAAGACCCGTTTACCTATCTTTATGAGAAGTTT
ACTGGGTGTGTCCAAATGCAGGGTCCTAGTGATTGGCTAAAAGACTTCAACATAGCATGCAATGCTTTTA
AGGGACTRSWATACATTTACCAAAAACTGTCAACATTCTTTGATTGGATAAGGGGACTTCTGAAGAAAAA
TGAAGATCCAGCCAGACATGAATTCCACGGGATGCTTGAAGACTGGCCAGAAATAACGGAAAAGATGGAC
TTAATAGAGAGAAACAGGAAAAACTATCATGATGCTGATGTTCTAGAAGTCTGTGAATTTGTYATCACAA
TTAAGAAATTGGCTGACACTTATGGCATTGAGAGAAACATGGCTACAACACAAATTATTAAATATCATGC
AAAAGCACAGAAACTGGCTCAATCTATTTAGGATCTTTGACAAGTAAGGTACAGGTACACACCTTTTTAT
CTCTATGTTNNNNNNNNNNNNNNNNNNNNNNNNNNNNNNNNNNNNNNNNNNNNNNNNNNNNNNNNNNNNN
NNNNNNNNNNNNNNNNNNNNNNNNNNNNNNNNNNNNNNNNNNNNNNNNNNNNNNNNNNNNNNNNNNNNNN
NNNNNNNNNNNNNNNNNNNNNNNNNNNNNNNTTGTGGCTGGGTCTGAAGCAGCTACAGCATCAAATGTGA
AAGATTTGTTAATCATTTGTTTACCAATGTCAGCAGAGGTTATGGTGATGTTTTCTTCATAAAACAGGAA
ATACCTTGAATACAAGTACTCAATGTCTGTTTCACGTAWAGAAAAGACMGCATTTGATTCCCTCAATTCC
ATACTAGTCTGCCCACTACTTTCTGATGTCTCTCCAGATTCAGCAGCTGTTAATGCAGGAGCATCACCTT
GTGTAATCTCTAGCCCATTGTTGATATGGATATAGCTGTGAGGTGGTTTGTAAGGCATTAGATAAGACTT
GTTCAACATGTGCATTTAGGGCAGCAGGTATTGTGTCAGTCTCTCCCTGGGTCTCGGCACTTCTGGGGGY
ACCTATGTTCACATTATCAGTATAATACTTGTTGTCTGACGGGATCCTAAACACGAAGTTGTCTGAAGCA
CTAACCAATCCTATAATTTGACAGGTTGAGGGGCAATTTGGTGGGACTACAATTGCTGTTTGATACCACA
TTGTTATCCACCCATCATAAGAAAGGGTTGTATTTGTCAACA

>Simian bocaparvovirus
TGGTGTGTCTAGAAACGAAAGCTCAAAATTAACCACCGCGTCTTCCTTCTGAGGTGTGAAGTTTTTGACT
AAAGTAGCGCACTTACTTGAGGCTTCAGCTCCAACCAGATCACCACAATCAGTGTCAAGAGGCAGATAAG
TCCCGCAATGACGGCAAATTCCGTTTTCATGAAGAGTGAAATCCTGTGAATGACCATCACAGATTTTATT
AAGAGGAAATGAATTTGGTGTACATTCAAGATTCCATTCAGAGTAAAAGCCTTGAAGCGTGCAATTGAAA
CGCGAGCCGCAAGTCAAGAGCCAGGCAGCCACTTCTTCTTCTGTGATTTCTCCAAATGTGCTCTCTAGTT
GATTCATGAAATTAAACTGAACCACTCTGTCTTTAATTGGCTTGGCATGCACTTGAATTACAGTGTTTCC
TCCTGTTGTAGCTCAAGCAGCACAAGCCGCGGTCAAAAAAGAGTTTCAGCTGAAACAAAGAACGAGAGAG
CCTGCATTCTCTTGTTATGTCCARGTAGAACTAGGCCGAGAGAATCTACACGTACATGTAGTGATTGGAG
GCAAAGGACTAAACAAATACAATGCTAAGCCGTGGAGAAATCCTCTGGCATACTACTGGCTGACATACAT
TAAAGAACACATTCAATCGACGGCAGACTTGATCCTTGAAAGGGTTGAATATACCAACATTCAATGGGCG
CTAGACAGAGAGATCACTAGATGCAAGGCTGCATACACTGACATTTGTAAGATTTTAGAATACAGAAACA
GAAATGGCCAGAAATATGCATGCAGAGTTGATCCGATGGAGTTCATTTCTAACTATCTGCTGTGCAAGAA
CTTAAAATACAGTCTATATCATGAACCTATTCAGATAACTCCATATAGAAGCTTCTTTGTTGGAACAAAT
AAGACGTATGCGATCACTTTAATAAATGGAAAATACATTCAGCCTTGGGTTAGAAAACACTGGACAGACG

AACTTCAAGCTAACCTCAGCAGAAACAAAGAGGAGCCTGTTTTTACAGGAGATCCATTTGGAATGTTGCC
TAAGGTAGACCAAGCGAATTGGACGGGAGCGACTCAATTGCGCGGGAAAATGACTAAACGAGAAAGTTTA
ATGATTGACTGCATGCAGAGATATCTAGAAGGGCACTTGTTAACCTATGAACTGTTGGTTGGCAGTCACC
CTGATCTTGTTGTAATGATTGAATCGCAACCTGGCGGGGCTAGATTACTAGAGCAGATCCTGGGCATGGT
TCACATCAGGCTGTGCCAGACATACACTGCGTTTACTTATCTAAAGACTTTGTACCCTAACTACCAAGTG
TACTCTGACAACAGAGTGTTTAAACTGCTGAACAAACAAGGGTACAATGCGTGGCAGGTTGGACATTGGA
TTTGCTGTGTGCTAGATAAAAAAGCTGGAAAACAAAATACCTTAT

>Simian protoparvovirus
GATAGGTTGGTTAGTAAAAAAATGACTTACATGGTTAGTATGTTAGATTGTGTAGTTAGGCATTGCTCTT
CCTGGCATGTATGGAATTTCAAAGTTTCCAATTTCATTTGGTACAGTTTCTTTCATGCTTTCTCTTGGAG
CGAGTGATGGTAGTTGATAGGTGTTCCATTGTCTTGGAGTTCTAAGTTTGGCTTTGATGATTAGTTTTCC
TGTCCACCAAAAGGTTCCAAATGTAACTATTCTTGATGAATTGCCATTGTTGTCTACAGTGTCTGTGTAG
TTTGGAGCTAGTTTAACAAAGATTTGTCCTGGTGGATTTGGTGTCATGAATGGAGCGTGTGCTGATGCCA
TTGGTTTGTGAGTTGTGTTTGGTTCTTTTCCCCATATGGCTCCCCATGGGTATTGTGCTCCATGGTCATC
AAATGCTGTGAATGGTCCGAATGTGTTGTGGTAGCCTTGTGTCCAGAATGCGTCTCGTCCAGCTACATTG
TTGTTGAGTTCTTTTTGGTGTATGTTTTTTGGTGCCCAGTTGGTTTGTCCTGTCATTTGTTGATTGTTTT
GCCACCATGTTTCTCTGAATTGGTGTTCTTCATCACCATGGTTCCAATCATATATTGCTTTTTCAGAAGC
TCCTTGTGTTAGTCTTGTGCCGGCAAATANNNNNNNNNNNNNNNNNNNNNNNNNNNNNNNNNNNNNNNNN
NNNNNNNNNNNNNNNNNNNNNNNNNNNNNNNNNNNNNNNNNNNNNNNNNNNNNNNNNNNNNNNNNNNNNN
NNNNNNNNNNNNNNNNNNNNNNNNNNNNNNNNNNNNNNNNNNNNNNNNNNNATAAGGAGGAGGAGTAAAA
GTTAAGGCTCAATGGATAGGTGGGACTTCTTTCTCTGATTCCTTCATAGTTACTTCCCATACCAGAACTT
CTATGTTAGCTGACAGAGGCAGTTATTTGCCAGTGTATAAGCAAGGTTCTCACAATGATGCTTCACAGCC
TGTGATGGGTATGCGCACTCCTTATTCTTATATTGATGTGAATGCAATCTCTGCTCATTTAACTCCGCGT
GATTTTCAGCAGCTGCTAGATGAATATGATGAAATTCGCCCTAAAGCTTTAACAATTAGCATCTCTGCAA
TTGTTGTTAAGGATGTGTCAACTAATGCAAATGGGACTACTGTCTCTGATAGCAGTAGTGGAGGCATTAC
AATTTTTGCTGATGACTCCTATGATTACCCCTATGTGCTGGGTCACAACCAGGACACGCTGCCAGGACAC
CTGCCAGGTGAACACTATGTACTTCCTCAGTATGGTTACATAACTAGAGGCCGAGAAATAGAGAAAAATG
GTTCTATTGTAGCCATTAGTGATCATAAGACAGAGTTGTTTTTTTTGAAGCATCATTTGCAAAGTCTTCT
TTATAACATTTTACTTGAAAGAGAGTAATCCTAGGACAGAGGACAATTTGCCTAAATGTATNNNNNNNNN
NNNNNNNNNNNNNNNNNNNNNNNNNNNNNNNNNNNNNNNNNNNNNNNNNNNNNNNNNNNNNNNNNNNNNN
NNNNNNNNNNNNNNNNNNNNNNNNNNNNNNNNNNNNNNNNNNNNNNNNNNNNNNNNNNNNNNNNNNNNNN
NNNNNNNNNNNNNAAAGTGGKTACAAAAACAGCTCAAACAATGGTTTCCGGATGAACCCCCTATGCTTCA
ATGACAGAWGGTTAATTCACCAACATTTGCGTGAGCTATCTTTGAGAAAGGAAACCACCCAAATTCAAAG
CATATCACCTCAAGAAAAGAAAAAAAAAAGATTAGAAACTGCAAAAGAACAATCAATTAAAGAAACAATA
TACACATTAACAAAAAACAGACTACATACACAAGAAAAATGGATGCTTGGTGATCCGGACAGTTACATCC
AACATATAGCCAATCCAGGAGGAGAAACTATAATCAAAGCTACACTTGATATAGTTACTCTAAAAATGTC
AGTAGAAAAAACTGCATATGACCTAATCATAGAAACTGAAACAACCGACCAACCAAAAAAAGCTAAAAAA
ACCAAAGCATGGAAACTTCTCAAAAACAATAACTTTAATCCAAAAAAAGTATGGCATTGCATTTCATGTG
CTTTAAACAAACAAATGGGAAAAAGAAATACAATACTACTATGCGGACCAGCAAGCACAGGAAAAAGCCT
AATAGCACAAAAAATTGCACAACTAGTTGGTAATGTTGGTTGTTACAATCCAAGCAATGTAAATTTCCCA
TTCAATGACTGCAGCAATAGAAATCTAATATGGGTTGAAGAAGCTGGAAACTTTGGTGGACAAGTAAACC
AATTTAAAGCAATTATGAGTGGTCAAAGTATAAGACTAGACCAAAAAGGAAAAGGCAGCAAAGCGATTGA
ACCAACACCAGTAATAATGACAACAAATGAAGACATAACAAAAGTAATAATAGGCTCAGAAACCAAACCA
GAACACAAACAACCAATAATGGACAGATGCTGCAGAATAGAAATGAAAAAAAGACTAGAAGGTGACTTTG
GTTTACTAGAAGATGGTGAAATTCCAAGCATCTTCAAATGGTTAGAAAGTCATGCATTCCTACCAACTAT
GGCAAACTATTGCATCAGATGGGGCGGTCCTCCAACCTGGGGAGAAAACTGGAATACAAAACCTAAAGNN
NNNNNNNNNNNNNNNNNNNNNNNNNNNNNNNNNNNNNNNNNNNNNNNNNNNNNNNNNNNNNNNNNNNNNN
NNNNNNNNNNNNNNNNNNNNNNNNNNNNNNNNNNNNNNNNNNNNNNNNNNNNNNNNNNNNNNNNNNNNNN
NNNNNNNNNNNNNNNNNNNNTCACAGAGAAAGAGATCTCTGCAATCAACTTTCTCAAACAAAACCTAGAA

AAAACAGGACTAAGCTTTGTATTCAAAATACATGAAACTACTTTCAAACCAACTGATGGAAAACCTATAA
TAATAGACTGGACTGACTACATGAAACCATCCAAAGATGAAGACACCAAAGAAGTCTGGAAACTAGATCA
ACAAACTGTGAGTAACAGAGATCCAGACTGGGGGTGGAAACCAGATATAAATGAAACTCCATACAAACAA
GCTCTAGTAGGAGTCAGTGGAATTAAAAGAATACTGTTTGACTACTTTAAAACTAAATGCATAGAACCTA
AAGAAGTCAGATGGTTCATACAAGCTGRACTAGGACAAGACTCAGGCCTACACTTTCATGTACTACTACA
ATCAGAAAAAATTCAACAATCATCTGGTAAATGGATGGTAAAATTCTTTGCTGAAAAATGGAGCCTCTTT
CTAACTCAATGCATACCAATGAAAAAAGAGGATCTCCATCAATTTTTCAACCAAACCAAATTCAGACACA
CAGTTGAAAACAATGATTGGATCCAAGTACTCTGCTACAC

>Simian dependoparvovirus
GATATTGATCTTGCCCGTGGTGGCCGGCCCAAAGAGCCAGATGGTGTTGCGCTTCCCAAACCTTTTCTGG
GCCCAGCCGAGAAAGACAGAGCCGGCGTAGGCAGGGTCGTAGCCGTTCAGCTCCAGGATGCGGTAAATGC
GGTTGGTTTTAATGTCCGCGGGCGGAGCGGGGCCTACCAGGTAGTCGGGCGCGGATTTGGTCAGCGCCAT
GATCTTGCCGGCATTGTCCAGAGCGGCCTTGATCTGGGACCGCGAGTTGGAGGCGGCGTTGAAGGAGATG
TACGAGGCCTGGTCCTCCTGGATCCMCYSCYYCYCGSAGGYSATGCCCCGGTCCACCAGCCACCCGACCA
GCTCCATGTAGCGCGCGGAGGTTTTTGATCGGATGACAGGCGCGTCAGAATTGGGGTTCAGATTCTCCTT
GTTCTGCTCCTGGGTCTGGCTGACGTGGGTCAGGTGCTGCGCCACGAGCCGTTTGCGCTCGGCCAGGTTC
AAACACGCGCTTATATACTCCTCCATGTTAGTCCACGCCCACTGCAGCTCGGGCTGAGTCTTGGGCAGGA
GGTAGTTGGGGATGTAGCACTCGTCCACTACCTTGTTCCCCCCTCCGGCGCCATTACGCGTCTTGGTCAC
CGCGAACCAGTTGGGCAGGGTCGGCTCGATCCCGCGGTAGATGGTCTGCACCAGCTTGTCCCTAATCTGA
CTCAGGAAGCGGCCCAGCACCATGGATTTGACNNNNNNNNNNNNNNNNNNNNNNNNNNNNNNNNNNNNNN
NNNNNNNNNNNNNNNNNNNNNNNNNNNNNNNNNNNNNNNNNNNNNNNNNNNNNNNNNNNNNNNNNNNNNN
NNNNNNNNNNNNNNNNNNNNNNNNNNNNNNNNNNNNNNNNNNNNNNNGTACTGCGTGATAAAAGAGTCCA
CTCTGGCTGCAGTGAAGGTTGTCGCCGGATKGDHAGGTACGGGGGTATTTTTGATGAAGATCTGAGGAGG
CGGGTGTTTCAGTCCAAACCCACCAATCAGCGGTGAAGGATGAAAGTGTCCATCCGCGTGTGGGATCTTG
GCCCAGATGGGTCCCTGGTAGTAAATGTCTCTGTTTTGCCAGACCATACCAGGCAACACGCCCATAGCCG
TAACATTGCCGGTGATGGGAGCGGTTGTGGCATTTTGATTATTGTCAGCAATTTGACCAAACATGTCGGT
GTCTCTCGGGTTGGTAGCAGCGATTTCTTCTTCTGATGTAAACAGCAAGTTATTGGCTGAAGTTGTGGTG
GTTCCAGTGATAGAAGGTCCAGGGAAAATGAGCTGTGCGTTGCTAAAGTCCCCATCTGAAGGTCCGGCTG
TGGCCATAGGAGGTCCAGGTGCAATGTTGCTCCATCGGTTGTTTAAGGTATAGTGGGTGTCATATTTTAA
CAAAGCGTTGCCTCCACTTGCAGGAATCTTGTAGTTTTGACTGGCAGTCTTTGAGAACATCTGCTGCTTA
ACACAAGGCCCAGGCAGCCAGTTCTTTCTGTAAAAGGCAAAATCCCCGCTTCTGATCTTTCCAAATGTGG
TTGCTGCATTGCCTTGATTCAGAGTCTCTCCAGAGGTGGTCGACTGCAAGTGCCACAGGTACTGGTCCAG
GAGAGGATTCATCAGTCTGTCCAAGCTCTGGCTGTGAGCATACATGGAGTGGAACGGCACCTTCTCAAAG
TTGTAAGCCATTTCAAAGTTGTTGCCAGTTCTCAGCATCTGTGAAGGAAAGTACTCCAGGCAGTAGAAGG
CATTTCTGTCCGTTTGGTTTTGATTTTCACCAGTTACGATGCCACAGTAGCCATATTGAGGCACCATGAA
GACGTCATTGGGGAACGGAGGCAGGCTCCCTTCTTGTCCTGCATCCATCACGTACGGGAGCTCATACGAC
GAGTCCGCAAATATTTGAACCGTGCTGGTAAGGTTATTAGCGACCGTAGTCTCGCCGTTCGACGTTGTGA
CCTCCTTAACTTGGATGTTGAAGATTTTGACCCGCATGGCTTTGGGTCGCAGTCCCCAGTTGTTGTTGAT
GAGTCGCTGCCAGTCACGTGGTGAGAAGTGGCRGTGGAATCTGTTGAAGTCAAAGTATCCCCAGGGGGTG
GAGAATCCGTTGTATGTATTGCTGCTGGACGTGGTTCCGAGCCGCAGGTACAGGTGGTTGTTGTAGGTGG
GCAAGACCCAGGTTCTGGTTGAGGTGGTTGTGACTTTGCCCTCAGACCAGGTGGAATCGCAATGCCAATC
ACCCGAGGCATTACCCACTCCATCGGAACCTTGTCCCGCATCGACAGAATTTCCGCCCGGTGCTGCACGC
ATTTCAATGTCTGAAGACATGGCGCTGGTATCAGATCCTTCAGGGGGTCCGTCTCCGGCTCCAGTGTCCT
CCTCAAAGTTAAGTCTTTTTTTGGCTGGTTGTTTGCCCTTTTTACCGATTCCCGTGGAGGAGTCGGGATG
CTGTGGTGACTCTACCGGTCTCTTCTTTCCAGGAGCCGTCTTAGCGCCTTCCTCAACCAGRCCSAGAGGT
TCGAGWACCCKCTTYTTGGCCTGRAAGACTGCTCGYCCGAGGTTGCCCCCAAAAGACGTATCTTCTTGCA
GACGCTCCTGAAACTCGGCGTCGGCGTGGTTATACCGCAGGTACGGATTGTCACCCGCTTTGAGCTGCTG
GTCGTAGGCCTTGTCGTGCTCGAGGGCCGCGGCGTCCGCCTCGTTGACGGGCTCCCCCTTGTCGAGTCCG
TTGAAGGGTCCGAGGTACTTGTAGCCAGGAAGCACCAGACCCCGGCCGTGGTCCTGYTTYTGCTGGTTGG

CYTTGGGCTTCGGGGCTCCAGGTTTCARGTCCCACCACTCGCGAATGCCCTCAGAGAGGTTGTCCTCGAG
CCAATCTGGAAGATAACCATCGGCAGCCATACCTGGTTTAAGTCATTTATTGCTCAGAAACACAGTCATC
CAAGTCCACGTTGACGAGATCGCAGGCCGAACACGCAATCTCGGGTGCCCGCCCCAGCAGATGATGAATG
GCACACAGTTTCTGATACGTCTTTTTTCTGACGACAGGAACGGGTTGAGATTCTGACGCTCCGGGGAAGC
ACTCGGAACAGTCTCTGACCCCGTGCGTGAAGCAAGTGTTGAAATTCTGATTCATTCTCTCGCATGTCTT
GCAGGGAAACAGCATCTGCAGCATGCCCGCGTGACGAGAACATTTGTTTTGGTACCTGTCGGCAAAGTCY
ACCGGWGCTNNNTCCGCGTCTGACGTCGAKGGMTCCGCGACTGAGGGGCAGGCYCKCTTGGGCTCGCTTW
TATCSGCGTCAYCGGGGGCGGGKCTCTTGTTGGCTCCACCCTTTCTGACGTGGAACTCATGCGCCACCTC
GGTCACGCGRTCCTGCGCCCAGSGGRRGRACTCTTTGACTYCCTGCTTKGTSACCTTGCCAAAGTCRTGC
TCCAGRCGGCGGGTGAGTTCAAAYTTGAACATCCGGTCCTGCAACGGCTGCTGGTSCTCGAAGGTGGTGC
TGTTCCCGTCAATCACGGCGCACATGTTGGTGTTGGAGGTRACGATCACGGGGGTGGGATCGATCTGGGC
GGAAGACTTGCACTTTTGGTTCACGCGCACTTTGCTGCCGCCGAGAATGGCCTTGGCGGACTCCACGACC
TNNNNNNNNNNNNNNNNNNNNNNNNNNNNNNNNNNNNNNNNNNNNNNNNNNNNNNNNNNNNNNNNNNNNN
NNNNNNNNNNNNNNNNNNNNNNNNNNNNNNNNNNNNAGCCAGGAGCTCCATTTTGCCCGCGGATTTCAAA
CGAGCAGCAACCATGCCGGGATTCTACGAGATCGTCGTGAAGGTTCCCAGCGACCTGGACGAACACCTGC
CGGGAGCTTCTGACAGTTTTGTGACCTGGGTGGCCGAGAAGGAATGGGAGCTGCCCCCGGATTCTGACAT
GGACATTTCACTCATTGAACAGGCACCCCTCACCGTGGCAGAAAAGATCCAGCGCGACTTTTACGTGGAG
TGGAAACGGGTGAGCAAGGCCTCAGAGCCGCTATTTTTCAATCAATTTGAGAAGGGGGACTCGTATTTCC
ACATTCATATGCTGATTGAGACCAGTGGAGTTAAATCCATGGTCCTCGGCCGATATCTCACACAAGTCAA
GGACAGGCTCGTAGCCCGCATTTTCCGGAATGTGGAGCCTACTATGCCAAACTGGTTCGCGGTGACCAAG
ACGCGTAATGGCGCCGGAGGGGGCAACAAGGTGGTGGATGAGTGCTACATCCCCAACTACCTGCTCCCAA
AAACCCAGCCGGAGCTCCAGTGGGCGTGGACTAACATCCCCGAGTATATAAGCGCGTGTCTGAACCTGGC
TGAGCGCAAAAGACTCGTGGCCCAGCACCTGACTCACGTCAGTCAGACTCGGGACGAGAACAAGGAGAAT
CTGATACCGAATTCAGACGCTCCCGTGATCAGATCAAAAACTTCTGCGCGCTACATGGAGCTGGTCGGGT
GGCTGGTGGACAAGGGCATCACCTCAGAAAAACAATGGATCCAGGAGGACCAGGCCTCGTACATCTCCTT
CAACGCCGCCTCCAACTCGCGGTCTCAGATCAAGGCCGCTCTGGATAATGCCGGAAAGATCATGAGCCTG
ACAAAAACCGCCATCGACTACCTGGTTGGGTCAGATCCACCGCAGAACATCTACCAAAACCGCATCTATC
GGATCTTACAAATGAACGGCTACGACCCCAGGTACGCGGCTTCCATTTTCCTGGGCTGGGCGCAAAAGAG
GTTCGGGAAGCGCAACACCATCTGGCTGTTTGGGCCCGCCACCACCGGTAAGACCAACATCGCGGAAGCC
ATCGCTCACGCGGTGCCCTTCTACGGCTGCGTCAACTGGACCAATGAGAACTTTCCCTTCAACGATTGCG
TCGACAAGATGGTGATCTGGTGGGAGGAGGGCAAGATGACCGCCAAGGTCGTGGAGTCCGCCAAGGCCAT
TCTCGGCGGCAGCAAGGTACGCGTGGACCAAAAGTGCAAGGCCTCGGCCCAGATCGACCCCACGCCCGTC
ATCGTCACCTCCAACACCAACATGTGCGCCGTGATCGACGGGAACAGCACCACCTTCGAGCACCAGCAAC
CGCTGCAGGATNNYGGATGTTCAAGTTCGAGCTCACTCATCGCCTGGAGCACACCTTCGGCAAGGTCACC
AAGCAGGAAGTGCGTGAGTTTTTCCGGTGGGCGCAAGACAATGACGTGCCCGTGACTCATGAGTTTTACG
TCAGAAAGGGCGGAGCCAACAAACGCCCCGCCCCCGATGACGAGGATATAAATGAGCCCAAGCGTGCCTG
CCCCTCAGTCGCGGATCCATCGACGTCAGACGCGGAAGCACCGGCGAACTTCTCCGACAGGTACCCGAAT
AAGTGTTCTCGGCACTTGGGCATGAATCTGATGCTTTTTCCGTGTAAAACTTGTGACAGAATGAATCAGA
ATGAGGACGTTTGCTTTACTCACGCTGTCAAAAACTGCGCGGAATGTTTTCCGTCAGAAGCTCCTCAGGT
TGTCAAAAAGACTTATAAGAAACTGTGTGCCGTTCATCACATCATGGGGCGGGCTCCGGAGATTGCCTGC
TCTGCCTGCGACCTGGTGAACGTGGACTTGGACGATTGCATTTCTGAGCAATAAATGACTTAAACCAGGT
ATGGCTGCTGATGGTTATCTTCCAGATTGGCTCGAGGACAACCTCTCTGAGGGCATTCGCGAGTGGCTGA
ACCTCAAACCTGGAGCGCCTCAGCCCAAGGCCAACCAAYAWNNNCAGGACAACGCTCGGGGTCTTGTGCT
TCCTGGATACAAGTACCTCGGACCCTTCAACGGACTCGACAAGGGGGATCCCGTCAACGAGGCTGACGCC
GCGGCCCTCGAGCACGACAAAGCCTACGACAAGCAGATTGCGGACGGAGATAACNNNNNNNNNNNNNNNN
NNNNNNNNNNNNNNNNNNNNNNNNNNNNNNNNNNNNNNNNNNNNNNNNNNNNNNNNNNNNNNNNNNNNNN
NNNNNNNNNNNNNNATACTGGGTGGCCGAGAAGGAATGGGAGCTGCCCCCGGATTCTGACATGGATCCGA
ATCTGATTGAGCAGGCACCCCTGACCGTGGTCGAGAAGCTGCAGCGCGACTTCCTGGTCCAATGGCGCCG
CGTGAGTAAGGCCCCGGAGGCCCTCTTCTTTGTTCAGTTCGAGAAGGGCGAGTCCTACTTTCACCTGCAC
GTTCTGGTGGAGACCACGGGGGTCAAATCCATGGTGCTGGGCCGCTTCCTGAGTCAGATTAGGGAAAAGC

TGGTGCAGACCATCTACCGCGGGATCGAGCCGACCCTGCCCAACTGGTTCGCGGTGACCAAGACGCGTAA
TGGCGCCGGMGGGGGGAACAAGGTGGTGGACGAGTGCTACATCCCCAACTACCTCCTGCCCAAGACTCAG
CCCGAGCTGCAGTGGGCGTGGACTAACATGGAGGAGTATATAAGCGCGTGTTTGAACCTGGCCGAGCGCA
AACGGCTCGTGGCGCAGCACCTGACCCACGTCAGCCAGACCCAGGAGCAGAACAAGGAGAATCTGAACCC
CAATTCTGACGCGCCTGTYATCCGGTCAAAAACCTCCGCGCGCTACATGGAGCTGGTCGGGTGGCTGGTG
GACCGGGGCATCACCTCCGAGAAGCAGTGGATCCAGGAGGACCAGGCCTCGTACATCTCCTTCAACGCCG
CCTCCAACTCGCGGTCCCAGATCAAGGCCGCTCTGGACAATGCCGGCAAGATCATGGCGCTGACCAAATC
CGCGCCCGACTACCTRGTAGGCCCCTCTTTGCCTGTGGACATTACGCAGAACCGCATCTATCGCATCCTC
GCTCTCAACGGCTACGACCCTGCCTACGCCGGCTCCGTCTTTCTCGGCTGGGCTCAGAAAAAGTTTGGAA
AGAGGAACACCATCTGGCTGTTTGGGCCCGCCACCACCGGGAAGACCAACATTGCGGAAGCCATCGCCCA
CGCCGTGCCCTTCTACGGCTGCGTCAACTGGACCAATGAGAACTTTCCCTTCAACGAYTGCGTCGACAAG
ATGGTGATCTGGTGGGAGGAGGGCAAGATGACGGCCAAGGTCGTGGAGTCCGCCAAGGCCATTCTCGGCG
GCAGCAARGTGCGCGTGGACCAAAAGTGCAAGTCTTCCGCCCAGATCGATCCCACCCCCGTGATCGTCAC
CTCCAACRCCAMCATGTGCGCCGTGATTGACGGGAACAGCACCACCTTCGAGCACCAGCAGCCGTTGCAG
GACCGGATGTTCAAATTTGAACTCACCCGCCGTCTGGAGCACGACTTTGGCAAGGTGACAAAGCAGGAAG
TCAAAGAGTTCTTCCGCTGGGCKMRKGATCACGTGACCGAGGTGRCGCATGAGTTCTACGTCAGNAWAGG
GTGGRGCCAACAAGAGACCCGCCCCCGATGACGCGGATATAAGCGAGCCCAAGCGAGCCTGCCCCTCAGT
CGCGGATCCATCGACGTCAGACGCGGAAGGAGCTCCGGTGGACTTTGCCGACAGGTACCAAAACAAATGT
TCTCGTCACGCGGGCATGCTTCAGATGCTGTTTCCCTGCAAGACATGCGAGAGAATGAATCAGAATTTCA
ACATTTGCTTCACGCACGGGACCAGAGACTGTTCAGAATGTTTCCCCGGCGTGTCAGAATCTCAACCGGT
CGTCAGAAAAAGGACGTATCGGAAGCTGTGTGCGATTCATCATCTGCTGGGGCGGGCACCCGAGATTGCT
TGCTCGGCCTGCGATCTGGTCAACGTGGATCTGGATGACTGTGTTTCTGAGCAATAAATGACTTAAACCA
GGTATGGCTGCCGATGGTTATCTTCCAGATTGGCTCGAGGACAACCTCTCTG

>Simian chaphamaparvovirus
TCCTCCAGTGGGAAACACACAAACTGGCGGCCATGGAAGACATAGAAGAACCAGARCCAACAWTRGARAT
CTGCAAATGCCAAAAAGCACACAGACCAGAAGCGCTCCTRTGCTACATGGTGAAAAACCCGCTCTTCATA
GCAGCACACAATCCAAAATCACTACAATACGCATATTCACTCTACTTCCATAACAAAGGCCAGAAATACT
TAGACAAAAAAGAGGCAGAAAGACAAAGAAAACAAACACTCCCAATAGAAACCATATCAGGAGGACACCC
AATCACCAGAGACATCCTCAGCATCATCTACAAATACAACTGCCTGACATCAGAAGACATCTTCAAACAC
GAGCCAGACATAATCATCCAACACCTACACAAACCAGGCTTCGCCAACATAATCAAAAACTGCCTGGCCT
TCGTAGAGGCCACCAAAGGCGAATGGTCACTAGAAAAGAACGCACACAGACACAGACCAGACCCCACAAC
AGNACACAWKWKCATCAACCACCAGGGCCTAGACACAGAAGAGGTAGATTACATGCTCTATCAGTGGATC
TCTAAATCACACCCAAAGAAAAACACCGTCCTCCTCATAGGACCCAGCAACACAGGCAAATCAGCCTTCA
TCAGAGGTCTCAGAGGAGTCCTAGAAACAGGAGAAATCTGCAACGGGCAAGTATTCTGCTTCGAGGGACT
GGTAGGCAAAAAGCTAGGCATATGGGAGGAACCTCTAATCAGTCCAGAGTCAGCAGAGAAAGCCAAACAG
GTATTCGAAGGGGCAGACACCTCAGTACCAGTAAAATACAAAAAACCACAACCACTAGGCAGAACTCCCA
TAATCATCACCAGCAATCACTGGCCGTGGCGCTACTGCACCGCGGAAGAAAACCCGTCCAGAAACAGGAT
GTTCATCNNNNNNNNNNNNNNNNNNNNNNNNNNNNNNNNNNNNNNNNNNNNNNNNNNNNNNNNNNNNNNNNN
NNNNNNNNNNNNNNNNNNNNNNNNNNNNNNNNNNNNNNNNNNACTCATGGGTCCCCAGGTGGGTCCATAG
TATCTTGATCTTCTTTTTTTCACTTTTAAATGGATTGTTGCTCTGTAACATCCTTGTGTGCTTGTTTGTA
TTAATGTATTATTTTCATCGAATAGTGGAATTCCTTTTATGAAACATTGTGAGGGTGGATATTTATAACT
TTGATATTCTGGACCTGGCCATCCTAATTGTGGTTTTTTTATGTCTGGTGTTTCTATCATTGTTTTGTTC
ATTTCTATCCAGAACCATGATACTGGTACGATTGGTGCATTGAGTAAATTTGGCATGTCATATCCTAGGT
ATCTTCCTTGGTATGATGGTGCTGCTTTGGGTGATTCTGCCTGTCCGCTTGTATATGATGTTTGTGGAAA
TGGATCTGATGGAAAGTTATTTGGTATGTAGCTTCCATCTGGTCCTAATCTGCTTTGTTCTGTTCCTGCT
GTATCTGTATACGTTTGGTCCATCCAGGGTCCTTCTGGTATGTATGGCATGTATTTTGCTAGTAGGTCTA
GGTTGTACCAGATGCCTTCATCACATGGATGTGCATTCCAGTTGAATGTCATAGCATTTTTCCCTGGTCT
TAGTTCTAGTATGTGATCTGGGTCTGTTAATGGGTCCCAATATGTACCTGTTCCTTGTGCTATATTTGGA
GTTCCTGTATTTGTATGTGCCCAGACGCTGGTAGCGTATGGATAATTTGGTGGTACTGGATCCCAGCTCC

AGGTTCTGACATCATATGGATCGTGTCCAGGTGTTTGCCATTTATATGTTGGCGCTATTAATCTTTGCCA
TGTTTGCTCTTGAGTGCTTCCTGTTCTTACTACTCCTTCTTTATATGCCACATAGAAATTTTTCCATATT
GGGTCTGTCCACCAGTTGTAATAACTTGTTTCATATAAGTTATCTTGCATTCCTATACTATAGATAGTAT
TATTGAAAGCTGTGAAGGTACTGGTTGCTTGGATAGCGAGGTTTTGTGTTAATGGTATTGGGTTATATAT
TGTTATGGTGTATCCTTCTACATGGTATGCTTCATAATNNNNNNNNNNNNNNNNNNNNNNNNNNNNNNNN
NNNNNNNNNNNNNNNNNNNNNNNNNNNNNNNNNNNNNNNNNNNNNNNNNNNNNNNNNNNNNNNNNNNNNN
NNNTATTTGTATGCTTGGAATTCTGTTCCTGGCCATCCTAGTTGTGCTTTCTTTGTATCTGGTAAGGTGA
CGAGGTTTCTGTTCATTTCTGTCCAGAACCAGGATACTGGTACGATTGGTAGATTTAGTAGGTTTGGCAT
GTCATATCCGAGGTATCTATTGTTGTATTGTGGTTCTGGTCTTCGTGTTTGGCCTGCTCCACTGGTGTAT
GAGGTTTGTGGGAATGGATCAGACATGTCATTTTGTGGAATGAAGCTTCCTGTTGGTCCATATCTGCTTG
TTTCTCCTTTACTGCTTGTGCTTGTTTCTCCTAACCATGGTCCGTCTGGGACATAAGGTAGGTATTTTGC
TAGTAAATCTAGATTGAACCATATGTTTTCGTCACATGGATGGTTGTTCCAATGGAAGCTTACGCTGTTT
TTTCCTGGTCTTAGTTCTAGGATGCTGTCTGGGTCTGTTAGAGGGTCCCAAAATATTCCTGTTGGGTGCG
CAATATTTGGAGTTCCGCGATTGGTGTGAGGCCACACGCTGGTGGCTTGTGGGTAGTCTGGTCTGGTTGG
ATCCCATGACCAAGTGATGGTGTCATATGGATCGTGGTCTGGAGTTTGCCATAAGTATGTTGGTAGTAAG
AGTCTCCTTTGTGATGTGGTTTCGGTTCCATATTTGATGNNNNNNNNNNNNNNNNNNNNNNNNNNNNNNN
NNNNNNNNNNNNNNNNNNNNNNNNNNNNNNNNNNNNNNNNNNNNNNNNNNNNNNNNNNNNNNNNNNNNNN
NNNNCGAGCATCGAGGACGTGATGATGCAGATCGCGGAGGAGCTGGGGGAACAGGGGATGGAAGTGGACA
GCCAGATCCTGACCAGCCTGGATCTGATAGAAGACAACATGAGGACCTACGCGAGGTGGTGGAGCTGGAC
GCCGGGCAGAAGAGCGGGCATCAAATGGAAACCCAAGAATCAGAGCTGGGTGGGGAGGTGGGTACCCTAA
CAATCCCCACCATGGTGGACTGGTGTAAATACTTCTCCTTTCTCTATTCGCAGTTTTCCAAATAAACGAC
CATGGCAGAATCCATCTCACTTGGCAATAATTATATGTGTTACTGGGACAACTCTCCCTACGTATACGGA
TCACCAGACTTCAGTCAAGTAAACACCCTCACATCAGTCAATACCGGATGGCATGTCTTATCTACCATGC
TATGGAAACATTTTCTCTCACCCAAACAGTGGTATGAAATGATGATTCACTATGAAGCATACCACGTAGA
AGGCTACACTGCAACACTATACAATCCTATTCCACTTACACAAAACCTTGCCATCCAAGGCACCAGTACC
TTCACAGCTTTCAATAATACCATATATACACTAGGAGCTCAAGACACATTATATGAAACCAGCTATCATA
ACTGGTGGACAGACCCTCTATGGAAAAACTTCTATGTAGCATACAAAGAAGGCTTCATCAAATATGGAAC
CACAACAGTAACACAAAAAAGACTWTTACTACCAACATATCTWTKGCRAACTCCAGRCCATGATCCATAT
GWTWYATACACCTGGTCATGGGATCCAACAAGACCAGACTATCCACAAGCCACCWKMKYATGGCCACATA
CCAACACCGGCACGCCCAATATAGCTCATCCAACAGGAATATACTGGGATCCACTAACAGACCCAGATAG
CATCCTAGAACTCAGACCAGGAAAAAATTCAATGACATTTAATTGGAGTGCTCACCCATGTGATGAAAAC
ATCTGGTTCAACCTAGACCNNNNNNNNNNNNNNNNNNNNNNNNNNNNNNNNNNNNNNNNNNNNNNNNNNN
NNNNNNNNNNNNNNNNNNNNNNNNNNNNNNNNNNNNNNNNNNNNNNNNNNNNNNGATAAGAAGATGAAGT
MTATTTCGTCTATGTCTAGTCCTTGGTGGCTGAGGCATGTGTGTATGGCTGTCGGATCTGCTTGGTATCT
CTGTGAGTTTTGTTGTAGTGACCACTCTCCTTTTGTGGCTTCCACGAAGGAGAGGCAGTTTTTTATGATC
TGCATGAACCCTGGTTTGTGTAGGTGTTGCACTATGATGTCTGGGTTGTGTTTGAATATGTCTTCTGCCG
TGAGGCAGTTGTGTGTGTATATGATGTTGAGGATGTCTTTTGTGATGGCGTGTCCCCCCTGGAGTACTTG
CTGCGGTAGGATCTGTTTTCTCCTTCTSTCTTCTTCTTTTTTGTCTAGGTATTTCTGTCCCGCGTTGAAG
TAGTACAGCGAGCAGGCGTAGCGCAGGTTTGTCGGGTTGAAGCCGCAGATGAAGAGCGGGTTCTTGACCA
TGTAGCTGGTGAGCGCCGAGGGTCTGTGAGCTTTCTGGCACTTGCAGATCTCTAGCGAGGGATCCGGGTC
TTCTATGTCTTCCATGGCCGCCAGGCGGCACTGTTCCCATTTTGTTTGGAGCGCCCTCTGCAGGCTGTCT
GCTCTCATCAGCGTCTTGCACAGGACGTGATAGTGTAGGATGTTTTCGTCGTTTTTCTCTCCCACCACTA
TGAAGTCCTGTACGTTCTTGATGCTGCTCATAAGGAAGGCGATGGGGCGTGGATCTTCTATATCCAGCAC
GCTGCCATCTCCCTTCCCTATGACCAGCACCAGACCCAGCCACGTCTGCATGTTCATGTTGGCGGTGTGC
GTGTCTATCTCCGCGCTGGACATGACCCAGGTGCTCAGAACCTCTGTNNNNNNNNNNNNNNNNNNNNNNN
NNNNNNNNNNNNNNNNNNNNNNNNNNNNNNNNNNNNNNNNNNNNNNNNNNNNNNNNNNNNNNNNNNNNNN
NNNNNNNNNNNNCCAACATTAGAGATCTGCAAATGCCAAAAAGCACACAGACCAGAAGCGCTCCTGTGCT
ACATGGTGAAAAACCCGCTCTTCATAGCAGCACACAATCCAAAATCACTACAATACGCATATTCACTCTA
CTTCCATAACAAAGGCCAGAAATACTTAGACAAAAAAGAGGCAGAAAGACAGAGAAAACAAACACTCCCA
ATAGAAACCATATCAGGAGGACACCCAATCACCAGAGACATCCTCAGCATCATCTACAAATACAACTGCC

TGACATCAGAAGACATCTTCAAACACGAGCCAGACATAATCATCCAACACCTACACAAACCAGGCTTCGC
CAACATAATCAAAAACTGCCTAGCCTTCGTAGAGGCCACCAAAGGCGAATGGTCACTAGAAAAGAACGCA
CACAGACACAAACCAGACCCCACAACAGTACACATGTGCATCAACCATCAGGGCCTAGACACAGAAGAGG
TAGATTACATGCTCTATCAATGGATCTCTAAATCACACCCAAAGAAAAACACCGTCCTCCTCATAGGACC
CAGCAACACAGGCAAATCAGCCTTCATCAGAGGTCTCAGAGGAGTCCTAGAAACAGGAGAGATCTGCAAC
GGGCAAGTATTCTGCTTCGAGGGACTAGTAGGCAAAAAGCTAGGCATATGGGAGGAACCTCTAATCAGTC
CAGAGTCAGCCGAGAAAGCCAAACAAGTATTCGAAGGGGCAGACACCTCAGTACCAGTAAAATACAAAAA
ACCACAACCACTAGGCAGAACTCCNNNNNNNNNNNNNNNNNNNNNNNNNNNNNNNNNNNNNNNNNNNNNN
NNNNNNNNNNNNNNNNNNNNNNNNNNNNNNNNNNNNNNNNNNNNNNNNNNNNNNNNNNNTACATCCTCGG
CGACAACGAGATAAGTTCACACTCGGCCACCCTAAACATGCAAACCTGGCAGGGGGTTGTCATAGTCGTC
GGAAGGGGGGACGGCACCATCCTAGACCTTGACAACCCCAGACCGATAGCCTTCTGTCTCAGCAGCATCA
AAAACATCCAAGACTTCATCATAGTGGGAGAGAACAACGACGACGGCATCCTGCACTTCCACTGCCTCTG
CAGAACCCTGATGCGAGGAGACAGCCTACAAAGAAGCATCCTAAACAAATGGGAGACGCACAAACTAGCA
GCCATGGAAGACATAGAAGAACCAGAACCCACACTGGAGATCTGCAAATGCCAAAAAGCACACAGACCAG
AAGCGCTCCTGTGCTACATGGTGAAAAACCCACTCTTCATAGCAGCATACAATCCAAAATCACTGCAATA
CGCATACTCACTCTACTTTCACAACAAAGGCCAAAAATACTTAGACAAAAAAGAGGCAGAAAGACAGAGA
AAACAAACACTCCCAATAGAGACCATAGCGGGGGACACCCGATCACCAGAGACATCCTCAGCATCATCT
ACAAATACAACTGCCTGACATCAGAAGACATCTTCAAACACGAACCAGACATAATCATCCAACACCTACA
CAAACCAGGCTTCGCCAACATAATCAAAAACTGCCTAGCCTTCGTAGAGGCCACCAAAGGCGAGTGGTCA
CTAGAAAAAAACGCACACAGATACAAACCAGACCCCACAGCCATACACATGTGCATCAACCATCAGGGCC
TAGACACAGAAGACATAGATTACACATTCTATCAGTGGATCACCAAAAAACACCCTAAAAAAAACACCAT
CCTTCTCATAGGACCTAGCAACACAGGCAAATCGGCCTTCATCAGAGGCCTCAGGGGAGTCTTCGAGACA
GGAGAGATATGCAACGGGCAGGTATTCTGCTTCGAAGGCATAGTGGGCAAAAAAATGGGCATATGGGAAG
AACCTCTAATCAGTCCAGAGTCAGCAGAGAAAGCCAAACAGGTATTCGAAGGGGCAGACACCTCAGTACC
AGTCAAATACAAAAAACCACAACCACTAGGCAGAACTCCCATAATCATCACCAGCAATCACTGGCCGTGG
CGCTACTGCACCGCGGAAGAAAACCCGTTCAGGAACAGGATGTTCATCCTACCCTGGAACCACTCCTCTG
ACCCTCCTCNNNNNNNNNNNNNNNNNNNNNNNNNNNNNNNNNNNNNNNNNNNNNNNNNNNNNNNNNNNNN
NNNNNNNNNNNNNNNNNNNNNNNNNNNNNNNNNNNNNNNNNNNNNTATACCGTGCCGGACCCGGGATGGA
TTCCATGTGGAGCCCCTCGAGCATCGAGGACGTGATGATGCAGATCGCGGAAGAACTGGAGGCGCAAGGG
ATGGAAGTGGACACACAGATCCTGACCAGCCTGGACCTGATAGAAGACAACATGAGGACCTACGCAAGAT
GGTGGAGCTGGACGCCAGGCAGGAGAGCGGGCATCAAGTGGAAACCCAAGAATCAGAGCTGGGTGGGGAA
ATGGGTACCCTGACAATCCCCACCATGGTGGACTGGTGTAAATACTTCTCTTTTCTCTATTCGCAGTTCT
CCAAATAAACAATTATGGCAGAATCAATCTCACTTGGCAACAATTATATGTGTTACTGGGATAACTCTCC
CTATGTATACGGATCGCCAGACTTCAGTCAAGTTAACTCCCTCGCATCAGTCAACACCGGATGGCATGTT
TTACCTACCATGCTATGGAAACACTTTCTCTCACCCAAACAATGGTACGAAATGATGATTCACTATGAAG
CATACCACGTAGAAGGCTACACTGCAACACAATACAATCCTATTCCACTCACCCAAAATCTTGCCATCCA
AGGAACCAGTACTTTCACAGCCTTCAATAATACCATATACACATTAGNNNNNNNNNNNNNNNNNNNNNNN
NNNNNNNNNNNNNNNNNNNNNNNNNNNNNNNNNNNNNNNNNNNNNNNNNNNNNNNNNNNNNNNNNNNNNN
NNNNNNNNNNNNATATGGTGGGGGTGAGGGTCCCTCCGGCAAAACAAGCTCGCATCGAAGCAGAAAGCGA
CGAACCTCCACCACTAGAGGGCGAAGAAGGGGATCCAGACAAAGACAACTACGCAGGTGAGTAACCATAT
GTTTCCTACTCTCATTCCAGAATGGCGGGCACAGGACAGGGCTTCTCCCTACTACTAGAATGCATACAAC
ACCCTGAGGGTTACGAGTCAGATCAGGCAGTGTTAGAGGACGCCATCCTACTGATCAACGGACGCTGGAA
CCTAGAAGGCTCAGTCATCGTCGACGATGACGGCAAACACTGGGGCTGGGTCGTATGCACGCGCTTCCTA
GTATCCACAGACACGGTAAGACGAGCCCTGGGTGATATGGTTCCAGATTACGTGAGGTTCTACAGAGGCA
GCGCCACGCAGACATCCGGACAAGCCATCAAACTGGTGAAACTCAAAAGAAAATGGAACGTGGAGGACAC
CCAAGAAGCACAGAGCTCCGAAGTGTCAGGAAGTGGCTCTGGACAGGGTCCGCTCTCGTGGAAGAGACCA
CGCTACTGACACACTACATCCTCGGCGACAACGAGATAAGTTCACACTCGGCCACCCTAAACATGCAAAC
CTGGCAGGGGGTTGTCATAGTCGTCGGAAGGGGGGACGGCACCATCCTAGACCTTGACAACCCCAGACCG
ATAGCCTTCTGTCTCAGCAGCATCAAAAACATCCAAGACTTCATCATAGTGGGAGAGAACAACGACGACG
GCATCCTGCACTTCCACTGCCTCTGCAGAACCCTGATGCGAGGAGACAGCCTACAAAGAAGCATCCTAAA

CAAATGGGAGACGCACAAACTAGCAGCCATGGAAGACATAGAAGAACCAGAACCCACACTGGAGATCTGC
AAATGCCAAAAAGCACACAGACCAGAAGCGCTCCTGTGCTACATGGTGAAAAACCCACTCTTCATAGCAG
CATACAATCCAAAATCACTGCAATACGCATACTCACTCTACTTTCACAACAAAGGCCAAAAATACTTAGA
CAAAAAAGAGGCAGAAAGACAGAGAAAACAAACACTCCCAATAGAGACCATAGCGGGGGACACCCGATC
ACCAGAGACATCCTCAGCATCATCTACAAATACAACTGCCTGACATCAGAAGACATCTTCAAACACGAAC
CAGACATAATCATCCAACACCTACACAAACCAGGCTTCGCCAACATAATCAAAAACTGCCTAGCCTTCGT
AGAGGCCACCAAAGGCGAGTGGTCACTAGAAAAAAACGCACACAGATACAAACCAGACCCCACAGCCATA
CACATGTGCATCAACCATCAGGGCCTAGACACAGAAGACATAGATTACACATTCTATCAGTGGATCACCA
AAAAACACCCTAAAAAAAACACCATCCTTCTCATAGGACCTAGCAACACAGGCAAATCGGCCTTCATCAG
AGGCCTCAGGGGAGTCTTCGAGACAGGAGAGATATGCAACGGGCAGGTATTCTGCTTCGAAGGCATAGTG
GGCAAAAAAATGGGCATATGGGAAGAACCTCTAATCAGTCCAGAGTCAGCAGAGAAAGCCAAACAGGTAT
TCGAAGGGGCAGACACCTCAGTACCAGTCAAATACAAAAAACCACAACCACTAGGCAGAACTCCCATAAT
CATCACCAGCAATCACTGGCCGTGGCGCTACTGCACCGCGGAAGAAAACCCGTTCAAGAACAGGATGTTC
ATCCTACCCTAGNNNNNNNNNNNNNNNNNNNNNNNNNNNNNNNNNNNNNNNNNNNNNNNNNNNNNNNNNN
NNNNNNNNNNNNNNNNNNNNNNNNNNNNNNNNNNNNNNNNNNNNNNNNATAGAGGAGAACATGGTAGCCTA
CAGCAGATGGTGGAGCTGGATAATGGGGCGGAAGGCGGGCGTGAAATGGAAACCCAAGAATCAGGCATGG
GTGGGCAAATGGTGTCCCTGAAAATCCCTACCAGCAATGACTGGTGCTCTTATTTCTCTTTCCTGTATTC
GCAGTACAAACAATAAAATGGCCGACTCGGTGTCGCTGAGCAGCAATTTCATGTGTTATTGGGATAACTC
ACCATATAATTATACTACATCACAACAAGCACAAATAAATTATGCCACCACAGTGGACACAGGTTGGCAC
GTTCTACCTAATATATTATGGAAACATTTCTTATCTCCAAGACAATGGTACGAGATGTGTATCAAATACG
AAGCTTACCACGTCTCAGGATATACTACCACATTATACAATGCAATACCTCTCACACAAAACCTAGCCAT
CCAAGGCACAAGTACATTCACTGCTTTCAATAATACCATCTACACCCTGGGAGCACAAGATAACCTTTAC
GAAACCAGCTACCATAACTGGTGGACAGATCCTATCTGGAAAGATTTCTACGTTGCATACAAAGAAGGTT
TAATAACTTCCGGACTACCTGAGGCTGCAACGCATAAAAGAGCATTACTCCCAAAATACTTATGGGAAAT
AGCAGATACAGATCCATACGATACCACTACATGGTGCTGGGACCCAGTACCACCACAATATCCACCAGCA
TACACTACATACCCACATACCAATCAACAAAATCAAATAGCAAGACCGTCAGGCGTATTCTGGGACCCAC
TAACAGACCCAGATTCAATCATGGAATTAAGACCAGGAAAAAACTCCATGTCATTCCATTGGAACAATCA
TAACTGCGATGACCACATTTGGTACAACCTAGACACCATGGCAAAACTACTACCATACGTACCAGAAGGA
CCATGGCTAGGAGTCACAACACAAGATGATACCTATTCAAGATATGGAGAAAATGGAACATACATACCAA
ACAGAGATTTCACAGATCCATTTCCACAAACATCTTACACAACAGGAGGACAAGCCACTACCAAACCATC
CATATACTACAAAAACAAATACCAAGACTATTCAGTACCAAACTTACTAAACCTACCAATAGTACCAATC
AAATGGTTCTGGGTAGAAGTTAACAAAAACATGATTGAAAACCCAGATCCAAACAAACCACAACTAGGAT
GGCCAGGAACAGAATATGAATCCTACAAATATGGACCAACACAATGTTTTGTTAAAGGAATTCCACTATT
TGACGAAAATGACACTCTAATACATACAAGCACACAAGGAATAATAAGAAACACAATACACCTAAAACTC
AAAAAACGAAGATCCAGAATATTTGCTCCAACATGGGGACCCATGAGTTGGGAAATGACAAGTACCATAA
AAGCACCATTTGTATTACCAACCATCAGATACAGAACAGCAGGCGCAAGAAGAGGATGGCAAAACAGAAC
ACAAAATCCAGACTATAAACAACCAACCAGATGGAATCCATATACAACAACTACATACACATCGACTACA
ACAACTAACTCAACTACATCAACACTATCATATACTCGCAAATAAAGCAACCGTGTCTTTACAACCACAC
ACACAACTATNNNNNNNNNNNNNNNNNNNNNNNNNNNNNNNNNNNNNNNNNNNNNNNNNNNNNNNNNNNN
NNNNNNNNNNNNNNNNNNNNNNNNNNNNNNNNNNNNNNNNNNNNNNNNATTATGGGAGTTCTGCCTAGTGGTT
GTGGTTTTTTGTATTTGACTGGTACTGAGGTGTCTGCCCCTTCGAATACCTGTTTGGCTTTCTCTGCTGA
CTCTGGTCTGATTAGAGGTTCTTCCCATATGCCCATTTTTTTGCCCACTATGCCTTCGAAGCAGAATACC
TGCCCGTTGCATATCTCTCCTGTCTCGAAGACTCCCCTGAGGCCTCTGATGAAGGCCGATTTGCCTGTGT
TGCTAGGTCCTATGAGAAGGATGGTGTTTTTTTTAGGGTGTTTTTTGGTGATCCACTGATAGAATGTGTA
ATCTATGTCTTCTGTGTCTAGGCCCTGATGGTTGATGCACATGTGTATGGCTGTGGGGTCTGGTTTGTAT
CTGTGTGCGTTTTTTTCTAGTGACCACTCGCCTTTGGTGGCCTCTACGAAGGCTAGGCAGTTTTTGATTA
TGTTGGCGAAGCCTGGTTTGTGTAGGTGTTGGATGATTATGTCTGGTTCGTGTTTGAAGATGTCTTCTGA
TGTCAGGCAGTTGTATTTGTAGATGATGCTGAGGATGTCTCTGGTGATCGGGTGTCCCCCCGCTATGGTC
TCTATTGGGAGTGTTTGTTTTCTCTGTCTTTCTGCCTCTTTTTTGTCTAAGTATTTTTGGCCTTTGTTGT
GAAAGTAGAGTGAGTATGCGTATTGCAGTGATTTTGGATTGTATGCTGCTATGAAGAGTGGGTTTTTCAC

CATGTAGCACAGGAGCGCTTCTGGTCTGTGTGCTTTTTGGCATTTGCAGATCTCCAGTGTGGGTTCTGGT
TCTTCTATGTCTTCCATGGCTGCTAGTTTGTGCGTCTCCCATTTGTTTAGGATGCTTCTTTGTAGGCTGT
CTCCTCGCATCAGGGTTCTGCAGAGGCAGTGGAAGTGCAGGATGCCGTCGTCGTTGTTCTCTCCCACTAT
GATGAAGTCTTGGATGTTTTTGATGCTGCTGAGACAGAAGGCTATCGGTCTGGGGTTGTCAAGGTCTAGG
ATGGTGCCGTCCCCCCTTCCGACGACTATGACAACCCCCTGCCAGGTTTGCATGTTTAGGGTGGCCGAGT
GTGAACTTATCTCGTTGTCGCCGAGGATGTAGTGTGTCAGTAGCGTGGTCTCTTCCACGAGAGCGGACCC
TGTCCAGAGCCACTTCCTGACACTTCGGAGCTCTGTGCTTCTTGGTGAAAAACCCACTCTTCATAGCAGC
ATACAATCCAAAATCACTGCAATACGCATACTCACTCTACTTTCACAACAAAGGCCAAAAATACTTAGAC
AAAAAAGAGGCAGAAAGACAGAGAAAACAAACACTCCCAATAGAGACCATAGCGGGGGGACACCCGATCA
CCCGAACCTATNNNNNNNNNNNNNNNNNNNNNNNNNNNNNNNNNNNNNNNNNNNNNNNNNNNNNNNNNNNN
NNNNNNNNNNNNNNNNNNNNNNNNNNNNNNNNNNNNNNNNNNNNNNTCCATATACTACAAAAACAAATAC
CAAGACTATTCAGTACCAAACTTACTAAACCTACCAATAGTACCAATCAAATGGTTCTGGGTAGAAGTTA
ACAAAAACATGATTGAAAACCCAGATCCAAACAAACCACAACTAGGATGGCCAGGAACAGAATATGAATC
CTACAAATATGGACCAACACAATGTTTTGTTAAAGGAATTCCACTATTTGACGAAAATGACACTCTAATA
CATACAAGCACACAAGGAATAATAAGAAACACAATACACCTAAAACTCAAAAAACGAAGATCCAGAATAT
TTGCTCCAACATGGGGACCCATGAGTTGGGAAATGACAAGTACCATAAAAGCACCATTTGTATTACCAAC
CATCAGATACAGAACAGCAGGCGCAAGAAGAGGATGGCAAAACAGAACACAAAATCCAGACTATAAACAA
CCAACCAGATGGAATCCATATACAACAACTACATACACATCGACTACAACAACTAACTCAACTACATCGA
CACTNNCATA

>Simian erythroparvovirus
AGTTAAAGCTGTTTTAATTTGATGAACCCCAGCAACTGAAGCACTTAGAGAAGCATACTCTTTAAAGTCT
ACCTCTTTCCATTTACTTTCATTAAATATATTCCTTTCAATTAAAGTGTCTACCAGAGAACAGAACTTTT
CTCCTTTCACAGTAACCACTTTAGACTTTTTTGCTGCAGGTTCTTCAAAACTTATAGAACTGTTCCCACT
CGCCTCATTGCTTATACCTGGCCCCTGCAGCTTTTTGTGCATAGTTCTTCTATAGCTTAAAGAATTACAT
ACATACTGATATTCATCCAAGTTAGTTACACTGTAAATTACATTAGGGTACAACTTAGGCATTAAGTAAT
TTGTAACAAAGTCAAAACCATCCCTGTGATATTTTCCTTTTTTAGTAACAGCAGGTTTAAACTGAACACA
AATTGGAGGTGATGTAAAATCAGCAAGCACTTTGTTAAAACCCCCATCAACCACACTACACACATTTCTA
GGAGTTATAAAGGGACCCCCTATAACAACATGAATATGAAAGCCATTATCATAATGATTGCCTTCAGCTT
GTAAAAAATACTTAGAGCCCAAAAAAGGCCCAGAAGGATTGTCTAACAGAGAAGCCACTTTACAAATAAG
GTGAGCTATAAGCCGCTCCGCTCCTCGGAGCTCTGGCCAGTCGTCATCATCTAACTGAAAAAAGCAGCAC
CACCAGTTATCGTTAGCAAAGTCAGTAAAGTTAGCATTCACCTGAATAACTCCTCTATACATCTCCATTG
TTAGTGAAAGCAGTAAGGTTAATTACCTGTTAGTAACAGAGCAGCAGCGTTATGCTCTCTCCGAACACCA
GCAAGCAAATGGACGAACACTTCCTGGTTTTGGCAATATATAGAGCAAGTGAACCAACCAGGCTGGAGAA
TTATTTAAAGTAGCCAATCAAAATACAGTAAACAACAAAATGGCCGCCAGTCAAAATGGCCGCCAAACTG
TCATCATTTCCCCTTGAGAACGACACCAAATCAATAGNNNNNNNNNNNNNNNNNNNNNNNNNNNNNNNNN
NNNNNNNNNNNNNNNNNNNNNNNNNNNNNNNNNNNNNNNNNNNNNNNNNNNNNNNNNNNNNNNNNNNNNN
NNNNNNNNNNNNNNNNNNNNNNNNNNNNNNNNNNNCACTGGAGKATAHGCGGAACACAGCCGGAAACATT
GAATTAAATGCCTTTCCTGGGGACTTAGAGCTCCATACATAGAGGGCGGCGGATTTCCTAAAGGACACAC
GTAATGTTTAAAGAGGGTGCACTCGCTTTTTGGCGCTCCAGCATTCTTCTGGTTTTTCATACCCGTGTCT
GTGGTTCTGCGAGTTTTTGGTAACCTCGGGGTCGTACAATATGTAGGGTAAATGTCCTGCAGCGTGGGGA
GGGATACACGCGGGCTGGGGATTCCATCTTCCCGTGTGTTTTCGTGGACCTAAAGCAAATGTAAGTGTTA
CTGTTAACACCCCGGTGGCATACTGAGCTAGGGTACTATTGGTAATACTGGCAAAGCCTTCAGGCCCTGG
AGCTGGTTGCATTTTTAGAAATATCATAGGAGGGGGCTTAGGTAGAGCCCAGCCACCTAAAGCTGAAAAT
TCAGTTTTAAAGCCTTCATCTAAGTTAGGAAGCTTTGTCCAGAGCTGTGTTTCATAGTGAAAAGCTCTTC
TATTCCATATAGCTCCTACCATTAGAGGCCTGTTAATTTCTTCAGTGTACTTTTGGTCTCCTTTGTTACT
ATAGAAAGTTTTAATATTTAACCCTTGTAACTGCTTATTTTGTTCTTTGTCATTTGGGTACCGTCCCACA
GCTTGAGAGGCTTCTGTTGGTTTTGCTGGATTACCAAAAGTTGTTACACCATAGCTTATGGAATCAATAC
CATTTACATACTTTTGATTTATTTCATCATAGTAATGGTAAGGTTGAGAGCGAGGCCCCGGTCTGAAAGA
TATTCTGGTATTTTGTGAGCTGCCTGTAGACATGCCTGTGAGTGCTTTAGCCGCACCTGTGTTGCTTGAG

TCTCCGTCTGCAGTGGTGACTGTGTTAACTAAGGGACCTGGCATAAAGTTCTGAGGTTGTATGGCATGAT
CTGTGGGCTGCACAAAGCGGACTTTTGGCTGGCCACCTAGGGCAGAGGGTACAGCTAATCTAGAAGAGTA
TAAAGGATTATACATTTCATAAAAGTGTTGAGAAGCTCCCTCTAGAGACTCAGCTGTTAGTGGTGGAAAA
GTGTAAGAAGTGCTGATGCTAGACCCAGTTCCTAACAGTAAGCAGTTTGCGTGCTCTAGTACATAGAAAG
CTGACTCCTCAGATGGCTGTTTGCGAGTGCTAGATGTTAACCCTTTTGTGTTTACTTCTCCTACAGTTAA
GTATGCATACTGTGGTAGTAGGTAGGTCCAAATAGGTAACTCTGGAGCTAAAGTGTCTTGCCCCTGTCCC
AGTACATAAGGAAACTGGTATTCATCATCCACTAAGAAACACAATCTACCCGTTGTGCTGTCAGTGACTT
GTACTCCCCCTCCTGGTTTGTCTGTTACATCTTTTATGCAAACTTCACTAAGTGTAACACTCATAGAAGA
TGGTTTTATAGATCCATAGTTTTCTAACAGTCTCTGAAATTCTAAAGGAGAAAAGTACAAAGCGGCACAG
TTTACATCAATGTAGTGCCAGGGGGTAGCATACCCCATGACTGGACTAATAGCACAAACGTTGCTTTCTC
CAGTGGCTGCTTTATGACAGTTTTCTGCTGGAGGAGAAAACACTCTATAGGCATGCTCAGCTGTATAGGG
CACTACAAACACCCTGCTAAAAGTACATGTTACAGAAGAATCAGTAAACACTGCTCCTTCACGCCACACT
CCGTGTACCGGGTTACTGCCGCCTCCTCCAGCTCCAGTAGTGCCTTCAGTAGATGTAACAGATGCCATGT
TTGGGTAGTTTTCAGACGGTGAGTACTGTGGAACTGCAGGAAGCTCTCCTTTAAAGTGAGCTGTAGAAGT
AAATAGGCCTTTTAAAGTAAAAAAATCTCTAACTACTTGTGCTTGAAAACCACTTTCATTTTTTATGTTA
TGCAGCAATTCATCATCAGCAACAGACCAGTGAGTATAAGGGTTTATTCCCAATTTAATAAGTTCAGAAT
ACCTGAAATCATGAATCCTAGCTGCAGCATCCACCACACTTTGTGGATTCCCAGCTTGTAACTGATTACC
AGGGCCAATGTAGTTACTAAAGGGTAGGACAAGACTAAGTCCACCCCCTTCGTGTAGGTCTTTTTCAGTA
AGGACTGCGTTGTCTGCTCTAGGCTCATTAGAGGATTCATGTTCTGACTGAGTTTCTCTGAAATCTGGAA
GAGGTCCATGAGACAAATCCAAAGTATACCTATCAGGACTATTTTTATAGTTTTCAAGATGAGTTTGTAA
ATAAGTGCCAAGAGTACTTTTGCCTTCACTATCTTTATTAATAGCATTCACAGACAGTGCATTGTCTGTT
AAAAATTTTAAAGCATCACTCGAGGTTAAAGCATCAACACCATACTTTAAATCTAACTTTAAATGGCTTT
GCAAAAACTTTAAGGCTAGCACTGGACCTTCTGCTTCTTCACCTATTAAGCTAATAGCATCTTTTACTAT
ATCTTTAAATTCAGACACCCAAGCATCAGTGTAAGCATTTTCTTCATTCCACCACACTTTTTGTTTTTTT
GCTGCAGGTTCACTCATGCTCTAGAAAGCTCATAAGGCCACTGAGGTCTCTGCATTTCTTACAAGTTAAT
ACATCAAATGGATTTGTGCAATCACCATGAGCTGAGCACCTCAGAGTATCTGGCAGAGTAACACTCTTCT
CTMTNWMKCWTANNNWACMCTTYTAAAGATAAACACAAATTACAATGCACACCAAACTCCTTATCATGCT
CCAAACAGCACACAGGACGCCCAACTGCTGAACCCCACACCCAATCAACATCAAATAACGGCCACTTCAG
TTCATTGAGTTCATGTACTGAATAATTTTTTTCCCACTTTAATTCTCCTCCTGCTCCGGTGGGAACTTGG
ACTGGCTCAGCCACTGCCACTGGGGTGCTGCAGCGGGCCGGGGGACAGCTATCCTCGCTGAGGTCTTCGA
TCCAGTTGTCGTTTGAGACGAGGTCTGCGATGTCCCAGCTGACATTAGAGTTTTCAGAACCGCCTGACTG
ATCTTCGACGTCAAGCCAGGAGGTTGAGAGGTCAGTTCCGTCAAAGCTTTCGATGGACTCTTCGTCAGCG
ACTGAGTCTGAGGATGAGTTTGGCTCCGGCTCACTCTGGAAGTCATGACACAGCTCTGTACGGTCCACCT
TCCAGGCAGTGTGATCGCTAGAGAGTTGCCACTGAACAAATGCATCGCAAGCATTATGCTGTGCCCACCA
AAGCCATTGTTTGGCCTCATCAGCGGGAATCAATCCCAGAGCATTTGAGCATCTCACAGTCAGGTCAAAC
CGAACCATGCGATCCTTCAACGCTCGCTGATGAACAAGGGTCACAGTGTTCCCGCAAACCACTCTAGTCA
TGTCACTGTTACTAGTGATAAGGACCGGTGTGCCAGTAACCTCTACGCTGCCTTTGTTTTTTTGGTCAAC
TCTACANNNNNNNNNNNNNNNNNNNNNNNNNNNNNNNNNNNNNNNNNNNNNNNNNNNNNNNNNNNNNNNN
NNNNNNNNNNNNNNNNNNNNNNNNNNNNNNNNNNNNNNNNNNNNNNNNNNNNNNNNNNNNNNNNNNNNNN
NNNNNNNNNNNNNNNNNGATAGGTTGGTTAGTAAAAAAATGACTTACATGGTTAGTATGTTAGATTGTGT
AGTTAGGCATTGCTCTTCCTGGCATGTATGGAATTTCAAAGTTTCCAATTTCATTTGGTACAGTTTCTTT
CATGCTTTCTCTTGGAGCGAGTGATGGTAGTTGATAGGTGTTCCATTGTCTTGGAGTTCTAAGTTTGGCT
TTGATGATTAGTTTTCCTGTCCACCAAAAGGTTCCAAATGTAACTATTCTTGATGAATTGCCATTGTTGT
CTACAGTGTCTGTGTAGTTTGGAGCTAGTTTAACAAAGATTTGTCCTGGTGGATTTGGTGTCATGAATGG
AGCGTGTGCTGATGCCATTGGTTTGTGAGTTGTGTTTGGTTCTTTTCCCCATATGGCTCCCCATGGGTAT
TGTGCTCCATGGTCATCAAATGCTGTGAATGGTCCGAATGTGTTGTGGTAGCCTTGTGTCCAGAATGCGT
CTCGTCCAGCTACATTGTTGTTGAGTTCTTTTTGGTGTATGTTTTTTGGTGCCCAGTTGGTTTGTCCTGT
CATTTGTTGATTGTTTTGCCACCATGTTTCTCTGAATTGGTGTTCTTCATCACCATGGTTCCAATCATAT
ATTGCTTTTTCAGAAGCTCCTTGTGTTAGTCTTGTGCCGGCAAATA
\

```
}
```
