# Supplementary material for: Enteric Viruses Nucleic Acids Distribution along the Digestive Tract of Rhesus Macaques with Idiopathic Chronic Diarrhea
Source: Viruses. 2022 Mar 19;14(3):638. doi: 10.3390/v14030638 (PMC8951234; doi:10.3390/v14030638)
Supplement: Supplementary file 1 [file viruses-14-00638-s001.zip › Table S1.pdf]

Supplemental Table S1. Composition of concatamers used to quantify reads matching different viral species and genotypes.

Best virus match	animal	location	sample type	Best virus match	length	% identity	Accession no	% coverage of genome
bocaparvovirus	46481	distal	tissue	bocaparvovirus	1445	78	KT961665	26
chaphamaparvovirus	45629	ileum	tissue	chapparvovirus	782	76	MN312221	80
chaphamaparvovirus	46700	distal	tissue	chapparvovirus	839	84	MN312221	
chaphamaparvovirus	46481	ileum	scraping	chapparvovirus	675	95	KT961662	
chaphamaparvovirus	45349	jejunum	tissue	chapparvovirus	917	77	MN312221	
chaphamaparvovirus	45349	jejunum	tissue	chapparvovirus	666	78	MN312221	
chaphamaparvovirus	45524	jejunum	tissue	chapparvovirus	995	84	MN312221	
chaphamaparvovirus	45524	jejunum	tissue	chapparvovirus	833	89	KT961661	
chaphamaparvovirus	46481	jejunum	tissue	chapparvovirus	1743	76	MN312221	
chaphamaparvovirus	46481	proximal	tissue	chapparvovirus	3393	73	MN312221	
chaphamaparvovirus	46481	stomach	tissue	chapparvovirus	1890	74	MN312221	
chaphamaparvovirus	46481	duodenum	scraping	chapparvovirus	2427	74	MN312221	
chaphamaparvovirus	45349	jejunum	tissue	chapparvovirus	1046	75	MN337628	
dependoparvovirus	47210	stomach	tissue	dependoparvovirus	732	97	DQ180605	79
dependoparvovirus	47210	stomach	tissue	dependoparvovirus	2754	92	AY631966	
enterovirus A SV19	45349	distal	scraping	enterovirus 19	4035	91	KT961653	98
enterovirus A SV19	45349	distal	scraping	enterovirus 19	2870	85	KT961654	
enterovirus A SV19	46776	distal	scraping	enterovirus 19	1676	86	KT961653	
enterovirus A SV19	46776	transverse	scraping	enterovirus 19	5983	85	KT961654	
enterovirus A SV19	47210	distal	scraping	enterovirus 19	6970	86	KT961654	
enterovirus A SV19	47210	ileum	scraping	enterovirus 19	607	92	KT961653	
enterovirus A SV19	47210	ileum	scraping	enterovirus 19	745	93	KT961653	
enterovirus A SV19	47210	proximal	scraping	enterovirus 19	7121	86	KT961654	
enterovirus A SV19	46636	ileum	scraping	enterovirus 19	3139	83	KT961654	
enterovirus A SV19	46636	ileum	scraping	enterovirus 19	3606	92	KT961653	
enterovirus A SV19	46636	proximal	scraping	enterovirus 19	7176	87	KT961654	
enterovirus A SV19	45349	distal	tissue	enterovirus 19	951	89	KT961654	
enterovirus A SV19	45349	distal	tissue	enterovirus 19	1297	85	KT961654	
enterovirus A SV19	45349	distal	tissue	enterovirus 19	872	82	KT961648	
enterovirus A SV19	45349	distal	tissue	enterovirus 19	1062	90	KT961653	
enterovirus A SV19	46776	distal	tissue	enterovirus 19	4120	91	KT961653	
enterovirus A SV19	47210	distal	tissue	enterovirus 19	6261	86	KT961653	
enterovirus A SV19	46636	proximal	tissue	enterovirus 19	1514	92	KT961653	
enterovirus A SV19	46636	proximal	tissue	enterovirus 19	1123	92	KT961653	
enterovirus A SV19	46636	transverse	tissue	enterovirus 19	1592	80	KT961654	
enterovirus A SV19	46636	transverse	tissue	enterovirus 19	3139	92	KT961653	
enterovirus A SV19	46636	transverse	tissue	enterovirus 19	1519	87	KT961654	
enterovirus A SV46	46776	distal	scraping	enterovirus 46	1133	85	KT961653	49
enterovirus A SV46	46776	proximal	scraping	enterovirus 46	2325	91	KT961655	
enterovirus A SV46	46776	transverse	scraping	enterovirus 46	2188	91	KT961655	
enterovirus A SV46	46636	proximal	scraping	enterovirus 46	754	93	KT961655	
enterovirus A SV46	46481	jejunum	tissue	enterovirus 46	3391	87	KT961658	
enterovirus A SV46	46481	stomach	scraping	enterovirus 46	2463	90	KT961658	
enterovirus A92	46776	distal	tissue	enterovirus A92	6587	84	NC001612	99
enterovirus A92	46776	distal	scraping	enterovirus A92	4661	85	KT961650	
enterovirus A92	46776	distal	scraping	enterovirus A92	1563	85	KT961646	
enterovirus A92	46676	proximal	scraping	enterovirus A92	1200	82	KT961637	
enterovirus A92	46776	transverse	scraping	enterovirus A92	3807	84	NC001612	
enterovirus A92	46481	distal	scraping	enterovirus A92	7283	85	NC001612	
enterovirus A92	46481	stomach	scraping	enterovirus A92	4530	81	NC001612	
enterovirus A92	46481	ileum	tissue	enterovirus A92	7071	85	NC001612	
enterovirus A92	46481	jejunum	tissue	enterovirus A92	2886	84	NC001612	
enterovirus J103	46776	distal	tissue	enterovirus J	1826	92	KT961657	94
enterovirus J103	46776	distal	tissue	enterovirus J	1146	90	KT961657	
enterovirus J103	46776	distal	tissue	enterovirus J	1716	92	KT961657	
enterovirus J103	46776	distal	scraping	enterovius J	2862	87	KT961657	
enterovirus J103	46776	distal	scraping	enterovius J	963	91	KT961657	
enterovirus J103	46776	distal	scraping	enterovius J	672	90	KT961657	
enterovirus J103	46776	transverse	scraping	enterovius J	7025	91	KT961657	
enterovirus J103	46481	ileum	scraping	enterovius J	651	94	KT961657	
enterovius J103	46776	proximal	scraping	enterovius J	3224	91	KT961657	
erythroparvovirus	46526	duodenum	scraping	erythroparvovirus	546	99	KT961659	81
erythroparvovirus	46526	duodenum	tissue	erythroparvovirus	1017	99	KT961659	
erythroparvovirus	46526	duodenum	tissue	erythroparvovirus	3471	99	KT961659	
protoparvovirus	46700	ileum	scraping	protoparvovirus	1035	88	NC039049	89
protoparvovirus	46700	ileum	scraping	protoparvovirus	580	73	MH645362	
protoparvovirus	46700	jejunum	tissue	protoparvovirus	659	89	NC039049	
protoparvovirus	46636	stomach	tissue	protoparvovirus	640	83	JN798211	
sapelovirus	45349	distal	scraping	sapelovirus	735	85	JX627573	73
sapelovirus	45349	distal	scraping	sapelovirus	1954	78	JX627573	
sapelovirus	45349	distal	scraping	sapelovirus	982	96	KT984501	
sapelovirus	45349	transverse	scraping	sapelovirus	1667	84	JX627573	
sapelovirus	45349	transverse	scraping	sapelovirus	1396	86	JX627573	
sapelovirus	45349	transverse	scraping	sapelovirus	580	85	JX627574	
sapelovirus	45349	transverse	tissue	sapelovirus	604	88	KT984503	
sappovirus	45629	jejunum	scraping	sappovirus	675	97	MG515475	99
sappovirus	45629	jejunum	tissue	sappovirus	7085	98	MG15475	
